# Loss of key endosymbiont genes may facilitate early host control of the chromatophore in *Paulinella*

**DOI:** 10.1016/j.isci.2022.104974

**Published:** 2022-08-17

**Authors:** Arwa Gabr, Timothy G. Stephens, Debashish Bhattacharya

**Affiliations:** 1 Graduate Program in Molecular Bioscience and Program in Microbiology and Molecular Genetics, Rutgers University, New Brunswick, NJ 08901, USA; 2 Department of Biochemistry and Microbiology, Rutgers University, New Brunswick, NJ 08901, USA

**Keywords:** Biological sciences, Genetics, Evolutionary biology

## Abstract

The primary plastid endosymbiosis (∼124 Mya) that occurred in the heterotrophic amoeba lineage, *Paulinella*, is at an earlier stage of evolution than in Archaeplastida, and provides an excellent model for studying organelle integration. Using genomic data from photosynthetic *Paulinella*, we identified a plausible mechanism for the evolution of host control of endosymbiont (termed the chromatophore) biosynthetic pathways and functions. Specifically, random gene loss from the chromatophore and compensation by nuclear-encoded gene copies enables host control of key pathways through a minimal number of evolutionary innovations. These gene losses impact critical enzymatic steps in nucleotide biosynthesis and the more peripheral components of multi-protein DNA replication complexes. Gene retention in the chromatophore likely reflects the need to maintain a specific stoichiometric balance of the encoded products (e.g., involved in DNA replication) rather than redox state, as in the highly reduced plastid genomes of algae and plants.

## Highlights

• Endosymbiont DNA replication cannot be completed without several key host proteins
• Endosymbiont nucleotide biosynthesis is completed by import of host proteins
• Limited gene loss allowed the host to gain control of endosymbiont division
• *Paulinella* regulates chromatophore function using the stringent response pathway

## Graphical abstract

**Figure:**
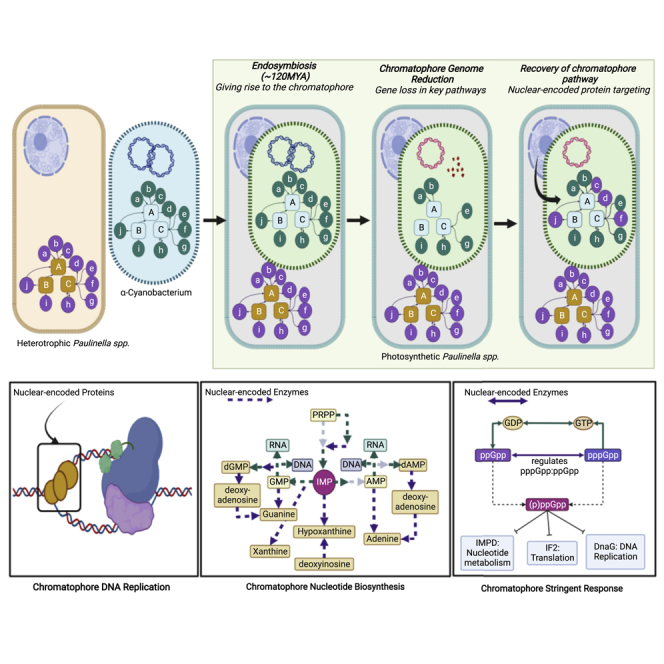

## Introduction

Although exceedingly rare, primary endosymbiosis has played a significant role in the evolution of life on our planet. By enabling eukaryotic cells to acquire prokaryotic functions in discrete compartments (organelles), endosymbiosis radically alters host cell biology, allowing these lineages to dominate a vast array of new, previously inaccessible, niches. Organellogenesis also laid the foundation for the radiation of novel divergent lineages, the most notable of which is eukaryotes. To date, there are only two known events of primary plastid endosymbiosis involving a non-photosynthetic protist engulfing a cyanobacterium. The first of these occurred 1.6–2.1 billion years ago in the ancestor of the Archaeplastida and gave rise to the canonical plastid (photosynthetic organelle) found in all algae and land plants (Strassert et al., 2021; Yoon et al., 2004). Over time, the plastid, which was transferred multiple times to other non-photosynthetic lineages via secondary and tertiary endosymbiosis, resulted in an astoundingly diverse range, both in form and function, of eukaryotic phototrophs. The other more recent case of primary endosymbiosis (ca. 124 Mya) gave rise to a novel photosynthetic organelle, termed the chromatophore, in the amoeba lineage *Paulinella* (Lhee et al., 2021).

The forces that drive endosymbiotic relationships and the formation of an organelle from a permanent endosymbiont are not yet fully understood. However, all endosymbiotic events appear to have specific landmarks (Gabr et al., 2020; Martin et al., 2002; Schleiff and Becker, 2011; Timmis et al., 2004), with the most notable being reduction of the endosymbiont genome following its internalization (Nowack et al., 2008; Reyes-Prieto et al., 2010). This process, believed to be driven by Muller’s ratchet acting on the captured cell that is unable to undergo recombination (Martin and Herrmann, 1998), is undoubtedly a driving force in the formation of endosymbiosis and is likely required to establish permanency and host control over the endosymbiont. The genome of the canonical plastid in Archaeplastida is highly reduced (compared to free-living cyanobacteria), with a size range of ca. 80–200 kbp and ∼600–1000 genes having been relocated to the nuclear genome (Nowack and Weber, 2018; Ponce-Toledo et al., 2019). In contrast, the chromatophore in photosynthetic *Paulinella* is at an intermediate stage of reduction, with a genome size of 1 Mbp and only ∼40–50 genes having been relocated to the nuclear genome (Lhee et al., 2021; Nowack et al., 2011, 2016; Nowack, 2014). However, the loss of genes from the chromatophore genome related to essential biosynthetic pathways, including amino acid metabolism, nucleotide metabolism, and enzyme cofactors (Gabr et al., 2020; Lhee et al., 2021; Nowack et al., 2008, 2016; Singer et al., 2017), makes this compartment reliant on the host for the production and provisioning of the missing proteins, placing the chromatophore firmly under host control.

The chromatophore, which is vertically transmitted to daughter cells, is a *bona fide* organelle, with neither the host nor the endosymbiont capable of surviving independently (Kies, 1974; Kies and Kremer, 1979; Nowack et al., 2008, 2016). Previous work with photosynthetic *Paulinella* suggests that many of the genes involved in rescuing chromatophore functions are of host origin, with only a few derived from endosymbiotic or horizontal gene transfer (EGT, HGT, respectively) (Lhee et al., 2021; Singer et al., 2017). Similar to the canonical plastid, photosynthetic *Paulinella* has evolved a novel transit peptide (crTP, ∼200 aa in length; which makes it highly distinctive and straightforward to identify) that targets proteins for transport into the chromatophore and is critical for the integration of host and endosymbiont metabolism (Lhee et al., 2021; Singer et al., 2017). Many nuclear-encoded proteins that compensate for genes lost from the chromatophore genome contain a crTP, demonstrating how remodeling of the host nuclear genome, in parallel with endosymbiont genome reduction, is vital for the evolution of endosymbiosis. These observations suggest that loss of function and host compensation seem to be the driving forces in establishing permanency during the transition of the endosymbiont to an organelle. Thus, photosynthetic *Paulinella* is an important model for understanding complex host-endosymbiont interactions and the rules that govern the earlier stages of organellogenesis.

Key functions associated with the endosymbiont can be broadly classified into two types, those that are novel to the host (i.e., are encoded only in the endosymbiont genome) and those that are redundant (i.e., are encoded in both host and endosymbiont genomes). Whereas the fate of both classes of genes (i.e., functions) is to become tightly integrated in host biology, the evolutionary trajectories and constraints posed by each class to the host are unique. The ‘chassis and engine’ model (Stephens et al., 2021), which is based on observations of the *Paulinella* system, describes the challenges associated with the integration and control of novel, highly efficient, and often chromatophore-specific functions into host biology. A model has not yet been proposed for how control of redundant functions, such as nucleotide metabolism and DNA replication (among others), evolves within a permanent endosymbiosis, specifically, how these functions are synchronized across the different compartments in which they are active.

In plants and algae, all proteins involved in plastid DNA replication (e.g., DNA polymerases, DNA primase, and DNA helicases) are nuclear-encoded, with the exception of the DnaB helicase in some algae (Hirakawa and Watanabe, 2019). These data suggest that host control over DNA replication is an essential step to establish permanency of the organelle. In *Paulinella chromatophora*, the majority of genes involved in DNA replication, including DNA helicase (*dnaB*), the single-strand binding protein SSB, DNA primase (*dnaG*), gyrase (*gyrA* and *gyrB*), topoisomerase (*topA*), the replication initiation protein dnaA, and a set of DNA polymerase III subunits (Nowack et al., 2008) are still encoded in the chromatophore genome. Other genes related to genetic information processing, including those involved in DNA replication and repair, such as DNA polymerase I-like (*polA*) and NAD-dependent DNA ligase (Kustka et al., 2014), are highly enriched among the set of proteins that contain a crTP in *Paulinella micropora* strain KR01 (Lhee et al., 2021). Lhee et al. (2021) also showed that genes annotated with functions related to nucleotide metabolism were among the “ancestral set” of chromatophore-targeted proteins in the photosynthetic *Paulinella* lineage, that is, the set of nuclear-encoded proteins that were retargeted to the chromatophore using a crTP before the split of photosynthetic *Paulinella* species (ca. ∼ 60 Ma). There is no evidence of subsequent nucleotide biosynthesis-related gene transfers to the nuclear genome after the divergence of these two species (Lhee et al., 2021). The significance and timing of these gene transfer events remain unclear, as is the role of host control of DNA replication and nucleotide biosynthesis in organelle establishment. We hypothesized that the targeting of nuclear-encoded DNA replication and nucleotide biosynthesis proteins to the chromatophore might have allowed the host amoeba to gain control over the division and biosynthetic functions of the endosymbiont during the early stages of endosymbiosis. To test this idea, we used bioinformatic approaches to reconstruct the chromatophore DNA replication and nucleotide biosynthesis pathways using genes encoded in the chromatophore and nuclear genomes. We demonstrate that the host controls key steps in the DNA replication and nucleotide biosynthesis pathways and speculate that this provides control over these functions and regulation of the division of the endosymbiont compartment, which are critical steps in photosynthetic organelle evolution.

## Results

### Nuclear and chromatophore encoded *P. micropora* KR01 genes annotated with KEGG orthologs

An initial screening (using KAAS; KEGG Automatic Annotation Server) identified 209 *P.* *micropora* KR01 proteins, 166 nuclear-encoded and 43 chromatophore-encoded (Table S1), annotated with KEGG Orthologs that function within the bacterial DNA replication (ko03030) complex, purine metabolism (map00230) or pyrimidine metabolism (map00240) pathways; five enzymes from the histidine metabolism pathway [map00340] were also analyzed. The nuclear-encoded gene models were examined and corrected, when appropriate, using the available RNA-seq data (Lhee et al., 2021). A total of 142 nuclear-encoded proteins were identified (Table S2) which are expressed, free from apparent mispredictions, and share homology with one of the KEGG Orthologs of interest in this study.

### Biosynthesis of endosymbiont nucleotide precursor molecules is controlled by the host

**Figure 1:**
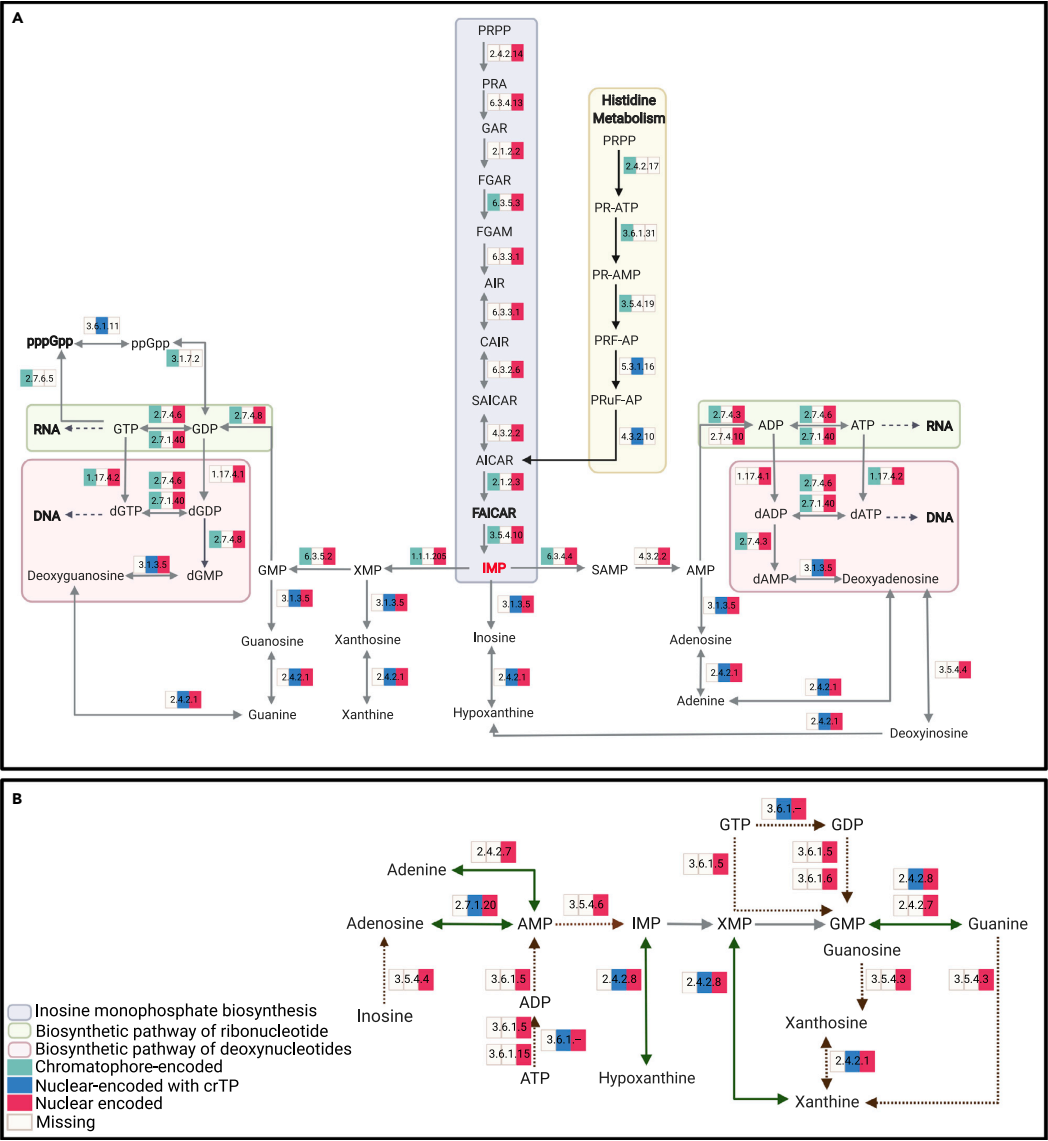
The purine metabolism pathway in *P. micropora* KR01 Diagram of the purine metabolism pathway separated into (A) *de novo* biosynthesis and (B) salvage and degradation reactions. The colored boxes associated with each enzymatic reaction show proteins that are chromatophore-encoded (green), nuclear-encoded (without a crTP; red), or nuclear-encoded with a crTP (blue). A colored box indicates that at least one annotated gene associated with that enzymatic step meets the specified definition. The figure was created with BioRender.com.

Inosine monophosphate (IMP) is the first ribonucleotide synthesized and the precursor molecule in *de novo* purine biosynthesis. In *P. micropora* KR01, all genes required for IMP biosynthesis are encoded in the nuclear genome (gray box in Figure 1A). The only IMP biosynthesis genes present in the chromatophore are those involved in the conversion of FGAR [N-Formylglycinamide ribonucleotide] to FGAM [5′-Phosphoribosyl-N-formylglycinamidine] (EC 6.3.5.3) and AICAR [1-(5′-Phosphoribosyl)-5-amino-4-imidazolecarboxamide] to IMP via FAICAR [1-(5′-Phosphoribosyl)-5-formamido-4-imidazolecarboxamide] (EC 2.1.2.3 and 3.5.4.10; both reactions are catalyzed by proteins from K00602). The only other way in which IMP synthesis can occur in the chromatophore is through the generation of AICAR [1-(5′-Phosphoribosyl)-5-amino-4-imidazolecarboxamide] as part of the histidine metabolism pathway (yellow box in Figure 1A). The first three steps in this pathway are carried out by enzymes that are exclusively chromatophore-encoded, whereas the remaining two reactions are done by enzymes that are single copy in the nuclear genome and contain a crTP (Table 1). These results suggest that the host amoeba controls synthesis of IMP in the cell.

**Table 1:** Summary of genes annotated with KO numbers associated with each major enzyme in the purine metabolism pathway

	EC No.	KO No.	Gene ID	Localization (transit pep.)	Origin	*Synechococcus* sp. WH5701 Proteins	Gene ID *P. chromatophora*
**Nuclear and chromatophore encoded**	1.1.1.205	K00088	MSTRG.5217.1.p1	Nuclear	Eukryotic	EAQ75021	m.38036
			APP88521.1	Chromatophore			PCH_840764_841927
	1.17.4.2	K00527	MSTRG.18906.1.p1	Nuclear	Bacterial	–	–
		K00524	APP88576.1	Chromatophore		–	PCH_911314_913644
	2.1.2.3 & 3.5.4.10	K00602	MSTRG.20503.1.p1	Nuclear (mtTP)	Eukryotic	EAQ73602;EAQ75486	m.37772
			APP88285.1	Chromatophore			PCH_581624_583204
	2.7.1.40	K00873	MSTRG.23381.1.p1^b^	Nuclear (mtTP)	Uncertain	EAQ74576	m.31965, m.36553, m.41188
			MSTRG.22796.1.p1^b^	Nuclear	Eukryotic		
			MSTRG.13414.1.p1	Nuclear (mtTP)	Eukryotic		
			MSTRG.24199.1.p1	Nuclear	Eukryotic		
			APP87965.1	Chromatophore			PCH_198293_200050
	2.7.4.3	K00939	MSTRG.8290.1.p1	Nuclear	Eukryotic	EAQ76717	m.91075, m.131899, m.46966, m.27298
			MSTRG.13579.1.p1	Nuclear	Eukryotic		m.27298
			MSTRG.15534.1.p1	Nuclear	Eukryotic		m.131899
			MSTRG.2607.1.p1	Nuclear	Eukryotic		m.64390
			MSTRG.1642.2.p1	Nuclear (mtTP)	Eukryotic		m.146726
			APP88343.1	Chromatophore			PCH_652542_653090
		K18532	MSTRG.8083.1.p1	Nuclear	Eukaryotic	–	m.91398
	2.7.4.6	K00940	MSTRG.7674.2.p1	Nuclear	Eukryotic	EAQ76583	m.143874
			MSTRG.13723.1.p1	Nuclear (mtTP)	Eukryotic		m.102663
			APP88146.1	Chromatophore			PCH_406754_407299
	2.7.4.8	K00942	MSTRG.12344.1.p1	Nuclear	Eukryotic	EAQ74985	m.86957
			APP88506.1	Chromatophore			PCH_828448_829026
	6.3.4.4	K01939	MSTRG.25312.1.p1^b^	Nuclear	Eukaryotic	EAQ73482;EAQ74936	m.54505
			MSTRG.20131.1.p1^b^	Nuclear (mtTP)	Eukaryotic		
			APP88625.1	Chromatophore			PCH_972494_973807
	6.3.5.2	K01951	MSTRG.17803.1.p1	Nuclear	Eukaryotic	EAQ75767	m.35054
			APP88209.1	Chromatophore			PCH_487179_488762
	6.3.5.3	K01952	MSTRG.834.1.p1	Nuclear	Uncertain	–	m.13621
		K23265	APP87930.1	Chromatophore		EAQ73739	–
		K23269	APP88186.1	Chromatophore		EAQ75725	–
**Chrom. encoded**	2.4.2.17	K00765	APP88666.1	Chromatophore		EAQ74750	PCH_1018315_1018971
	2.7.6.5 & 3.1.7.2	K01139	APP88130.1	Chromatophore		EAQ76636	PCH_386204_387925
	3.5.4.19 & 3.6.1.31	K11755	APP87820.1	Chromatophore		EAQ74855	PCH_10921_11655
**Nuclear encoded**	1.17.4.1	K10807	MSTRG.3989.1.p1	Nuclear	Eukaryotic	–	m.19028
		K10808	MSTRG.12045.1.p1	Nuclear	Eukaryotic	–	m.79073, m.89037, m.148939, m.53968
	2.1.2.2	K11175	MSTRG.838.1.p1	Nuclear	Bacterial	EAQ74543	m.95502
	2.4.2.1	K03783	MSTRG.4695.1.p1^b^	Nuclear	Eukaryotic	–	m.81060, m.70572
		K09913	MSTRG.6408.1.p1	Nuclear	Uncertain	–	–
			MSTRG.6409.1.p1	Nuclear (crTP)	Uncertain		
	2.4.2.7	K00759	MSTRG.6689.1.p1	Nuclear	Eukaryotic	EAQ74398	m.69775
	2.4.2.8	K00760	MSTRG.27853.2.p1	Nuclear (crTP)	Eukryotic	–	m.60233 (crTP), m.96498, m.21561, m.103930
			MSTRG.15480.1.p1^b^	Nuclear	Eukryotic		
			MSTRG.17958.1.p1^b^	Nuclear	Eukryotic		
	2.4.2.14	K00764	MSTRG.835.1.p1	Nuclear	Eukaryotic	EAQ75726	m.33221
	2.7.1.20	K00856	MSTRG.24012.1.p1^b^	Nuclear (crTP)	Eukryotic	–	m.46151 (crTP), m.43287, m.47560
			MSTRG.18860.1.p1^a^	Nuclear	Eukryotic		
	2.7.4.10	K00944	MSTRG.11129.2.p1	Nuclear	Eukryotic	–	m.85710, m.63610
			MSTRG.23120.1.p1	Nuclear	Eukryotic		
	3.1.3.5	K01081	MSTRG.17384.1.p1	Nuclear	Eukryotic	–	m.15216, m.102769, m.22838, m.28026, m.42900, m.63166
			MSTRG.27764.1.p1	Nuclear	Eukryotic		
		K11751	MSTRG.831.1.p1	Nuclear	Eukaryotic	–	m.106789, m.5449, m.145463
			MSTRG.19222.1.p1	Nuclear	Eukaryotic		
		K24242	MSTRG.21922.1.p1	Nuclear (crTP)	Eukaryotic	–	m.63166 (crTP)
			MSTRG.16505.1.p1	Nuclear	Eukaryotic		
	3.5.4.3	K01487	MSTRG.9046.t1.1.p1^b^	Nuclear	Uncertain	–	–
	3.5.4.4	K01488	MSTRG.10333.1.p1	Nuclear	Eukaryotic	–	m.41872
			MSTRG.21591.1.p1^b^	Nuclear	Eukaryotic	–	m.47071
			MSTRG.21594.1.p1^b^	Nuclear	Eukaryotic	–	m.59944
			MSTRG.13131.1.p1	Nuclear	Eukaryotic	–	m.68534
			MSTRG.13131.6.p1	Nuclear	Eukaryotic	–	
			MSTRG.4653.2.p1	Nuclear	Eukaryotic	–	
			MSTRG.26161.6.p1^b^	Nuclear	Eukaryotic	–	
	3.5.4.6	K01490	MSTRG.9371.1.p1	Nuclear	Eukaryotic	–	m.73797, m.27282, m.160032
			MSTRG.16814.2.p1	Nuclear	Eukaryotic	–	
	3.6.1.5	K01510	MSTRG.21143.1.p1	Nuclear	Eukaryotic	–	m.107005, m.139053
	3.6.1.6	K12304	MSTRG.971.1.p1	Nuclear	Eukaryotic	–	m.93542, m.61014
			MSTRG.2459.4.p1	Nuclear	Eukaryotic	–	
	3.6.1.11	K01514	MSTRG.13432.1.p1	Nuclear (crTP)	Eukaryotic	–	m.41082 (crTP), m.56304
	3.6.1.15	K06928	MSTRG.15872.1.p1	Nuclear	Eukaryotic	–	–
	3.6.1.-	K01519	MSTRG.11874.1.p1	Nuclear (crTP)	Eukaryotic	–	m.41123 (crTP)
			MSTRG.24583.1.p1	Nuclear	Eukaryotic		m.104136
	4.3.2.2	K01756	MSTRG.6096.1.p1	Nuclear	Eukaryotic	EAQ76193	m.90258, m.131755, m.52838, m.53423
			MSTRG.9958.1.p1	Nuclear (mtTP)	Eukaryotic		
	4.3.2.10	K01663	MSTRG.5172.1	Nuclear (crTP)	Eukaryotic	–	m.24429 (crTP)
	5.3.1.16	K01814	MSTRG.7507.1	Nuclear (crTP)	Eukaryotic	EAQ73877	m.45228 (crTP)
	6.3.2.6	K01923	MSTRG.7998.1.p1	Nuclear	Eukaryotic	EAQ76110	m.80556, m.62628
	6.3.3.1	K01933	MSTRG.9541.1.p1	Nuclear	Eukaryotic	EAQ76774	m.40437
	6.3.4.13	K01945	MSTRG.11290.1.p1^b^	Nuclear	Uncertain	EAQ76112	m.48871

Chromatophore transit peptides (crTP); mitochondrial transit peptides (mtTP).

a Possible large insertion that disrupts the functional region.

b Protein is 3′ or 5′ partial.

**Figure 2:**
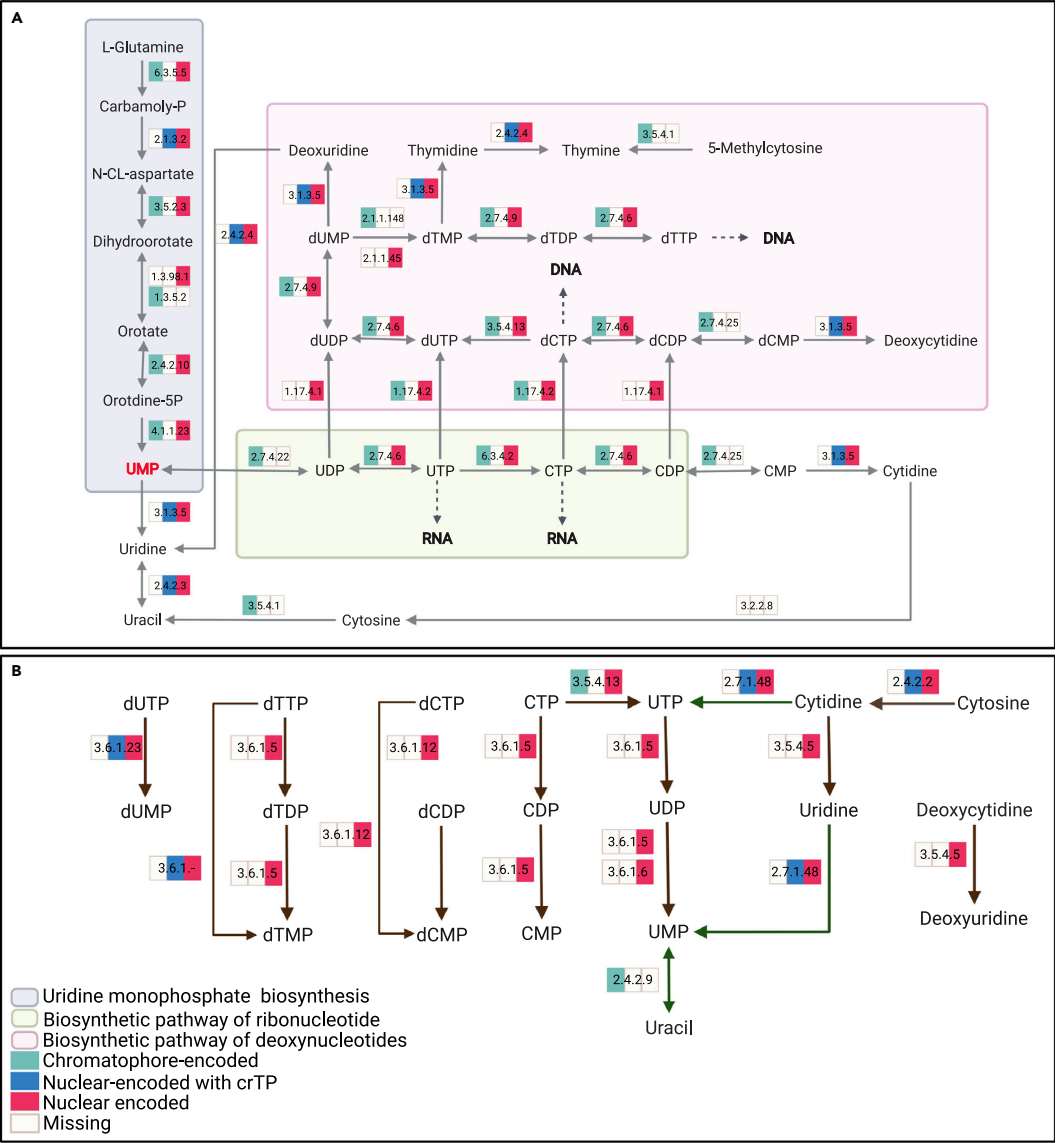
The pyrimidine metabolism pathway in *P. micropora* KR01 Diagram of the pyrimidine metabolism pathway separated into (A) *de novo* biosynthesis and (B) salvage and degradation reactions. The colored boxes associated with each enzymatic reaction show proteins that are chromatophore-encoded (green), nuclear-encoded (without a crTP; red), or nuclear-encoded with a crTP (blue). A colored box indicates that at least one annotated gene associated with that enzymatic step meets the specified definition. The figure was created with BioRender.com.

Uridine monophosphate (UMP) is the first nucleotide synthesized as part of the pyrimidine metabolism pathway and is the precursor molecule for the synthesis of thymidine, uracil, and cytosine. There are seven enzymatic reactions involved in the synthesis of UMP from L-glutamine (Figure 2A). In *P. micropora* KR01, four of these reactions are encoded by genes present in both the nuclear and chromatophore genomes, two additional reactions, EC 1.3.5.2 and 1.3.98.1, catalyze the same step in the pathway and are encoded in the chromatophore and nuclear genomes, respectively. This suggests that five out of the six UMP synthesis steps are encoded in both genomes. The one reaction step, catalyzed by EC 2.1.3.2, is annotated to three nuclear-encoded genes (Table 2), one of which has a crTP. This suggests that although most genes involved in the synthesis of UMP in the cell are encoded in both genomes, and are localized to both compartments, the host amoeba has control over this pathway in the chromatophore.

**Table 2:** Summary of genes annotated with KO numbers associated with each major enzyme in the pyrimidine metabolism pathway

	EC No.	KO No.	Gene ID	Localization (transit pep.)	Origin	*Synechococcus* sp. WH5701 Proteins	Gene ID *P. chromatophora*
Nuclear and chromatophore encoded	1.17.4.2	K00527	MSTRG.18906.1.p1	Nuclear	Bacterial	–	–
		K00524	APP88576.1	Chromatophore		–	PCH_911314_913644
	2.4.2.10	K13421	MSTRG.5317.1.p1	Nuclear	Eukaryotic	–EAQ74889;EAQ76532	m.76923, m.59611
		K00762	APP88279.1	Chromatophore			PCH_574488_575072
	2.7.4.6	K00940	MSTRG.7674.2.p1	Nuclear	Eukaryotic	EAQ76583	m.102663, m.143874
			MSTRG.13723.1.p1	Nuclear (mtTP)	Eukaryotic		
			APP88146.1	Chromatophore			PCH_406754_407299
	2.7.4.9	K00943	MSTRG.9240.1.p1	Nuclear	Eukaryotic	EAQ75333	m.93602, m.114252
			APP88085.1	Chromatophore			–
	3.5.4.13	K01494	MSTRG.7644.1.p1	Nuclear	Eukaryotic	EAQ75459;EAQ76479	–
			APP88297.1	Chromatophore			PCH_596488_597081
	4.1.1.23	K13421	MSTRG.5317.1.p1	Nuclear	Eukaryotic	–	m.76923, m.59611
		K01591	APP88463.1	Chromatophore		EAQ76096	PCH_781998_782753
	6.3.4.2	K01937	MSTRG.26400.1.p1	Nuclear	Eukaryotic	EAQ76380	m.39956
			APP88166.1	Chromatophore			PCH_436530_438125
	6.3.5.5 & 3.5.2.3	K11540	MSTRG.13416.1.p1^a^	Nuclear	Eukaryotic	–	m.4707
		K11541	MSTRG.9073.1.p1^a^	Nuclear	Eukaryotic	–	m.143618
		K01955	APP88558.1	Chromatophore		EAQ73983	PCH_890549_893851
		K01956	APP88018.1	Chromatophore		EAQ75106	PCH_250855_252036
Chromatophore encoded	1.3.5.2	K00254	APP88135.1	Chromatophore		EAQ76618	PCH_393519_394685
	2.1.1.148	K03465	APP88298.1	Chromatophore		–	PCH_597091_597813
	2.4.2.9	K00761	APP88061.1	Chromatophore		EAQ73733	PCH_305134_305784
	2.7.4.22	K09903	APP88634.1	Chromatophore		EAQ74917	PCH_983960_984673
	2.7.4.25	K13799	APP88384.1	Chromatophore		EAQ76776	PCH_688242_689084
	3.5.4.1	K01485	APP87895.1	Chromatophore		EAQ73641;EAQ74433	PCH_89917_91152
Nuclear encoded	1.3.98.1	K00226	MSTRG.1224.1.p1^a^	Nuclear	Eukaryotic	EAQ74251	m.72251
			MSTRG.1225.1.p1	Nuclear	Eukaryotic		
	1.17.4.1	K10807	MSTRG.3989.1.p1	Nuclear	Eukaryotic	–	m.19028
		K10808	MSTRG.12045.1.p1	Nuclear	Eukaryotic	–	m.79073, m.89037, m.148939, m.53968
	2.1.1.45	K13998	MSTRG.21233.1.p1	Nuclear	Eukaryotic	–	m.50936
			MSTRG.27860.1.p1^a^	Nuclear	Eukaryotic	–	
	2.1.3.2	K00609	MSTRG.21874.1.p1^a^	Nuclear (crTP)	Uncertain	EAQ75419	m.86993, m.62292 (crTP)
		*K11540*	MSTRG.13416.1.p1^a^	Nuclear	Eukaryotic	–	m.4707
		*K11541*	MSTRG.9073.1.p1^a^	Nuclear	Eukaryotic	–	–
	2.4.2.2 & 2.4.2.3 & 2.4.2.4	K09913	MSTRG.6408.1.p1	Nuclear	Uncertain	–	–
			MSTRG.6409.1.p1	Nuclear (crTP)	Uncertain		
	2.7.1.48	K00876	MSTRG.28031.1.p1	Nuclear (crTP)	Eukaryotic	–	m.65065
			MSTRG.25663.1.p1	Nuclear	Bacteria		
	3.1.3.5	K01081	MSTRG.17384.1.p1	Nuclear	Eukaryotic	–	m.15216, m.102769, m.22838, m.28026, m.42900, m.63166
			MSTRG.27764.1.p1	Nuclear	Eukaryotic		
		K11751	MSTRG.831.1.p1	Nuclear	Eukaryotic	–	m.106789, m.5449, m.145463
			MSTRG.19222.1.p1	Nuclear	Eukaryotic		
		K24242	MSTRG.21922.1.p1	Nuclear (crTP)	Eukaryotic	–	m.63166 (crTP)
			MSTRG.16505.1.p1	Nuclear	Eukaryotic		m.22838
	3.5.4.5	K01489	MSTRG.23658.1.p1^a^	Nuclear	Eukaryotic	–	m.58556
	3.6.1.5	K01510	MSTRG.21143.1.p1	Nuclear	Eukaryotic	–	m.107005, m.139053
	3.6.1.6	K12304	MSTRG.971.1.p1	Nuclear	Eukaryotic	–	m.93542, m.61014
			MSTRG.2459.4.p1	Nuclear	Eukaryotic		
	3.6.1.12	K16904	MSTRG.9895.1.p1	Nuclear	Eukaryotic	–	m.73914
	3.6.1.-	K01519	MSTRG.11874.1.p1	Nuclear (crTP)	Eukaryotic	–	m.41123 (crTP)
			MSTRG.24583.1.p1	Nuclear	Eukaryotic		m.104136
	3.6.1.23	K01520	MSTRG.27457.1.p1	Nuclear (crTP)	Eukaryotic	–	m.87559 (crTP)
			MSTRG.19767.1.p1	Nuclear	Eukaryotic		m.118837, m.201858, m.56738

KO numbers in italics are associated with multiple enzyme reactions within the Pyrimidine Metabolism pathway.

Chromatophore transit peptides (crTP); mitochondrial transit peptides (mtTP).

a Protein is 3′ or 5′ partial.

### Purine biosynthesis is under host control

Enzymatic reactions for the synthesis of the ribonucleotides GTP (Guanosine 5′-triphosphate) (via, XMP [Xanthylic acid], GMP [Guanosine monophosphate], and GDP [Guanosine diphosphate]) and ATP (Adenosine 5′-triphosphate) (via, SAMP [Adenylosuccinate], AMP [Adenosine 5′-monophosphate], and ADP [Adenosine 5′-diphosphate]) from IMP are all encoded by genes (none of which have a crTP) found in both the nuclear and chromatophore genomes, with the only exception being the enzyme reaction EC 4.3.2.2, which converts SAMP to AMP. This function is annotated to two nuclear-encoded genes, neither of which encode a crTP, although one has a predicted mtTP (Table 1). Additional analysis aimed at confirming if genes associated with a given enzymatic reaction are indeed missing for a particular genome, did not turn up any evidence of genes associated with 10.13039/501100000780EC 4.3.2.2 in the chromatophore genome (Table S3), further supporting its nuclear localization. The enzymatic reactions for the generation of deoxynucleotides through the conversion of GTP to dGMP (via dGTP and dGDP) and ATP to dAMP (via dATP and dADP) are also encoded by genes in both genomes. The reactions (EC 2.4.2.1 and 3.1.3.5) involved in the production of guanine, guanosine, and deoxyguanosine, as well as adenine, adenosine, and deoxyadenosine (Figure 1A) are each annotated to multiple nuclear-encoded genes, with one gene associated with each reaction also possessing a crTP (Table 1). These enzymes also control the production of inosine, hypoxanthine, and xanthine. Moreover, all purine degradation and salvage (brown and green arrows in Figure 1B, respectively) reactions are only annotated to nuclear-encoded genes (i.e., none are annotated to chromatophore-encoded genes), with only some of the reactions assigned to genes that contain a crTP. Fourteen of the nuclear-encoded purine metabolism genes are partial (determined by visual inspection of the protein alignments [Data S1] and coverage of the top hits to sequences in UniProt [Table S2]) or have large regions of non-conserved residues that disrupt the conserved region of the protein. In all cases, there are full-length nuclear-encoded genes associated with the same reaction steps as the partial genes or there are alternative reactions that could recover that part of the pathway. Many of the partial genes are located close (<1.5 kbp) to the ends of their scaffolds, possibly explaining why they appear incomplete.

Remarkably, most of the genes associated with purine metabolism are encoded either in just the nuclear genome or both the nuclear and chromatophore genomes. The only pathways encoded exclusively in the chromatophore genome are EC 2.7.6.5 and 3.1.7.2, both of which are encoded by the same gene (APP88130.1; Table 1 and Figure 1A). These two enzyme reactions catalyze the conversion of GTP to pppGpp (EC 2.7.6.5) and the bidirectional conversion of ppGpp to/from GDP (EC 3.1.7.2). The enzyme responsible for the bidirectional conversion of pppGpp and ppGpp (EC 3.6.1.11) is annotated to a single nuclear-encoded gene (MSTRG.13432.1.p1) that is predicted to contain a crTP, suggesting that this reaction occurs in the chromatophore but is under host control because of the nuclear gene localization. MSTRG.13432.1.p1 is also clearly of eukaryotic origin (Data S1). The molecules pppGpp and ppGpp (collectively abbreviated to (p)ppGpp) are the major signaling molecules in the “stringent response” pathway, which is ubiquitous in bacteria. This gene shows a weak diurnal expression pattern under control light (Figure S1), with a slight increase in its expression in the dark compared to light, and a relatively strong response to high light, with its expression increasing over time.

### Pyrimidine biosynthesis in the chromatophore is controlled by both the host and chromatophore

In contrast to purine metabolism, some of the key enzyme reactions in the main backbone of pyrimidine metabolism are only chromatophore-encoded. EC 2.7.4.22 catalyzes the bidirectional conversion of UDP (Uridine 5′-diphosphate) to/from UMP, which is the start of the ribonucleotides (CTP [cytidine triphosphate] and UTP [uridine triphosphate]) and deoxynucleotides (dTTP [deoxythymidine triphosphate] and dCTP [deoxycytidine triphosphate]) synthesis pathway and is only encoded in the chromatophore genome (Figure 2A and Table 2). EC 2.7.4.25 and EC 2.1.1.148 are also key enzymatic reactions and are only annotated to chromatophore-encoded genes. Similar to the purine metabolism pathway, reactions that represent the final steps for some of the major products of the pathway (i.e., uridine, uracil, cytidine, deoxycytidine, thymine, thymidine, and deoxyuridine) are only encoded in the nuclear genome; in all cases one of the nuclear-encoded genes has a crTP. Surprisingly, the enzyme reaction that represents the bidirectional conversion of uracil from UMP (EC 2.4.2.9), which is part of the salvage pathway (green arrows in Figure 2B), is only encoded in the chromatophore. Aside from EC 3.5.4.13, which is encoded in both nuclear and chromatophore genomes, all other salvage and degradation pathway enzyme reactions (green and brown arrows in Figure 2B) are assigned to nuclear-encoded genes, four of these genes are predicted to have a crTP. In addition, no genes were found in either of the genomes that can catalyze the synthesis of cytosine (EC 3.2.2.8 or EC 3.2.2.10); an additional search of both genomes (Table S3) was unsuccessful in identifying any genes associated with these enzymatic reactions, suggesting that they are absent from *P. micropora* KR01. The enzyme reaction (EC 3.5.4.1) that represents the degradation of cytosine to uracil is encoded in the chromatophore genome and the reaction (EC 2.4.2.2) that represents the degradation of cytosine to cytidine is nuclear encoded with a gene copy that encodes a crTP. Eight of the pyrimidine nuclear-encoded genes in *P. micropora* KR01 are partial (Table 2) however, most are associated with reaction steps that have other annotated full-length gene copies. The only gene (MSTRG.23658.1.p1) annotated with EC 3.5.4.5 appears to be partial; the putative loss of this gene, which functions as part of the salvage and degradation pathway, would result in *P. micropora* KR01 not being able to convert deoxycytidine to deoxyuridine and cytidine to uridine, although cytidine can still be converted to UMP via alternative reaction steps. Many of these genes are located at the ends of scaffolds (<10 kbp), raising the possibility that regions are missing because of a fragmented genome assembly.

### Nuclear-encoded nucleotide metabolism genes are predominantly of eukaryotic origin

The majority of nuclear-encoded genes associated with purine biosynthesis are of eukaryotic origin, or of uncertain provenance, but do not provide strong evidence of having arisen via HGT from non-eukaryotic sources (Table 1). The strongest evidence of a bacterial HGT is for MSTRG.18906.1.p1 (Data S2), which is a ribonucleoside-triphosphate reductase (thioredoxin; K00527) gene associated with EC 1.17.4.2. The *P. micropora* KR01 sequence in this tree is separated from the major clade of eukaryotic sequences by a strongly supported node (BS = 100%) and is affiliated with Epsilonproteobacteria (BS = 100%). There are five KEGG Orthology (KO) numbers that are annotated to multiple nuclear-encoded genes and that have one of their annotated genes encoding a crTP. Four of the five KOs show grouping of all *P. micropora* KR01 sequences in the tree (Table S1; Data S1), demonstrating their origin from recent gene duplication events (i.e., in the common ancestor of photosynthetic or heterotrophic *Paulinella*). The genes annotated as K00856, which is the one KO that has *P. micropora* KR01 sequences positioned in different positions in the tree, are partial or have large insertions, potentially explaining their position in the tree. Of the five KOs, one (K00760) is annotated to three nuclear-encoded genes, whereas the remainder are annotated to only two genes each.

Consistent with the purine biosynthesis pathway, many of the nuclear-encoded pyrimidine biosynthesis genes are of eukaryotic or uncertain origin (Table 2). The clearest case for a bacterial HGT is the ribonucleoside-triphosphate reductase (K00527; EC 1.17.4.2) gene MSTRG.18906.1.p1 (Data S2), which is also part of the purine metabolism pathway. A less convincing example of HGT from bacteria is the uridine kinase (K00876; EC 2.7.1.48) gene MSTRG.25663.1.p1 (Data S3). This gene sequence diverges basal to a clade of predominantly bacterial sequence and is separated from the two major clades of eukaryotic sequences in the tree by strongly supported nodes (BS = 100%). This gene is also positioned in the tree close to single genes from *P. chromatophora* (scaffold8477-m65065) and *Paulinella ovalis* (SAG1_utg7180000024255.g11047.t1). The *P. ovalis* gene is on a scaffold (SAG1_utg7180000024255) with one other gene (SAG1_utg7180000024255.g11046.t1) that has top hits to eukaryotic sequences in the NCBI nr database (searched online Oct. 2021), suggesting that this gene may have arisen in *Paulinella* via an HGT event in the common ancestor of both the photosynthetic and heterotrophic lineages. Interestingly, of the two uridine kinase genes, the one of eukaryotic origin encodes a crTP, whereas the one of putative bacterial origin does not. There are five KO numbers (two of which are also associated with purine metabolism) that are assigned to multiple nuclear-encoded genes, with one gene encoding a crTP. The *P. micropora* KR01 genes associated with 3/5 KOs group together in their respective phylogenetic trees (Table S2) and have likely originated from recent duplication events. All five KOs have two annotated nuclear-encoded genes each.

### Some chromatophore DNA replication proteins are encoded in the nuclear genome

**Figure 3:**
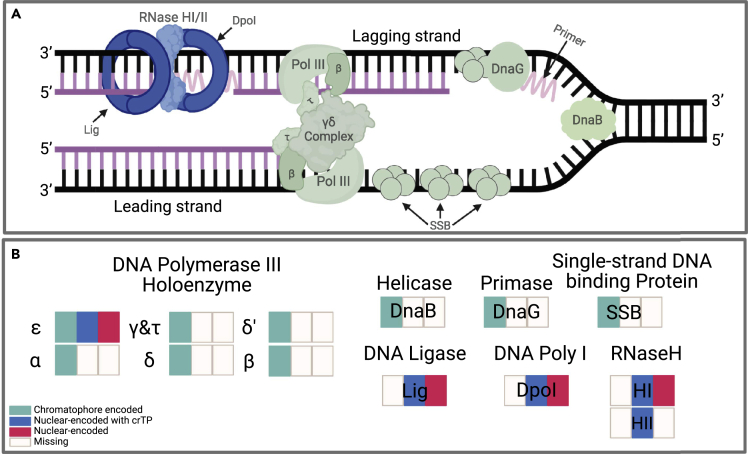
Diagram of the bacterial DNA replication and repair pathway. (A) The DNA replication complex shown along a segment of DNA with proteins that are nuclear-encoded and chromatophore targeted shown in blue and proteins that are chromatophore-encoded shown in green. (B) Proteins encoded in the chromatophore (green), nuclear (red), or nuclear with a crTP (blue) genomes. A colored box indicates that at least one annotated gene associated with that protein meets the specified definition. The figure was created with BioRender.com.

Of the 17 proteins in the KEGG bacterial DNA replication complex, 13 are annotated in *P. micropora* KR01 (Table 3) and are also all present in *Synechococcus* sp. WH5701, which is the cyanobacterial lineage most closely related to the putative chromatophore donor (Rae et al., 2013; Reyes-Prieto et al., 2010; Yoon et al., 2009). The four proteins from the bacterial DNA replication pathway that are not identified in *P. micropora* KR01 (DNA polymerase III subunits theta [holE; K02345], psi [holD; K02344], and chi [holC; K02339], and RNase HIII [rnhC; K03471]) are also absent from *Synechococcus* sp. WH5701. Of the 13 proteins present in *P. micropora* KR01, four are encoded only by genes in the nuclear genome and eight by genes in the chromatophore genome (Figure 3B; Table 3). Except for RNase HII (rnhB; K03470), each of the nuclear-encoded proteins have multiple annotated genes; only one gene associated with each of the nuclear-encoded proteins encoding a crTP. DNA polymerase III subunit epsilon (dnaQ; K02342), the only protein encoded in both genomes, has three annotated genes: one nuclear-encoded with a mitochondrial targeting peptide (mtTP), one nuclear-encoded with a crTP, and one chromatophore-encoded. Moreover, DNA ligase (ligA, ligB; K01972) and DNA polymerase I (polA; K02335) proteins also have annotated genes that contain a mtTP, in addition to gene copies that encode a crTP. Of the nuclear-encoded genes, only RNase HI (rnhA; K03469) is involved in the eukaryotic DNA replication complex, the other genes are specific to the bacterial complex.

**Table 3:** Summary of genes annotated with KO numbers associated with each protein in the bacterial DNA replication complex

	EC No.	KO No.	Name	Gene ID	Localization (transit pep.)	Origin	*Synechococcus* sp. WH5701 Proteins	Gene ID *P. chromatophora*
**Nuc. and chrom. encoded**	2.7.7.7	K02342	DNA polymerase III subunit epsilon	MSTRG.13717.1.p1	Nuclear (crTP)	Uncertain	EAQ74371	m.66968, m125241
				MSTRG.8071.1.p1	Nuclear (mtTP)	Bacterial		
				APP88013.1_204	Chromatophore			PCH_246209_247117
**Chromatophore encoded**	3.6.4.12	K02314	Replicative DNA helicase	APP88154.1_345	Chromatophore		EAQ75589	PCH_420124_421542
	2.7.7.101	K02316	DNA primase	APP88027.1_218	Chromatophore		EAQ75091	PCH_261592_263637
	2.7.7.7	K02337	DNA polymerase III subunit alpha	APP88023.1_214	Chromatophore		EAQ75099	PCH_256107_259634
	2.7.7.7	K02338	DNA polymerase III subunit beta	APP88184.1_375	Chromatophore		EAQ75723	PCH_460012_461196
	2.7.7.7	K02340	DNA polymerase III subunit delta	APP88227.1_418	Chromatophore		EAQ75796	PCH_510667_511635
	2.7.7.7	K02341	DNA polymerase III subunit delta'	APP88084.1_275	Chromatophore		EAQ75332	PCH_333304_334296
	2.7.7.7	K02343	DNA polymerase III subunit gamma/tau	APP88218.1_409	Chromatophore		EAQ75781	PCH_497417_499171
	–	K03111	Single-strand DNA-binding protein	APP88236.1_427	Chromatophore		EAQ75861	PCH_521937_522311
**Nuclear encoded**	6.5.1.2	K01972	DNA ligase	MSTRG.15911.1.p1	Nuclear (crTP)	Eukaryotic	EAQ75604	m.13705 (crTP)
				MSTRG.8876.1.p1	Nuclear (mtTP)	Eukaryotic		m.92521, m.34888, m.39298
				MSTRG.7660.1.p1	Nuclear	Eukaryotic		
	2.7.7.7	K02335	DNA polymerase I	MSTRG.17738.1.p1	Nuclear	Eukaryotic	EAQ74696	m.105893, m.106755 (truncated), m.25805, m.133252, m.8010 (truncated), m.41193, m.183634 (truncated), m.52383 (+150aa)
				MSTRG.27742.1.p1	Nuclear (crTP)	Maybe-Bacterial		
				MSTRG.6340.1.p1	Nuclear (mtTP)	Eukaryotic		
				MSTRG.4007.1.p1	Nuclear	Eukaryotic		
				MSTRG.13387.1.p1	Nuclear	Uncertain		
	3.1.26.4	K03469	RNase HI	MSTRG.11892.1.p1	Nuclear	Uncertain	EAQ76617	m.71861
				MSTRG.19480.1.p1^a^	Nuclear (crTP)	Uncertain		m.147684 (truncated)
	3.1.26.4	K03470	RNase HII	MSTRG.7053.1.p1^a^	Nuclear (crTP)	Uncertain	EAQ75396	m.103917 (truncated), m.150289 (truncated), m.59563 (truncated)

Chromatophore transit peptides (crTP); mitochondrial transit peptides (mtTP).

a Partial crTP.

### Nuclear-encoded chromatophore DNA replication proteins are of eukaryotic origin

Phylogenetic analysis of the nuclear-encoded bacterial DNA replication proteins (Table 3) was undertaken to identify their provenance in *Paulinella*. Of the 13 nuclear-encoded genes, 11 were of eukaryotic or uncertain origin and two were of putative bacterial origin. One of the putative bacterial genes (MSTRG.27742.1.p1), encoding a DNA polymerase I (K02335), is positioned in a well-supported (BS ≥ 95%) clade of mostly bacteria (Betaproteobacteria) and some eukaryotes (Data S4). This gene encodes a crTP and is positioned near two *P. ovalis* proteins. The scaffolds (SAG1_utg7180000008502 and SAG1_utg7180000023942) that encode the two *P. ovalis* proteins in Data S4 are short (3,085 bp and 1,012 bp, respectively) but encode a few other genes that all have top hits to proteins from *Pseudoalteromonas* spp. (Gammaproteobacteria). This result suggests that these contigs, and the proteins they encode, are from *P. ovalis*-associated bacteria. The MSTRG.27742.1.p1 protein groups with Betaproteobacteria in the tree, separate from the bacterium-derived *P. ovalis* sequences (which groups with Gammaproteobacteria), however, the presence of these sequences demonstrates that the *P. ovalis* genes associate with Proteobacteria, which is the putative donor lineage of this gene in *P. micropora* KR01.

The two DNA polymerase III subunit epsilon (K02342) proteins are in a tree (Data S5) composed primarily of bacterial homologs, with a limited number of eukaryotic and viral sequences spread throughout. MSTRG.13717.1.p1, which contains a crTP, is positioned in the tree with a eukaryotic sequence and has no significant hits to bacterial sequences in the database used for phylogenetic analysis. Manual inspection of MSTRG.13717.1.p1 reveals that it contains significant stretches of serine residues (Figure S2) that are not present in MSTRG.8071.1.p1 (which encodes a mtTP and is likely of bacterial origin) or the five sequence (all bacterial) from the NCBI nr database with the highest scoring hits to MSTRG.8071.1.p1. The long region that is predicted between the crTP and the conserved section of the protein (Figure S2), and the regions of repeated serine residues in the protein, are supported by the RNA-seq data making them unlikely to be artifacts of mis-assembly or misprediction. There is a high frequency of serine residues around the predicted crTP cleavage site (Oberleitner et al., 2022), suggesting that these serine repeat regions may have evolved to facilitate the function of the crTP, although this remains to be further tested. It is also unknown if the additional sequence in the protein affects its function however, it is noteworthy that DNA polymerase III subunit epsilon in *P. micropora* KR01 is the only subunit encoded in both genomes. Two proteins, MSTRG.19480.1.p1 and MSTRG.7053.1.p1 (the only gene annotated as a RNase HII [K03470] protein), encode partial crTP sequences. These proteins encode the terminal 26.6 and 37.9% of the crTP motif (respectively); the functional consequence of this is unknown.

### Only some of the nuclear-encoded bacterial DNA replication genes are diurnally regulated

Of the five bacterial DNA replication proteins that have nuclear-encoded genes, two (DNA polymerase III subunit epsilon, and RNase HI) are encoded by genes with a crTP that follow a diurnal expression cycle (with higher expression levels during the dark when compared to the light periods; Figure S3). The DNA ligase and DNA polymerase I proteins have annotated genes that also follow a diurnal pattern, however, these genes are not the copies that encode a crTP (in both cases the crTP-encoding genes have low expression values that do not vary across the time points). The one gene annotated as a RNase HII protein increases over the sampled timepoint but does not follow any obvious patterns. The DNA polymerase III subunit epsilon, DNA ligase, and DNA polymerase I proteins each have one annotated gene that encodes a mtTP (Table 3). These mtTP-encoding genes follow a diurnal expression pattern (Figure S3), suggesting that DNA replication in the mitochondrion occurs primarily at night in *Paulinella*.

### DNA replication and nucleotide biosynthesis genes in *P. chromatophora*

To estimate the relative timing of gene loss from the chromatophore, we analyzed DNA replication and nucleotide biosynthesis pathways in the available *P. chromatophora* CCAC0185 nuclear (Nowack et al., 2016) and chromatophore (Nowack et al., 2008) proteomes. The distribution of genes encoding enzymes involved in purine biosynthesis in *P. chromatophora* CCAC0185 was very similar to that of *P. micropora* KR01 (Table 1). All proteins that were not detected in the chromatophore genome of *P. micropora* KR01 were also not detected in the chromatophore genome of *P. chromatophora* CCAC0185. Similarly, proteins that were reported to be nuclear encoded with a crTP in *P. micropora* KR01 were also shown (except for EC 2.4.2.1) to contain a crTP in *P. chromatophora* CCAC0185 (Table 1). There are three enzyme genes (EC 1.17.4.2, EC 3.5.4.3, and EC 3.6.1.15) detected in the nuclear genome of *P. micropora* KR01 that were not detected in the nuclear genome of *P. chromatophora* CCAC0185. Moreover, one enzyme (EC 6.3.5.3) was not detected in the chromatophore genome, even though a nuclear-encoded copy was detected (both chromatophore and nuclear-encoded copies were identified in *P. micropora* KR01). Similarly, the pyrimidine biosynthesis pathway in *P. chromatophora* CCAC0185 mirrors almost exactly what was observed in *P. micropora* KR01 (Table 2). There were only three enzymes not detected in the nuclear genome (EC 1.17.4.2, EC 3.5.4.13, and EC 2.4.2.2/2.4.2.3/2.4.2.4) and one enzyme not detected in the chromatophore genome (EC 2.7.4.9) of *P. chromatophora* CCAC0185 that we expected based on the results from *P. micropora* KR01 (Table 2). Finally, like in *P. micropora* KR01, the DNA replication genes that encode the DNA ligase, DNA polymerase, and RNase HI/HII proteins were not detected in the chromatophore genome. Only DNA ligase had a gene copy that encoded a crTP-containing protein, however DNA polymerase I and ribonuclase HI/HII have gene copies that encoded 5′-truncated proteins (Table 3), making it possible that crTP-containing copies of these genes are present in the nuclear genome of *P. chromatophora* CCAC0185.

## Discussion

### Nucleotide biosynthesis in the chromatophore is likely controlled by the host

Key enzymatic reactions that are part of the initial (i.e., generation of IMP and UMP) and final (i.e., generation of guanosine, adenine, and thymine) steps of *de novo* nucleotide biosynthesis are assigned to genes only present in the nuclear genome. We hypothesize that this allows the host to control generation of the precursor molecules required for this pathway to function in the endosymbiont. Moreover, the genes involved in nucleotide degradation and salvage are all (except for the two gene associated with EC 2.4.2.9 and 3.5.4.13; Figure 2B) nuclear encoded; 4/11 purine degradation and salvage reaction associated proteins are chromatophore targeted, whereas 4/10 of the genes encoding the pyrimidine salvage and degradation enzyme reactions are chromatophore targeted. These results demonstrate that nucleotide synthesis in the cell is gradually becoming chromatophore localized, but is under host control, whereas nucleotide metabolism occurs in the cytosol. In plant cells, nucleotide synthesis is compartmentalized: *de novo* synthesis of precursor molecules (IMP and UMP) is mainly localized to the plastid and mitochondria, whereas catabolism and salvage reactions take place in the cytosol (Witte and Herde, 2020; Zrenner et al., 2006). Although the localization of these reactions in *Paulinella* is not as clear-cut as it is in plants, nucleotide biosynthesis seems to follow a similar trend, suggesting that localization of certain reactions might be energetically, physiologically, or regulatorily favored in specific compartments.

The enzyme required for the synthesis of cytosine from cytidine (ribonucleoside hydrolase, EC 3.2.2.8), was not detected in either genome. However, it is unclear if the absence of this enzyme in photosynthetic *Paulinella* is significant, given that this enzyme has only been characterized in *Escherichia coli* (Petersen and Moller, 2001) and has been identified in only a small number of non-photosynthetic prokaryotes. Only the enzyme that catalyzes the conversion of cytosine to uracil was identified in *P. micropora* KR01, and it was assigned to a single chromatophore-encoded gene. Some key enzyme reactions, such as EC 1.17.4.1 which represents the conversion of UDP to dUDP (Figure 2A), are only assigned to nuclear genes that do not encode a crTP. There are also some key reactions, such as EC 2.7.4.22 which is the bidirectional conversion of UMP to/from UDP, that are only assigned to chromatophore genes. These instances often stand in contrast to the adjoining enzymatic reactions in the pathways which are either annotated to genes in both genomes, or to genes in the nuclear genome that encode a crTP. This might suggest that these steps are only occurring in one compartment of the cell, demonstrating a progression toward localization of redundant biosynthetic reactions to specific cellular compartments. Alternative explanations are that some genes have been missed during prediction, that nuclear-encoded genes encoding a crTP exist but are absent from the available genome data, or that the nuclear-encoded proteins use a highly diverged targeting sequence. The cellular localization of these reactions remains to be further explored, with additional genomic and biophysical analyses required to confirm the absence of putative missing genes and to confirm the localization of the proteins and enzymatic reactions.

### The host may control the chromatophore using the stringent response pathway

The “stringent response” is a stress signaling pathway in bacteria and plant chloroplasts that is activated in response to nutrient starvation. This pathway targets a broad range of cellular processes, including DNA replication, transcription, and translation, ribosome biogenesis and function, lipid metabolism, and nucleotide synthesis, to limit nutrient use during periods of starvation (Irving et al., 2021). The major signaling molecules involved in this pathway are ppGpp and pppGpp (collectively known as (p)ppGpp) which are synthesized from GDP and GTP (respectively) by two enzyme reactions (EC 3.1.7.2 and 2.7.6.5), which in *P. micropora* KR01 are annotated to a single gene in the chromatophore genome (APP88130.1; Table 1). The enzyme reaction (EC 3.6.1.11) that catalyzes the bidirectional conversion of ppGpp to/from pppGpp is annotated to a single chromatophore-targeted nuclear-encoded protein (MSTRG.13432.1.p1; Table 1). In heterotrophic bacteria (such as *E. coli*) (p)ppGpp inhibits the function of DnaG (DNA primase), preventing DNA replication. It also inhibits some of the proteins involved in purine metabolism (Irving et al., 2021), specifically those that convert PRPP to PRA (5-phosphoribosylamine; EC 2.4.2.14), IMP to XMP (EC 1.1.1.205), GMP to GDP (EC 2.7.4.8), and IMP to SAMP (EC 6.3.4.4). In all cases except for EC 2.4.2.14, which is assigned only to nuclear-encoded proteins that are not chromatophore targeted, these enzyme reactions in *P. micropora* KR01 occur through genes encoded in both the nuclear and chromatophore genomes. (p)ppGpp can also inhibit or enhance the activity of proteins involved in purine degradation however, because all genes encoding these proteins in *P. micropora* KR01 are nuclear-encoded, it is unclear if they would be regulated by the stringent response like those that are chromatophore-encoded. The stringent response in photosynthetic cyanobacteria is less well characterized then in heterotrophic bacteria, however, ppGpp in *Synechococcus elongatus* (a photosynthetic cyanobacteria) is functional in the light/dark cycle and globally downregulates gene expression in the dark when the primary energy source of the organism is not available (Hood et al., 2016).

We hypothesize that *Paulinella* might utilize the stringent response to exert control over the replication and function of the chromatophore through expression of a chromatophore targeted protein that catalyzes the EC 3.6.1.11 enzyme reaction. The host, by controlling the conversion of pppGpp to ppGpp can potentially activate the stringent response in the chromatophore (which appears to be predominantly controlled by ppGpp), halting replication and downregulating major functions such as amino acid biosynthesis and potentially, photosynthesis. The host may also alleviate the strongest response because the same enzyme catalyzes the conversion of ppGpp to pppGpp. The chromatophore, by retaining a gene that catalyzes the EC 3.1.7.2 reaction (which can synthesize or degrade ppGpp) is able to activate the stringent response pathway in response to stress or light deprivation (as in free-living cyanobacteria) and can also alleviate the stringent response regardless of whether it was activated by the host or itself, by conversion of ppGpp to GDP. In addition, the same protein can catalyze the EC 2.7.6.5 reaction, giving the chromatophore control over the synthesis of pppGpp (from GTP), which might also allow it to regulate the host’s control over the stringent response. The nuclear-encoded EC 3.6.1.11 enzyme gene shows a weak diurnal expression pattern (Figure S1) and relatively strongly upregulated under high-light stress*.* Given that photosynthetic *Paulinella* have a doubling time of 5–7 days, the weak diurnal pattern that we observe might be a result of the cell cycle of the culture not being synchronized, with only a fraction of the cells dividing during each 24h period. In addition, because this enzyme is bidirectional it is not possible to identify if its upregulation would result in the synthesis or degradation of ppGpp. However, the light-dependent response of this gene suggests that it could play a role in host control of chromatophore activity. Additional research is needed into the degree of control that the stringent response has over gene expression in the chromatophore, and if the expression of the host-encoded protein that can putatively catalyze the EC 3.6.1.11 reaction has a noticeable effect on the accumulation of (p)ppGpp in the chromatophore.

### Evolution of chromatophore targeted proteins

Interestingly, of the eight KEGG orthogroups from the purine and pyrimidine metabolism pathways that are assigned to multiple nuclear-encoded genes (Tables 1 and 2), and where one of the encoded proteins contains a crTP, five have all their genes grouped together in the same clade in phylogenetic trees (Data S1). Of interest, there tends to be only two genes annotated to each of these KEGG orthogroups. This suggests that these genes underwent recent duplication events, possibly after endosymbiosis, before one of the gene copies could acquire a crTP encoding sequence for chromatophore targeting. In contrast, the DNA ligase and DNA polymerase I genes are positioned in different parts of their respective trees, suggesting that the gene copies are derived from ancient duplication events. These results suggest that evolution favors the modification of existing gene copies (that is, gene copies that evolved before the extant selective pressure) rather than modification of gene copies derived from duplication of an extant gene.

### The chimeric chromatophore DNA replication pathway in *Paulinella*

The bacterial DNA replication pathway present in the *P. micropora* KR01 chromatophore comprises proteins derived from both the host and endosymbiont genomes (Figure 3B). Four of the protein listed in the KEGG bacterial DNA replication pathway that were not identified in either of the *P. micropora* KR01 genomes are also not present in *Synechococcus* sp. WH5701, suggesting that they were absent from the ancestor of the chromatophore and that all genes required for DNA replication in the chromatophore are present in *P. micropora* KR01. The four exclusively nuclear-encoded proteins all have a single annotated gene that encodes a crTP, demonstrating that the host has compensated for the loss of these genes from the chromatophore by targeting nuclear-encoded proteins to the nascent organelle. The one protein, DNA polymerase III subunit epsilon, present in both nuclear and chromatophore genomes, also has a nuclear-encoded gene encoding a crTP. The presence of this gene in both genomes (and with a crTP on one of the nuclear encoded proteins) likely results from the ongoing evolution of *Paulinella* to accommodate and control the chromatophore. The nuclear-encoded proteins with a crTP appear to have long, non-canonical, serine-rich regions (Figure S2) that might impair or abolish gene function, or they might have a function related to cleavage of the crTP (Oberleitner et al., 2022). Inactivation of this gene would explain why a copy is still maintained in the chromatophore genome. It is possible that this gene recently acquired a crTP encoding region but did not provide a strong selective advantage to the cell (e.g., because of low targeting efficiency, inefficient expression control, or because the serine repeats affected protein function). It is also possible that the nuclear-encoded gene is still active and that the chromatophore-encoded copy is no longer under selection and will eventually be purged from the organelle. However, because the chromatophore-encoded gene does not show signs of degradation, the first theory, that the nuclear-encoded copy failed to provide a strong selective advantage to the cell, likely explains why this protein is encoded in both genomes.

Only two of the genes annotated as nuclear-encoded bacterial DNA replication pathway proteins show evidence of having been derived from bacterial HGTs, and surprisingly, none show evidence of being derived from EGT. Of the five DNA polymerase I genes, the one which encodes a crTP is putatively of bacterial origin (Data S4). This gene is positioned in a well-supported clade (BS ≥ 95%) of mostly bacteria; however, there are several other eukaryotes in this clade that make the origin of this protein difficult to identify with confidence. In plants and algae, the DNA polymerase I enzyme is involved in plastid and mtDNA replication and is known as POP (plant organellar DNA polymerase (Moriyama et al., 2014)). The presence of the crTP on this putative bacterial-derived protein suggests that although DNA polymerase I proteins were already present in *P. micropora* KR01 (likely functioning as part of the mtDNA replication pathway [POP enzyme]), none of them were compatible with the chromatophore pathway. Thus, a bacterial gene copy had to be acquired and its product targeted to the chromatophore.

Of the two DNA polymerase III subunit epsilon genes in *P. micropora* KR01, one (MSTRG.8071.1.p1; mitochondrial-targeted) is of putative bacterial origin (Data S5), whereas the other (chromatophore-targeted) is potentially of bacterial origin but is likely non-functional because of the presence of non-canonical serine repeats in the protein that disrupt the conserved functional region (Figure S2). The limited similarity of these two genes to sequences from eukaryotes, combined with the fact that they are annotated as proteins that function exclusively as part of bacterial DNA replication, suggest that they originated in *P. micropora* KR01 via HGT from bacteria. Whereas our phylogenetic analysis did not show a common origin for these proteins in *P. micropora* KR01, if one of the genes is non-functional (as we suggest based on its sequence) then its placement in the tree should be interpreted cautiously. Furthermore, if these two proteins have a common origin, then the presence of a mtTP in one of the proteins suggests that it could have been acquired before endosymbiosis, potentially functioning in mtDNA replication. This provides a mechanism through which selection would have driven the retention and integration of the foreign gene into the host genome before endosymbiosis has occurred. This scenario would overcome the problem of why weak selective pressure acting on the “pre-adaptive” genes would be maintained by selection, before endosymbiosis (Ku et al., 2015). However, if these proteins have different origins, then this theory would not be supported. The lack of putative EGT events suggests that existing bacterial genes in *Paulinella* were a prerequisite for endosymbiosis and might have allowed the host to rapidly gain control over the chromatophore.

### Some DNA replication proteins are constrained to the chromatophore genome

Interestingly, DNA polymerase III subunits, DNA helicase, DNA primase, and single-stranded DNA binding (SSB) proteins, which interact during DNA replication (Antony and Lohman, 2019; Shereda et al., 2008), are all (assuming that the nuclear-encoded DNA polymerase III subunit epsilon is non-functional) exclusively chromatophore-encoded (Figure 3A). During DNA replication, SSB proteins in *E. coli* (and in other bacteria) interact with DNA primase (DnaG) proteins and the χ subunit of DNA polymerase III (Shereda et al., 2008); although the latter was not detected in *P. micropora* KR01 or *Synechococcus* sp. WH5701, it is possible that the SSB still interacts with the DNA polymerase III complex via another subunit or that the subunit has been missed during gene prediction. DNA primase and DNA helicase (DnaB) also interact during DNA replication (Shereda et al., 2008). It has been proposed that the assembly and subsequent function of a protein complex is affected by the stoichiometric balance of its members (Birchler and Veitia, 2012), which can be affected by the timing of gene expression (Wang et al., 2019). Regulation of expression of nuclear-encoded genes by signals from the chromatophore and the import of the translated proteins into the chromatophore are likely to be inefficient and may have resulted in a significant lag between the initial signal and the resulting change in protein concentration in the organelle. Therefore, the need to maintain stoichiometric balance of the DNA polymerase III subunits, DnaB, DnaG, and SSB proteins (which all interact) may prevent the transfer of a subset of these proteins to the nuclear genome, because it would likely lead to inefficient regulation and expression of these proteins that would affect the assembly and function of the functional complex. This situation is analogous to the “co-localization for redox regulation” (CORR) hypothesis (Allen, 1993) which describes the need for genes to be maintained in organelle genomes to allow their expression to be controlled by the redox state of their products. In the case of *Paulinella*, chromatophore genome localization of genes is likely driven by the need to maintain a specific stoichiometric balance of the products rather than their redox state. Furthermore, what we observe in *Paulinella* may result from the fundamental underlying process that shaped the evidence for the CORR hypothesis, but because the endosymbiosis in *Paulinella* is at an intermediate stage, we are seeing its effects on a broad range of functions, not just the ones that are redox regulated. This could explain why the epsilon subunit has not been lost from the chromatophore genome despite the existence of a (albeit, potentially non-functional) nuclear-encoded chromatophore targeted gene product. It could also explain why the DNA ligase, DNA polymerase I and RNase HI and HII proteins are now exclusively nuclear encoded. That is, they do not form strong protein-protein interactions with other parts of the DNA replication process, however, RNase HI has been shown to interact with SSB in *E. coli* (Antony and Lohman, 2019). The transfer of the remaining DNA replication genes from the chromatophore might have to progress in an “all or nothing” scenario, whereby all the remaining genes are transferred to the nuclear genome and acquire expression regulation and chromatophore targeting to maintain cell viability.

It should be noted that a comprehensive analysis of all multi-gene protein complexes that are completely or partially encoded in the chromatophore genome is required before the “all or nothing” scenario can be proven. A counter-example of this scenario in *Paulinella* is the nuclear-encoded PsaE and PsaK genes, which are subunits of the highly coordinated photosystem I (PSI) complex (Nowack and Grossman, 2012). However, targeted inactivation of PsaE (Jeanjean et al., 2008) and PsaK (Naithani et al., 2000) in the model cyanobacterium *Synechocystis* sp. PCC 6803 showed minimal negative effects on the function of PSI. If this is also true for PS1 in *Paulinella*, then this may support the “all or nothing” scenario, that is, if the PsaE and PsaK genes are not essential for the correct function of 10.13039/100015727PSI then the “all or nothing” scenario actually predicts that they would be the first to be relocated to the nuclear genome. Whereas additional research is needed, *Paulinella* represents an excellent model for studying the forces that govern the transfer of multi-protein complex-forming genes between the endosymbiont and its host.

There are significant differences in the expression patterns of the DNA replication proteins assigned to nuclear-encoded proteins containing a crTP (i.e., two of the genes encoding a crTP follow a diurnal cycle, whereas the other three do not). Assuming that these genes will evolve a diurnal expression profile, these results suggest that evolution may drive the acquisition of a crTP before it drives the refinement of the expression pattern of the gene. This discordance in the expression profile of these functionally linked genes may also act as an example of why more tightly associated proteins (e.g., the DNA polymerase III subunits) are still encoded in the chromatophore genome.

### Host control of chromatophore cell division as an early step in the endosymbiosis of *Paulinella*

The location of enzymes involved in the DNA replication and nucleotide biosynthesis pathways in *P. chromatophora* CCAC0185 strongly mirror what is observed in *P. micropora* KR01. All genes that were not detected in the chromatophore genome of *P. micropora* KR01 were also absent from the chromatophore genome of *P. chromatophora* CCAC0185*.* Moreover, many genes that were nuclear-encoded with a crTP in *P. micropora* KR01 also had the same configuration in *P. chromatophora* CCAC0185, with the exceptions (EC 2.4.2.1, EC 2.4.2.2/2.4.2.3/2.4.2.4, EC 2.7.1.48, DNA polymerase I, and RNase HI/HII) possibly explained by missing or fragmented gene models in the nuclear proteome of *P. chromatophora* CCAC0185. For example, many of the gene copies assigned to the DNA polymerase I and RNase HI/HII enzymes had truncated 5′-termini, making it possible that crTP-encoding copies of these genes will be recovered once a more complete proteome is available. Furthermore, the fact that some nuclear-encoded proteins were not detected in our analysis (e.g., EC 2.4.2.2/2.4.2.3/2.4.2.4) may be explained by an incomplete predicted nuclear proteome in *P. chromatophora* CCAC0185, and not gene loss*.* These data were derived from assembled transcripts because the nuclear genome of this species is ∼9.6 Gbp in size (Nowack et al., 2016) and the initial assembly is highly fragmented and partial. Therefore, it is not yet possible to conclude that these genes are truly missing from the nuclear genome or which enzymes are not targeted to the chromatophore. In contrast to the nuclear genome, the chromatophore proteome of *P. chromatophora* CCAC0185 is highly complete because of the high-quality chromatophore genome assembly that is available (Nowack et al., 2008)*.* Therefore, enzymes detected in the chromatophore genome of *P. micropora* KR01 but not detected in the *P. chromatophora* CCAC0185 chromatophore genome (i.e., EC 6.3.5.3 and EC 2.7.4.9) might represent genes that were lost after these two species diverged ca. 60 Ma. The distribution of the genes that encode the enzymes involved in the nucleotide biosynthesis and DNA replication pathways demonstrates that the transition to these genes being exclusively nuclear-encoded likely occurred before the split of these two species ca. 60 Ma, making this process one of the key early steps in the establishment of endosymbiosis.

### Control of endosymbiont pathways through gene loss

**Figure 4:**
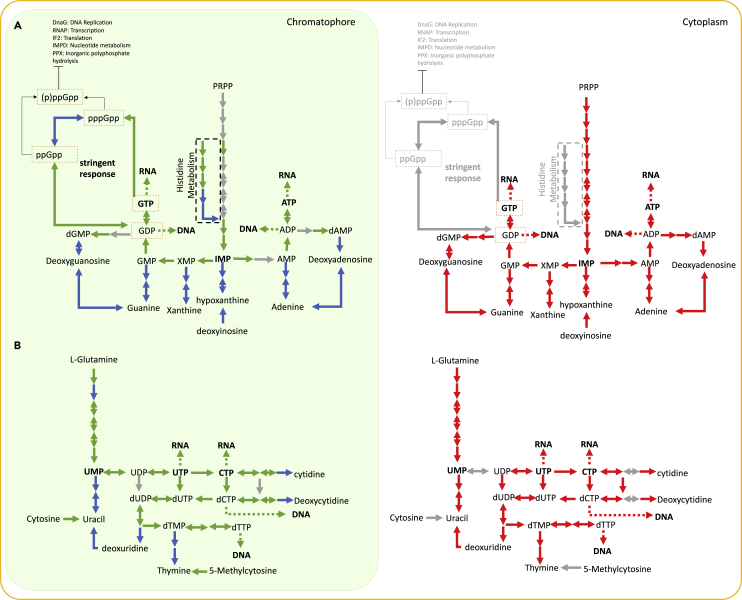
Summary of enzyme localization of nucleotide *de novo* biosynthesis pathways in photosynthetic *Paulinella* (A) Purine and (B) Pyrimidine *de novo* biosynthesis pathway enzymes that are localized to the chromatophore (green box) and the cytosol (orange outline) in photosynthetic *Paulinella*. Red arrows represent enzymes encoded by the nuclear genome and localized to the cytosol, green arrows represent enzymes encoded by the chromatophore genome and localized to the chromatophore, and blue arrows represent enzymes that are nuclear-encoded and chromatophore localized (i.e., transported into the chromatophore by a crTP). Gray arrows represent enzymes that are not predicted to be localized to that compartment.

During the initial stages of endosymbiosis, the host needs to rapidly gain control over endosymbiont biology. Full integration and control of these complex functions, particularly those that are performed in and across both compartments, is likely to occur over longer evolutionary time-scales: i.e., not during the early stages of endosymbiosis. *Paulinella* appears to have overcome the hurdle of endosymbiont integration by encoding key genes involved in complex functions that occur (and are encoded) in both compartments (such as nucleotide biosynthesis) exclusively in the nuclear genome (Figure 4). This control is gained through loss of key genes from the chromatophore genome (either through outright gene loss or transfer to the nuclear genome) that occupy central parts of target functions or pathways, leaving only the nuclear-encoded versions that give control of these functions in the chromatophore to the host. This process was observed to have occurred with the chromatophore-encoded nucleotide precursor, nucleotide synthesis, and stringent response pathways, as well as for the chromatophore DNA replication complex. This process has likely given the host amoeba control of these pathways in both compartments through a small number of evolutionary steps, thereby cementing the endosymbiotic relationship. This theory complements the ‘chassis and engine’ model (Stephens et al., 2021), which describes the challenges associated with integration and control of novel, highly efficient, and endosymbiont-specific functions into host metabolism.

### Retrograde signaling likely does not play a role in *Paulinella* nuclear gene regulation

In eukaryotic cells, tight coordination between the host (nucleus) and the endosymbionts (organelles) is essential for survival of the organism. This is particularly true for photosynthetic organisms in which proteins are encoded by many separate genomes in different cellular compartments (i.e., nucleus, plastid, and mitochondrion). During the Archaeplastida plastid endosymbiosis, the organelle genome underwent extensive reduction, resulting in the loss (outright or from transfer to the nuclear genome) of many genes and functions; the plastid therefore relied on proteins from the cytosol to compensate for the functions of these genes (Jarvis and Lopez-Juez, 2013). The encoding (and there for regulatory control) of proteins that function in an organelle in the nuclear genome necessitates the evolution of a system for transmission of information between the different compartments and genomes. This system is known as retrograde signaling (Nott et al., 2006) and allows for the metabolic state of the organelle to directly affect the expression of nuclear-encoded genes. A system that functions in the opposite direction, anterograde signaling, allows for the regulation of organelle gene expression in response to the metabolic state or stimuli perceived by the nucleus (Woodson and Chory, 2008). In plants, retrograde signaling regulates the expression of nuclear genes that encode chloroplast-localized proteins (Biehl et al., 2005; Richly et al., 2003). This regulation was observed in response to chloroplast biogenesis (Terry and Smith, 2013), high light (Estavillo et al., 2011), and redox stress (Pfalz et al., 2012). Clearly, refined communication between a host and its organelle contributes to the success of primary endosymbiosis and integration of the symbiont into host metabolism. In photosynthetic *Paulinella,* whereas chromatophore gene regulation has not been explored in depth, one study looked at the impact of light on *P. chromatophora* CCAC0185 gene expression and found that light-induced transcriptional regulation is lacking in chromatophore-encoded and most EGT-derived nuclear genes, including the EGT-derived nuclear-encoded PSII and PSI genes (Zhang et al., 2017). This suggests that in photosynthetic *Paulinella,* chromatophore-encoded genes and EGT-derived genes are not light-regulated. Given that retrograde signaling in other photoautotrophs is involved in light-regulation of nuclear-encoded genes with plastid functions (Leister, 2012), it would appear that retrograde signaling does not occur in *Paulinella*, or that it occurs under stimuli that have not yet been tested, that it has a weak effect on transcription, or that it only regulates the expression of a limited set of genes. All this suggests that the nuclear-encoded nucleotide biosynthesis and DNA replication pathway genes are likely to be host regulated under the conditions used in our study, and not be under chromatophore control via retrograde signaling. Our research was, however, not designed to address retrograde signaling, therefore, additional work is required to fully assess the potential role of this mechanism on the regulation of nuclear-encoded genes in the *Paulinella* lineage.

### Limitations of the study

The results of this study are based on analysis of the available genome assemblies and only explored if key genes were present or absent in either genome. Additional analysis, using RNA-seq and metabolomic data, is needed to explore if changes in the expression of the key nuclear-encoded genes correspond with a predictable shift in the metabolite pools in the chromatophore. Subcellular localization of these proteins is also needed to show the extent of compartmentalization of these functions in *Paulinella*.

## STAR★Methods

### Key resources table

**Table:** 

REAGENT or RESOURCE	SOURCE	IDENTIFIER
**Deposited data**		

Manually corrected gene models and phylogenetic trees	This paper	https://doi.org/10.5281/zenodo.6418901
Original code associated with this research	https://github.com/TimothyStephens/Paulinella_micropore_KR01_pathways_analysis	Version 1
*Paulinella micropora* KR01 genome data	http://cyanophora.rutgers.edu/P_micropora/; Lhee et al. (2021)	Version 1
*Paulinella micropora* KR01 RNA-Seq reads	https://www.ncbi.nlm.nih.gov/bioproject/PRJNA568118; Lhee et al. (2021)	BioProject PRJNA568118
NCBI RefSeq v.95 protein database	https://www.ncbi.nlm.nih.gov/refseq/	Version 95
Pfam	http://pfam.xfam.org/	Release 33.1
UniProt	https://www.uniprot.org/	Releases 2019_10 & 2020_05

**Experimental models: Organisms/strains**		

*Paulinella micropora* KR01	http://cyanophora.rutgers.edu/P_micropora/	KR01

**Software and algorithms**		

BLAST	Camacho et al. (2009)	https://ftp.ncbi.nlm.nih.gov/blast/executables/LATEST/; RRID:SCR_004870
KAAS	Moriya et al. (2007)	http://www.genome.jp/kegg/kaas/
KEGG pathway mapper	Kanehisa and Sato (2020)	https://www.genome.jp/kegg/mapper/reconstruct.html; RRID:SCR_018145
Trimmomatic v0.38	Lea et al. (2015)	http://www.usadellab.org/cms/?page=trimmomatic; RRID:SCR_011848
HISAT2 v2.1.0	Kim et al. (2019)	http://daehwankimlab.github.io/hisat2/; RRID:SCR_015530
samtools v1.8	Li et al. (2009)	http://www.htslib.org/; RRID:SCR_002105
StringTie2 v2.0.6	Kovaka et al. (2019)	https://ccb.jhu.edu/software/stringtie/; RRID:SCR_016323
IGV v2.8.12	Robinson et al. (2011)	https://software.broadinstitute.org/software/igv/; RRID:SCR_011793
Minimap2 v2.17	Li (2018)	https://github.com/lh3/minimap2; RRID:SCR_018550
Trans-Decoder v5.5.0	N/A	https://github.com/TransDecoder/TransDecoder; RRID:SCR_017647
HMMER v3.1b2	Eddy (2011)	http://hmmer.org/; RRID:SCR_005305
Salmon v1.1.0	Patro et al. (2017)	https://salmon.readthedocs.io/en/latest/index.html; RRID:SCR_017036
MAFFT v7.453	Katoh and Standley (2013)	https://mafft.cbrc.jp/alignment/software/; RRID:SCR_011811
IQTREE v1.6.12	Nguyen et al. (2015)	http://www.iqtree.org/
TreeViewer v1.2.2	N/A	https://github.com/arklumpus/TreeViewer
AliStat v1.12	Wong et al. (2020)	https://github.com/thomaskf/AliStat
TargetP-2.0	Almagro Armenteros et al. (2019)	https://services.healthtech.dtu.dk/service.php?TargetP-2.0; RRID:SCR_019022
Trinity v2.9.0	Grabherr et al. (2011); Haas et al. (2013)	https://github.com/trinityrnaseq/trinityrnaseq; RRID:SCR_013048
Jellyfish v2.3.0	Marcais and Kingsford (2011)	https://genome.umd.edu/jellyfish.html; RRID:SCR_005491
Bowtie2 v2.3.5.1	Langmead and Salzberg (2012)	http://bowtie-bio.sourceforge.net/bowtie2/index.shtml; RRID:SCR_016368

### Resource availability

#### Lead contact

Further information and requests for resources and reagents should be directed to and will be fulfilled by the lead contact, Timothy Stephens (ts942@sebs.rutgers.edu).

#### Materials availability

This study did not generate new unique reagents.

### Method details

#### Annotation of *Paulinella* genes to KEGG pathways

A BLASTp (Camacho et al., 2009) query against an in-house database composed of NCBI RefSeq v.95 proteins was used to functionally annotate proteins predicted in the *P. micropora* KR01 nuclear and chromatophore genomes (Lhee et al., 2017, 2021). KEGG Orthology (KO) numbers were assigned to the nuclear and chromatophore derived proteins from *P. micropora* KR01 (Lhee et al., 2017, 2021) and *P. chromatophora* CCAC0185 [https://www.ebi.ac.uk/pride/archive/: PXD006531] (Nowack et al., 2008, 2016) using KAAS (KEGG Automatic Annotation Server: http://www.genome.jp/kegg/kaas/) (Moriya et al., 2007). The resulting KO file, containing all predicted proteins with assigned K numbers, was used to generate metabolic maps using the KEGG pathway mapper (Kanehisa and Sato, 2020). KEGG maps were used to identify genes related to DNA replication (KO-03030), DNA repair (KO-03430/KO-03440) and nucleotide biosynthesis (KO-00230/KO-00240). All genes related to these pathways were then extracted, manually validated, and used for downstream analyses.

#### Correction of *P. micropora* KR01 genes using RNA-seq data

*P. micropora* KR01 proteins annotated as being part of the KEGG pathways of interest had their underlying gene models checked for inconsistencies using aligned RNA-seq reads. The assembled *P. micropora* KR01 nuclear genome was retrieved from http://cyanophora.rutgers.edu/P_micropora/ and RNA-seq reads from *P. micropora* KR01 (BioProject PRJNA568118) were retrieved from NCBI Sequencing Read Archive (Lhee et al., 2021). RNA-seq reads were trimmed using Trimmomatic (v0.38; ‘ILLUMINACLIP:adapters.fa:2:30:10 SLIDINGWINDOW:4:5 LEADING:5 TRAILING:5 MINLEN:25’) (Lea et al., 2015) and read pairs where both mates survived trimming were aligned against the reference genome using HISAT2 (v2.1.0; ‘-q --phred33 --no-unal --dta --rf’) (Kim et al., 2019). Aligned reads were sorted using ‘samtools sort’ (v1.8) (Li et al., 2009) and RNA-seq-based gene models were constructed for each library using StringTie2 (v2.0.6; ‘--rf’) (Kovaka et al., 2019) before being merged into a combined set (‘stringtie2 --merge’).

The program IGV (v2.8.12) (Robinson et al., 2011) was used to visualize the *P. micropora* KR01 gene models, the RNA-seq-based gene models constructed by StringTie2, PacBio long reads (SRR10230249) aligned using Minimap2 (v2.17;--secondary=no -ax map-pb (Li, 2018)), and the aligned RNA-seq reads for manual inspection. For each *P. micropora* KR01 gene in the target KEGG pathways, the best StringTie2 gene model, which most closely matched the intron-exon structure of the *P. micropora* KR01 gene, was selected and used for downstream analysis. If multiple best gene models were available, then the one with the highest expression (inferred by StringTie2) was taken. The terminal exons of StringTie2 based gene models were adjusted or removed if they had expression that was significantly lower than the rest of the gene or if they had very few reads anchoring them to the adjoining exons. Open Reading Frames (ORFs) were predicted in each of the selected StringTie2 gene models by Trans-Decoder (v5.5.0; https://github.com/TransDecoder/TransDecoder; ‘Trans-Decoder.LongOrfs -S -m 30’; ‘Trans-Decoder.Predict --single_best_only’); HMMER v3.1b2 (Eddy, 2011) was used to search the candidate ORFs against the Pfam database (release 33.1) and BLASTp (v2.10.1+; ‘-max_target_seqs 1 -outfmt 6 -evalue 1e-5’) was used to search the candidate ORFs against the SwissProt database (release 2020_05), with the resulting homology information used by Trans-Decoder to guide ORF prediction. If a predicted ORF was missing a start codon (i.e., the predicted ORF extended to the 5′-end of the gene model) then the first downstream start codon that was <30 aa or <15% from the current 5′-termini was used (if one was available; approach based on Trans-Decoder’s start codon refinement procedure). Information about the initial and corrected *P. micropora* KR01 genes are shown in Table S1. This strategy, which reconstructed the gene models using aligned RNA-seq data, served as a means of verifying the gene model structure, removing or correcting mispredicted exons, and correcting the start codons of 5′-incomplete ORFs.

#### Expression quantification of manually corrected genes

The initial set of genes that were identified (using KAAS) as being part of the bacterial DNA replication complex, purine metabolism, or pyrimidine metabolism pathways were removed from the set of all genes predicted in *P. micropora* KR01. The remaining *P. micropora* KR01 gene CDS sequences were combined with the transcripts of the manually corrected gene modes to create a sequence database that was used for quantification analysis. Salmon v1.1.0 (‘index --index puff --kmerLen 31’; ‘quant --validateMappings --seqBias --gcBias --libType ISR’; (Patro et al., 2017)) was used with the trimmed *P. micropora* KR01 RNA-seq libraires (BioProject PRJNA568118; used for manual correction of the targeted gene models) to quantify the expression (in Transcripts Per Million [TPM]) of the manually corrected genes.

#### Comparison of *P. micropora* KR01 genes against orthologs of KEGG pathways in UniProt

Protein sequences from UniProt (SwissProt + TrEMBL; release 2019_10), annotated with one of the KEGG orthologs from the KEGG pathways being examined, were retrieved (using information available from https://www.kegg.jp; accessed 27-Jan-2021). The corrected *P. micropora* KR01 nuclear genes were compared (BLASTp v2.10.1+; default settings) against the UniProt sequences from the KEGG Ortholog that they (i.e., the initial KR01 gene) were originally annotated with. The top scoring UniProt hit (*e*-value < 1e-5) for each *P. micropora* KR01 gene was retrieved and used to assess if the corrected *P. micropora* KR01 genes still had homology to the KEGG Orthologs to which they were originally annotated; corrected genes without any homology to UniProt KEGG Ortholog sequences (above the *e*-value < 1e-5 threshold) were excluded from downstream analysis. Results are shown in Tables S1 and S2.

#### Phylogenetic analysis of corrected *P. micropora* KR01 genes

Sequences from NCBI RefSeq, plus available algal and protist genome and transcriptome data from dbEST, TBestDB, the JGI Genome Portal (https://genome.jgi.doe.gov) and the Moore Microbial Eukaryote Transcriptome Sequencing Project (Keeling et al., 2014) were retrieved and partitioned into four sets based on taxonomic origin: (1) Sequences from bacteria, (2) sequences from Opisthokonta, (3) the remaining sequences not from bacteria or Opisthokonta, and (4) sequences from the Moore Microbial Eukaryote Transcriptome Sequencing Project database. The corrected *P. micropora* KR01 proteins were searched independently (BLASTp v1.10.1; ‘-max_target_seqs 2000 -evalue 1000’) against each of the four (i-iv) database subsets. For each query, the top hits against each set were filtered (*e*-value ≤1e-10), combined, and sorted by bitscore (descending order). From the sorted list of hits a taxonomically broad selection of top hits was extracted. The selected top hits from each corrected *P. micropora* KR01 nuclear-encoded protein annotated with the same KEGG Ortholog were combined with proteins annotated with the same KO number from the other available *Paulinella* species, had duplicate top hit sequences removed, and associated proteins aligned together with the corrected *P. micropora* KR01 protein sequences using MAFFT (v7.453; ‘--localpair --maxiterate 1000’) (Katoh and Standley, 2013). IQTREE (v1.6.12; ‘-m LG+R7 -bb 2000 -quiet’) (Nguyen et al., 2015) was used to construct maximum-likelihood phylogenetic trees, with automatic model selection and node support tested via 2,000 ultrafast phylogenetic bootstraps (Minh et al., 2013). Trees and alignments were visualized together using TreeViewer (v1.2.2; https://github.com/arklumpus/TreeViewer); the completeness score for each sequence in the alignment (Cr values) was computed by AliStat (v1.12) (Wong et al., 2020) and visualized alongside the trees shown in Data S1.

#### Prediction of organelle transit peptides

Chromatophore transit peptides (crTP) were predicted in the manually corrected *P. micropora* KR01 proteins using an HMM constructed from *P. micropora* KR01 crTP peptide sequences identified by Lhee et al. (2021). Briefly, HMMER v3.1b2 was used to build the *P. micropora* KR01 crTP HMM from a manually curated alignment (from Lhee et al. (2021)) of the identified *P. micropora* KR01 crTP sequences; crTP hits retuned using HMMER were retained if they had a c-Evalue < 1x10^−5^ (results shown in Table S2). The previously generated chromatophore-targeting peptide (crTP) proteins database (Nowack et al., 2016) was used to identify proteins with crTP in *P. chromatophora* CCAC0185. TargetP-2.0 was used to predict mitochondrial transit peptides (mtTP) in the manually corrected *P. micropora* KR01 proteins (Almagro Armenteros et al., 2019). The “organism” parameter of TargetP-2.0 was set to “non-plant” (as opposed to the other possible option of “plant”) as *P. micropora* KR01 has recently evolved from a heterotrophic lineage and the chromatophore has a separate origin to the canonical plastid in all other photosynthetic eukaryotes, that is, *Paulinella* does not fall under the definition of “plant” used by TargetP-2.0.

#### Prediction of KEGG Ortholog numbers in *Synechococcus* and other available *Paulinella* species

KEGG ortholog numbers were predicted in the *P. chromatophora* nuclear and chromatophore-derived proteins, the *Paulinella ovalis* (heterotrophic sister species) predicted proteins (Bhattacharya et al., 2012), and the *Synechococcus* sp. WH5701 predicted proteins using kofamscan (v1.1.0; KOfam database retrieved November 2020) (Aramaki et al., 2020). Selected *P. ovalis* predicted proteins and their associated genome scaffolds were compared against the RefSeq nr and nt databases in order to assess their taxonomic provenance.

#### Conformation of key genes not detected in *P. micropora* KR01

The absence of proteins in the *P. micropora* KR01 nuclear and chromatophore genomes annotated as enzymes involved in cytosine metabolism (EC 3.5.4.1 [K01485 and K03365], EC 2.4.2.2 [K00756 and K09913], EC 3.2.2.8 [K10213], and EC 3.2.2.10 [K06966]) and conversion of SAMP to AMP (EC 4.3.2.2 [K01756]) were confirmed through additional analysis. To assess if these enzymes are missing from the chromatophore genome protein sequences from UniProt (SwissProt + TrEMBL; release 2019_10), annotated with one of the KEGG orthologs from the targeted KEGG reactions, were retrieved (using information available from https://www.kegg.jp; accessed Jan-2022). The retrieved UniProt sequences were compared against the *P. micropora* KR01 chromatophore genome using tBLASTn (v2.10.1+) with the resulting hits filtered using an *e*-value <1 × 10^−5^. If no hits remained after filtering, for all of the KO numbers associated with a given enzyme reaction, then that step was considered to be not encoded in the chromatophore genome of *P. micropora* KR01. To assess if the target enzymes are missing from the nuclear genome, a *de novo* transcriptome was assembled for *P. micropora* KR01 by Trinity (v2.9.0; --SS_lib_type RF; (Grabherr et al., 2011; Haas et al., 2013), dependencies: jellyfish v2.3.0 (Marcais and Kingsford, 2011), Salmon v1.1.0 (Patro et al., 2017), and bowtie2 v2.3.5.1 (Langmead and Salzberg, 2012)) using the RNA-seq reads from BioProject PRJNA568118 (Lhee et al., 2021) (reads are the same a described previously and were trimmed using the same parameters). The assembled transcripts were annotated with KO numbers by KAAS (http://www.genome.jp/kegg/kaas/; query type: “nuc”; program: “BLAST”; method: “SBH” (Moriya et al., 2007)) using both the eukaryotic and prokaryotic gene datasets. The transcript annotations were then queried for the KO numbers associated with the targeted enzyme reactions; if no transcripts were annotated with any of the KO numbers associated with a given enzyme reaction, then that step was considered to be not encoded in the nuclear genome of *P. micropora* KR01.

### Quantification and statistical analysis

For gene expression quantification analysis (Figures S1 and S3) three replicate samples were available per condition, per time point; the average expression value (calculated from the three replicate samples) is shown for each condition and time point.

## Data Availability

• Manually corrected gene models and their associated alignments and phylogenetic trees have been deposited at Zenodo and are publicly available as of the date of publication. The DOI is listed in the key resources table. • All original code has been deposited at GitHub and is publicly available as of the date of publication. The link is listed in the key resources table. • Any additional information required to reanalyze the data reported in this paper is available from the lead contact on request.

## Supplemental information

Document S1. Figures S1–S3

Table S1. Original KR01 genes annotated as part of the KEGG pathways and description of new RNA-seq verified gene models, related to Tables 1–3 and Figures 1–3

Table S2. KR01 genes annotated as part of the KEGG pathways (nuclear genes corrected using RNA-seq) with information about crTP, BLASTp top hit, and phylogeny information shown, related to Tables 1–3 and Figures 1–3

Table S3. Results from the seach for key missing enzymes in the KR01 nuclear and chromatophore genomes. Related to Tables 1 and 2 and Figures 1 and 2

Data S1. Phylogenetic trees of KEGG orthologs listed in Table S2; also related to Tables 1-3 and Figures 1-3 Node support values are based on 2,000 ultrafast bootstrap approximations; nodes with support ≥ 95% are annotated with black circles. Sequence labels are colored based on the taxonomic domain of the organism; the internal branches of monophyletic clades are colored the same as its members, polyphyletic clades are colored black. *Paulinella* sequences with predicted transit peptides are shown using colored hexagons. The legend in the top left described the colors and shapes used in each tree. The AliStat completeness score for each sequence (Cr) is presented next to the alignment in the tree. This score represents the proportion of unambiguous characters for a given sequence in the alignment divided by the total number of sites in the alignment. There for, the closer this value is to 1.0 the more “complete” the sequence is in the alignment (i.e., fewer gaps), and conversely the close to 0 this score is the more incomplete the sequence is in the alignment (i.e., more gaps).

Data S2. Phylogenetic tree showing the P. micropora KR01 ribonucleoside-triphosphate reductase (thioredoxin; K00527) proteins along with protein sequences retrieved using BLASTp from a taxonomically broad local database Node support values are based on 2,000 ultrafast bootstrap approximations; nodes with support ≥ 95% are annotated with black circles. Sequence labels are colored based on the taxonomic domain of the organism; the internal branches of monophyletic clades are colored the same as its members, polyphyletic clades are colored black. *Paulinella* sequences with predicted transit peptides are shown using colored hexagons. The legend in the top left described the colors and shapes used in the Figure. Related to Tables 1 and 2 and Figures 1 and 2.

Data S3. Phylogenetic tree showing the P. micropora KR01 uridine kinase (K00876) proteins along with protein sequences retrieved using BLASTp from a taxonomically broad local database Node support values are based on 2,000 ultrafast bootstrap approximations; nodes with support ≥ 95% are annotated with black circles. Sequence labels are colored based on the taxonomic domain of the organism; the internal branches of monophyletic clades are colored the same as its members, polyphyletic clades are colored black. *Paulinella* sequences with predicted transit peptides are shown using colored hexagons. The legend in the top left described the colors and shapes used in the Figure. Related to Table 2 and Figure 2.

Data S4. Phylogenetic tree showing the P. micropora KR01 DNA polymerase I (K02335) proteins along with protein sequences retrieved using BLASTp from a taxonomically broad local database Node support values are based on 2,000 ultrafast bootstrap approximations; nodes with support ≥ 95% are annotated with black circles. Sequence labels are colored based on the taxonomic Domain of the organism; the internal branches of monophyletic clades are colored the same as its members, polyphyletic clades are colored black. *Paulinella* sequences with predicted transit peptides are shown using colored hexagons. The legend in the top left described the colors and shapes used in the Figure, Related to Table 3 and Figure 3.

Data S5. Phylogenetic tree showing the P. micropora KR01 DNA polymerase III (K02342) proteins along with protein sequences retrieved using BLASTp from a taxonomically broad local database Node support values are based on 2,000 ultrafast bootstrap approximations; nodes with support ≥ 95% are annotated with black circles. Sequence labels are colored based on the taxonomic Domain of the organism; the internal branches of monophyletic clades are colored the same as its members, polyphyletic clades are colored black. *Paulinella* sequences with predicted transit peptides are shown using colored hexagons. The legend in the top left described the colors and shapes used in the Figure. Related to Table 3 and Figure 3.
