# Supplementary material for: Loss of key endosymbiont genes may facilitate early host control of the chromatophore in Paulinella
Source: iScience. 2022 Aug 17;25(9):104974. doi: 10.1016/j.isci.2022.104974 (PMC9450145; doi:10.1016/j.isci.2022.104974)
Supplement: Document S1. Figures S1–S3 [file mmc1.pdf]

iScience, Volume *25*

Supplemental information

Loss of key endosymbiont genes may facilitate early host control of the chromatophore in *Paulinella*

Arwa Gabr, Timothy G. Stephens, and Debashish Bhattacharya

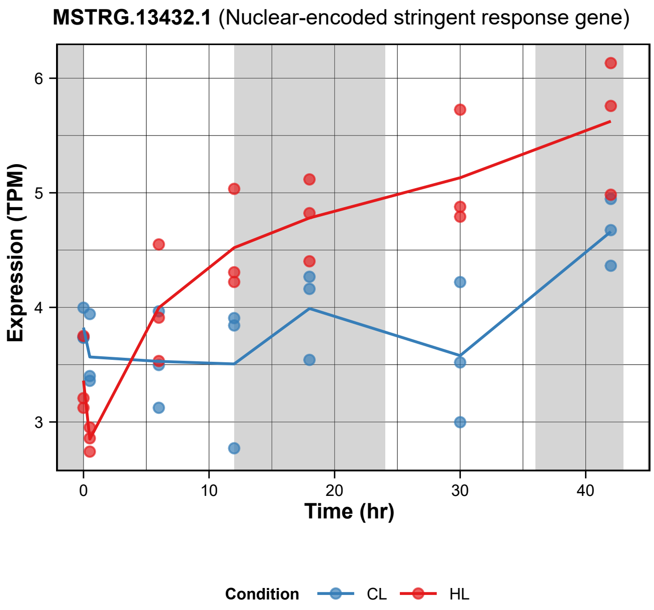

Figure S1: Expression of the nuclear-encoded stringent response gene (MSTRG.13432.1.p1) over a 48hr diurnal cycle under control and high light conditions. Gray shading represents the dark periods of the cycle. Expression values are in transcripts per million (TPM). Each dot represents a replicate sample (n = 3 per condition, per time point) and each line represents the average expression (across the three replicates per condition, per time point). Related to Table 1 and Figure 1.

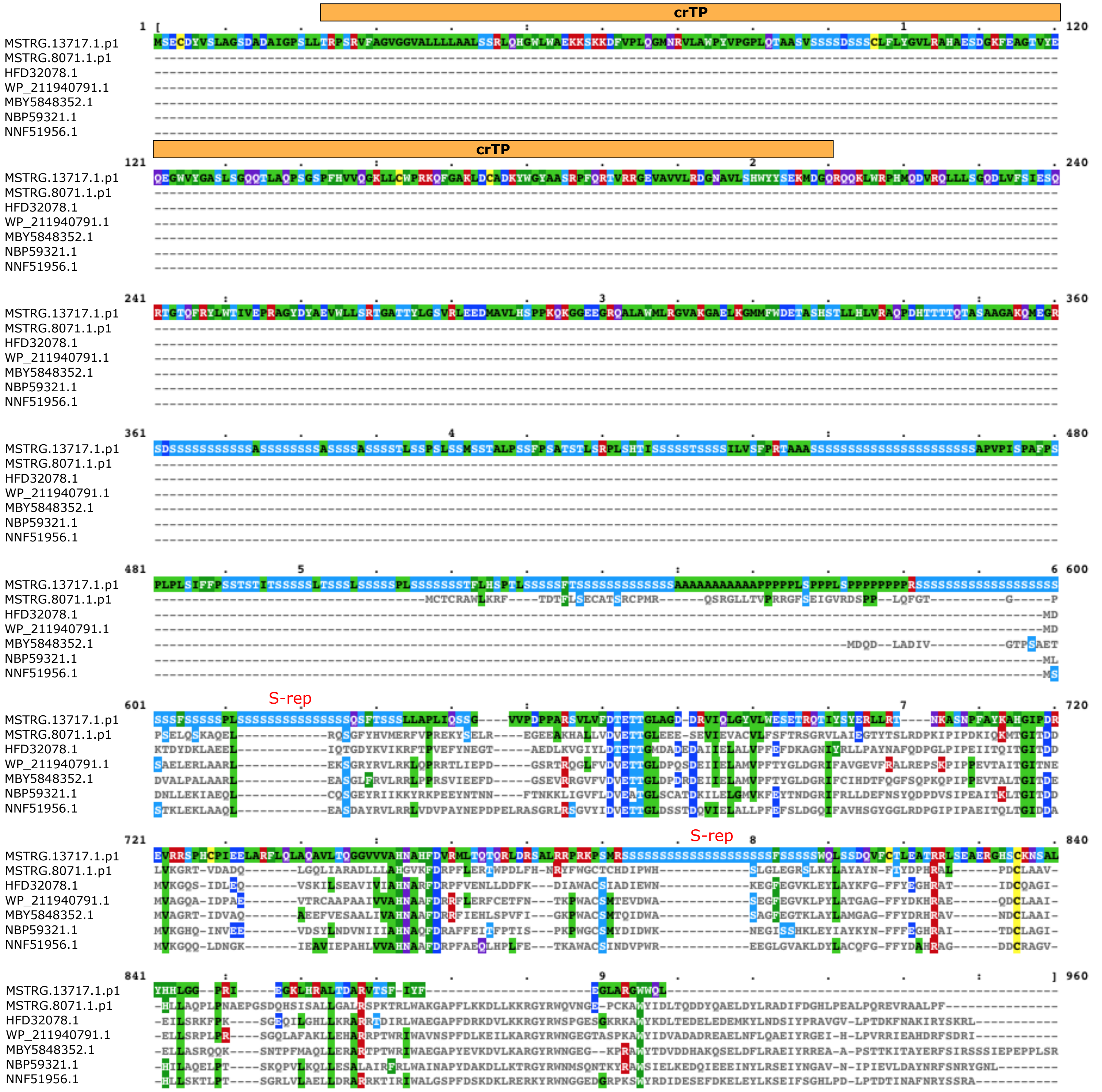

Figure S2: Alignment of the two *P. micropora* KR01 DNA polymerase III proteins (MSTRG. 13717.1.p1 and MSTRG.8071.1.p1) and five proteins from NCBI nr with the highest scoring hits (using the NCBIs online BLASTp tool; Jan 2022) to MSTRG.8071.1.p1. Sequences were aligned using Clustal Omega (https://www.ebi.ac.uk/Tools/msa/clustalo/; ORDER=input) and visualized using MView (ALIGNMENT WIDTH=120, CONSENSUS=OFF). The predicted crTP in MSTRG.13717.1.p1 is shown using a light orange colored bar; “S-rep” are the serine repeats that interrupt the conserved region of the protein. Related to Table 3 and Figure 3.

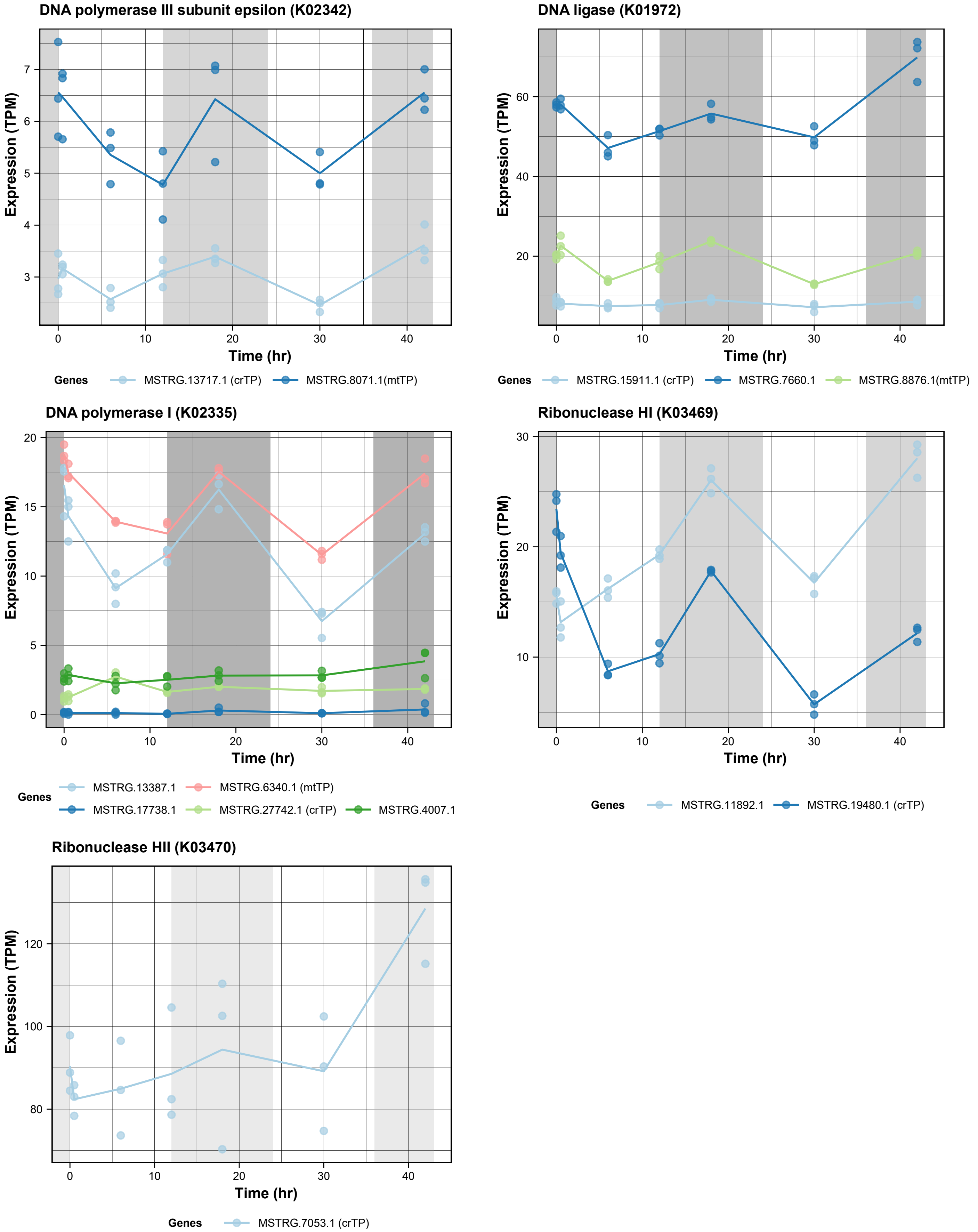

Figure S3: Expression of manually corrected nuclear-encoded bacterial DNA replication pathways genes over a 48hr diurnal cycle under control light conditions. Gray shading represents the dark periods of the cycle. Genes are grouped and plotted according to which replication protein they are annotated with and expression values are in transcripts per million (TPM). Each dot represents a replicate sample (n = 3 per condition, per time point) and each line represents the average expression (across the three replicates per condition, per time point). Related to Table 3 and Figure 3.
