# Supplementary material for: Loss of key endosymbiont genes may facilitate early host control of the chromatophore in Paulinella
Source: iScience. 2022 Aug 17;25(9):104974. doi: 10.1016/j.isci.2022.104974 (PMC9450145; doi:10.1016/j.isci.2022.104974)

DNA polymerase III (K02342)

Groups

- *Paulinella*
- Archaea
- Bacteria
- Eukaryota
- Viruses
- Other/Misc

Nodes

- *BS* ≥ 95%

BS: ultrafast bootstrap support

Organelle targeting peptides

- Mitochondrial
- Chromatophore (≥100aa)
- Chromatophore (<100aa)

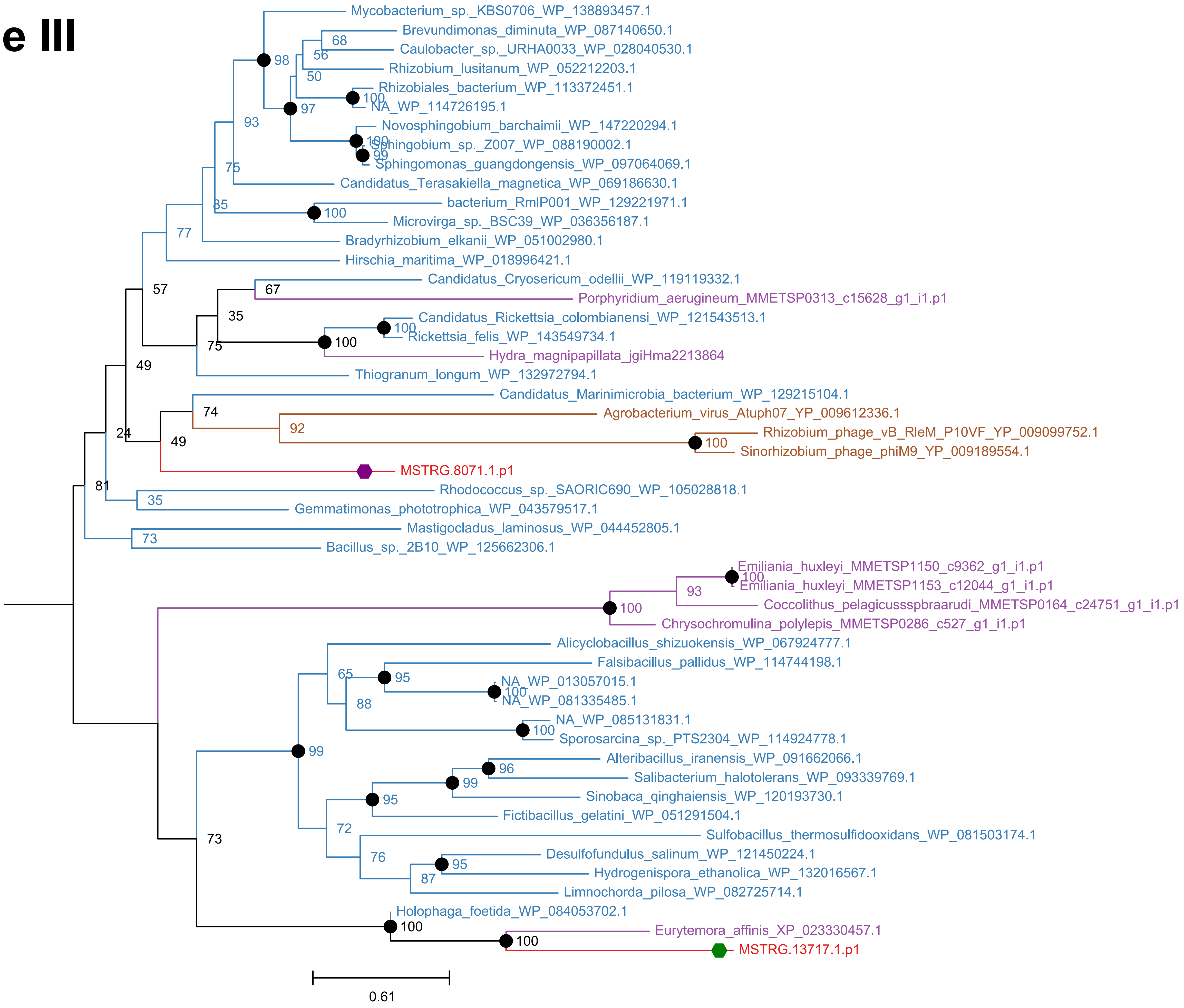

Supplement: Data S5. Phylogenetic tree showing the P. micropora KR01 DNA polymerase III (K02342) proteins along with protein sequences retrieved using BLASTp from a taxonomically broad local database — Node support values are based on 2,000 ultrafast bootstrap approximations; nodes with support ≥ 95% are annotated with black circles. Sequence labels are colored based on the taxonomic Domain of the organism; the internal branches of monophyletic clades are colored the same as its members, polyphyletic clades are colored black. Paulinella sequences with predicted transit peptides are shown using colored hexagons. The legend in the top left described the colors and shapes used in the Figure. Related to Table 3 and Figure 3. [file mmc9.pdf]
