# Supplementary material for: Loss of key endosymbiont genes may facilitate early host control of the chromatophore in Paulinella
Source: iScience. 2022 Aug 17;25(9):104974. doi: 10.1016/j.isci.2022.104974 (PMC9450145; doi:10.1016/j.isci.2022.104974)
Supplement: Data S1. Phylogenetic trees of KEGG orthologs listed in Table S2; also related to Tables 1-3 and Figures 1-3 — Node support values are based on 2,000 ultrafast bootstrap approximations; nodes with support ≥ 95% are annotated with black circles. Sequence labels are colored based on the taxonomic domain of the organism; the internal branches of monophyletic clades are colored the same as its members, polyphyletic clades are colored black. Paulinella sequences with predicted transit peptides are shown using colored hexagons. The legend in the top left described the colors and shapes used in each tree. The AliStat completeness score for each sequence (Cr) is presented next to the alignment in the tree. This score represents the proportion of unambiguous characters for a given sequence in the alignment divided by the total number of sites in the alignment. There for, the closer this value is to 1.0 the more “complete” the sequence is in the alignment (i.e., fewer gaps), and conversely the close to 0 this score is the more incomplete the sequence is in the alignment (i.e., more gaps). [file mmc5.pdf]

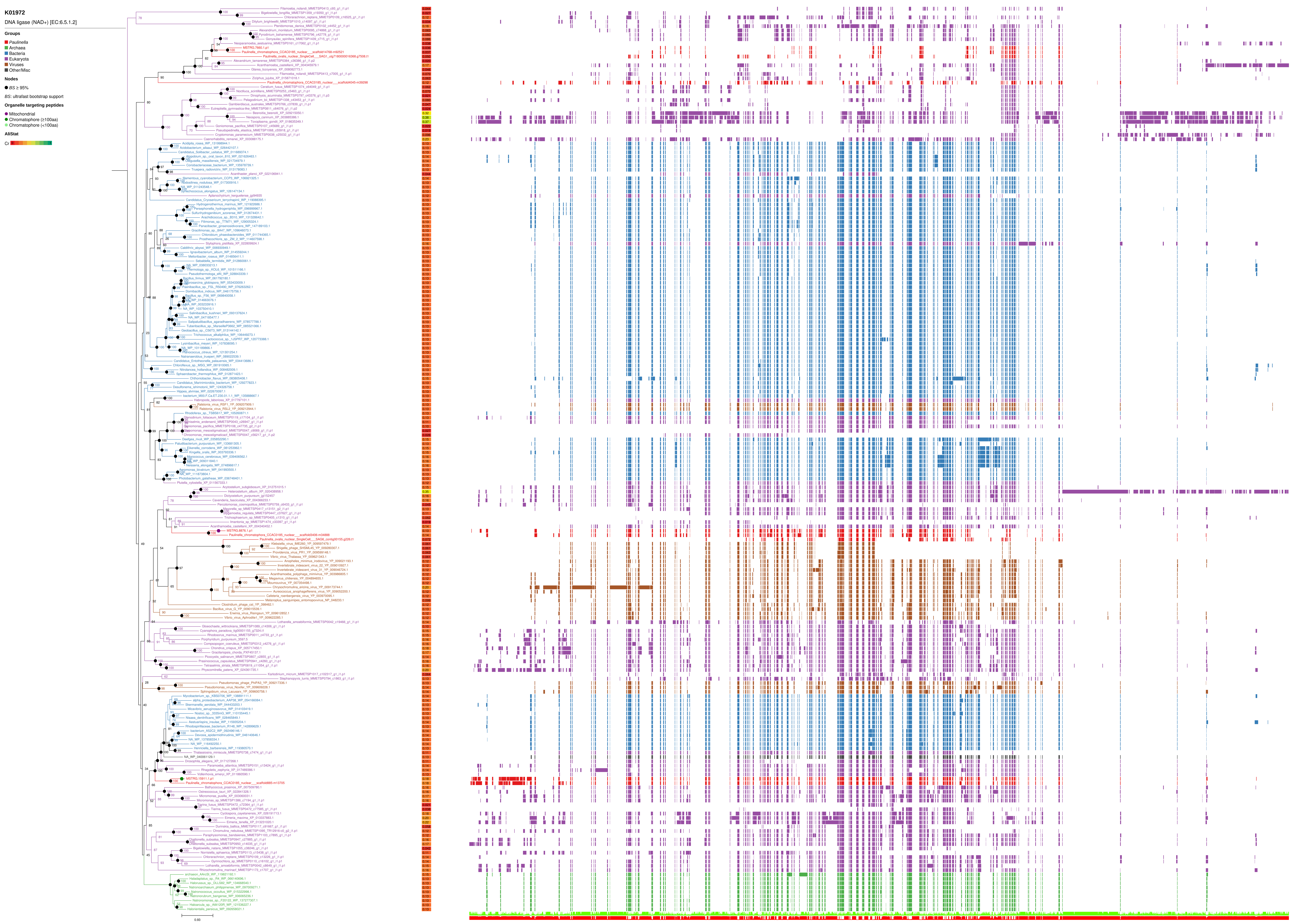
K01972
DNA ligase (NAD+) [EC:6.5.1.2]
Groups
Paulinella
Archaea
Bacteria
Eukaryota
Viruses
Other/Misc
Nodes
BS ≥ 95%
BS: ultrafast bootstrap support
Organelle targeting peptides
Mitochondrial
Chromatophore (≥100aa)
Chromatophore (<100aa)
AliStat
Cr
0.93

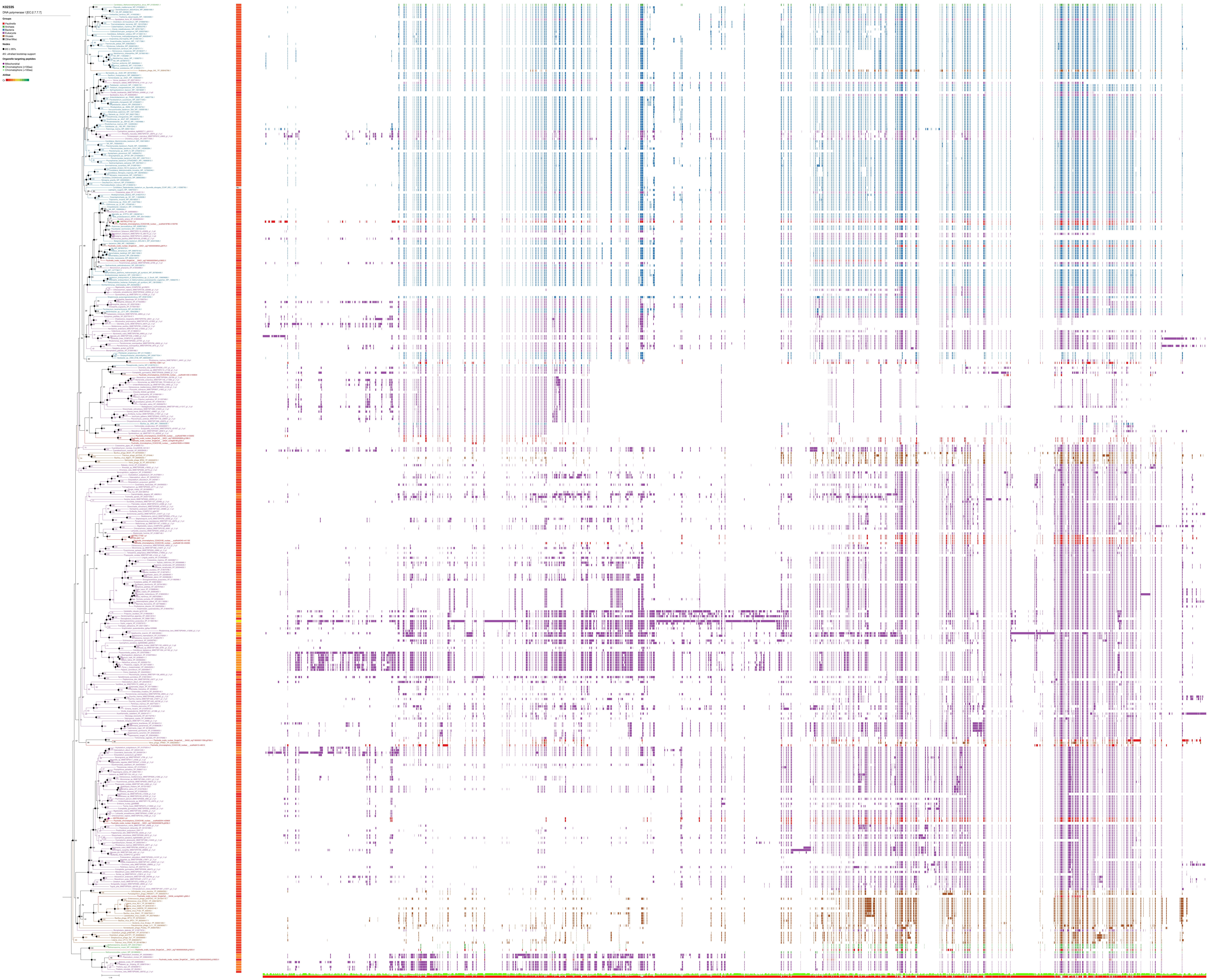

K02335
DNA polymerase I [EC:2.7.7.7]
Groups
Paulinella
Archaea
Bacteria
Eukaryota
Viruses
Other/Misc
Nodes
BS ≥ 95%
BS: ultrafast bootstrap support
Organelle targeting peptides
Mitochondrial
Chromatophore (≥100aa)
Chromatophore (<100aa)
AliStat
Cr 0 1

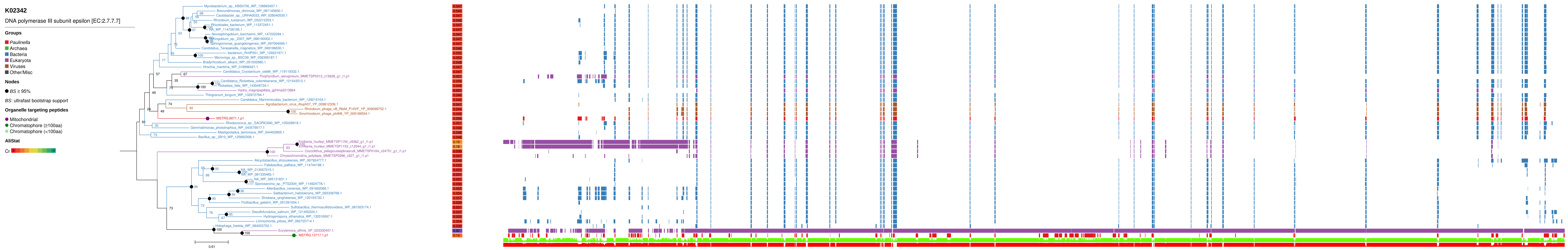

K02342
DNA polymerase III subunit epsilon [EC:2.7.7.7]
Groups
Paulinella
Archaea
Bacteria
Eukaryota
Viruses
Other/Misc
Nodes
BS ≥ 95%
BS: ultrafast bootstrap support
Organelle targeting peptides
Mitochondrial
Chromatophore (≥100aa)
Chromatophore (<100aa)
AliStat
Cr 0 1
Mycobacterium_sp._KBS0706_WP_138893457.1
Brevundimonas_diminuta_WP_087140650.1
Caulobacter_sp._URHA0033_WP_028040530.1
Rhizobium_lusitanum_WP_052212203.1
Rhizobiales_bacterium_WP_113372451.1
NA_WP_114726195.1
Novosphingobium_barchaimii_WP_147220294.1
Sphingobium_sp._Z007_WP_088190002.1
Sphingomonas_guangdongensis_WP_097064069.1
Candidatus_Terasakiella_magnetica_WP_069186630.1
bacterium_RmlP001_WP_129221971.1
Microvirga_sp._BSC39_WP_036356187.1
Bradyrhizobium_elkanii_WP_051002980.1
Hirschia_maritima_WP_018996421.1
Candidatus_Cryosericum_odellii_WP_119119332.1
Porphyridium_aerugineum_MMETSP0313_c15628_g1_i1.p1
Candidatus_Rickettsia_colombianensi_WP_121543513.1
Rickettsia_felis_WP_143549734.1
Hydra_magnipapillata_jgiHma2213864
Thiogranum_longum_WP_132972794.1
Candidatus_Marinimicrobia_bacterium_WP_129215104.1
Agrobacterium_virus_Atuph07_YP_009612336.1
Rhizobium_phage_vB_RleM_P10VF_YP_009099752.1
Sinorhizobium_phage_phiM9_YP_009189554.1
MSTRG.8071.1.p1
Rhodococcus_sp._SAORIC690_WP_105028818.1
Gemmatimonas_phototrophica_WP_043579517.1
Mastigocladus_laminosus_WP_044452805.1
Bacillus_sp._2B10_WP_125662306.1
Emiliania_huxleyi_MMETSP1150_c9362_g1_i1.p1
Emiliania_huxleyi_MMETSP1153_c12044_g1_i1.p1
Coccolithus_pelagicussspbraarudi_MMETSP0164_c24751_g1_i1.p1
Chrysochromulina_polylepis_MMETSP0286_c527_g1_i1.p1
Alicyclobacillus_shizuokensis_WP_067924777.1
Falsibacillus_pallidus_WP_114744198.1
NA_WP_013057015.1
NA_WP_081335485.1
NA_WP_085131831.1
Sporosarcina_sp._PTS2304_WP_114924778.1
Alteribacillus_iranensis_WP_091662066.1
Salibacterium_halotolerans_WP_093339769.1
Sinobaca_qinghaiensis_WP_120193730.1
Fictibacillus_gelatini_WP_051291504.1
Sulfobacillus_thermosulfidooxidans_WP_081503174.1
Desulfofundulus_salinum_WP_121450224.1
Hydrogenispora_ethanolica_WP_132016567.1
Limnochorda_pilosa_WP_082725714.1
Holophaga_foetida_WP_084053702.1
Eurytemora_affinis_XP_023330457.1
MSTRG.13717.1.p1
0.61

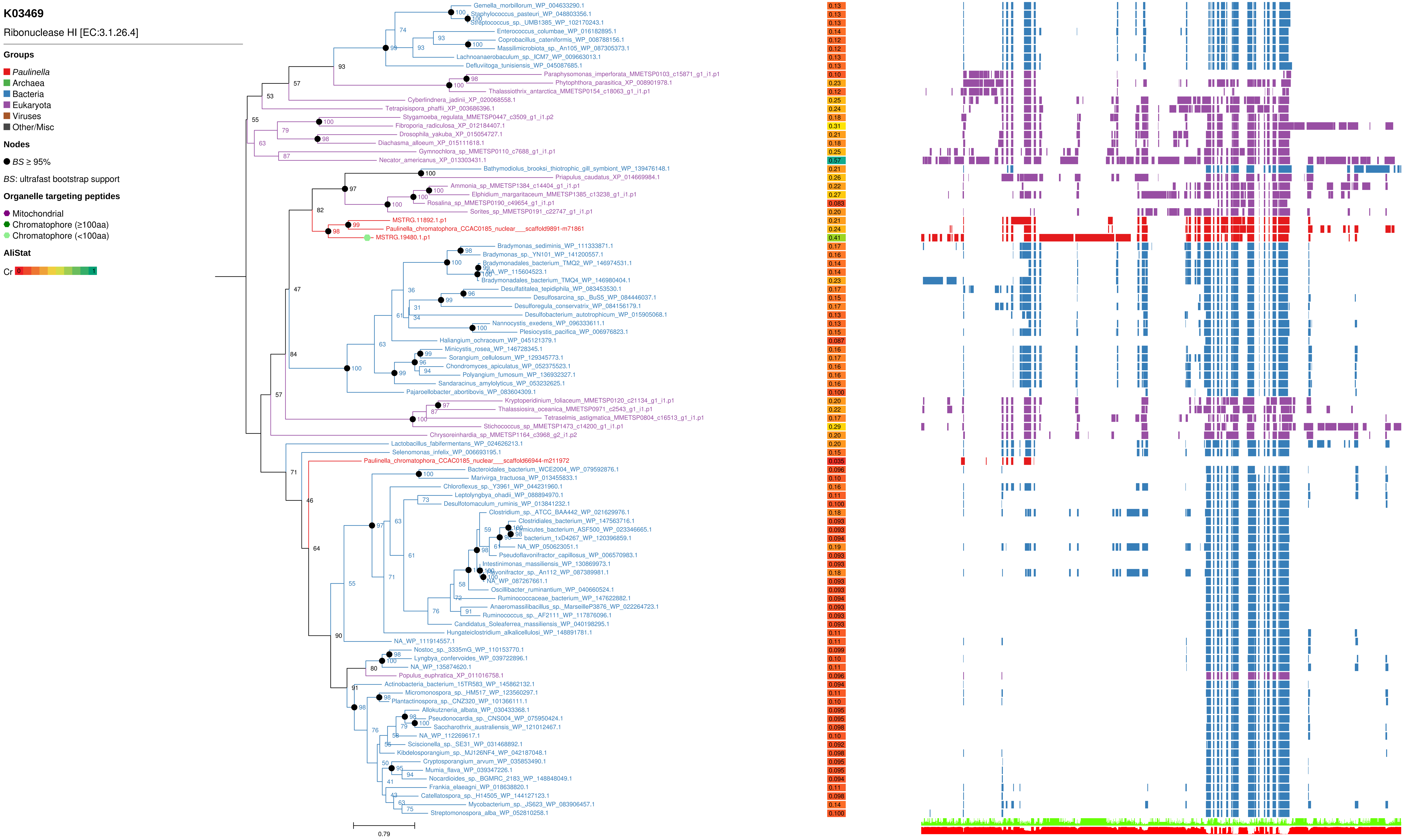

K03469
Ribonuclease HI [EC:3.1.26.4]
Groups
Paulinella
Archaea
Bacteria
Eukaryota
Viruses
Other/Misc
Nodes
BS ≥ 95%
BS: ultrafast bootstrap support
Organelle targeting peptides
Mitochondrial
Chromatophore (≥100aa)
Chromatophore (<100aa)
AliStat
Cr 0 1
Gemella_morbillorum_WP_004633290.1
Staphylococcus_pasteuri_WP_048803356.1
Streptococcus_sp._UMB1385_WP_102170243.1
Enterococcus_columbae_WP_016182895.1
Coprobacillus_cateniformis_WP_008788156.1
Massilimicrobiota_sp._An105_WP_087305373.1
Lachnoanaerobaculum_sp._ICM7_WP_009663013.1
Defluviitoga_tunisiensis_WP_045087685.1
Paraphysomonas_imperforata_MMETSP0103_c15871_g1_i1.p1
Phytophthora_parasitica_XP_008901978.1
Thalassiothrix_antarctica_MMETSP0154_c18063_g1_i1.p1
Cyberlindnera_jadinii_XP_020068558.1
Tetrapisispora_phaffii_XP_003686396.1
Stygamoeba_regulata_MMETSP0447_c3509_g1_i1.p2
Fibroporia_radiculosa_XP_012184407.1
Drosophila_yakuba_XP_015054727.1
Diachasma_alloeum_XP_015111618.1
Gymnochlora_sp_MMETSP0110_c7688_g1_i1.p1
Necator_americanus_XP_013303431.1
Bathymodiolus_brooksi_thiotrophic_gill_symbiont_WP_139476148.1
Priapulus_caudatus_XP_014669984.1
Ammonia_sp_MMETSP1384_c14404_g1_i1.p1
Elphidium_margaritaceum_MMETSP1385_c13238_g1_i1.p1
Rosalina_sp_MMETSP0190_c49654_g1_i1.p1
Sorites_sp_MMETSP0191_c22747_g1_i1.p1
MSTRG.11892.1.p1
Paulinella_chromatophora_CCAC0185_nuclear___scaffold9891-m71861
MSTRG.19480.1.p1
Bradymonas_sediminis_WP_111333871.1
Bradymonas_sp._YN101_WP_141200557.1
Bradymonadales_bacterium_TMQ2_WP_146974531.1
NA_WP_115604523.1
Bradymonadales_bacterium_TMQ4_WP_146980404.1
Desulfatitalea_tepidiphila_WP_083453530.1
Desulfosarcina_sp._BuS5_WP_084446037.1
Desulforegula_conservatrix_WP_084156179.1
Desulfobacterium_autotrophicum_WP_015905068.1
Nannocystis_exedens_WP_096333611.1
Plesiocystis_pacifica_WP_006976823.1
Haliangium_ochraceum_WP_045121379.1
Minicystis_rosea_WP_146728345.1
Sorangium_cellulosum_WP_129345773.1
Chondromyces_apiculatus_WP_052375523.1
Polyangium_fumosum_WP_136932327.1
Sandaracinus_amylolyticus_WP_053232625.1
Pajaroellobacter_abortibovis_WP_083604309.1
Kryptoperidinium_foliaceum_MMETSP0120_c21134_g1_i1.p1
Thalassiosira_oceanica_MMETSP0971_c2543_g1_i1.p1
Tetraselmis_astigmatica_MMETSP0804_c16513_g1_i1.p1
Stichococcus_sp_MMETSP1473_c14200_g1_i1.p1
Chrysoreinhardia_sp_MMETSP1164_c3968_g2_i1.p2
Lactobacillus_fabifermentans_WP_024626213.1
Selenomonas_infelix_WP_006693195.1
Paulinella_chromatophora_CCAC0185_nuclear___scaffold66944-m211972
Bacteroidales_bacterium_WCE2004_WP_079592876.1
Marivirga_tractuosa_WP_013455833.1
Chloroflexus_sp._Y3961_WP_044231960.1
Leptolyngbya_ohadii_WP_088894970.1
Desulfotomaculum_ruminis_WP_013841232.1
Clostridium_sp._ATCC_BAA442_WP_021629976.1
Clostridiales_bacterium_WP_147563716.1
Firmicutes_bacterium_ASF500_WP_023346665.1
bacterium_1xD4267_WP_120396859.1
NA_WP_050623051.1
Pseudoflavonifractor_capillosus_WP_006570983.1
Intestinimonas_massiliensis_WP_130869973.1
Flavonifractor_sp._An112_WP_087389981.1
NA_WP_087267661.1
Oscillibacter_ruminantium_WP_040660524.1
Ruminococcaceae_bacterium_WP_147622882.1
Anaeromassilibacillus_sp._MarseilleP3876_WP_022264723.1
Ruminococcus_sp._AF2111_WP_117876096.1
Candidatus_Soleaferrea_massiliensis_WP_040198295.1
Hungateiclostridium_alkalicellulosi_WP_148891781.1
NA_WP_111914557.1
Nostoc_sp._3335mG_WP_110153770.1
Lyngbya_confervoides_WP_039722896.1
NA_WP_135874620.1
Populus_euphratica_XP_011016758.1
Actinobacteria_bacterium_15TR583_WP_145862132.1
Micromonospora_sp._HM517_WP_123560297.1
Plantactinospora_sp._CNZ320_WP_101366111.1
Allokutzneria_albata_WP_030433368.1
Pseudonocardia_sp._CNS004_WP_075950424.1
Saccharothrix_australiensis_WP_121012467.1
NA_WP_112269617.1
Sciscionella_sp._SE31_WP_031468892.1
Kibdelosporangium_sp._MJ126NF4_WP_042187048.1
Cryptosporangium_arvum_WP_035853490.1
Mumia_flava_WP_039347226.1
Nocardioides_sp._BGMRC_2183_WP_148848049.1
Frankia_elaeagni_WP_018638820.1
Catellatospora_sp._H14505_WP_144127123.1
Mycobacterium_sp._JS623_WP_083906457.1
Streptomonospora_alba_WP_052810258.1
0.79

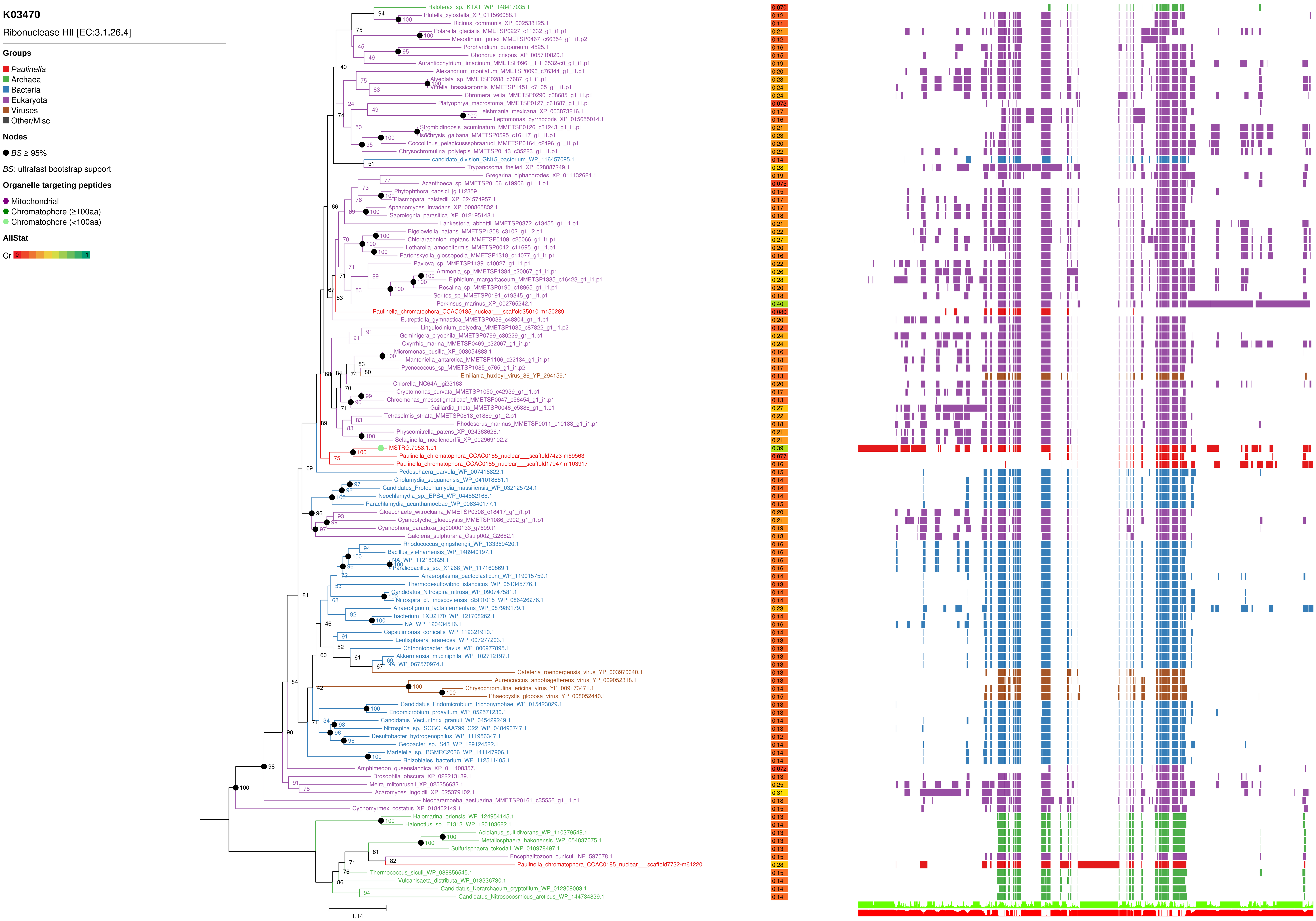

K03470
Ribonuclease HII [EC:3.1.26.4]
Groups
Paulinella
Archaea
Bacteria
Eukaryota
Viruses
Other/Misc
Nodes
BS ≥ 95%
BS: ultrafast bootstrap support
Organelle targeting peptides
Mitochondrial
Chromatophore (≥100aa)
Chromatophore (<100aa)
AliStat
Cr
Haloferax_sp._KTX1_WP_148417035.1
Plutella_xylostella_XP_011566088.1
Ricinus_communis_XP_002538125.1
Polarella_glacialis_MMETSP0227_c11632_g1_i1.p1
Mesodinium_pulex_MMETSP0467_c66354_g1_i1.p2
Porphyridium_purpureum_4525.1
Chondrus_crispus_XP_005710820.1
Aurantiochytrium_limacinum_MMETSP0961_TR16532-c0_g1_i1.p1
Alexandrium_monilatum_MMETSP0093_c76344_g1_i1.p1
Alveolata_sp_MMETSP0288_c7687_g1_i1.p1
Vitrella_brassicaformis_MMETSP1451_c7105_g1_i1.p1
Chromera_velia_MMETSP0290_c38685_g1_i1.p1
Platyophrya_macrostoma_MMETSP0127_c61687_g1_i1.p1
Leishmania_mexicana_XP_003873216.1
Leptomonas_pyrrhocoris_XP_015655014.1
Strombidinopsis_acuminatum_MMETSP0126_c31243_g1_i1.p1
Isochrysis_galbana_MMETSP0595_c16117_g1_i1.p1
Coccolithus_pelagicussspbraarudi_MMETSP0164_c2496_g1_i1.p1
Chrysochromulina_polylepis_MMETSP0143_c35223_g1_i1.p1
candidate_division_GN15_bacterium_WP_116457095.1
Trypanosoma_theileri_XP_028887249.1
Gregarina_niphandrodes_XP_011132624.1
Acanthoeca_sp_MMETSP0106_c19906_g1_i1.p1
Phytophthora_capsici_jgi112359
Plasmopara_halstedii_XP_024574957.1
Aphanomyces_invadans_XP_008865832.1
Saprolegnia_parasitica_XP_012195148.1
Lankesteria_abbottii_MMETSP0372_c13455_g1_i1.p1
Bigelowiella_natans_MMETSP1358_c3102_g1_i2.p1
Chlorarachnion_reptans_MMETSP0109_c25066_g1_i1.p1
Lotharella_amoebiformis_MMETSP0042_c11695_g1_i1.p1
Partenskyella_glossopodia_MMETSP1318_c14077_g1_i1.p1
Pavlova_sp_MMETSP1139_c10027_g1_i1.p1
Ammonia_sp_MMETSP1384_c20067_g1_i1.p1
Elphidium_margaritaceum_MMETSP1385_c16423_g1_i1.p1
Rosalina_sp_MMETSP0190_c18965_g1_i1.p1
Sorites_sp_MMETSP0191_c19345_g1_i1.p1
Perkinsus_marinus_XP_002765242.1
Paulinella_chromatophora_CCAC0185_nuclear___scaffold35010-m150289
Eutreptiella_gymnastica_MMETSP0039_c48304_g1_i1.p1
Lingulodinium_polyedra_MMETSP1035_c87822_g1_i1.p2
Geminigera_cryophila_MMETSP0799_c30229_g1_i1.p1
Oxyrrhis_marina_MMETSP0469_c32067_g1_i1.p1
Micromonas_pusilla_XP_003054888.1
Mantoniella_antarctica_MMETSP1106_c22134_g1_i1.p1
Pycnococcus_sp_MMETSP1085_c765_g1_i1.p2
Emiliania_huxleyi_virus_86_YP_294159.1
Chlorella_NC64A_jgi23163
Cryptomonas_curvata_MMETSP1050_c42939_g1_i1.p1
Chroomonas_mesostigmaticacf_MMETSP0047_c56454_g1_i1.p1
Guillardia_theta_MMETSP0046_c5386_g1_i1.p1
Tetraselmis_striata_MMETSP0818_c1889_g1_i2.p1
Rhodosorus_marinus_MMETSP0011_c10183_g1_i1.p1
Physcomitrella_patens_XP_024368626.1
Selaginella_moellendorffii_XP_002969102.2
MSTRG.7053.1.p1
Paulinella_chromatophora_CCAC0185_nuclear___scaffold7423-m59563
Paulinella_chromatophora_CCAC0185_nuclear___scaffold17947-m103917
Pedosphaera_parvula_WP_007416822.1
Criblamydia_sequanensis_WP_041018651.1
Candidatus_Protochlamydia_massiliensis_WP_032125724.1
Neochlamydia_sp._EPS4_WP_044882168.1
Parachlamydia_acanthamoebae_WP_006340177.1
Gloeochaete_witrockiana_MMETSP0308_c18417_g1_i1.p1
Cyanoptyche_gloeocystis_MMETSP1086_c902_g1_i1.p1
Cyanophora_paradoxa_tig00000133_g7699.t1
Galdieria_sulphuraria_Gsulp002_G2682.1
Rhodococcus_qingshengii_WP_133369420.1
Bacillus_vietnamensis_WP_148940197.1
NA_WP_112180829.1
Paraliobacillus_sp._X1268_WP_117160869.1
Anaeroplasma_bactoclasticum_WP_119015759.1
Thermodesulfovibrio_islandicus_WP_051345776.1
Candidatus_Nitrospira_nitrosa_WP_090747581.1
Nitrospira_cf._moscoviensis_SBR1015_WP_086426276.1
Anaerotignum_lactatifermentans_WP_087989179.1
bacterium_1XD2170_WP_121708262.1
NA_WP_120434516.1
Capsulimonas_corticalis_WP_119321910.1
Lentisphaera_araneosa_WP_007277203.1
Chthoniobacter_flavus_WP_006977895.1
Akkermansia_muciniphila_WP_102712197.1
NA_WP_067570974.1
Cafeteria_roenbergensis_virus_YP_003970040.1
Aureococcus_anophagefferens_virus_YP_009052318.1
Chrysochromulina_ericina_virus_YP_009173471.1
Phaeocystis_globosa_virus_YP_008052440.1
Candidatus_Endomicrobium_trichonymphae_WP_015423029.1
Endomicrobium_proavitum_WP_052571230.1
Candidatus_Vecturithrix_granuli_WP_045429249.1
Nitrospina_sp._SCGC_AAA799_C22_WP_048493747.1
Desulfobacter_hydrogenophilus_WP_111956347.1
Geobacter_sp._S43_WP_129124522.1
Martelella_sp._BGMRC2036_WP_141147906.1
Rhizobiales_bacterium_WP_112511405.1
Amphimedon_queenslandica_XP_011408357.1
Drosophila_obscura_XP_022213189.1
Meira_miltonrushii_XP_025356633.1
Acaromyces_ingoldii_XP_025379102.1
Neoparamoeba_aestuarina_MMETSP0161_c35556_g1_i1.p1
Cyphomyrmex_costatus_XP_018402149.1
Halomarina_oriensis_WP_124954145.1
Halonotius_sp._F1313_WP_120103682.1
Acidianus_sulfidivorans_WP_110379548.1
Metallosphaera_hakonensis_WP_054837075.1
Sulfurisphaera_tokodaii_WP_010978497.1
Encephalitozoon_cuniculi_NP_597578.1
Paulinella_chromatophora_CCAC0185_nuclear___scaffold7732-m61220
Thermococcus_siculi_WP_088856545.1
Vulcanisaeta_distributa_WP_013336730.1
Candidatus_Korarchaeum_cryptofilum_WP_012309003.1
Candidatus_Nitrosocosmicus_arcticus_WP_144734839.1
1.14

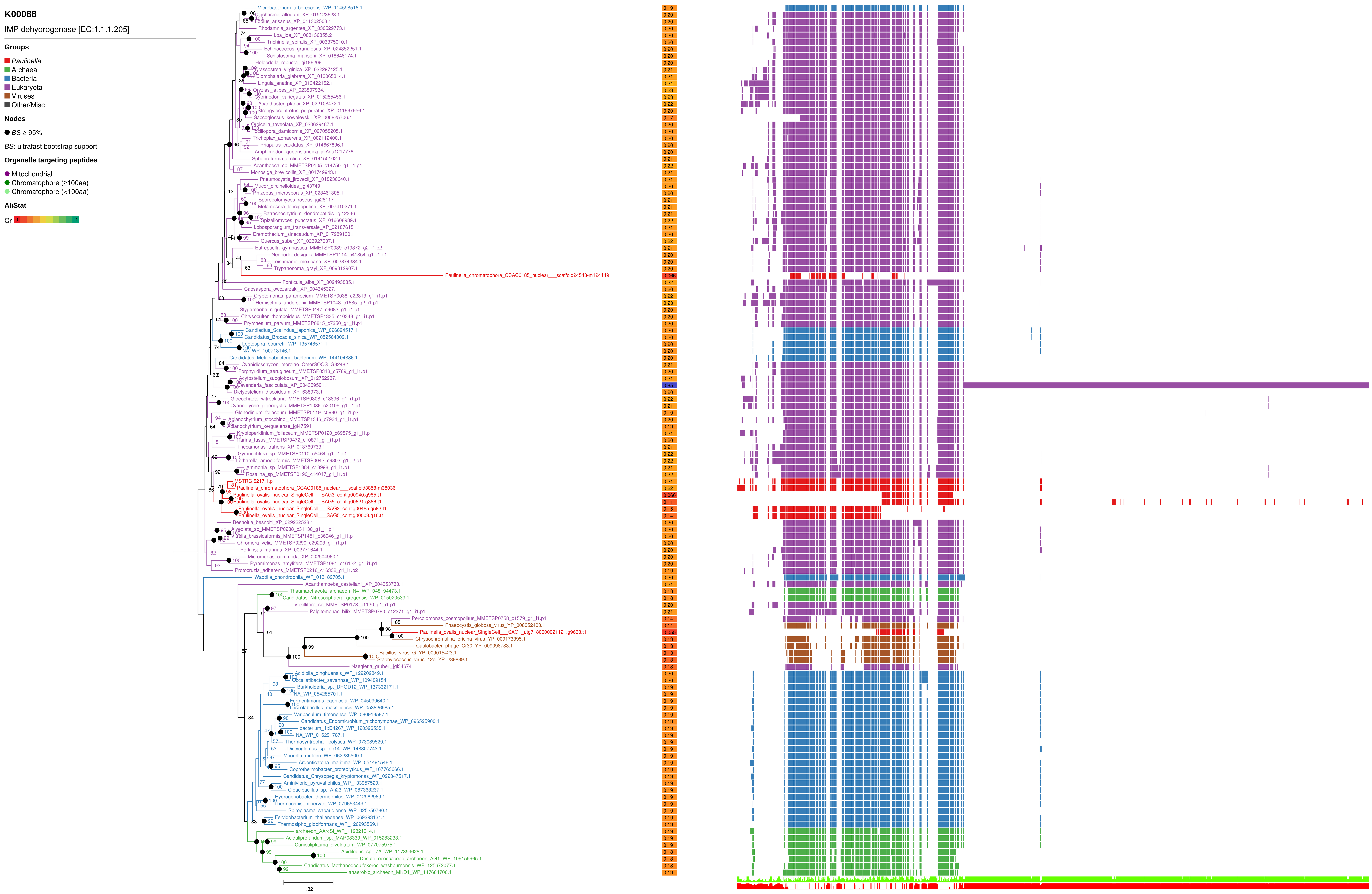

K00088
IMP dehydrogenase [EC:1.1.1.205]
Groups
Paulinella
Archaea
Bacteria
Eukaryota
Viruses
Other/Misc
Nodes
BS ≥ 95%
BS: ultrafast bootstrap support
Organelle targeting peptides
Mitochondrial
Chromatophore (≥100aa)
Chromatophore (<100aa)
AliStat
Cr 0 1
Microbacterium_arborescens_WP_114598516.1
Diachasma_alloeum_XP_015123628.1
Fopius_arisanus_XP_011302503.1
Rhodamnia_argentea_XP_030529773.1
Loa_loa_XP_003136355.2
Trichinella_spiralis_XP_003375010.1
Echinococcus_granulosus_XP_024352251.1
Schistosoma_mansoni_XP_018648174.1
Helobdella_robusta_jgi186209
Crassostrea_virginica_XP_022297425.1
Biomphalaria_glabrata_XP_013065314.1
Lingula_anatina_XP_013422152.1
Oryzias_latipes_XP_023807934.1
Cyprinodon_variegatus_XP_015255456.1
Acanthaster_planci_XP_022108472.1
Strongylocentrotus_purpuratus_XP_011667956.1
Saccoglossus_kowalevskii_XP_006825706.1
Orbicella_faveolata_XP_020629487.1
Pocillopora_damicornis_XP_027058205.1
Trichoplax_adhaerens_XP_002112400.1
Priapulus_caudatus_XP_014667896.1
Amphimedon_queenslandica_jgiAqu1217776
Sphaeroforma_arctica_XP_014150102.1
Acanthoeca_sp_MMETSP0105_c14750_g1_i1.p1
Monosiga_brevicollis_XP_001749943.1
Pneumocystis_jirovecii_XP_018230640.1
Mucor_circinelloides_jgi43749
Rhizopus_microsporus_XP_023461305.1
Sporobolomyces_roseus_jgi28117
Melampsora_laricipopulina_XP_007410271.1
Batrachochytrium_dendrobatidis_jgi12346
Spizellomyces_punctatus_XP_016608989.1
Lobosporangium_transversale_XP_021876151.1
Eremothecium_sinecaudum_XP_017989130.1
Quercus_suber_XP_023927037.1
Eutreptiella_gymnastica_MMETSP0039_c19372_g2_i1.p2
Neobodo_designis_MMETSP1114_c41854_g1_i1.p1
Leishmania_mexicana_XP_003874334.1
Trypanosoma_grayi_XP_009312907.1
Paulinella_chromatophora_CCAC0185_nuclear___scaffold24548-m124149
Fonticula_alba_XP_009493835.1
Capsaspora_owczarzaki_XP_004345327.1
Cryptomonas_paramecium_MMETSP0038_c22813_g1_i1.p1
Hemiselmis_andersenii_MMETSP1043_c1685_g2_i1.p1
Stygamoeba_regulata_MMETSP0447_c9683_g1_i1.p1
Chrysoculter_rhomboideus_MMETSP1335_c10343_g1_i1.p1
Prymnesium_parvum_MMETSP0815_c7250_g1_i1.p1
Candidatus_Scalindua_japonica_WP_096894517.1
Candidatus_Brocadia_sinica_WP_052564009.1
Leptospira_bourretii_WP_135748571.1
NA_WP_100718146.1
Candidatus_Melainabacteria_bacterium_WP_144104886.1
Cyanidioschyzon_merolae_CmerSOOS_G3248.1
Porphyridium_aerugineum_MMETSP0313_c5769_g1_i1.p1
Acytostelium_subglobosum_XP_012752937.1
Cavenderia_fasciculata_XP_004359521.1
Dictyostelium_discoideum_XP_638973.1
Gloeochaete_witrockiana_MMETSP0308_c18896_g1_i1.p1
Cyanoptyche_gloeocystis_MMETSP1086_c20109_g1_i1.p1
Glenodinium_foliaceum_MMETSP0119_c5980_g1_i1.p2
Aplanochytrium_stocchinoi_MMETSP1346_c7934_g1_i1.p1
Aplanochytrium_kerguelense_jgi47591
Kryptoperidinium_foliaceum_MMETSP0120_c69875_g1_i1.p1
Tarina_fusus_MMETSP0472_c10871_g1_i1.p1
Thecamonas_trahens_XP_013760733.1
Gymnochlora_sp_MMETSP0110_c5464_g1_i1.p1
Lotharella_amoebiformis_MMETSP0042_c9803_g1_i2.p1
Ammonia_sp_MMETSP1384_c18998_g1_i1.p1
Rosalina_sp_MMETSP0190_c14017_g1_i1.p1
MSTRG.5217.1.p1
Paulinella_chromatophora_CCAC0185_nuclear___scaffold3858-m38036
Paulinella_ovalis_nuclear_SingleCell___SAG3_contig00940.g985.t1
Paulinella_ovalis_nuclear_SingleCell___SAG5_contig00621.g866.t1
Paulinella_ovalis_nuclear_SingleCell___SAG3_contig00465.g583.t1
Paulinella_ovalis_nuclear_SingleCell___SAG5_contig00003.g16.t1
Besnoitia_besnoiti_XP_029222528.1
Alveolata_sp_MMETSP0288_c31130_g1_i1.p1
Vitrella_brassicaformis_MMETSP1451_c36946_g1_i1.p1
Chromera_velia_MMETSP0290_c29293_g1_i1.p1
Perkinsus_marinus_XP_002771644.1
Micromonas_commoda_XP_002504960.1
Pyramimonas_amylifera_MMETSP1081_c16122_g1_i1.p1
Protocruzia_adherens_MMETSP0216_c16332_g1_i1.p2
Waddlia_chondrophila_WP_013182705.1
Acanthamoeba_castellanii_XP_004353733.1
Thaumarchaeota_archaeon_N4_WP_048194473.1
Candidatus_Nitrososphaera_gargensis_WP_015020539.1
Vexillifera_sp_MMETSP0173_c1130_g1_i1.p1
Palpitomonas_bilix_MMETSP0780_c12271_g1_i1.p1
Percolomonas_cosmopolitus_MMETSP0758_c1579_g1_i1.p1
Phaeocystis_globosa_virus_YP_008052403.1
Paulinella_ovalis_nuclear_SingleCell___SAG1_utg7180000021121.g9663.t1
Chrysochromulina_ericina_virus_YP_009173395.1
Caulobacter_phage_Cr30_YP_009098783.1
Bacillus_virus_G_YP_009015423.1
Staphylococcus_virus_42e_YP_239889.1
Naegleria_gruberi_jgi34674
Acidipila_dinghuensis_WP_129209849.1
Occallatibacter_savannae_WP_109489154.1
Burkholderia_sp._DHOD12_WP_137332171.1
NA_WP_054285701.1
Fermentimonas_caenicola_WP_045090640.1
Lascolabacillus_massiliensis_WP_053826985.1
Variibaculum_timonense_WP_080913587.1
Candidatus_Endomicrobium_trichonymphae_WP_096525900.1
bacterium_1xD4267_WP_120396535.1
NA_WP_016291787.1
Thermosyntropha_lipolytica_WP_073089529.1
Dictyoglomus_sp._ob14_WP_148807743.1
Moorella_mulderi_WP_062285500.1
Ardenticatena_maritima_WP_054491546.1
Coprothermobacter_proteolyticus_WP_107763666.1
Candidatus_Chrysopegis_kryptomonas_WP_092347517.1
Aminivibrio_pyruvatiphilus_WP_133957529.1
Cloacibacillus_sp._An23_WP_087363237.1
Hydrogenobacter_thermophilus_WP_012962969.1
Thermocrinis_minervae_WP_079653449.1
Spiroplasma_sabaudiense_WP_025250780.1
Fervidobacterium_thailandense_WP_069293131.1
Thermosipho_globiformans_WP_126993569.1
archaeon_AArcSl_WP_119821314.1
Aciduliprofundum_sp._MAR08339_WP_015283233.1
Cuniculiplasma_divulgatum_WP_077075975.1
Acidilobus_sp._7A_WP_117354628.1
Desulfurococcaceae_archaeon_AG1_WP_109159965.1
Candidatus_Methanodesulfokores_washburnensis_WP_125672077.1
anaerobic_archaeon_MKD1_WP_147664708.1
1.32

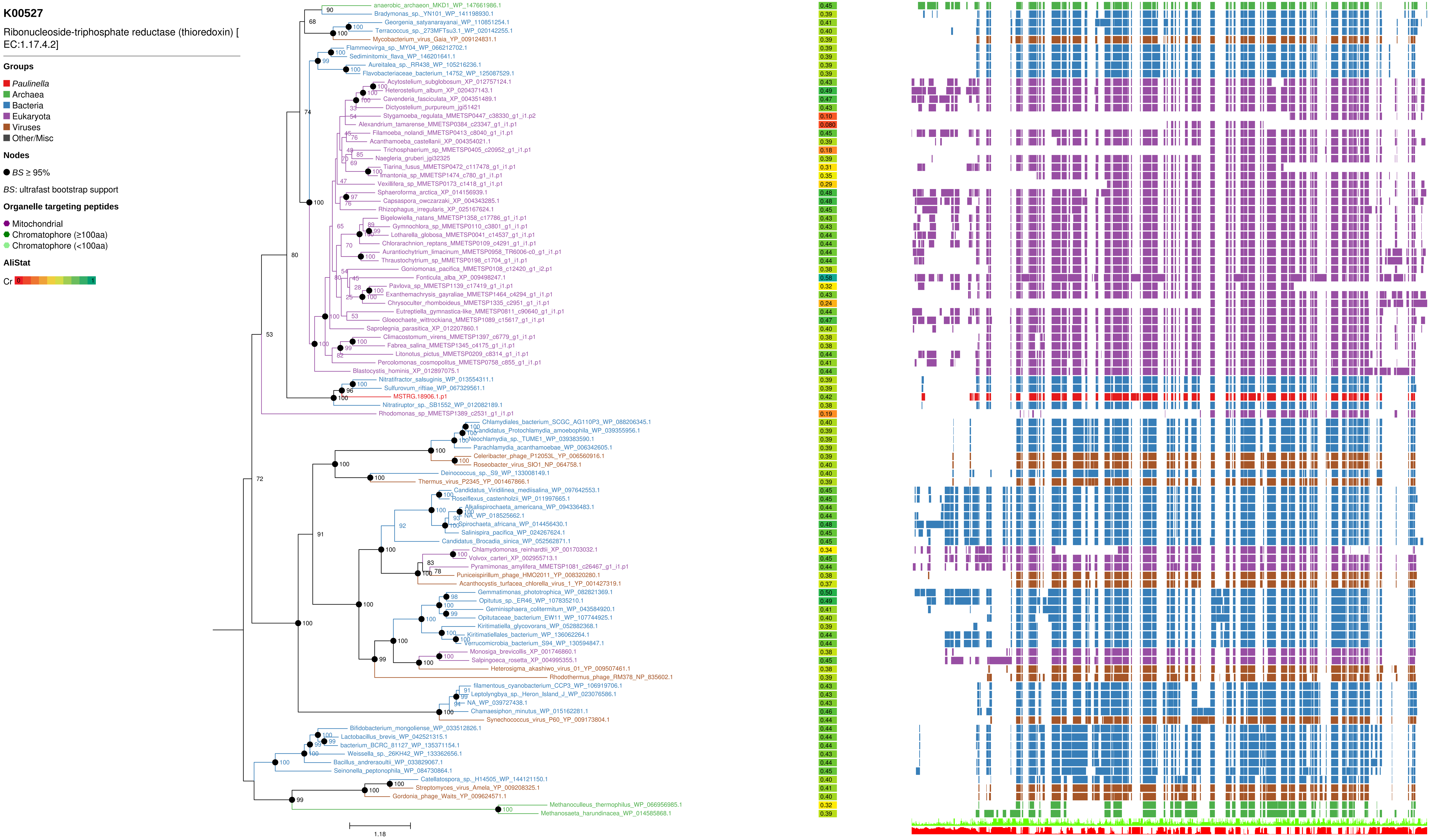
K00527
Ribonucleoside-triphosphate reductase (thioredoxin) [EC:1.17.4.2]
Groups
Paulinella
Archaea
Bacteria
Eukaryota
Viruses
Other/Misc
Nodes
BS ≥ 95%
BS: ultrafast bootstrap support
Organelle targeting peptides
Mitochondrial
Chromatophore (≥100aa)
Chromatophore (<100aa)
AliStat
Cr 0 1
anaerobic_archaeon_MKD1_WP_147661986.1
Bradymonas_sp._YN101_WP_141198930.1
Georgenia_satyanarayanai_WP_110851254.1
Terracoccus_sp._273MFTsu3.1_WP_020142255.1
Mycobacterium_virus_Gaia_YP_009124831.1
Flammeovirga_sp._MY04_WP_066212702.1
Sediminitomix_flava_WP_146201641.1
Aureitalea_sp._RR438_WP_105216236.1
Flavobacteriaceae_bacterium_14752_WP_125087529.1
Acytostelium_subglobosum_XP_012757124.1
Heterostelium_album_XP_020437143.1
Cavenderia_fasciculata_XP_004351489.1
Dictyostelium_purpureum_jgi51421
Stygamoeba_regulata_MMETSP0447_c38330_g1_i1.p2
Alexandrium_tamarense_MMETSP0384_c23347_g1_i1.p1
Filamoeba_nolandi_MMETSP0413_c8040_g1_i1.p1
Acanthamoeba_castellanii_XP_004354021.1
Trichosphaerium_sp_MMETSP0405_c20952_g1_i1.p1
Naegleria_gruberi_jgi32325
Tiarina_fusus_MMETSP0472_c117478_g1_i1.p1
Imantonia_sp_MMETSP1474_c780_g1_i1.p1
Vexillifera_sp_MMETSP0173_c1418_g1_i1.p1
Sphaeroforma_arctica_XP_014156939.1
Capsaspora_owczarzaki_XP_004343285.1
Rhizophagus_irregularis_XP_025167624.1
Bigelowiella_natans_MMETSP1358_c17786_g1_i1.p1
Gymnochlora_sp_MMETSP0110_c3801_g1_i1.p1
Lotharella_globosa_MMETSP0041_c14537_g1_i1.p1
Chlorarachnion_reptans_MMETSP0109_c4291_g1_i1.p1
Aurantiochytrium_limacinum_MMETSP0958_TR6006-c0_g1_i1.p1
Thraustochytrium_sp_MMETSP0198_c1704_g1_i1.p1
Goniomonas_pacifica_MMETSP0108_c12420_g1_i2.p1
Fonticula_alba_XP_009498247.1
Pavlova_sp_MMETSP1139_c17419_g1_i1.p1
Exanthemachrysis_gayraliae_MMETSP1464_c4294_g1_i1.p1
Chrysoculter_rhomboideus_MMETSP1335_c2951_g1_i1.p1
Eutreptiella_gymnastica-like_MMETSP0811_c90640_g1_i1.p1
Gloeochaete_wittrockiana_MMETSP1089_c15617_g1_i1.p1
Saprolegnia_parasitica_XP_012207860.1
Climacostomum_virens_MMETSP1397_c6779_g1_i1.p1
Fabrea_salina_MMETSP1345_c4175_g1_i1.p1
Litonotus_pictus_MMETSP0209_c8314_g1_i1.p1
Percolomonas_cosmopolitus_MMETSP0758_c855_g1_i1.p1
Blastocystis_hominis_XP_012897075.1
Nitratifractor_salsuginis_WP_013554311.1
Sulfurovum_riftiae_WP_067329561.1
MSTRG.18906.1.p1
Nitratiruptor_sp._SB1552_WP_012082189.1
Rhodomonas_sp_MMETSP1389_c2531_g1_i1.p1
Chlamydiales_bacterium_SCGC_AG110P3_WP_088206345.1
Candidatus_Protochlamydia_amoebophila_WP_039355956.1
Neochlamydia_sp._TUME1_WP_039383590.1
Parachlamydia_acanthamoebae_WP_006342605.1
Celeribacter_phage_P12053L_YP_006560916.1
Roseobacter_virus_SIO1_NP_064758.1
Deinococcus_sp._S9_WP_133008149.1
Thermus_virus_P2345_YP_001467866.1
Candidatus_Viridilinea_mediisalina_WP_097642553.1
Roseiflexus_castenholzii_WP_011997665.1
Alkalispirochaeta_americana_WP_094336483.1
NA_WP_018525662.1
Spirochaeta_africana_WP_014456430.1
Salinispira_pacifica_WP_024267624.1
Candidatus_Brocadia_sinica_WP_052562871.1
Chlamydomonas_reinhardtii_XP_001703032.1
Volvox_carteri_XP_002955713.1
Pyramimonas_amylifera_MMETSP1081_c26467_g1_i1.p1
Puniceispirillum_phage_HMO2011_YP_008320280.1
Acanthocystis_turfacea_chlorella_virus_1_YP_001427319.1
Gemmatimonas_phototrophica_WP_082821369.1
Opitutus_sp._ER46_WP_107835210.1
Geminisphaera_colitermitum_WP_043584920.1
Opitutaceae_bacterium_EW11_WP_107744925.1
Kiritimatiella_glycovorans_WP_052882368.1
Kiritimatiellales_bacterium_WP_136062264.1
Verrucomicrobia_bacterium_S94_WP_130594847.1
Monosiga_brevicollis_XP_001746860.1
Salpingoeca_rosetta_XP_004995355.1
Heterosigma_akashiwo_virus_01_YP_009507461.1
Rhodothermus_phage_RM378_NP_835602.1
filamentous_cyanobacterium_CCP3_WP_106919706.1
Leptolyngbya_sp._Heron_Island_J_WP_023076586.1
NA_WP_039727438.1
Chamaesiphon_minutus_WP_015162281.1
Synechococcus_virus_P60_YP_009173804.1
Bifidobacterium_mongoliense_WP_033512826.1
Lactobacillus_brevis_WP_042521315.1
bacterium_BCRC_81127_WP_135371154.1
Weissella_sp._26KH42_WP_133262656.1
Bacillus_andreraoultii_WP_033829067.1
Seinonella_peptonophila_WP_084730864.1
Catellatospora_sp._H14505_WP_144121150.1
Streptomyces_virus_Amela_YP_009208325.1
Gordonia_phage_Waits_YP_009624571.1
Methanoculleus_thermophilus_WP_066956985.1
Methanosaeta_harundinacea_WP_014585868.1
1.18

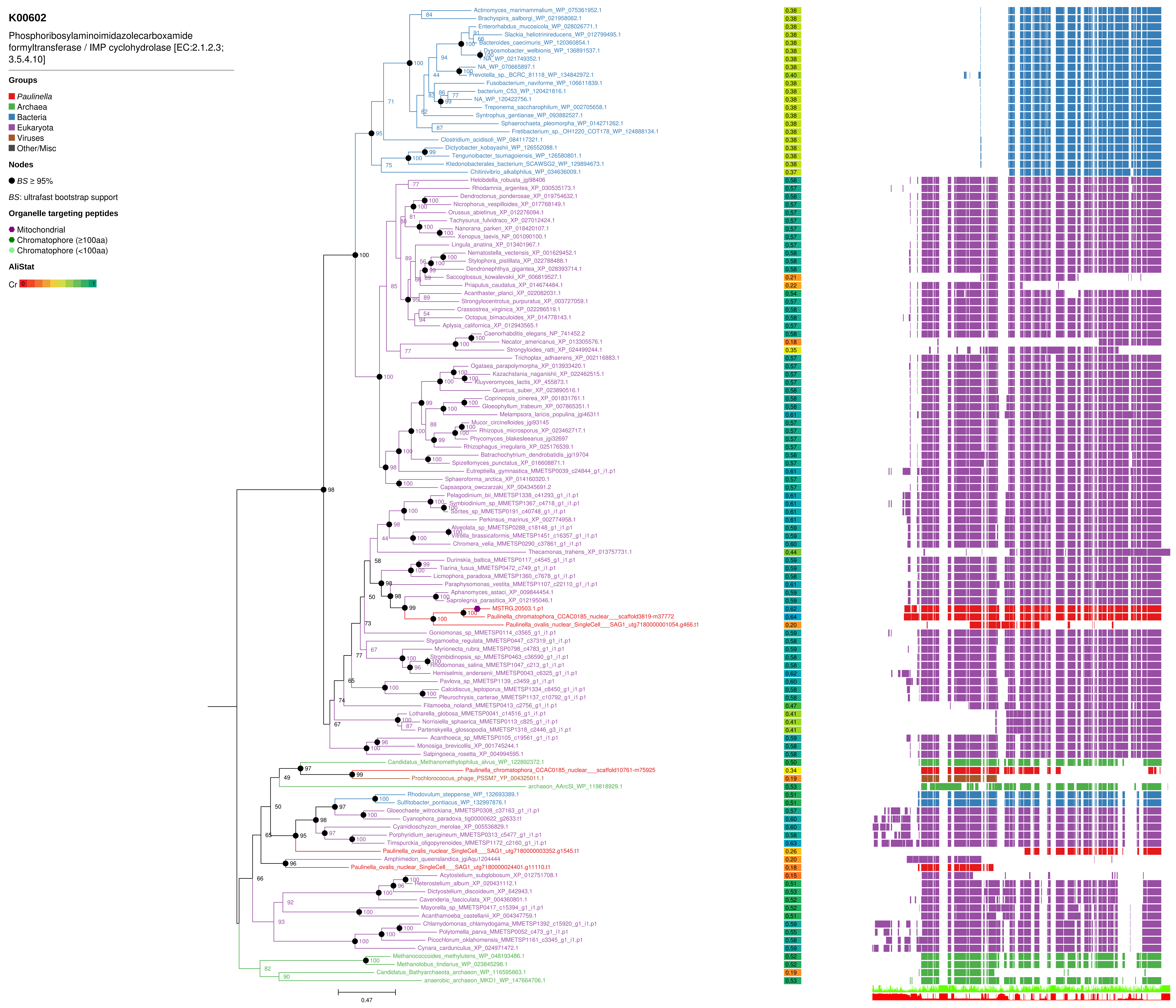
K00602
Phosphoribosylaminoimidazolecarboxamide formyltransferase / IMP cyclohydrolase [EC:2.1.2.3; 3.5.4.10]
Groups
Paulinella
Archaea
Bacteria
Eukaryota
Viruses
Other/Misc
Nodes
BS ≥ 95%
BS: ultrafast bootstrap support
Organelle targeting peptides
Mitochondrial
Chromatophore (≥100aa)
Chromatophore (<100aa)
AliStat
Cr 0 1
Actinomyces_marimammalium_WP_075361952.1
Brachyspira_aalborgi_WP_021958062.1
Enterorhabdus_mucosicola_WP_028026771.1
Slackia_heliotrinireducens_WP_012799495.1
Bacteroides_caecimuris_WP_120360854.1
Dysosmobacter_welbionis_WP_136891537.1
NA_WP_021749352.1
NA_WP_070665897.1
Prevotella_sp._BCRC_81118_WP_134842972.1
Fusobacterium_naviforme_WP_106611839.1
bacterium_C53_WP_120421816.1
NA_WP_120422756.1
Treponema_saccharophilum_WP_002705658.1
Syntrophus_gentianae_WP_093882527.1
Sphaerochaeta_pleomorpha_WP_014271262.1
Fretibacterium_sp._OH1220_COT178_WP_124888134.1
Clostridium_acidisoli_WP_084117321.1
Dictyobacter_kobayashii_WP_126552088.1
Tengunoibacter_tsumagoiensis_WP_126580801.1
Ktedonobacterales_bacterium_SCAWSG2_WP_129894673.1
Chitinivibrio_alkaliphilus_WP_034636009.1
Helobdella_robusta_jgi98406
Rhodamnia_argentea_XP_030535173.1
Dendroctonus_ponderosae_XP_019754632.1
Nicrophorus_vespilloides_XP_017768149.1
Orussus_abietinus_XP_012276094.1
Tachysurus_fulvidraco_XP_027012424.1
Nanorana_parkeri_XP_018420107.1
Xenopus_laevis_NP_001090100.1
Lingula_anatina_XP_013401967.1
Nematostella_vectensis_XP_001629452.1
Stylophora_pistillata_XP_022788488.1
Dendronephthya_gigantea_XP_028393714.1
Saccoglossus_kowalevskii_XP_006819527.1
Priapulus_caudatus_XP_014674484.1
Acanthaster_planci_XP_022082031.1
Strongylocentrotus_purpuratus_XP_003727059.1
Crassostrea_virginica_XP_022286519.1
Octopus_bimaculoides_XP_014778143.1
Aplysia_californica_XP_012943565.1
Caenorhabditis_elegans_NP_741452.2
Necator_americanus_XP_013305576.1
Strongyloides_ratti_XP_024499244.1
Trichoplax_adhaerens_XP_002116883.1
Ogataea_parapolymorpha_XP_013933420.1
Kazachstania_naganishii_XP_022462515.1
Kluyveromyces_lactis_XP_455873.1
Quercus_suber_XP_023890516.1
Coprinopsis_cinerea_XP_001831761.1
Gloeophyllum_trabeum_XP_007865351.1
Melampsora_laricis_populina_jgi46311
Mucor_circinelloides_jgi93145
Rhizopus_microsporus_XP_023462717.1
Phycomyces_blakesleeanus_jgi32697
Rhizophagus_irregularis_XP_025176539.1
Batrachochytrium_dendrobatidis_jgi19704
Spizellomyces_punctatus_XP_016608871.1
Eutreptiella_gymnastica_MMETSP0039_c24844_g1_i1.p1
Sphaeroforma_arctica_XP_014160320.1
Capsaspora_owczarzaki_XP_004345691.2
Pelagodinium_bii_MMETSP1338_c41293_g1_i1.p1
Symbiodinium_sp_MMETSP1367_c4718_g1_i1.p1
Sorites_sp_MMETSP0191_c40748_g1_i1.p1
Perkinsus_marinus_XP_002774958.1
Alveolata_sp_MMETSP0288_c18148_g1_i1.p1
Vitrella_brassicaformis_MMETSP1451_c16357_g1_i1.p1
Chromera_velia_MMETSP0290_c37861_g1_i1.p1
Thecamonas_trahens_XP_013757731.1
Durinskia_baltica_MMETSP0117_c4545_g1_i1.p1
Tiarina_fusus_MMETSP0472_c749_g1_i1.p1
Licmophora_paradoxa_MMETSP1360_c7678_g1_i1.p1
Paraphysomonas_vestita_MMETSP1107_c22110_g1_i1.p1
Aphanomyces_astaci_XP_009844454.1
Saprolegnia_parasitica_XP_012195046.1
MSTRG.20503.1.p1
Paulinella_chromatophora_CCAC0185_nuclear___scaffold3819-m37772
Paulinella_ovalis_nuclear_SingleCell___SAG1_utg7180000001054.g466.t1
Goniomonas_sp_MMETSP0114_c3565_g1_i1.p1
Stygamoeba_regulata_MMETSP0447_c37319_g1_i1.p1
Myrionecta_rubra_MMETSP0798_c4783_g1_i1.p1
Strombidinopsis_sp_MMETSP0463_c36590_g1_i1.p1
Rhodomonas_salina_MMETSP1047_c213_g1_i1.p1
Hemiselmis_andersenii_MMETSP0043_c6325_g1_i1.p1
Pavlova_sp_MMETSP1139_c3459_g1_i1.p1
Calcidiscus_leptoporus_MMETSP1334_c8450_g1_i1.p1
Pleurochrysis_carterae_MMETSP1137_c10792_g1_i1.p1
Filamoeba_nolandi_MMETSP0413_c2756_g1_i1.p1
Lotharella_globosa_MMETSP0041_c14516_g1_i1.p1
Norrisiella_sphaerica_MMETSP0113_c825_g1_i1.p1
Partenskyella_glossopodia_MMETSP1318_c2446_g3_i1.p1
Acanthoeca_sp_MMETSP0105_c19561_g1_i1.p1
Monosiga_brevicollis_XP_001745244.1
Salpingoeca_rosetta_XP_004994595.1
Candidatus_Methanomethylophilus_alvus_WP_122892372.1
Paulinella_chromatophora_CCAC0185_nuclear___scaffold10761-m75925
Prochlorococcus_phage_PSSM7_YP_004325011.1
archaeon_AArcSl_WP_119818929.1
Rhodovulum_steppense_WP_132693389.1
Sulfitobacter_pontiacus_WP_132997876.1
Gloeochaete_wittrockiana_MMETSP0308_c37163_g1_i1.p1
Cyanophora_paradoxa_tig00000622_g2633.t1
Cyanidioschyzon_merolae_XP_005536829.1
Porphyridium_aerugineum_MMETSP0313_c5477_g1_i1.p1
Timspurckia_oligopyrenoides_MMETSP1172_c2160_g1_i1.p1
Paulinella_ovalis_nuclear_SingleCell___SAG1_utg7180000003352.g1545.t1
Amphimedon_queenslandica_jgiAqu1204444
Paulinella_ovalis_nuclear_SingleCell___SAG1_utg7180000024401.g11110.t1
Acytostelium_subglobosum_XP_012751708.1
Heterostelium_album_XP_020431112.1
Dictyostelium_discoideum_XP_642943.1
Cavenderia_fasciculata_XP_004360801.1
Mayorella_sp_MMETSP0417_c15394_g1_i1.p1
Acanthamoeba_castellanii_XP_004347759.1
Chlamydomonas_chlamydogama_MMETSP1392_c15920_g1_i1.p1
Polytomella_parva_MMETSP0052_c473_g1_i1.p1
Picochlorum_oklahomensis_MMETSP1161_c3345_g1_i1.p1
Cynara_cardunculus_XP_024971472.1
Methanococcoides_methylutens_WP_048193486.1
Methanolobus_tindarius_WP_023845298.1
Candidatus_Bathyarchaeota_archaeon_WP_116595863.1
anaerobic_archaeon_MKD1_WP_147664706.1
0.47

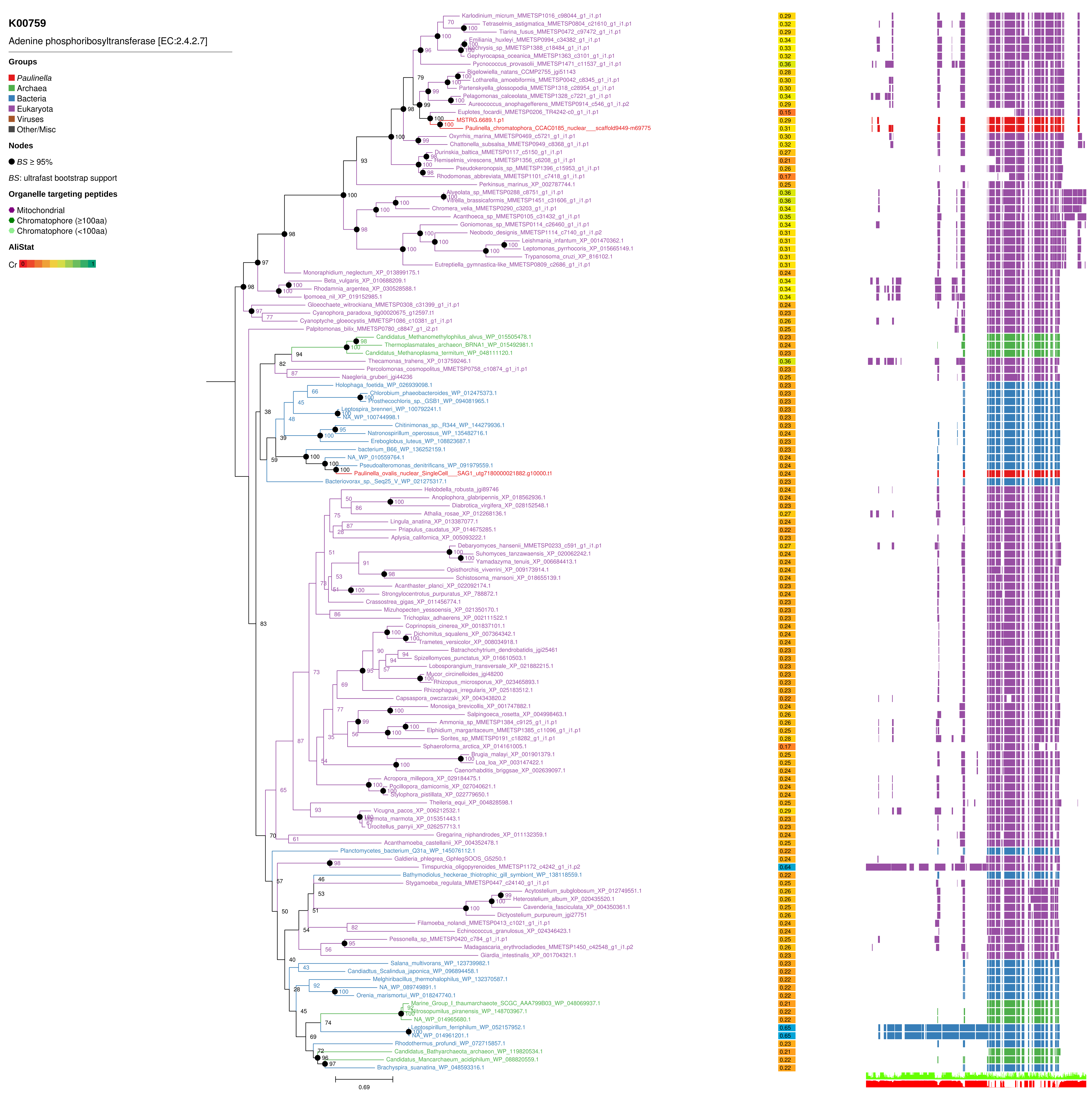

K00759
Adenine phosphoribosyltransferase [EC:2.4.2.7]
Groups
Paulinella
Archaea
Bacteria
Eukaryota
Viruses
Other/Misc
Nodes
BS ≥ 95%
BS: ultrafast bootstrap support
Organelle targeting peptides
Mitochondrial
Chromatophore (≥100aa)
Chromatophore (<100aa)
AliStat
Cr
Karlodinium_micrum_MMETSP1016_c98044_g1_i1.p1
Tetraselmis_astigmatica_MMETSP0804_c21610_g1_i1.p1
Tiarina_fusus_MMETSP0472_c97472_g1_i1.p1
Emiliania_huxleyi_MMETSP0994_c34382_g1_i1.p1
Gephyrocapsa_oceanica_MMETSP1363_c3101_g1_i1.p1
Pycnococcus_provasolii_MMETSP1471_c11537_g1_i1.p1
Bigelowiella_natans_CCMP2755_jgi51143
Lotharella_amoebiformis_MMETSP0042_c8345_g1_i1.p1
Partenskyella_glossopodia_MMETSP1318_c28954_g1_i1.p1
Pelagomonas_calceolata_MMETSP1328_c7221_g1_i1.p1
Aureococcus_anophagefferens_MMETSP0914_c546_g1_i1.p2
Euplotes_focardii_MMETSP0206_TR4242-c0_g1_i1.p1
MSTRG.6689.1.p1
Paulinella_chromatophora_CCAC0185_nuclear___scaffold9449-m69775
Oxyrrhis_marina_MMETSP0469_c5721_g1_i1.p1
Chattonella_subsalsa_MMETSP0949_c8368_g1_i1.p1
Durinskia_baltica_MMETSP0117_c5150_g1_i1.p1
Hemiselmis_virescens_MMETSP1356_c6208_g1_i1.p1
Pseudokeronopsis_sp_MMETSP1396_c15953_g1_i1.p1
Rhodomonas_abbreviata_MMETSP1101_c7418_g1_i1.p1
Perkinsus_marinus_XP_002787744.1
Alveolata_sp_MMETSP0288_c8751_g1_i1.p1
Vitrella_brassicaformis_MMETSP1451_c31606_g1_i1.p1
Chromera_velia_MMETSP0290_c3203_g1_i1.p1
Acanthoeca_sp_MMETSP0105_c31432_g1_i1.p1
Goniomonas_sp_MMETSP0114_c26460_g1_i1.p1
Neobodo_designis_MMETSP1114_c7140_g1_i1.p2
Leishmania_infantum_XP_001470362.1
Leptomonas_pyrrhocoris_XP_015665149.1
Trypanosoma_cruzi_XP_816102.1
Eutreptiella_gymnastica-like_MMETSP0809_c2686_g1_i1.p1
Monoraphidium_neglectum_XP_013899175.1
Beta_vulgaris_XP_010688209.1
Rhodamnia_argentea_XP_030528588.1
Ipomoea_nil_XP_019152985.1
Gloeochaete_witrockiana_MMETSP0308_c31399_g1_i1.p1
Cyanophora_paradoxa_tig00020675_g12597.t1
Cyanoptyche_gloeocystis_MMETSP1086_c10381_g1_i1.p1
Palpitomonas_bilix_MMETSP0780_c8847_g1_i2.p1
Candidatus_Methanomethylophilus_alvus_WP_015505478.1
Thermoplasmatales_archaeon_BRNA1_WP_015492981.1
Candidatus_Methanoplasma_termitum_WP_048111120.1
Thecamonas_trahens_XP_013759246.1
Percolomonas_cosmopolitus_MMETSP0758_c10874_g1_i1.p1
Naegleria_gruberi_jgi44236
Holophaga_foetida_WP_026939098.1
Chlorobium_phaeobacteroides_WP_012475373.1
Prosthecochloris_sp._GSB1_WP_094081965.1
Leptospira_brenneri_WP_100792241.1
NA_WP_100744998.1
Chitinimonas_sp._R344_WP_144279936.1
Natronospirillum_operosus_WP_135482716.1
Ereboglobus_luteus_WP_108823687.1
bacterium_B66_WP_136252159.1
NA_WP_010559764.1
Pseudoalteromonas_denitrificans_WP_091979559.1
Paulinella_ovalis_nuclear_SingleCell___SAG1_utg7180000021882.g10000.t1
Bacteriovorax_sp._Seq25_V_WP_021275317.1
Helobdella_robusta_jgi89746
Anoplophora_glabripennis_XP_018562936.1
Diabrotica_virgifera_XP_028152548.1
Athalia_rosae_XP_012268136.1
Lingula_anatina_XP_013387077.1
Priapulus_caudatus_XP_014675285.1
Aplysia_californica_XP_005093222.1
Debaryomyces_hansenii_MMETSP0233_c591_g1_i1.p1
Suhomyces_tanzawaensis_XP_020062242.1
Yamadazyma_tenuis_XP_006684413.1
Opisthorchis_viverrini_XP_009173914.1
Schistosoma_mansoni_XP_018655139.1
Acanthaster_planci_XP_022092174.1
Strongylocentrotus_purpuratus_XP_788872.1
Crassostrea_gigas_XP_011456774.1
Mizuhopecten_yessoensis_XP_021350170.1
Trichoplax_adhaerens_XP_002111522.1
Coprinopsis_cinerea_XP_001837101.1
Dichomitus_squalens_XP_007364342.1
Trametes_versicolor_XP_008034918.1
Batrachochytrium_dendrobatidis_jgi25461
Spizellomyces_punctatus_XP_016610503.1
Lobosporangium_transversale_XP_021882215.1
Mucor_circinelloides_jgi48200
Rhizopus_microsporus_XP_023465893.1
Rhizophagus_irregularis_XP_025183512.1
Capsaspora_owczarzaki_XP_004343820.2
Monosiga_brevicollis_XP_001747882.1
Salpingoeca_rosetta_XP_004998463.1
Ammonia_sp_MMETSP1384_c9125_g1_i1.p1
Elphidium_margaritaceum_MMETSP1385_c11096_g1_i1.p1
Sorites_sp_MMETSP0191_c18282_g1_i1.p1
Sphaeroforma_arctica_XP_014161005.1
Brugia_malayi_XP_001901379.1
Loa_loa_XP_003147422.1
Caenorhabditis_briggsae_XP_002639097.1
Acropora_millepora_XP_029184475.1
Pocillopora_damicornis_XP_027040621.1
Stylophora_pistillata_XP_022779650.1
Theileria_equi_XP_004828598.1
Vicugna_pacos_XP_006212532.1
Marmota_marmota_XP_015351443.1
Urocitellus_parryii_XP_026257713.1
Gregarina_niphandrodes_XP_011132359.1
Acanthamoeba_castellanii_XP_004352478.1
Planctomycetes_bacterium_Q31a_WP_145076112.1
Galdieria_phlegrea_GphlegSOOS_G5250.1
Timspurckia_oligopyrenoides_MMETSP1172_c4242_g1_i1.p2
Bathymodiolus_heckerae_thiotrophic_gill_symbiont_WP_138118559.1
Stygamoeba_regulata_MMETSP0447_c24140_g1_i1.p1
Acytostelium_subglobosum_XP_012749551.1
Heterostelium_album_XP_020435520.1
Cavenderia_fasciculata_XP_004350361.1
Dictyostelium_purpureum_jgi27751
Filamoeba_nolandi_MMETSP0413_c1021_g1_i1.p1
Echinococcus_granulosus_XP_024346423.1
Pessonella_sp_MMETSP0420_c784_g1_i1.p1
Madagascaria_erythrocladiodes_MMETSP1450_c42548_g1_i1.p2
Giardia_intestinalis_XP_001704321.1
Salana_multivorans_WP_123739982.1
Candiadtus_Scalindua_japonica_WP_096894458.1
Melghiribacillus_thermohalophilus_WP_132370587.1
NA_WP_089749891.1
Orenia_marismortui_WP_018247740.1
Marine_Group_I_thaumarchaeote_SCGC_AAA799B03_WP_048069937.1
Nitrosopumilus_piranensis_WP_148703967.1
NA_WP_014965680.1
Leptospirillum_ferriphilum_WP_052157952.1
NA_WP_014961201.1
Rhodothermus_profundi_WP_072715857.1
Candidatus_Bathyarchaeota_archaeon_WP_119820534.1
Candidatus_Mancarchaeum_acidiphilum_WP_088820559.1
Brachyspira_suanatina_WP_048593316.1
0.69

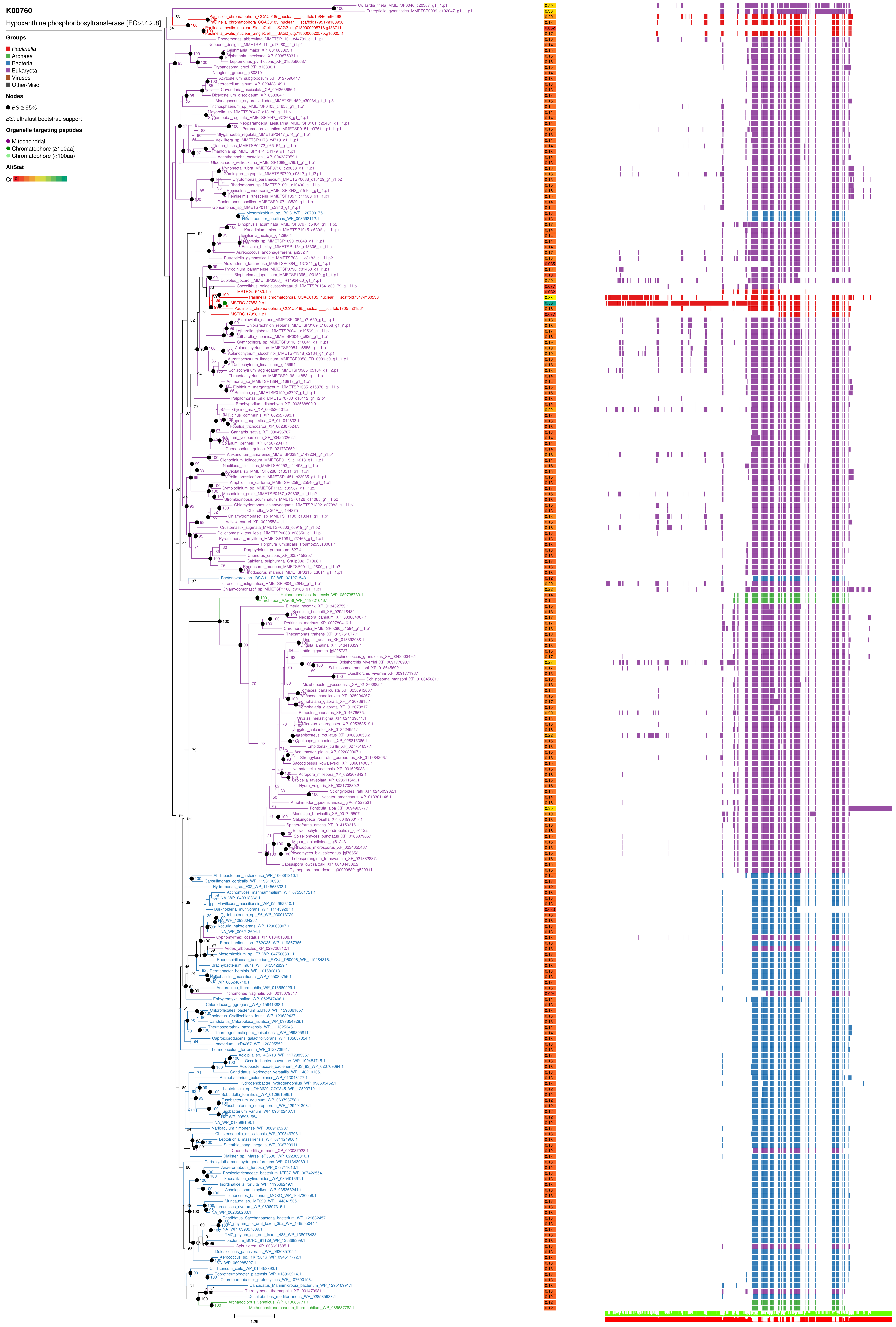

K00760
Hypoxanthine phosphoribosyltransferase [EC:2.4.2.8]
Groups
Paulinella
Archaea
Bacteria
Eukaryota
Viruses
Other/Misc
Nodes
BS ≥ 95%
BS: ultrafast bootstrap support
Organelle targeting peptides
Mitochondrial
Chromatophore (≥100aa)
Chromatophore (<100aa)
AliStat
Cr 0 1
1.29

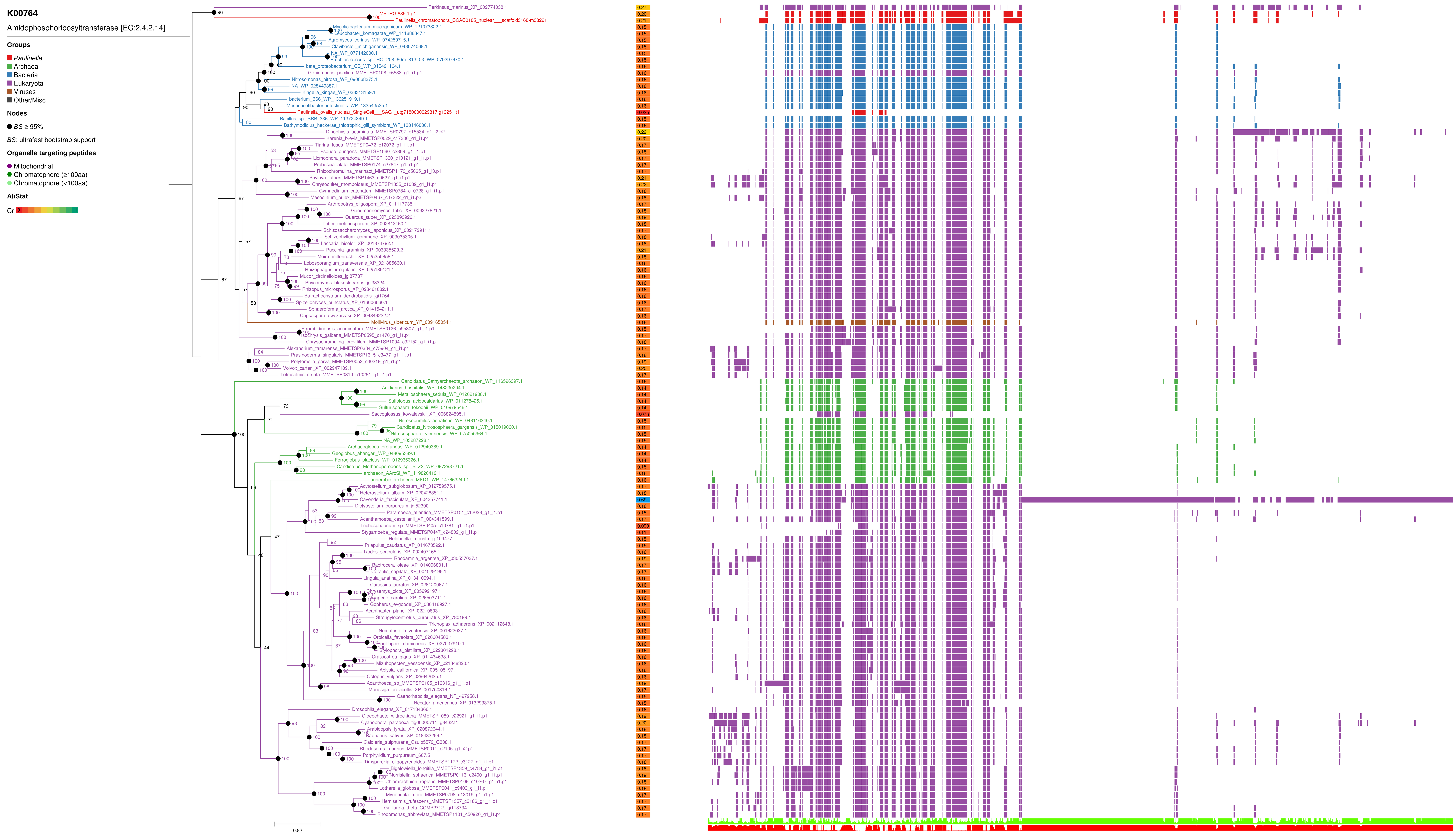
K00764
Amidophosphoribosyltransferase [EC:2.4.2.14]
Groups
Paulinella
Archaea
Bacteria
Eukaryota
Viruses
Other/Misc
Nodes
BS ≥ 95%
BS: ultrafast bootstrap support
Organelle targeting peptides
Mitochondrial
Chromatophore (≥100aa)
Chromatophore (<100aa)
AliStat
Cr
0.82

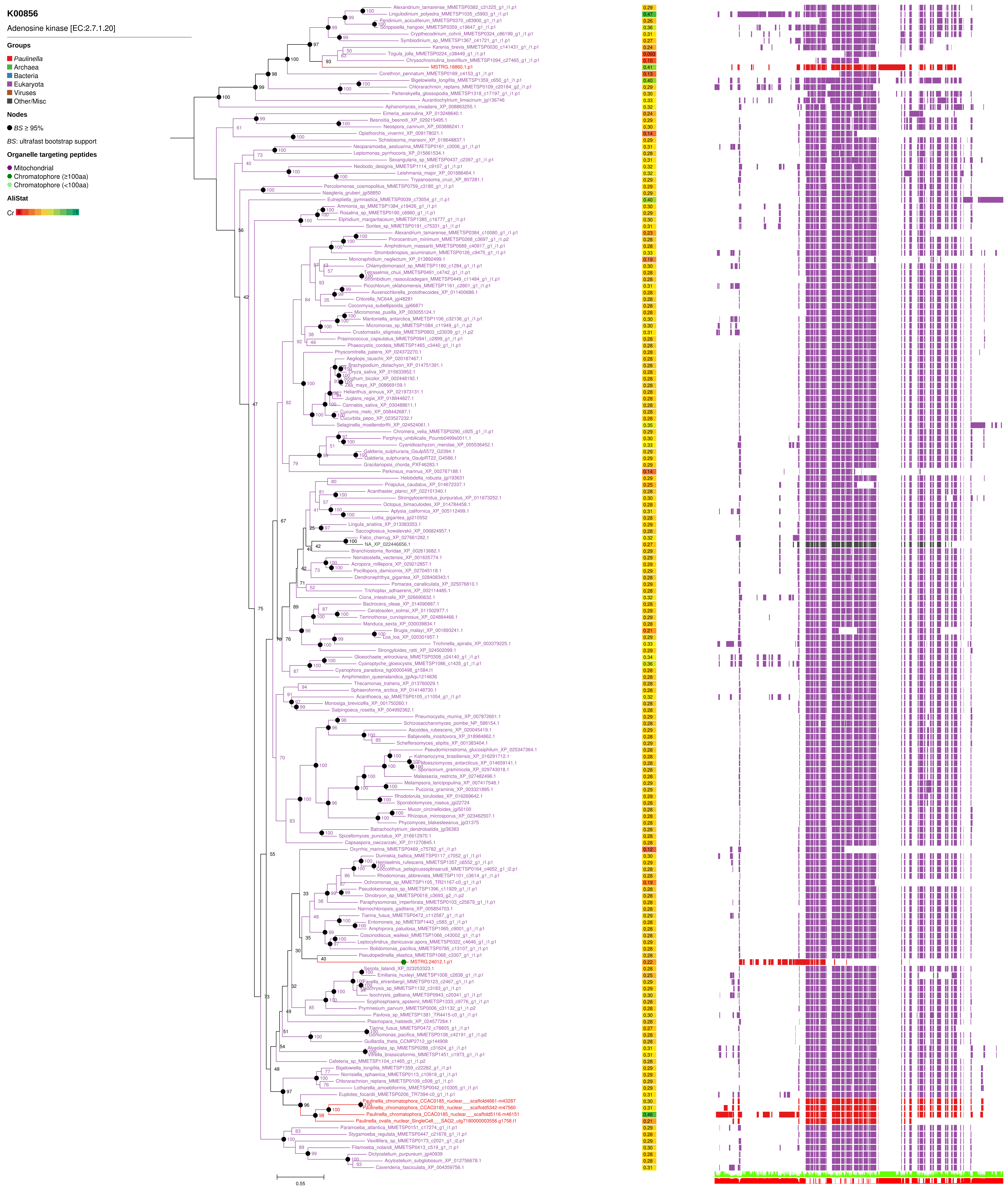
K00856
Adenosine kinase [EC:2.7.1.20]
Groups
Paulinella
Archaea
Bacteria
Eukaryota
Viruses
Other/Misc
Nodes
BS ≥ 95%
BS: ultrafast bootstrap support
Organelle targeting peptides
Mitochondrial
Chromatophore (≥100aa)
Chromatophore (<100aa)
AliStat
Cr
0
1
0.55

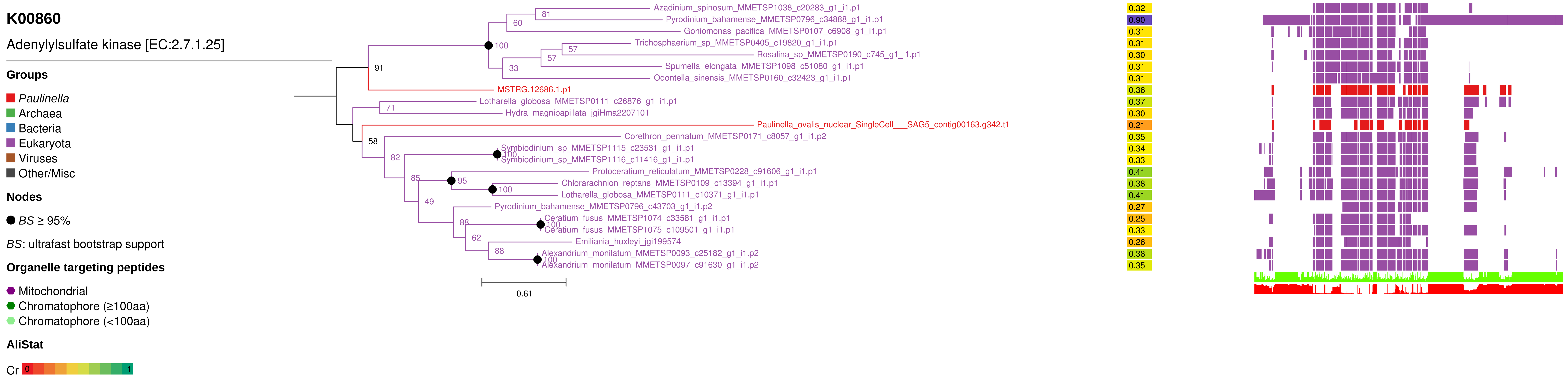

K00860
Adenylylsulfate kinase [EC:2.7.1.25]
Groups
Paulinella
Archaea
Bacteria
Eukaryota
Viruses
Other/Misc
Nodes
BS ≥ 95%
BS: ultrafast bootstrap support
Organelle targeting peptides
Mitochondrial
Chromatophore (≥100aa)
Chromatophore (<100aa)
AliStat
Cr 0 1
Azadinium_spinosum_MMETSP1038_c20283_g1_i1.p1
Pyrodinium_bahamense_MMETSP0796_c34888_g1_i1.p1
Goniomonas_pacifica_MMETSP0107_c6908_g1_i1.p1
Trichosphaerium_sp_MMETSP0405_c19820_g1_i1.p1
Rosalina_sp_MMETSP0190_c745_g1_i1.p1
Spumella_elongata_MMETSP1098_c51080_g1_i1.p1
Odontella_sinensis_MMETSP0160_c32423_g1_i1.p1
MSTRG.12686.1.p1
Lotharella_globosa_MMETSP0111_c26876_g1_i1.p1
Hydra_magnipapillata_jgiHma2207101
Paulinella_ovalis_nuclear_SingleCell___SAG5_contig00163.g342.t1
Corethron_pennatum_MMETSP0171_c8057_g1_i1.p2
Symbiodinium_sp_MMETSP1115_c23531_g1_i1.p1
Symbiodinium_sp_MMETSP1116_c11416_g1_i1.p1
Protoceratium_reticulatum_MMETSP0228_c91606_g1_i1.p1
Chlorarachnion_reptans_MMETSP0109_c13394_g1_i1.p1
Lotharella_globosa_MMETSP0111_c10371_g1_i1.p1
Pyrodinium_bahamense_MMETSP0796_c43703_g1_i1.p2
Ceratium_fusus_MMETSP1074_c33581_g1_i1.p1
Ceratium_fusus_MMETSP1075_c109501_g1_i1.p1
Emiliania_huxleyi_jgi199574
Alexandrium_monilatum_MMETSP0093_c25182_g1_i1.p2
Alexandrium_monilatum_MMETSP0097_c91630_g1_i1.p2
91
81
60
100
57
57
33
71
58
100
82
85
95
100
49
88
100
62
88
100
0.61
0.32
0.90
0.31
0.31
0.30
0.31
0.31
0.36
0.37
0.30
0.21
0.35
0.34
0.33
0.41
0.38
0.41
0.27
0.25
0.33
0.26
0.38
0.35

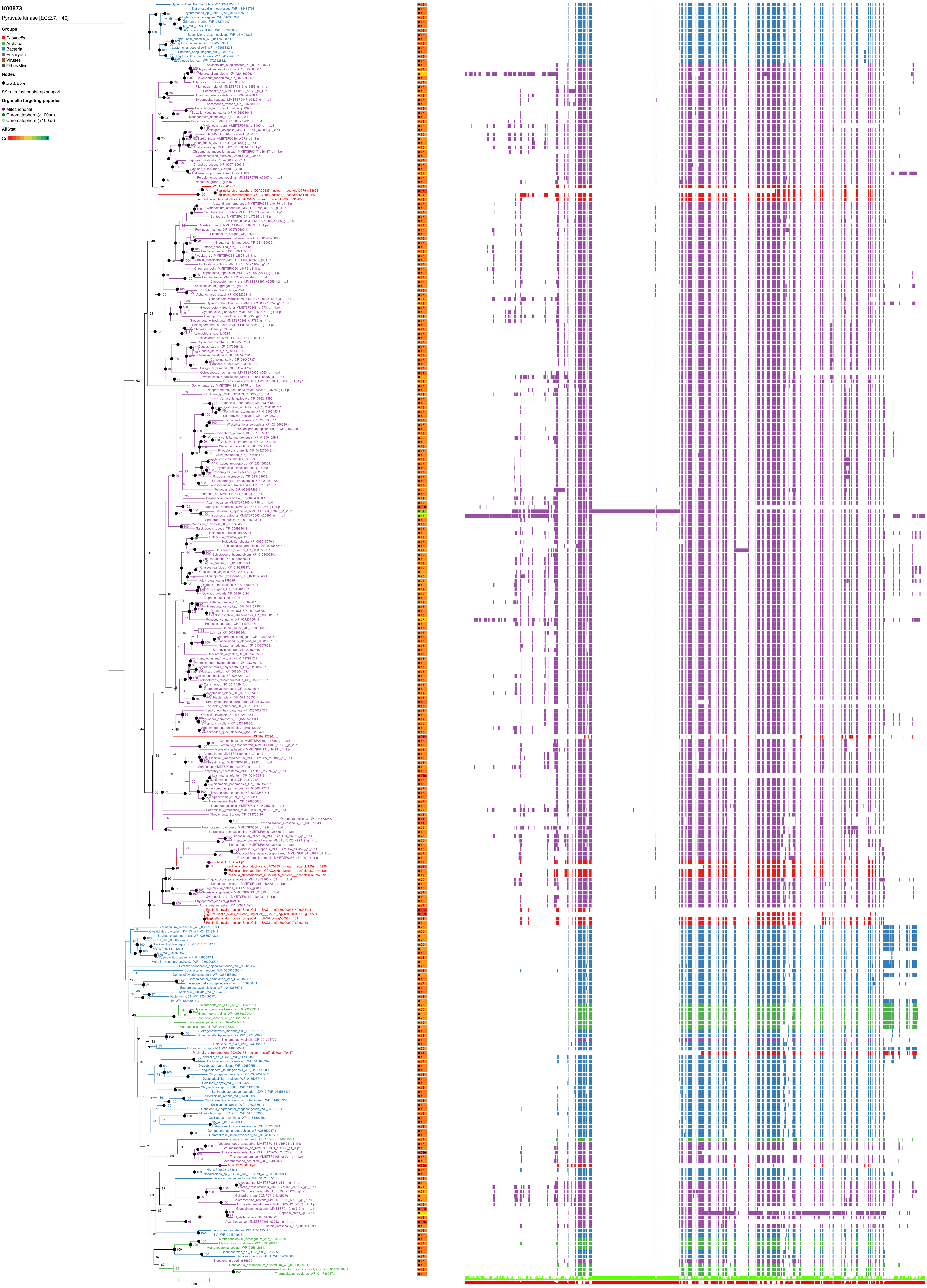

K00873
Pyruvate kinase [EC:2.7.1.40]
Groups
Paulinella
Archaea
Bacteria
Eukaryota
Viruses
Other/Misc
Nodes
BS ≥ 95%
BS: ultrafast bootstrap support
Organelle targeting peptides
Mitochondrial
Chromatophore (≥100aa)
Chromatophore (<100aa)
AliStat
Cr
0.88

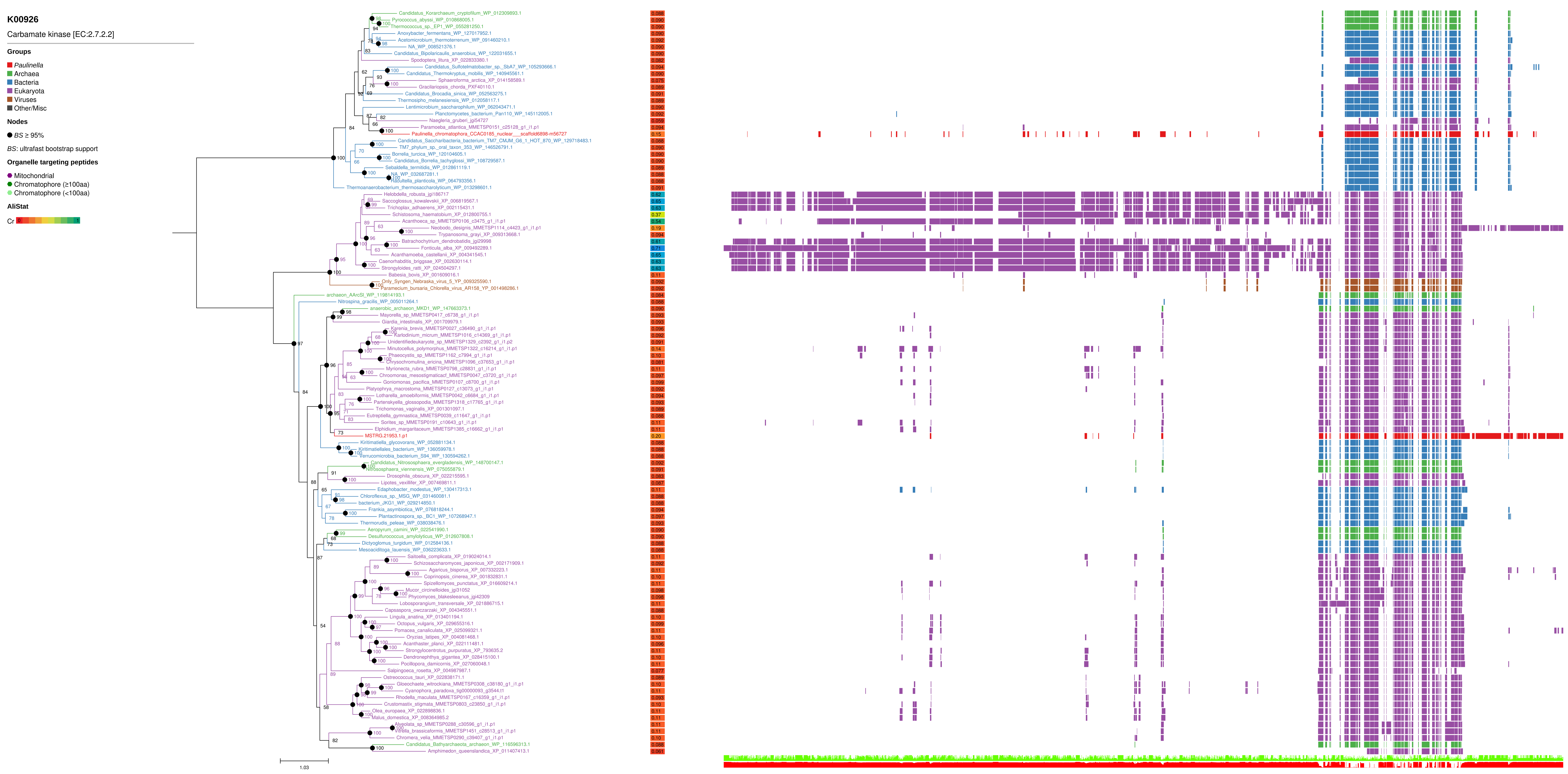
K00926
Carbamate kinase [EC:2.7.2.2]
Groups
Paulinella
Archaea
Bacteria
Eukaryota
Viruses
Other/Misc
Nodes
BS ≥ 95%
BS: ultrafast bootstrap support
Organelle targeting peptides
Mitochondrial
Chromatophore (≥100aa)
Chromatophore (<100aa)
AliStat
Cr
1.03

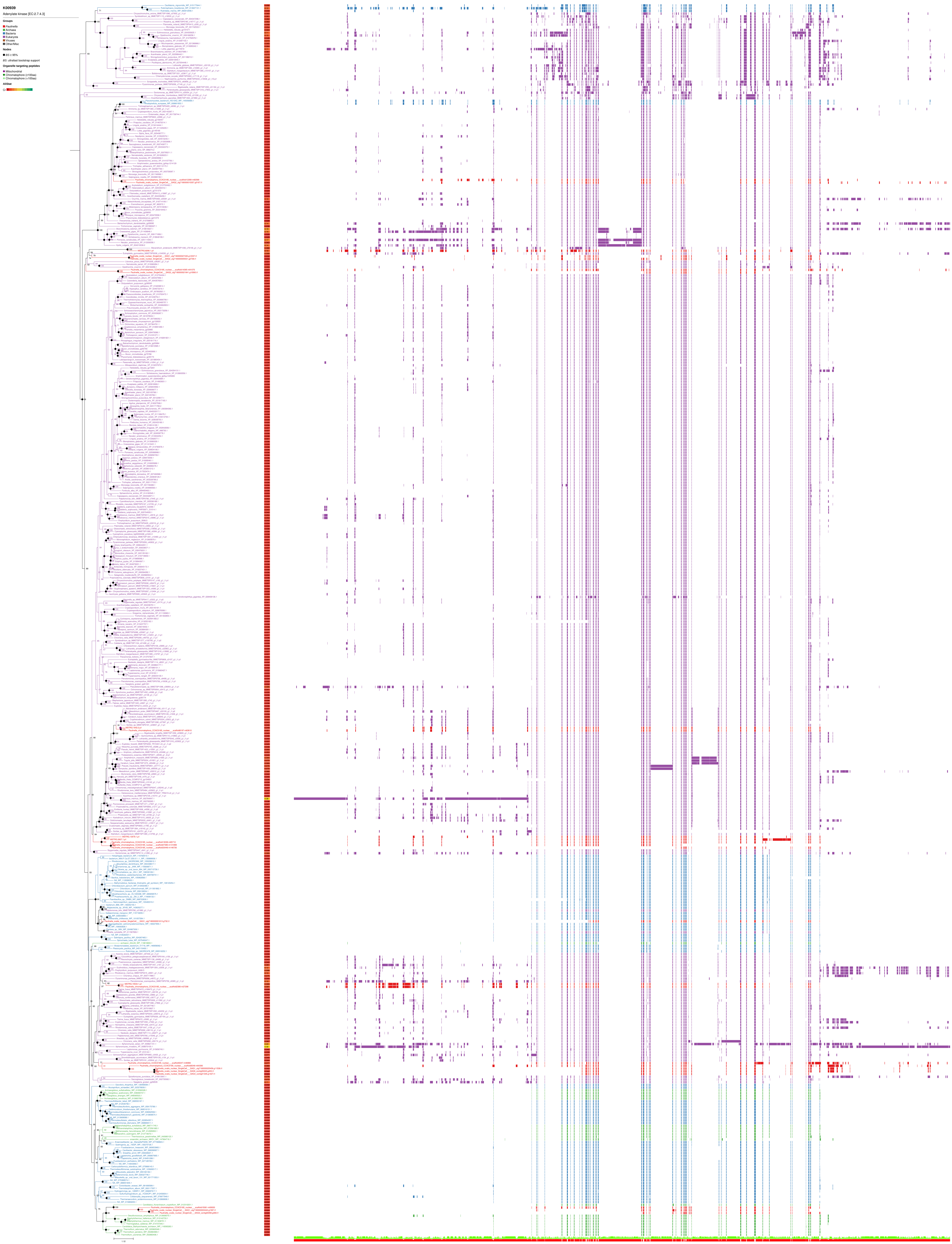

K00939
Adenylate kinase [EC:2.7.4.3]
Groups
Paulinella
Archaea
Bacteria
Eukaryota
Viruses
Other/Misc
Nodes
BS ≥ 95%
BS: ultrafast bootstrap support
Organelle targeting peptides
Mitochondrial
Chromatophore (≥100aa)
Chromatophore (<100aa)
AliStat
Cr 0 1

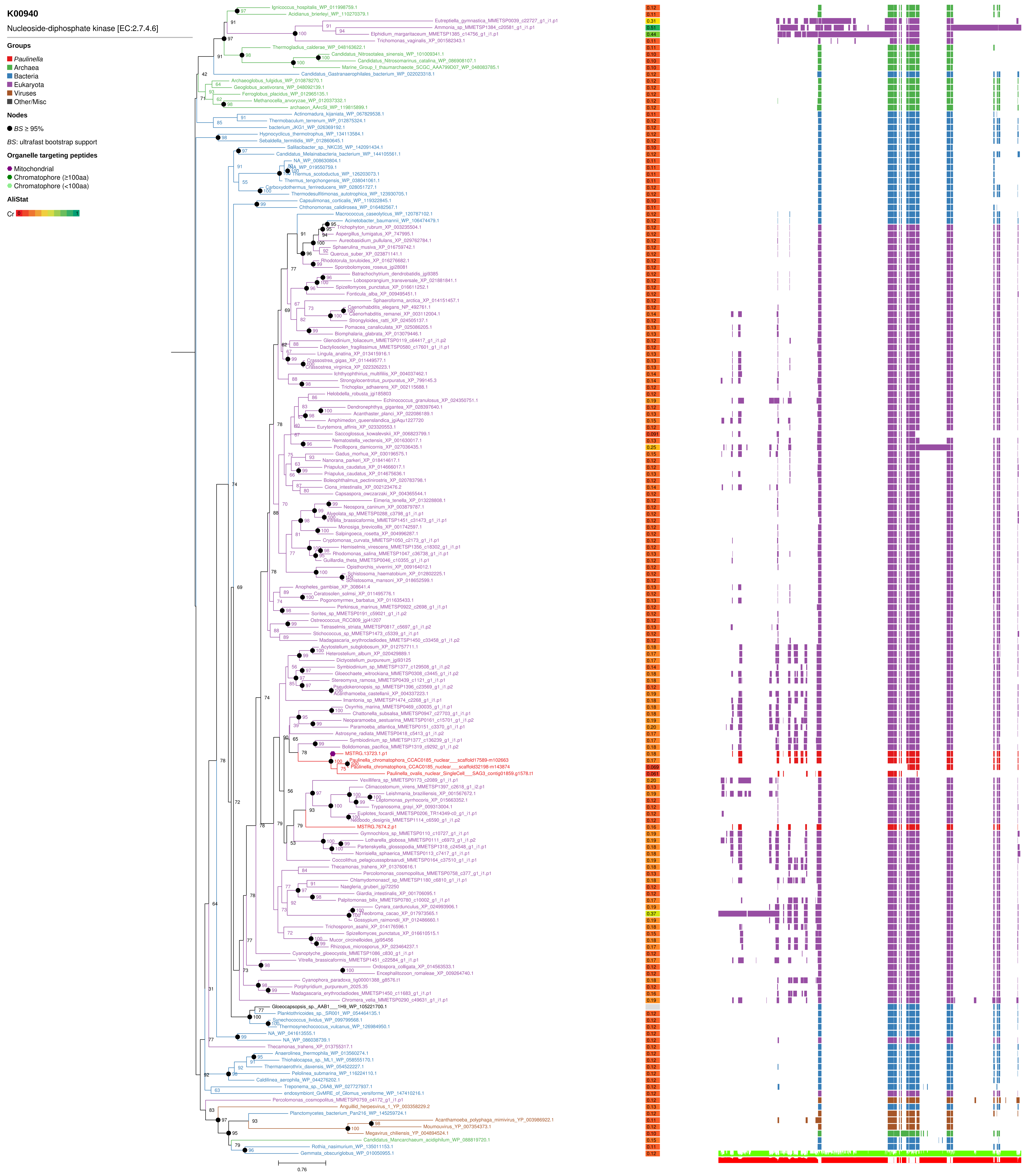

K00940
Nucleoside-diphosphate kinase [EC:2.7.4.6]
Groups
Paulinella
Archaea
Bacteria
Eukaryota
Viruses
Other/Misc
Nodes
BS ≥ 95%
BS: ultrafast bootstrap support
Organelle targeting peptides
Mitochondrial
Chromatophore (≥100aa)
Chromatophore (<100aa)
AliStat
Cr 0 1
0.76

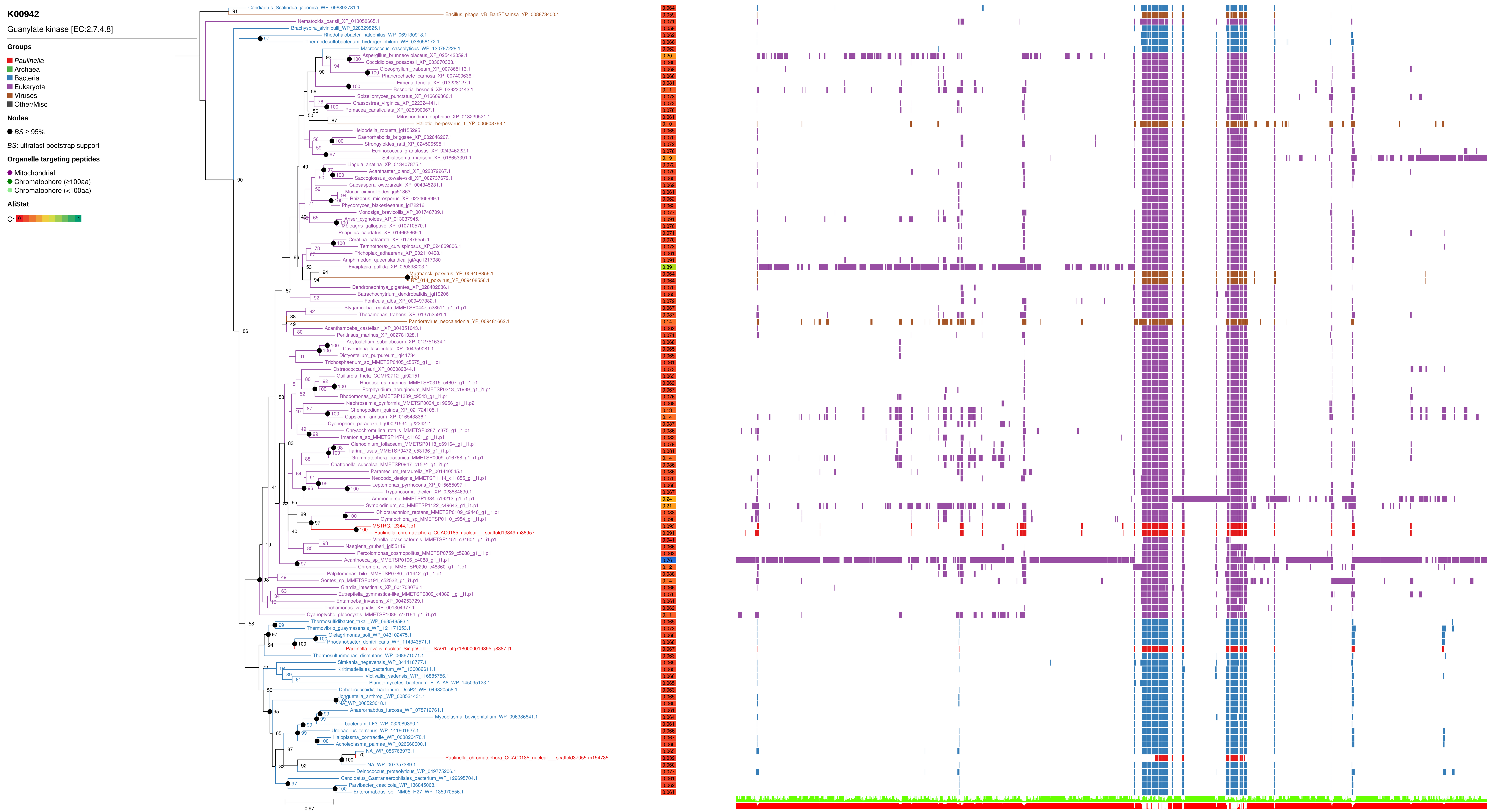

K00942
Guanylate kinase [EC:2.7.4.8]
Groups
Paulinella
Archaea
Bacteria
Eukaryota
Viruses
Other/Misc
Nodes
BS ≥ 95%
BS: ultrafast bootstrap support
Organelle targeting peptides
Mitochondrial
Chromatophore (≥100aa)
Chromatophore (<100aa)
AliStat
Cr
0.97

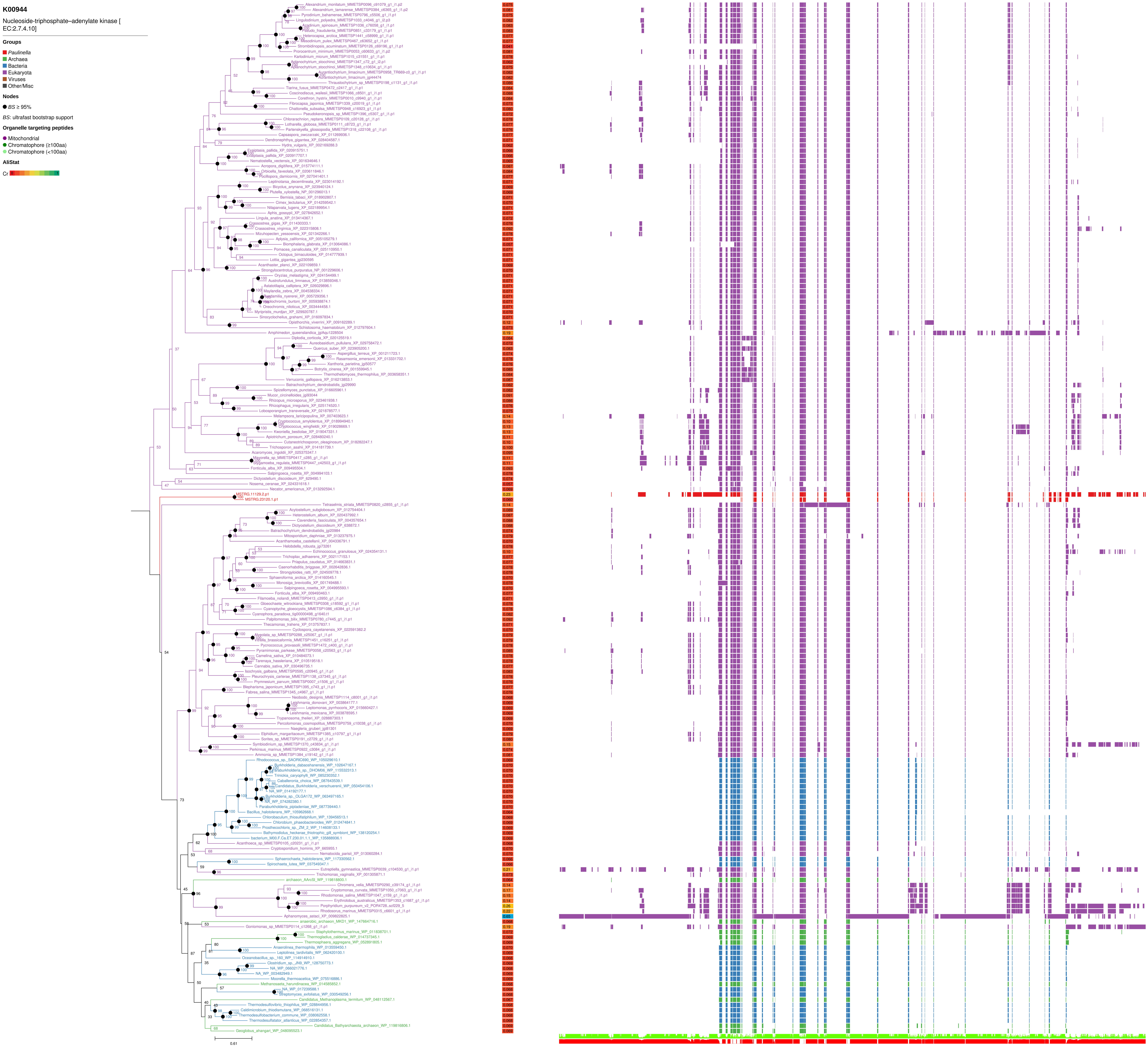
K00944
Nucleoside-triphosphate–adenylate kinase [EC:2.7.4.10]
Groups
Paulinella
Archaea
Bacteria
Eukaryota
Viruses
Other/Misc
Nodes
BS ≥ 95%
BS: ultrafast bootstrap support
Organelle targeting peptides
Mitochondrial
Chromatophore (≥100aa)
Chromatophore (<100aa)
AliStat
Cr
0.61

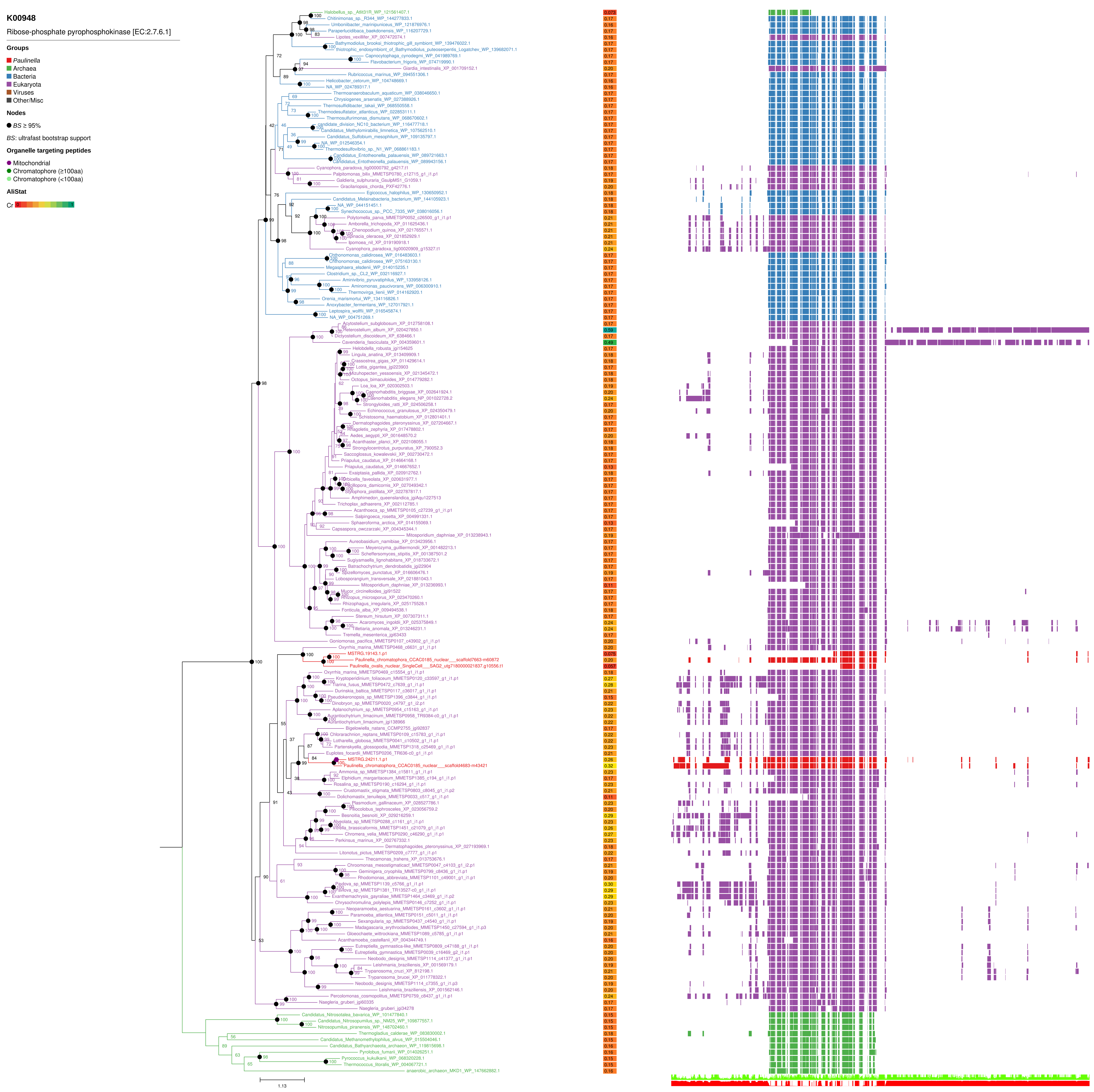
K00948
Ribose-phosphate pyrophosphokinase [EC:2.7.6.1]
Groups
Paulinella
Archaea
Bacteria
Eukaryota
Viruses
Other/Misc
Nodes
BS ≥ 95%
BS: ultrafast bootstrap support
Organelle targeting peptides
Mitochondrial
Chromatophore (≥100aa)
Chromatophore (<100aa)
AliStat
Cr
1.13

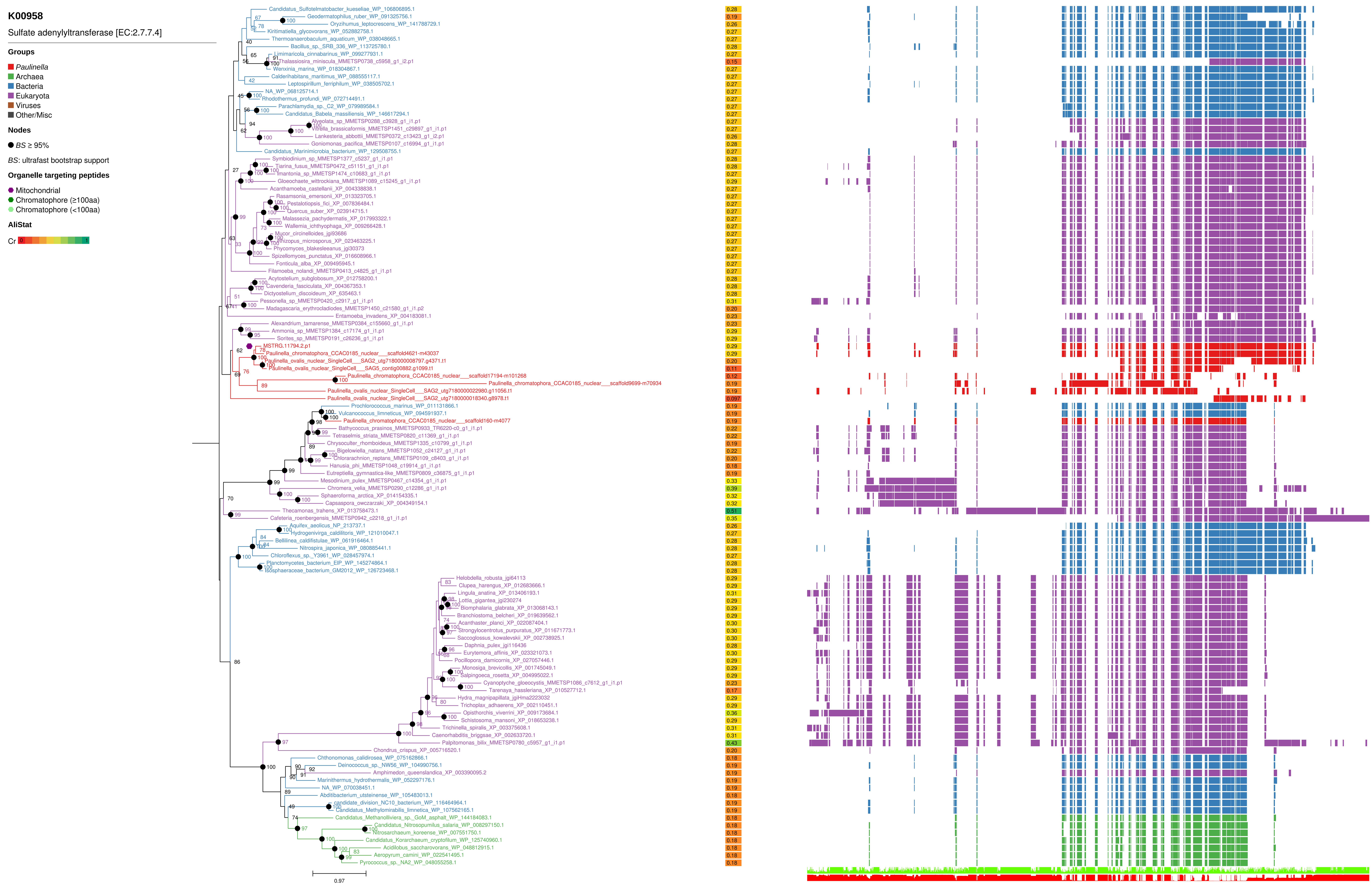
K00958
Sulfate adenylyltransferase [EC:2.7.7.4]
Groups
Paulinella
Archaea
Bacteria
Eukaryota
Viruses
Other/Misc
Nodes
BS ≥ 95%
BS: ultrafast bootstrap support
Organelle targeting peptides
Mitochondrial
Chromatophore (≥100aa)
Chromatophore (<100aa)
AliStat
Cr 0 1
Candidatus_Sulfotelmatobacter_kueseliae_WP_106806895.1
Geodermatophilus_ruber_WP_091325756.1
Oryzihumus_leptocrescens_WP_141788729.1
Kiritimatiella_glycovorans_WP_052882758.1
Thermoanaerobaculum_aquaticum_WP_038048665.1
Bacillus_sp._SRB_336_WP_113725780.1
Limimaricola_cinnabarinus_WP_099277931.1
Thalassiosira_miniscula_MMETSP0738_c5958_g1_i2.p1
Wenxinia_marina_WP_018304867.1
Calderihabitans_maritimus_WP_088555117.1
Leptospirillum_ferriphilum_WP_038505702.1
NA_WP_068125714.1
Rhodothermus_profundi_WP_072714491.1
Parachlamydia_sp._C2_WP_079989584.1
Candidatus_Babela_massiliensis_WP_146617294.1
Alveolata_sp_MMETSP0288_c3928_g1_i1.p1
Vitrella_brassicaformis_MMETSP1451_c29897_g1_i1.p1
Lankesteria_abbottii_MMETSP0372_c13423_g1_i2.p1
Goniomonas_pacifica_MMETSP0107_c16994_g1_i1.p1
Candidatus_Marinimicrobia_bacterium_WP_129508755.1
Symbiodinium_sp_MMETSP1377_c5237_g1_i1.p1
Tiarina_fusus_MMETSP0472_c51151_g1_i1.p1
Imantonia_sp_MMETSP1474_c10683_g1_i1.p1
Gloeochaete_wittrockiana_MMETSP1089_c15245_g1_i1.p1
Acanthamoeba_castellanii_XP_004338838.1
Rasamsonia_emersonii_XP_013323705.1
Pestalotiopsis_fici_XP_007836484.1
Quercus_suber_XP_023914715.1
Malassezia_pachydermatis_XP_017993322.1
Wallemia_ichthyophaga_XP_009266428.1
Mucor_circinelloides_jgi93686
Rhizopus_microsporus_XP_023463225.1
Phycomyces_blakesleeanus_jgi30373
Spizellomyces_punctatus_XP_016608966.1
Fonticula_alba_XP_009495945.1
Filamoeba_nolandi_MMETSP0413_c4825_g1_i1.p1
Acytostelium_subglobosum_XP_012758200.1
Cavenderia_fasciculata_XP_004367353.1
Dictyostelium_discoideum_XP_635463.1
Pessonella_sp_MMETSP0420_c2917_g1_i1.p1
Madagascaria_erythrocladiodes_MMETSP1450_c21580_g1_i1.p2
Entamoeba_invadens_XP_004183081.1
Alexandrium_tamarense_MMETSP0384_c155660_g1_i1.p1
Ammonia_sp_MMETSP1384_c17174_g1_i1.p1
Sorites_sp_MMETSP0191_c26236_g1_i1.p1
MSTRG.11794.2.p1
Paulinella_chromatophora_CCAC0185_nuclear___scaffold4621-m43037
Paulinella_ovalis_nuclear_SingleCell___SAG2_utg7180000008797.g4371.t1
Paulinella_ovalis_nuclear_SingleCell___SAG5_contig00882.g1099.t1
Paulinella_chromatophora_CCAC0185_nuclear___scaffold17194-m101268
Paulinella_chromatophora_CCAC0185_nuclear___scaffold9699-m70934
Paulinella_ovalis_nuclear_SingleCell___SAG2_utg7180000022980.g11056.t1
Paulinella_ovalis_nuclear_SingleCell___SAG2_utg7180000018340.g8978.t1
Prochlorococcus_marinus_WP_011131866.1
Vulcanococcus_limneticus_WP_094591937.1
Paulinella_chromatophora_CCAC0185_nuclear___scaffold160-m4077
Bathycoccus_prasinos_MMETSP0933_TR6220-c0_g1_i1.p1
Tetraselmis_striata_MMETSP0820_c11369_g1_i1.p1
Chrysoculter_rhomboideus_MMETSP1335_c10799_g1_i1.p1
Bigelowiella_natans_MMETSP1052_c24127_g1_i1.p1
Chlorarachnion_reptans_MMETSP0109_c8403_g1_i1.p1
Hanusia_phi_MMETSP1048_c19914_g1_i1.p1
Eutreptiella_gymnastica-like_MMETSP0809_c36875_g1_i1.p1
Mesodinium_pulex_MMETSP0467_c14354_g1_i1.p1
Chromera_velia_MMETSP0290_c12286_g1_i1.p1
Sphaeroforma_arctica_XP_014154335.1
Capsaspora_owczarzaki_XP_004349154.1
Thecamonas_trahens_XP_013758473.1
Cafeteria_roenbergensis_MMETSP0942_c2218_g1_i1.p1
Aquifex_aeolicus_NP_213737.1
Hydrogenivirga_caldilitoris_WP_121010047.1
Bellilinea_caldifistulae_WP_061916464.1
Nitrospira_japonica_WP_080885441.1
Chloroflexus_sp._Y3961_WP_028457974.1
Planctomycetes_bacterium_EiP_WP_145274864.1
Isosphaeraceae_bacterium_GM2012_WP_126723468.1
Helobdella_robusta_jgi64113
Clupea_harengus_XP_012683666.1
Lingula_anatina_XP_013406193.1
Lottia_gigantea_jgi230274
Biomphalaria_glabrata_XP_013068143.1
Branchiostoma_belcheri_XP_019639562.1
Acanthaster_planci_XP_022087404.1
Strongylocentrotus_purpuratus_XP_011671773.1
Saccoglossus_kowalevskii_XP_002738925.1
Daphnia_pulex_jgi116436
Eurytemora_affinis_XP_023321073.1
Pocillopora_damicornis_XP_027057446.1
Monosiga_brevicollis_XP_001745049.1
Salpingoeca_rosetta_XP_004995022.1
Gyanoptyche_gloeocystis_MMETSP1086_c7612_g1_i1.p1
Tarenaya_hassleriana_XP_010527712.1
Hydra_magnipapillata_jgiHma2223032
Trichoplax_adhaerens_XP_002110451.1
Opisthorchis_viverrini_XP_009173684.1
Schistosoma_mansoni_XP_018653238.1
Trichinella_spiralis_XP_003375608.1
Caenorhabditis_briggsae_XP_002633720.1
Palpitomonas_bilix_MMETSP0780_c5957_g1_i1.p1
Chondrus_crispus_XP_005716520.1
Chthonomonas_calidirosea_WP_075162866.1
Deinococcus_sp._NW56_WP_104990756.1
Amphimedon_queenslandica_XP_003390095.2
Marinithermus_hydrothermalis_WP_052297176.1
NA_WP_070038451.1
Abditibacterium_utsteinense_WP_105483013.1
candidate_division_NC10_bacterium_WP_116464964.1
Candidatus_Methylomirabilis_limnetica_WP_107562165.1
Candidatus_Methanolliviera_sp._GoM_asphalt_WP_144184083.1
Candidatus_Nitrosopumilus_salaria_WP_008297150.1
Nitrosarchaeum_koreense_WP_007551750.1
Candidatus_Korarchaeum_cryptofilum_WP_125740960.1
Acidilobus_saccharovorans_WP_048812915.1
Aeropyrum_camini_WP_022541495.1
Pyrococcus_sp._NA2_WP_048055258.1
0.97

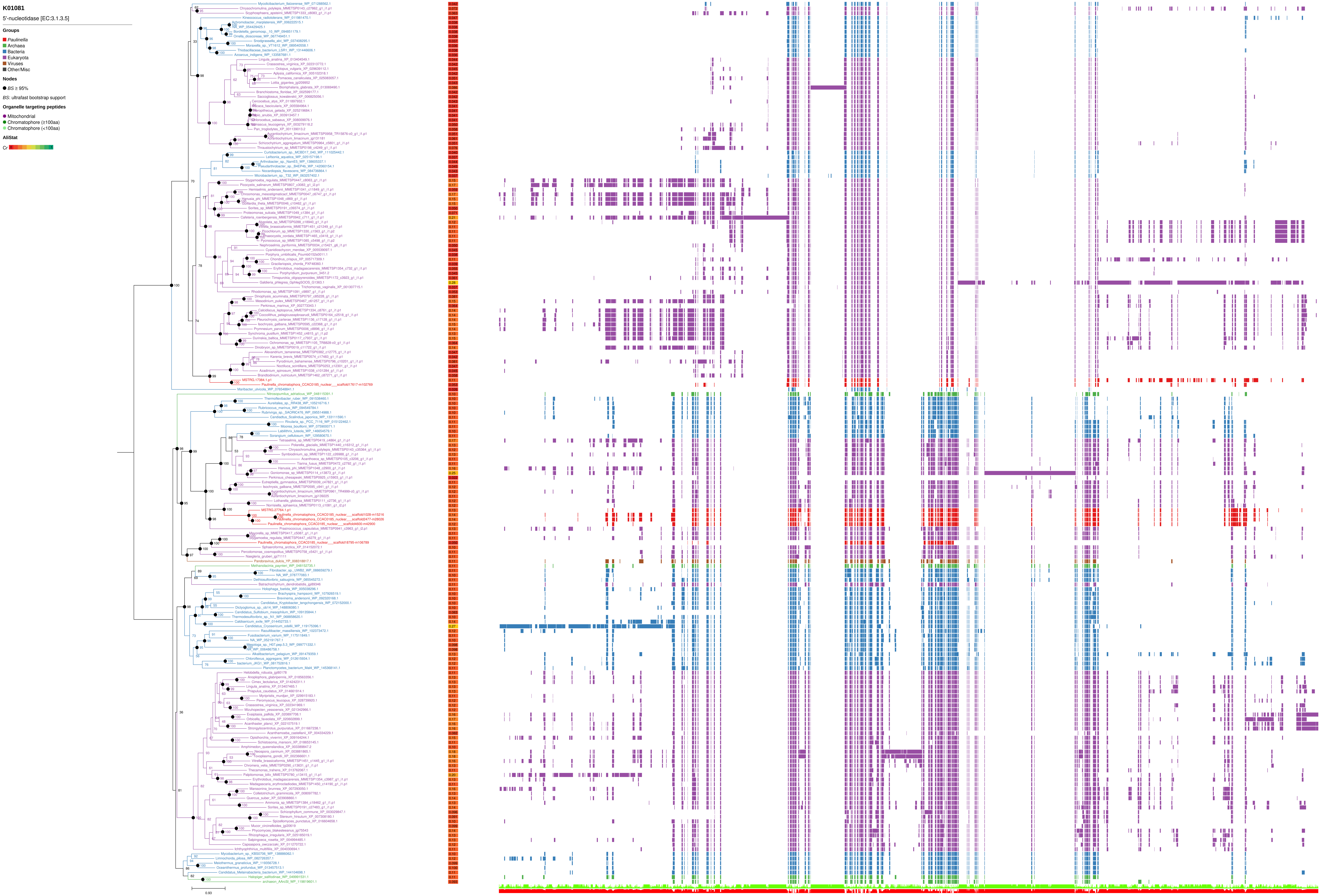

K01081
5'-nucleotidase [EC:3.1.3.5]
Groups
Paulinella
Archaea
Bacteria
Eukaryota
Viruses
Other/Misc
Nodes
BS ≥ 95%
BS: ultrafast bootstrap support
Organelle targeting peptides
Mitochondrial
Chromatophore (≥100aa)
Chromatophore (<100aa)
AliStat
Cr
0.93

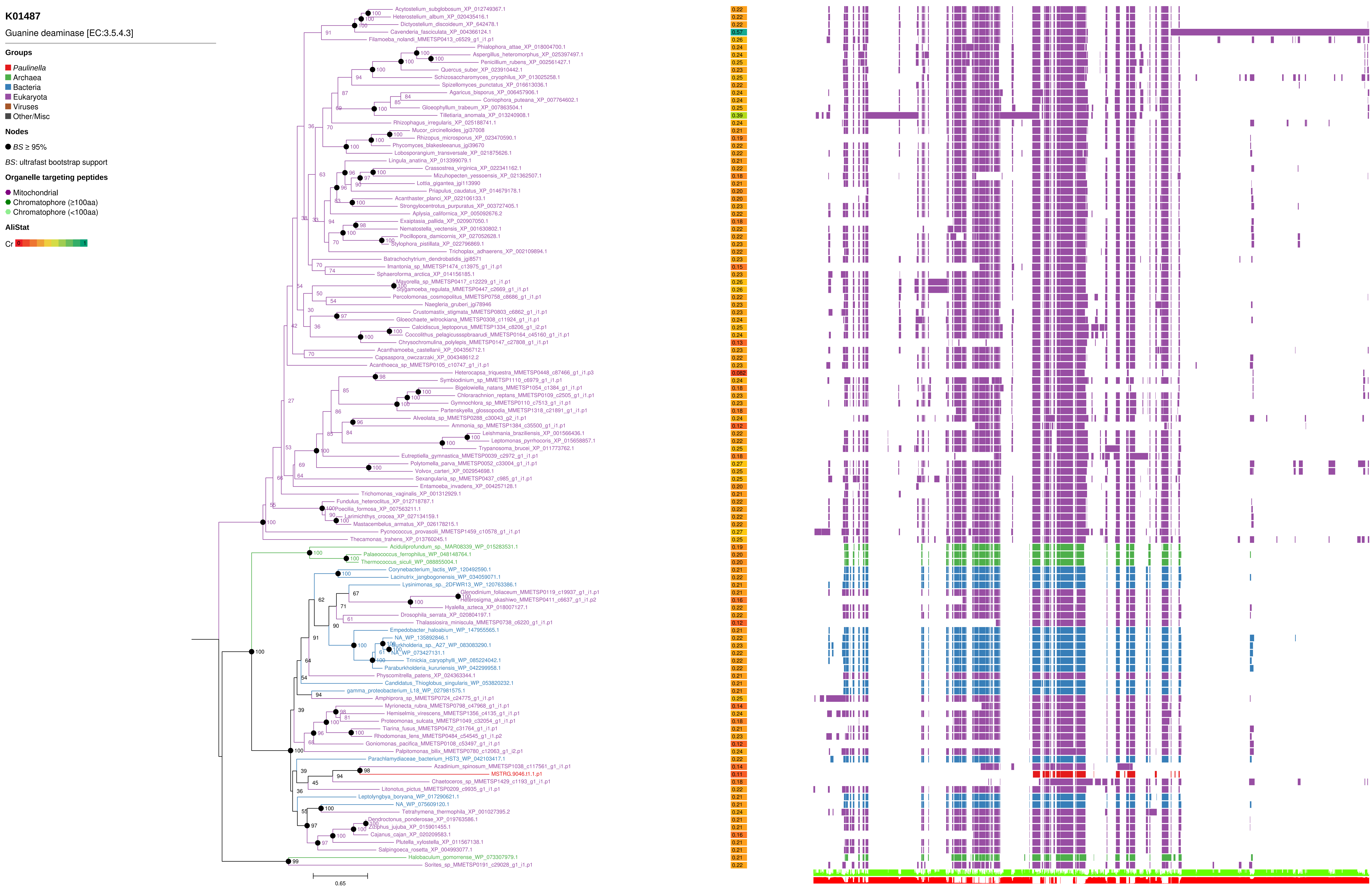

K01487
Guanine deaminase [EC:3.5.4.3]
Groups
Paulinella
Archaea
Bacteria
Eukaryota
Viruses
Other/Misc
Nodes
BS ≥ 95%
BS: ultrafast bootstrap support
Organelle targeting peptides
Mitochondrial
Chromatophore (≥100aa)
Chromatophore (<100aa)
AliStat
Cr 0 1
Acytostelium_subglobosum_XP_012749367.1
Heterostelium_album_XP_020435416.1
Dictyostelium_discoideum_XP_642478.1
Cavenderia_fasciculata_XP_004366124.1
Filamoeba_nolandi_MMETSP0413_c6529_g1_i1.p1
Phialophora_attae_XP_018004700.1
Aspergillus_heteromorphus_XP_025397497.1
Penicillium_rubens_XP_002561427.1
Quercus_suber_XP_023910442.1
Schizosaccharomyces_cryophilus_XP_013025258.1
Spizellomyces_punctatus_XP_016613036.1
Agaricus_bisporus_XP_006457906.1
Coniophora_puteana_XP_007764602.1
Gloeophyllum_trabeum_XP_007863504.1
Tilletiaria_anomala_XP_013240908.1
Rhizophagus_irregularis_XP_025188741.1
Mucor_circinelloides_jgi37008
Rhizopus_microsporus_XP_023470590.1
Phycomyces_blakesleeanus_jgi39670
Lobosporangium_transversale_XP_021875626.1
Lingula_anatina_XP_013399079.1
Crassostrea_virginica_XP_022341162.1
Mizuhopecten_yessoensis_XP_021362507.1
Lottia_gigantea_jgi113990
Priapulus_caudatus_XP_014679178.1
Acanthaster_planci_XP_022106133.1
Strongylocentrotus_purpuratus_XP_003727405.1
Aplysia_californica_XP_005092676.2
Exaiptasia_pallida_XP_020907050.1
Nematostella_vectensis_XP_001630802.1
Pocillopora_damicornis_XP_027052628.1
Stylophora_pistillata_XP_022796869.1
Trichoplax_adhaerens_XP_002109894.1
Batrachochytrium_dendrobatidis_jgi8571
Imantonia_sp_MMETSP1474_c13975_g1_i1.p1
Sphaeroforma_arctica_XP_014156185.1
Mayorella_sp_MMETSP0417_c12229_g1_i1.p1
Stygamoeba_regulata_MMETSP0447_c2669_g1_i1.p1
Percolomonas_cosmopolitus_MMETSP0758_c8686_g1_i1.p1
Naegleria_gruberi_jgi78946
Crustomastix_stigmata_MMETSP0803_c6862_g1_i1.p1
Gloeochaete_witrockiana_MMETSP0308_c11924_g1_i1.p1
Calcidiscus_leptoporus_MMETSP1334_c8206_g1_i2.p1
Coccolithus_pelagicussspbraarudi_MMETSP0164_c45160_g1_i1.p1
Chrysochromulina_polylepis_MMETSP0147_c27808_g1_i1.p1
Acanthamoeba_castellanii_XP_004356712.1
Capsaspora_owczarzaki_XP_004348612.2
Acanthoeca_sp_MMETSP0105_c10747_g1_i1.p1
Heterocapsa_triquestra_MMETSP0448_c87466_g1_i1.p3
Symbiodinium_sp_MMETSP1110_c6979_g1_i1.p1
Bigelowiella_natans_MMETSP1054_c1384_g1_i1.p1
Chlorarachnion_reptans_MMETSP0109_c2505_g1_i1.p1
Gymnochlora_sp_MMETSP0110_c7513_g1_i1.p1
Partenskyella_glossopodia_MMETSP1318_c21891_g1_i1.p1
Alveolata_sp_MMETSP0288_c30043_g2_i1.p1
Ammonia_sp_MMETSP1384_c35500_g1_i1.p1
Leishmania_braziliensis_XP_001566436.1
Leptomonas_pyrrhocoris_XP_015658857.1
Trypanosoma_brucei_XP_011773762.1
Eutreptiella_gymnastica_MMETSP0039_c2972_g1_i1.p1
Polytomella_parva_MMETSP0052_c33004_g1_i1.p1
Volvox_carteri_XP_002954698.1
Sexangularia_sp_MMETSP0437_c985_g1_i1.p1
Entamoeba_invadens_XP_004257128.1
Trichomonas_vaginalis_XP_001312929.1
Fundulus_heteroclitus_XP_012718787.1
Poecilia_formosa_XP_007563211.1
Larimichthys_crocea_XP_027134159.1
Mastacembelus_armatus_XP_026178215.1
Pycnococcus_provasolii_MMETSP1459_c10578_g1_i1.p1
Thecamonas_trahens_XP_013760245.1
Aciduliprofundum_sp._MAR08339_WP_015283531.1
Palaeococcus_ferrophilus_WP_048148764.1
Thermococcus_siculi_WP_088855004.1
Corynebacterium_lactis_WP_120492590.1
Lacinutrix_jangbogonensis_WP_034059071.1
Lysinimonas_sp._2DFWR13_WP_120763386.1
Glenodinium_foliaceum_MMETSP0119_c19937_g1_i1.p1
Heterosigma_akashiwo_MMETSP0411_c6637_g1_i1.p2
Hyalella_azteca_XP_018007127.1
Drosophila_serrata_XP_020804197.1
Thalassiosira_miniscula_MMETSP0738_c6220_g1_i1.p1
Empedobacter_haloabium_WP_147955565.1
NA_WP_135892846.1
Burkholderia_sp._A27_WP_083083290.1
NA_WP_073427131.1
Trinickia_caryophylli_WP_085224042.1
Paraburkholderia_kururiensis_WP_042299958.1
Physcomitrella_patens_XP_024363344.1
Candidatus_Thioglobus_singularis_WP_053820232.1
gamma_proteobacterium_L18_WP_027981575.1
Amphiprora_sp_MMETSP0724_c24775_g1_i1.p1
Myrionecta_rubra_MMETSP0798_c47968_g1_i1.p1
Hemiselmis_virescens_MMETSP1356_c4135_g1_i1.p1
Proteomonas_sulcata_MMETSP1049_c32054_g1_i1.p1
Tiarina_fusus_MMETSP0472_c31764_g1_i1.p1
Rhodomonas_lens_MMETSP0484_c54545_g1_i1.p2
Goniomonas_pacifica_MMETSP0108_c53497_g1_i1.p1
Palpitomonas_bilix_MMETSP0780_c12063_g1_i2.p1
Parachlamydiaceae_bacterium_HST3_WP_042103417.1
Azadinium_spinosum_MMETSP1038_c117561_g1_i1.p1
MSTRG.9046.t1.1.p1
Chaetoceros_sp_MMETSP1429_c1193_g1_i1.p1
Litonotus_pictus_MMETSP0209_c9935_g1_i1.p1
Leptolyngbya_boryana_WP_017290621.1
NA_WP_075609120.1
Tetrahymena_thermophila_XP_001027395.2
Dendroctonus_ponderosae_XP_019763586.1
Ziziphus_jujuba_XP_015901455.1
Cajanus_cajan_XP_020209583.1
Plutella_xylostella_XP_011567138.1
Salpingoeca_rosetta_XP_004993077.1
Halobaculum_gomorrense_WP_073307979.1
Sorites_sp_MMETSP0191_c29028_g1_i1.p1
0.65

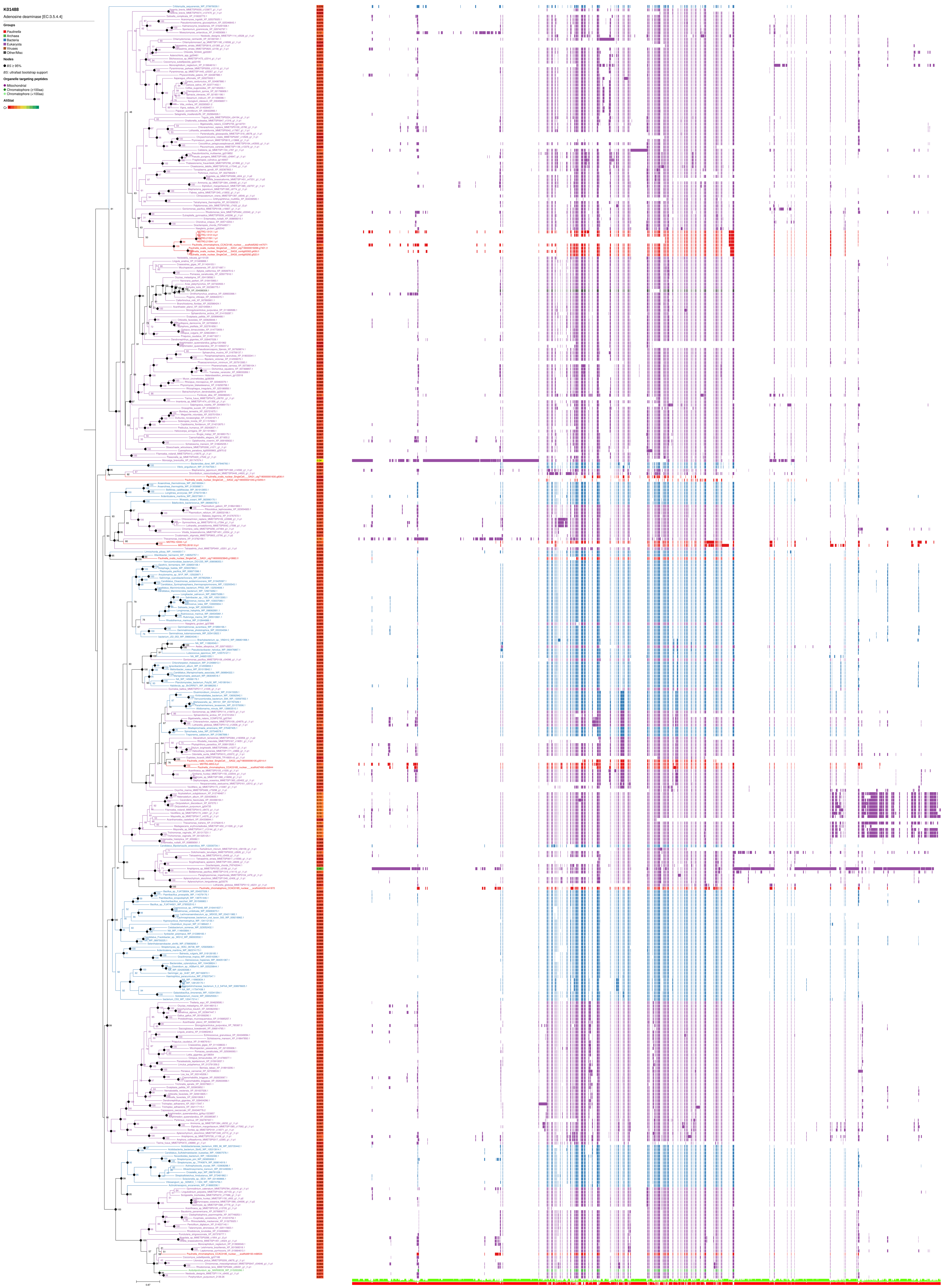

K01488
Adenosine deaminase [EC:3.5.4.4]
Groups
Paulinella
Archaea
Bacteria
Eukaryota
Viruses
Other/Misc
Nodes
BS ≥ 95%
BS: ultrafast bootstrap support
Organelle targeting peptides
Mitochondrial
Chromatophore (≥100aa)
Chromatophore (<100aa)
AliStat
Cr 0 1

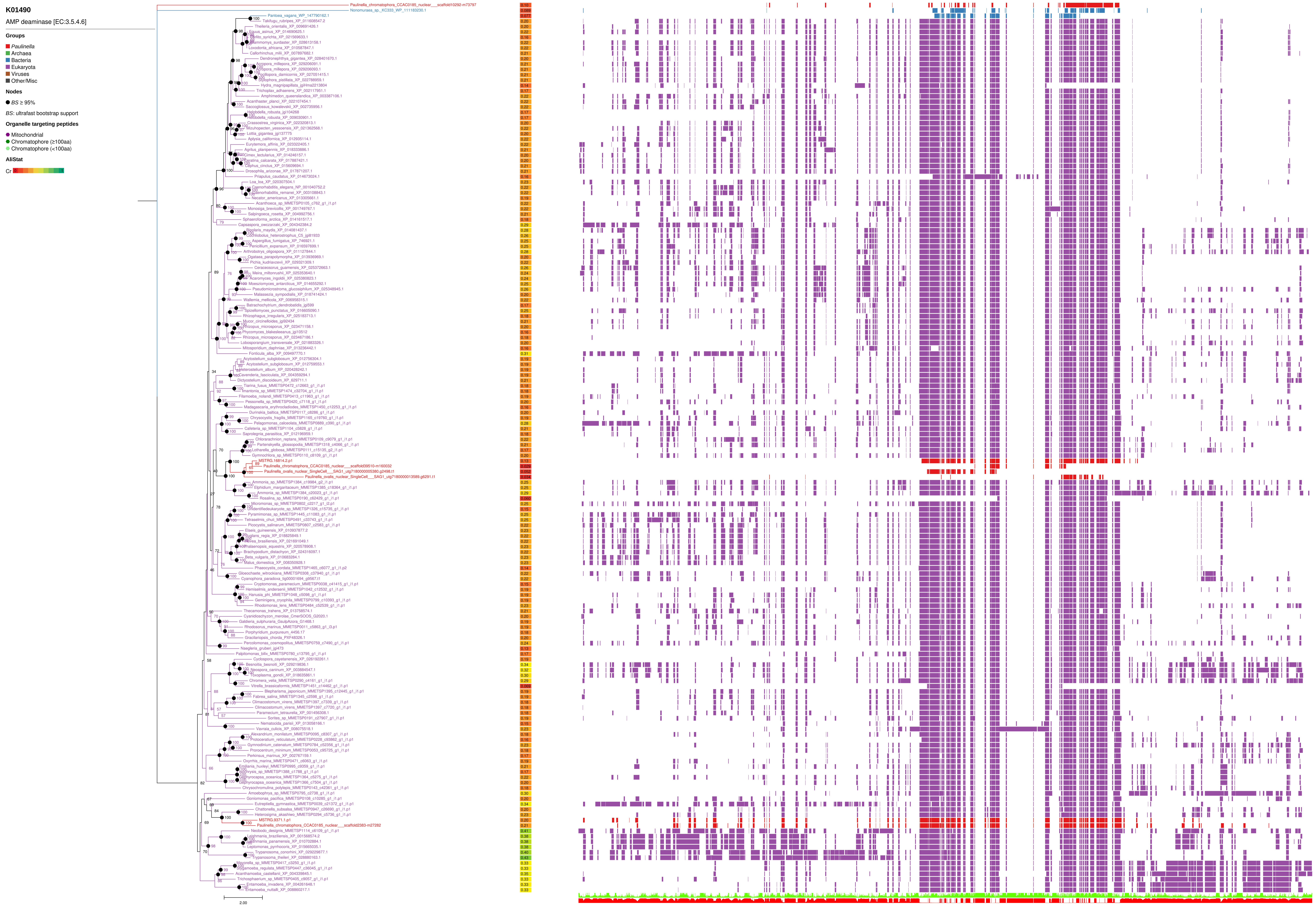

K01490
AMP deaminase [EC:3.5.4.6]
Groups
Paulinella
Archaea
Bacteria
Eukaryota
Viruses
Other/Misc
Nodes
BS ≥ 95%
BS: ultrafast bootstrap support
Organelle targeting peptides
Mitochondrial
Chromatophore (≥100aa)
Chromatophore (<100aa)
AliStat
Cr
2.00

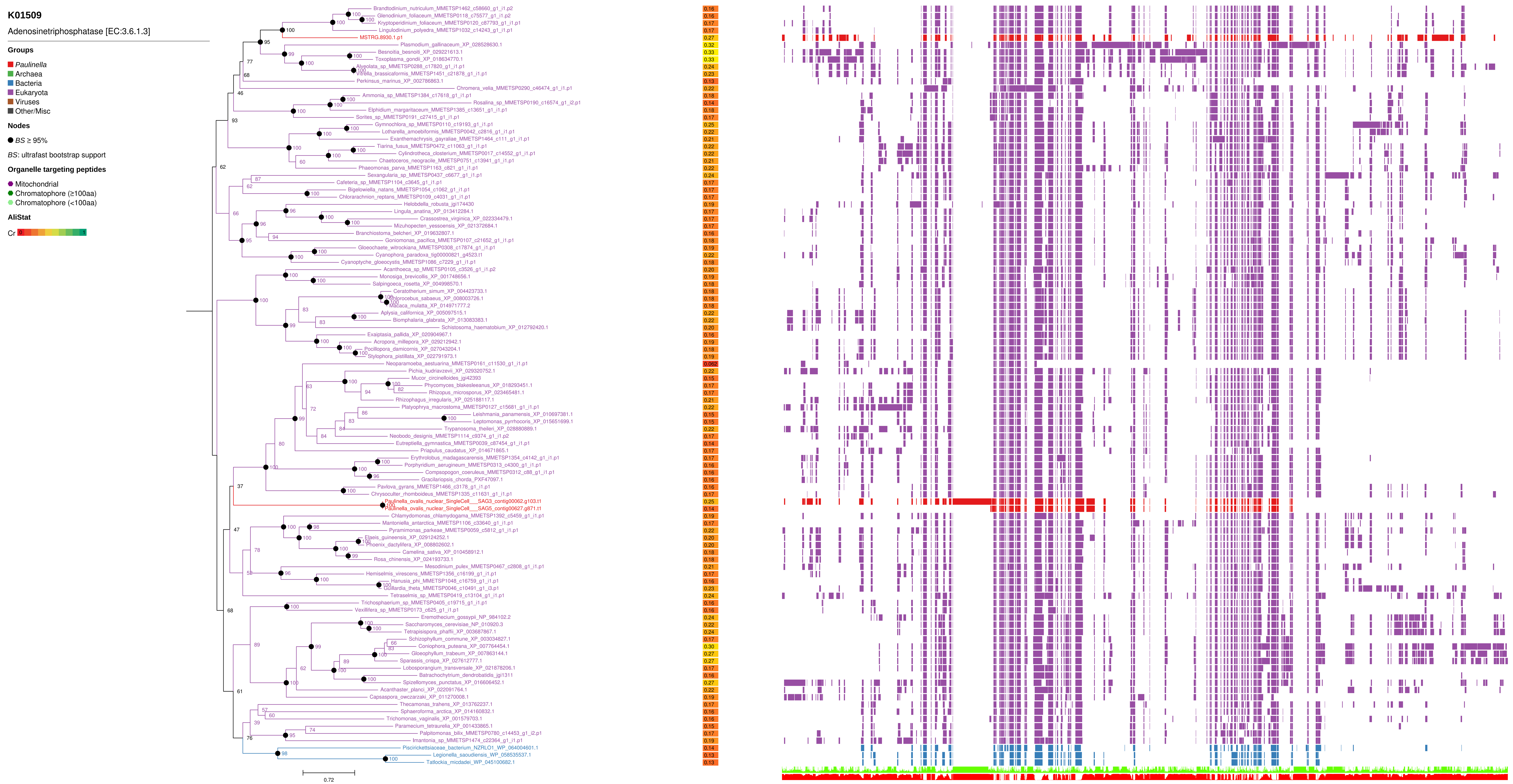

K01509
Adenosinetriphosphatase [EC:3.6.1.3]
Groups
Paulinella
Archaea
Bacteria
Eukaryota
Viruses
Other/Misc
Nodes
BS ≥ 95%
BS: ultrafast bootstrap support
Organelle targeting peptides
Mitochondrial
Chromatophore (≥100aa)
Chromatophore (<100aa)
AliStat
Cr
Brandtodinium_nutriculum_MMETSP1462_c58660_g1_i1.p2
Glenodinium_foliaceum_MMETSP0118_c75577_g1_i1.p2
Kryptoperidinium_foliaceum_MMETSP0120_c87793_g1_i1.p1
Lingulodinium_polyedra_MMETSP1032_c14243_g1_i1.p1
MSTRG.8930.1.p1
Plasmodium_gallinaceum_XP_028528630.1
Besnoitia_besnoiti_XP_029221613.1
Toxoplasma_gondii_XP_018634770.1
Alveolata_sp_MMETSP0288_c17820_g1_i1.p1
Vitrella_brassicaformis_MMETSP1451_c21878_g1_i1.p1
Perkinsus_marinus_XP_002786863.1
Chromera_velia_MMETSP0290_c46474_g1_i1.p1
Ammonia_sp_MMETSP1384_c17618_g1_i1.p1
Rosalina_sp_MMETSP0190_c16574_g1_i2.p1
Elphidium_margaritaceum_MMETSP1385_c13651_g1_i1.p1
Sorites_sp_MMETSP0191_c27415_g1_i1.p1
Gymnochlora_sp_MMETSP0110_c19193_g1_i1.p1
Lotharella_amoebiformis_MMETSP0042_c2816_g1_i1.p1
Exanthemachrysis_gayraliae_MMETSP1464_c111_g1_i1.p1
Tiarina_fusus_MMETSP0472_c11063_g1_i1.p1
Cylindrotheca_closterium_MMETSP0017_c14552_g1_i1.p1
Chaetoceros_neogracile_MMETSP0751_c13941_g1_i1.p1
Phaeomonas_parva_MMETSP1163_c821_g1_i1.p1
Sexangularia_sp_MMETSP0437_c6677_g1_i1.p1
Cafeteria_sp_MMETSP1104_c3645_g1_i1.p1
Bigelowiella_natans_MMETSP1054_c1062_g1_i1.p1
Chlorarachnion_reptans_MMETSP0109_c4031_g1_i1.p1
Helobdella_robusta_jgi174430
Lingula_anatina_XP_013412284.1
Crassostrea_virginica_XP_022334479.1
Mizuhopecten_yessoensis_XP_021372684.1
Branchiostoma_belcheri_XP_019632807.1
Goniomonas_pacifica_MMETSP0107_c21652_g1_i1.p1
Gloeochaete_witrockiana_MMETSP0308_c17874_g1_i1.p1
Cyanophora_paradoxa_tig00000821_g4523.t1
Cyanoptyche_gloeocystis_MMETSP1086_c7229_g1_i1.p1
Acanthoeca_sp_MMETSP0105_c3526_g1_i1.p2
Monosiga_brevicollis_XP_001748656.1
Salpingoeca_rosetta_XP_004998570.1
Ceratotherium_simum_XP_004423733.1
Chlorocebus_sabaeus_XP_008003726.1
Macaca_mulatta_XP_014971777.2
Aplysia_californica_XP_005097515.1
Biomphalaria_glabrata_XP_013083383.1
Schistosoma_haematobium_XP_012792420.1
Exaiptasia_pallida_XP_020904967.1
Acropora_millepora_XP_029212942.1
Pocillopora_damicornis_XP_027043204.1
Stylophora_pistillata_XP_022791973.1
Neoparamoeba_aestuarina_MMETSP0161_c11530_g1_i1.p1
Pichia_kudriavzevii_XP_029320752.1
Mucor_circinelloides_jgi42393
Phycomyces_blakesleeanus_XP_018293451.1
Rhizopus_microsporus_XP_023465481.1
Rhizophagus_irregularis_XP_025188117.1
Platyophrya_macrostoma_MMETSP0127_c15681_g1_i1.p1
Leishmania_panamensis_XP_010697381.1
Leptomonas_pyrrhocoris_XP_015651699.1
Trypanosoma_theileri_XP_028880889.1
Neobodo_designis_MMETSP1114_c9374_g1_i1.p2
Eutreptiella_gymnastica_MMETSP0039_c87454_g1_i1.p1
Priapulus_caudatus_XP_014671865.1
Erythrolobus_madagascarensis_MMETSP1354_c4142_g1_i1.p1
Porphyridium_aerugineum_MMETSP0313_c4300_g1_i1.p1
Compsopogon_coeruleus_MMETSP0312_c88_g1_i1.p1
Gracilariopsis_chorda_PXF47097.1
Pavlova_gyrans_MMETSP1466_c3178_g1_i1.p1
Chrysoculter_rhomboideus_MMETSP1335_c11631_g1_i1.p1
Paulinella_ovalis_nuclear_SingleCell___SAG3_contig00062.g103.t1
Paulinella_ovalis_nuclear_SingleCell___SAG5_contig00627.g871.t1
Chlamydomonas_chlamydogama_MMETSP1392_c5459_g1_i1.p1
Mantoniella_antarctica_MMETSP1106_c33640_g1_i1.p1
Pyramimonas_parkeae_MMETSP0059_c5812_g1_i1.p1
Elaeis_guineensis_XP_029124252.1
Phoenix_dactylifera_XP_008802602.1
Camelina_sativa_XP_010458912.1
Rosa_chinensis_XP_024193733.1
Mesodinium_pulex_MMETSP0467_c2808_g1_i1.p1
Hemiselmis_virescens_MMETSP1356_c16199_g1_i1.p1
Hanusia_phi_MMETSP1048_c16759_g1_i1.p1
Guillardia_theta_MMETSP0046_c10491_g1_i3.p1
Tetraselmis_sp_MMETSP0419_c13104_g1_i1.p1
Trichosphaerium_sp_MMETSP0405_c19715_g1_i1.p1
Vexillifera_sp_MMETSP0173_c625_g1_i1.p1
Eremothecium_gossypii_NP_984102.2
Saccharomyces_cerevisiae_NP_010920.3
Tetrapisispora_phaffii_XP_003687867.1
Schizophyllum_commune_XP_003034827.1
Coniophora_puteana_XP_007764454.1
Gloeophyllum_trabeum_XP_007863144.1
Sparassis_crispa_XP_027612777.1
Lobosporangium_transversale_XP_021878206.1
Batrachochytrium_dendrobatidis_jgi1311
Spizellomyces_punctatus_XP_016606452.1
Acanthaster_planci_XP_022091764.1
Capsaspora_owczarzaki_XP_011270008.1
Thecamonas_trahens_XP_013762237.1
Sphaeroforma_arctica_XP_014160832.1
Trichomonas_vaginalis_XP_001579703.1
Paramecium_tetraurelia_XP_001433865.1
Palpitomonas_bilix_MMETSP0780_c14453_g1_i2.p1
Imantonia_sp_MMETSP1474_c22364_g1_i1.p1
Piscirickettsiaceae_bacterium_NZRLO1_WP_064004601.1
Legionella_saoudiensis_WP_058535537.1
Tatlockia_micdadei_WP_045100682.1
0.72

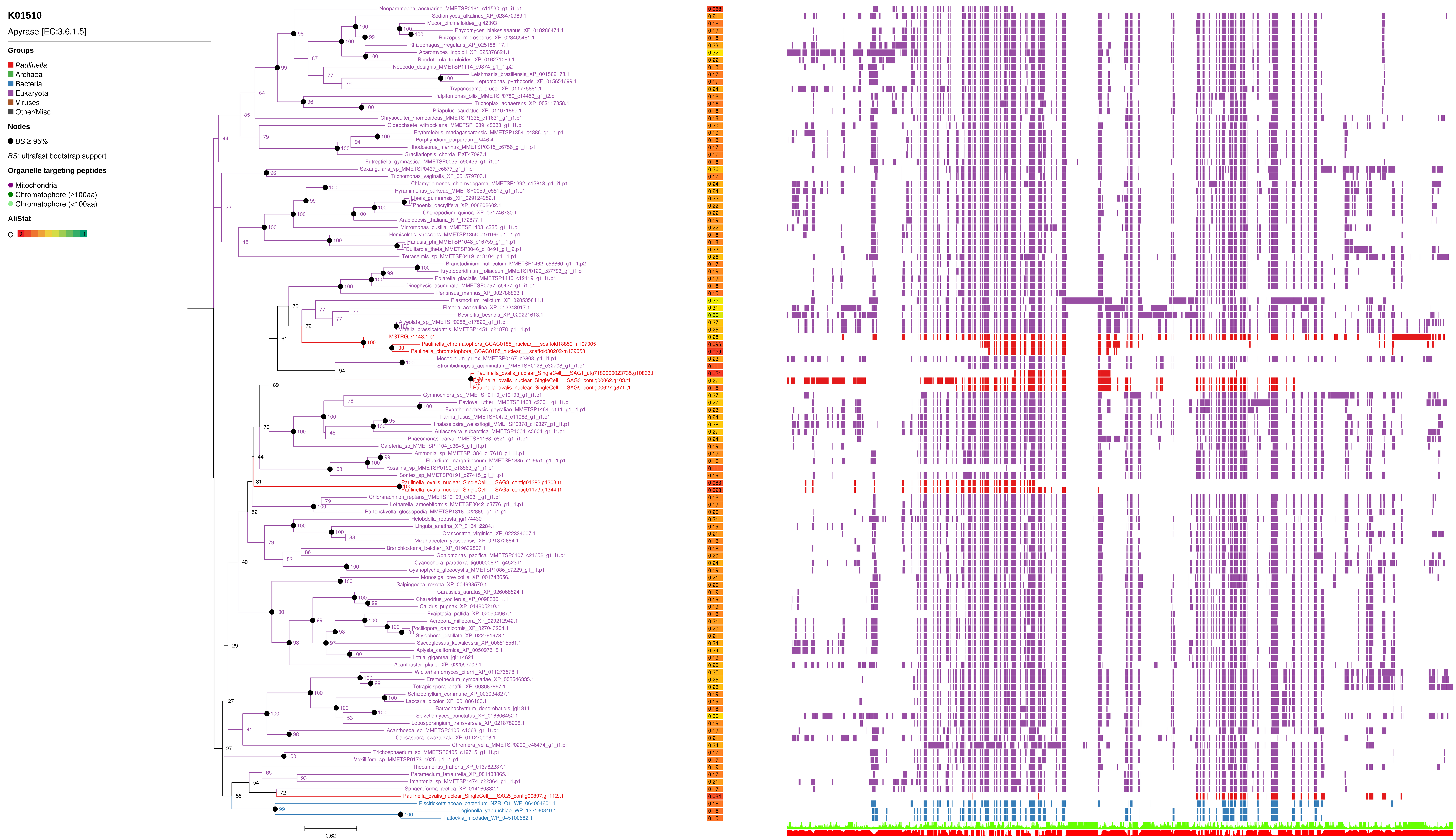
K01510
Apyrase [EC:3.6.1.5]
Groups
Paulinella
Archaea
Bacteria
Eukaryota
Viruses
Other/Misc
Nodes
BS ≥ 95%
BS: ultrafast bootstrap support
Organelle targeting peptides
Mitochondrial
Chromatophore (≥100aa)
Chromatophore (<100aa)
AliStat
Cr
0
1
Neoparamoeba_aestuarina_MMETSP0161_c11530_g1_i1.p1
Sodiomyces_alkalinus_XP_028470969.1
Mucor_circinelloides_jgi42393
Phycomyces_blakesleeanus_XP_018286474.1
Rhizopus_microsporus_XP_023465481.1
Rhizophagus_irregularis_XP_025188117.1
Acaromyces_ingoldii_XP_025376824.1
Rhodotorula_toruloides_XP_016271069.1
Neobodo_designis_MMETSP1114_c9374_g1_i1.p2
Leishmania_braziliensis_XP_001562178.1
Leptomonas_pyrrhocoris_XP_015651699.1
Trypanosoma_brucei_XP_011775681.1
Palpitomonas_bilix_MMETSP0780_c14453_g1_i2.p1
Trichoplax_adhaerens_XP_002117858.1
Priapulus_caudatus_XP_014671865.1
Chrysoculter_rhomboideus_MMETSP1335_c11631_g1_i1.p1
Gloeochaete_wittrockiana_MMETSP1089_c8333_g1_i1.p1
Erythrolobus_madagascarensis_MMETSP1354_c4886_g1_i1.p1
Porphyridium_purpureum_2446.4
Rhodosorus_marinus_MMETSP0315_c6756_g1_i1.p1
Gracilariopsis_chorda_PXF47097.1
Eutreptiella_gymnastica_MMETSP0039_c90439_g1_i1.p1
Sexangularia_sp_MMETSP0437_c6677_g1_i1.p1
Trichomonas_vaginalis_XP_001579703.1
Chlamydomonas_chlamydogama_MMETSP1392_c15813_g1_i1.p1
Pyramimonas_parkeae_MMETSP0059_c5812_g1_i1.p1
Elaeis_guineensis_XP_029124252.1
Phoenix_dactylifera_XP_008802602.1
Chenopodium_quinoa_XP_021746730.1
Arabidopsis_thaliana_NP_172877.1
Micromonas_pusilla_MMETSP1403_c335_g1_i1.p1
Hemiselmis_virescens_MMETSP1356_c16199_g1_i1.p1
Hanusia_phi_MMETSP1048_c16759_g1_i1.p1
Guillardia_theta_MMETSP0046_c10491_g1_i2.p1
Tetraselmis_sp_MMETSP0419_c13104_g1_i1.p1
Brandtodinium_nutriculum_MMETSP1462_c58660_g1_i1.p2
Kryptoperidinium_foliaceum_MMETSP0120_c87793_g1_i1.p1
Polarella_glacialis_MMETSP1440_c12119_g1_i1.p1
Dinophysis_acuminata_MMETSP0797_c5427_g1_i1.p1
Perkinsus_marinus_XP_002786863.1
Plasmodium_relictum_XP_028535841.1
Eimeria_acervulina_XP_013248917.1
Besnoitia_besnoiti_XP_029221613.1
Alveolata_sp_MMETSP0288_c17820_g1_i1.p1
Vitrella_brassicaformis_MMETSP1451_c21878_g1_i1.p1
MSTRG.21143.1.p1
Paulinella_chromatophora_CCAC0185_nuclear___scaffold18859-m107005
Paulinella_chromatophora_CCAC0185_nuclear___scaffold30202-m139053
Mesodinium_pulex_MMETSP0467_c2808_g1_i1.p1
Strombidinopsis_acuminatum_MMETSP0126_c32708_g1_i1.p1
Paulinella_ovalis_nuclear_SingleCell___SAG1_utg7180000023735.g10833.t1
Paulinella_ovalis_nuclear_SingleCell___SAG3_contig00062.g103.t1
Paulinella_ovalis_nuclear_SingleCell___SAG5_contig00627.g871.t1
Gymnochlora_sp_MMETSP0110_c19193_g1_i1.p1
Pavlova_lutheri_MMETSP1463_c2001_g1_i1.p1
Exanthemachrysis_gayraliae_MMETSP1464_c111_g1_i1.p1
Tiarina_fusus_MMETSP0472_c11063_g1_i1.p1
Thalassiosira_weissflogii_MMETSP0878_c12827_g1_i1.p1
Aulacoseira_subarctica_MMETSP1064_c3604_g1_i1.p1
Phaeomonas_parva_MMETSP1163_c821_g1_i1.p1
Cafeteria_sp_MMETSP1104_c3645_g1_i1.p1
Ammonia_sp_MMETSP1384_c17618_g1_i1.p1
Elphidium_margaritaceum_MMETSP1385_c13651_g1_i1.p1
Rosalina_sp_MMETSP0190_c18583_g1_i1.p1
Sorites_sp_MMETSP0191_c27415_g1_i1.p1
Paulinella_ovalis_nuclear_SingleCell___SAG3_contig01392.g1303.t1
Paulinella_ovalis_nuclear_SingleCell___SAG5_contig01173.g1344.t1
Chlorarachnion_reptans_MMETSP0109_c4031_g1_i1.p1
Lotharella_amoebiformis_MMETSP0042_c3776_g1_i1.p1
Partenskyella_glossopodia_MMETSP1318_c22885_g1_i1.p1
Helobdella_robusta_jgi174430
Lingula_anatina_XP_013412284.1
Crassostrea_virginica_XP_022334007.1
Mizuhopecten_yessoensis_XP_021372684.1
Branchiostoma_belcheri_XP_019632807.1
Goniomonas_pacifica_MMETSP0107_c21652_g1_i1.p1
Cyanophora_paradoxa_tig00000821_g4523.t1
Cyanoptyche_gloeocystis_MMETSP1086_c7229_g1_i1.p1
Monosiga_brevicollis_XP_001748656.1
Salpingoeca_rosetta_XP_004998570.1
Carassius_auratus_XP_026068524.1
Charadrius_vociferus_XP_009888611.1
Calidris_pugnax_XP_014805210.1
Exaiptasia_pallida_XP_020904967.1
Acropora_millepora_XP_029212942.1
Pocillopora_damicornis_XP_027043204.1
Stylophora_pistillata_XP_022791973.1
Saccoglossus_kowalevskii_XP_006815561.1
Aplysia_californica_XP_005097515.1
Lottia_gigantea_jgi114621
Acanthaster_planci_XP_022097702.1
Wickerhamomyces_ciferrii_XP_011276578.1
Eremothecium_cymbalariae_XP_003646335.1
Tetrapisispora_phaffii_XP_003687867.1
Schizophyllum_commune_XP_003034827.1
Laccaria_bicolor_XP_001886100.1
Batrachochytrium_dendrobatidis_jgi1311
Spizellomyces_punctatus_XP_016606452.1
Lobosporangium_transversale_XP_021878206.1
Acanthoeca_sp_MMETSP0105_c1068_g1_i1.p1
Capsaspora_owczarzaki_XP_011270008.1
Chromera_velia_MMETSP0290_c46474_g1_i1.p1
Trichosphaerium_sp_MMETSP0405_c19715_g1_i1.p1
Vexillifera_sp_MMETSP0173_c625_g1_i1.p1
Thecamonas_trahens_XP_013762237.1
Paramecium_tetraurelia_XP_001433865.1
Imantonia_sp_MMETSP1474_c22364_g1_i1.p1
Sphaeroforma_arctica_XP_014160832.1
Paulinella_ovalis_nuclear_SingleCell___SAG5_contig00897.g1112.t1
Piscirickettsiaceae_bacterium_NZRLO1_WP_064004601.1
Legionella_yabuuchiae_WP_133130840.1
Tatlockia_micdadei_WP_045100682.1
0.62

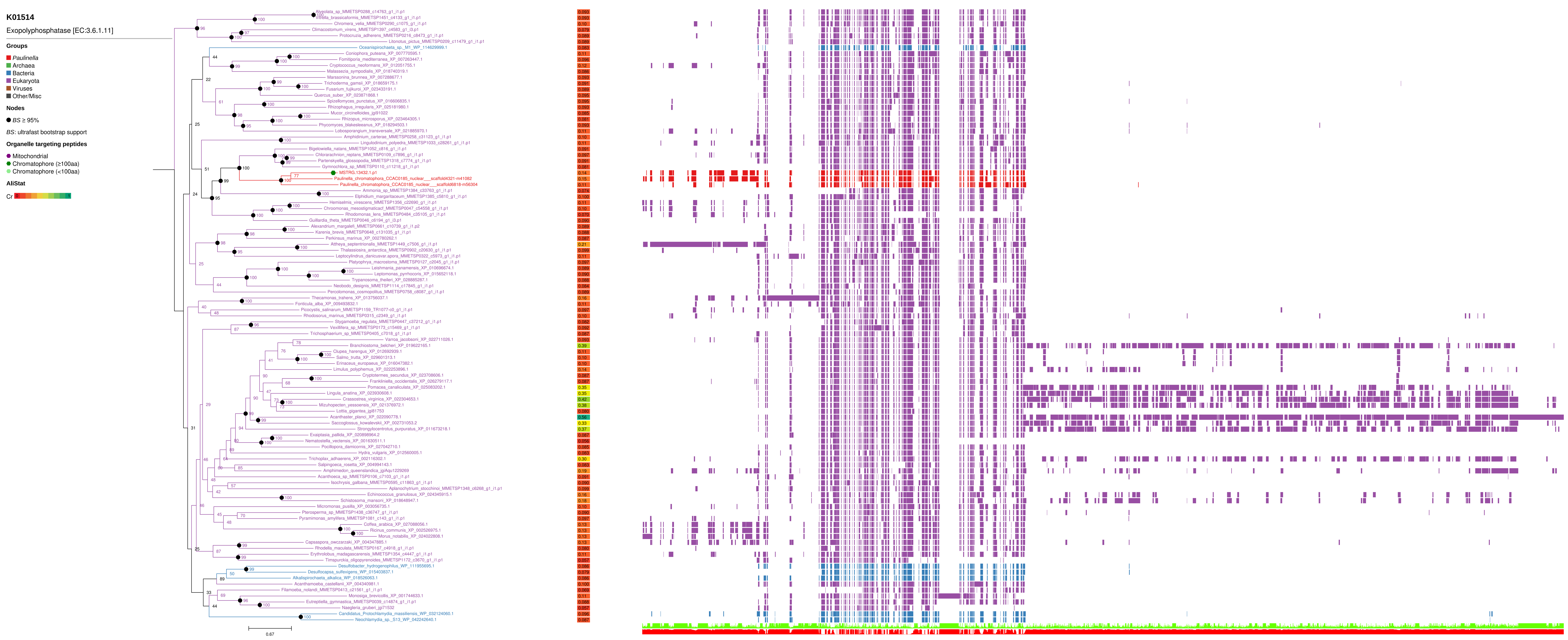

K01514
Exopolyphosphatase [EC:3.6.1.11]
Groups
Paulinella
Archaea
Bacteria
Eukaryota
Viruses
Other/Misc
Nodes
BS ≥ 95%
BS: ultrafast bootstrap support
Organelle targeting peptides
Mitochondrial
Chromatophore (≥100aa)
Chromatophore (<100aa)
AliStat
Cr
0.67

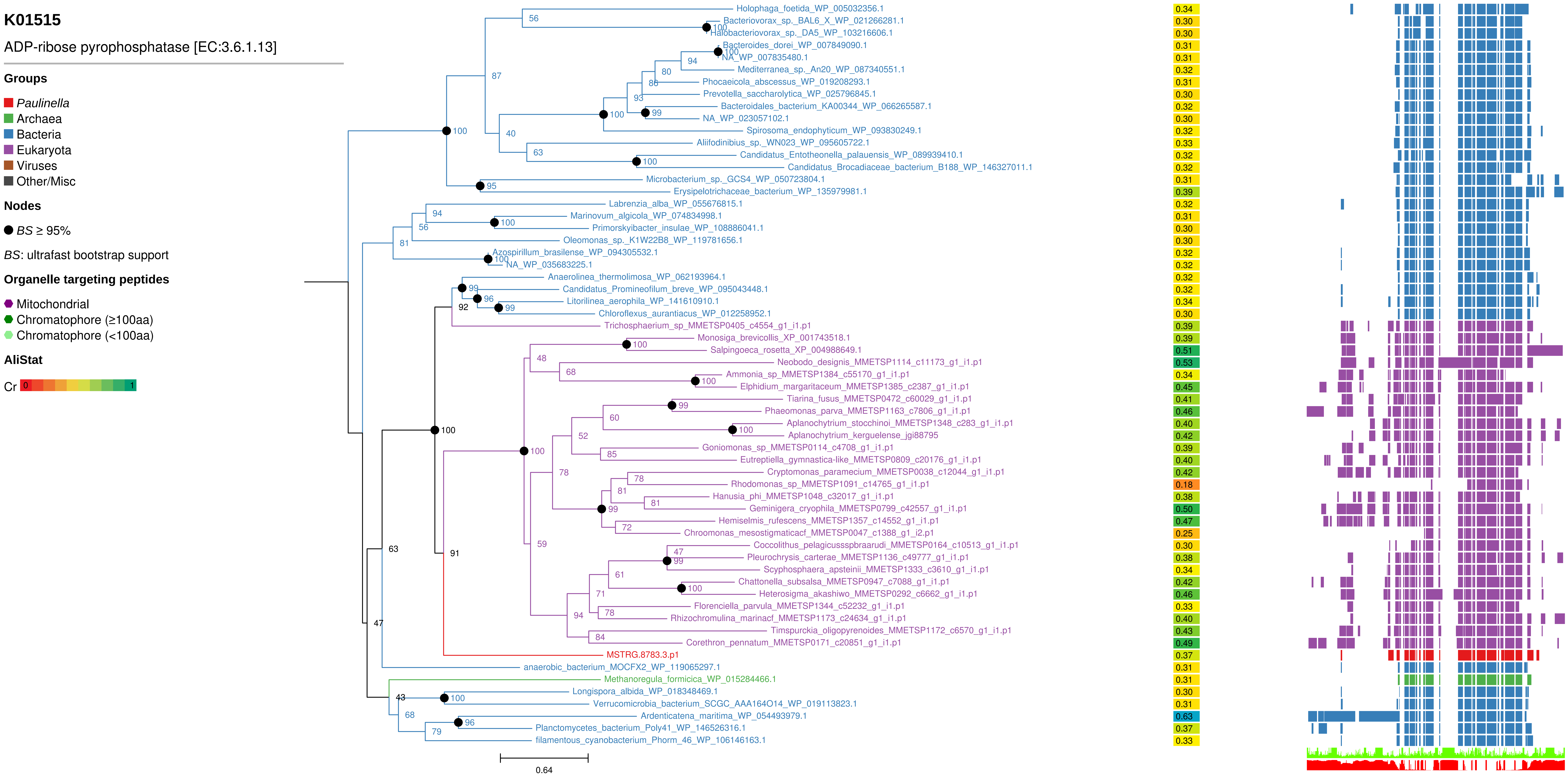

K01515
ADP-ribose pyrophosphatase [EC:3.6.1.13]
Groups
Paulinella
Archaea
Bacteria
Eukaryota
Viruses
Other/Misc
Nodes
BS ≥ 95%
BS: ultrafast bootstrap support
Organelle targeting peptides
Mitochondrial
Chromatophore (≥100aa)
Chromatophore (<100aa)
AliStat
Cr
Holophaga_foetida_WP_005032356.1
Bacteriovorax_sp._BAL6_X_WP_021266281.1
Halobacteriovorax_sp._DA5_WP_103216606.1
Bacteroides_dorei_WP_007849090.1
NA_WP_007835480.1
Mediterranea_sp._An20_WP_087340551.1
Phocaeicola_abscessus_WP_019208293.1
Prevotella_saccharolytica_WP_025796845.1
Bacteroidales_bacterium_KA00344_WP_066265587.1
NA_WP_023057102.1
Spirosoma_endophyticum_WP_093830249.1
Aliifodinibius_sp._WN023_WP_095605722.1
Candidatus_Entotheonella_palauensis_WP_089939410.1
Candidatus_Brocadiaceae_bacterium_B188_WP_146327011.1
Microbacterium_sp._GCS4_WP_050723804.1
Erysipelotrichaceae_bacterium_WP_135979981.1
Labrenzia_alba_WP_055676815.1
Marinovum_algicola_WP_074834998.1
Primorskyibacter_insulae_WP_108886041.1
Oleomonas_sp._K1W22B8_WP_119781656.1
Azospirillum_brasilense_WP_094305532.1
NA_WP_035683225.1
Anaerolinea_thermolimosa_WP_062193964.1
Candidatus_Promineofilum_breve_WP_095043448.1
Litorilinea_aerophila_WP_141610910.1
Chloroflexus_aurantiacus_WP_012258952.1
Trichosphaerium_sp_MMETSP0405_c4554_g1_i1.p1
Monosiga_brevicollis_XP_001743518.1
Salpingoeca_rosetta_XP_004988649.1
Neobodo_designis_MMETSP1114_c11173_g1_i1.p1
Ammonia_sp_MMETSP1384_c55170_g1_i1.p1
Elphidium_margaritaceum_MMETSP1385_c2387_g1_i1.p1
Tiarina_fusus_MMETSP0472_c60029_g1_i1.p1
Phaeomonas_parva_MMETSP1163_c7806_g1_i1.p1
Aplanochytrium_stocchinoi_MMETSP1348_c283_g1_i1.p1
Aplanochytrium_kerguelense_jgi88795
Goniomonas_sp_MMETSP0114_c4708_g1_i1.p1
Eutreptiella_gymnastica-like_MMETSP0809_c20176_g1_i1.p1
Cryptomonas_paramecium_MMETSP0038_c12044_g1_i1.p1
Rhodomonas_sp_MMETSP1091_c14765_g1_i1.p1
Hanusia_phi_MMETSP1048_c32017_g1_i1.p1
Geminigera_cryophila_MMETSP0799_c42557_g1_i1.p1
Hemiselmis_rufescens_MMETSP1357_c14552_g1_i1.p1
Chroomonas_mesostigmaticacf_MMETSP0047_c1388_g1_i2.p1
Coccolithus_pelagicussspbraarudi_MMETSP0164_c10513_g1_i1.p1
Pleurochrysis_carterae_MMETSP1136_c49777_g1_i1.p1
Scyphosphaera_apsteinii_MMETSP1333_c3610_g1_i1.p1
Chattonella_subsalsa_MMETSP0947_c7088_g1_i1.p1
Heterosigma_akashiwo_MMETSP0292_c6662_g1_i1.p1
Florenciella_parvula_MMETSP1344_c52232_g1_i1.p1
Rhizochromulina_marinacf_MMETSP1173_c24634_g1_i1.p1
Timspurckia_oligopyrenoides_MMETSP1172_c6570_g1_i1.p1
Corethron_pennatum_MMETSP0171_c20851_g1_i1.p1
MSTRG.8783.3.p1
anaerobic_bacterium_MOCFX2_WP_119065297.1
Methanoregula_formicica_WP_015284466.1
Longispora_albida_WP_018348469.1
Verrucomicrobia_bacterium_SCGC_AAA164O14_WP_019113823.1
Ardenticatena_maritima_WP_054493979.1
Planctomycetes_bacterium_Poly41_WP_146526316.1
filamentous_cyanobacterium_Phorm_46_WP_106146163.1
0.64

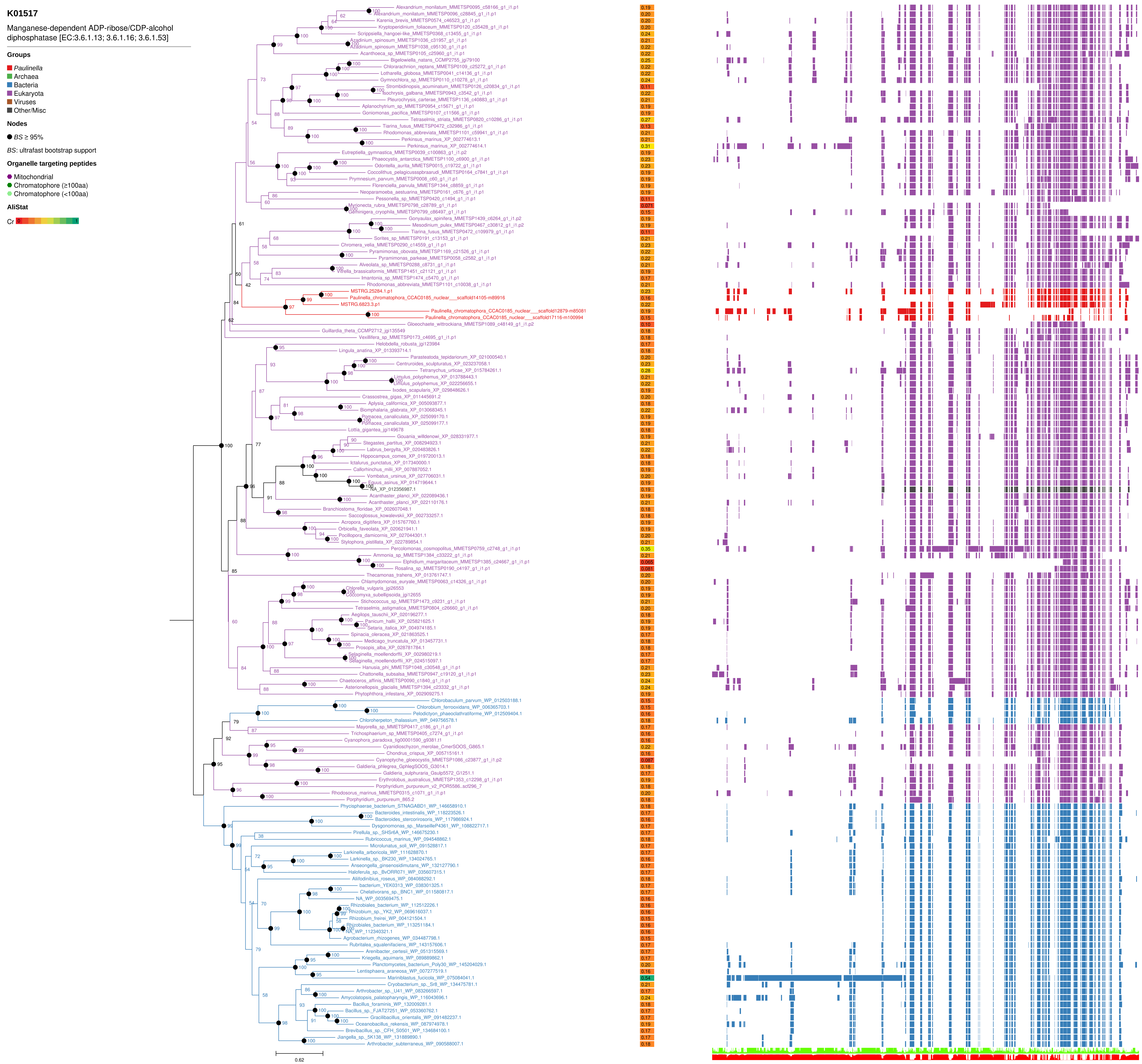

K01517
Manganese-dependent ADP-ribose/CDP-alcohol diphosphatase [EC:3.6.1.13; 3.6.1.16; 3.6.1.53]
Groups
Paulinella
Archaea
Bacteria
Eukaryota
Viruses
Other/Misc
Nodes
BS ≥ 95%
BS: ultrafast bootstrap support
Organelle targeting peptides
Mitochondrial
Chromatophore (≥100aa)
Chromatophore (<100aa)
AliStat
Cr
0.62

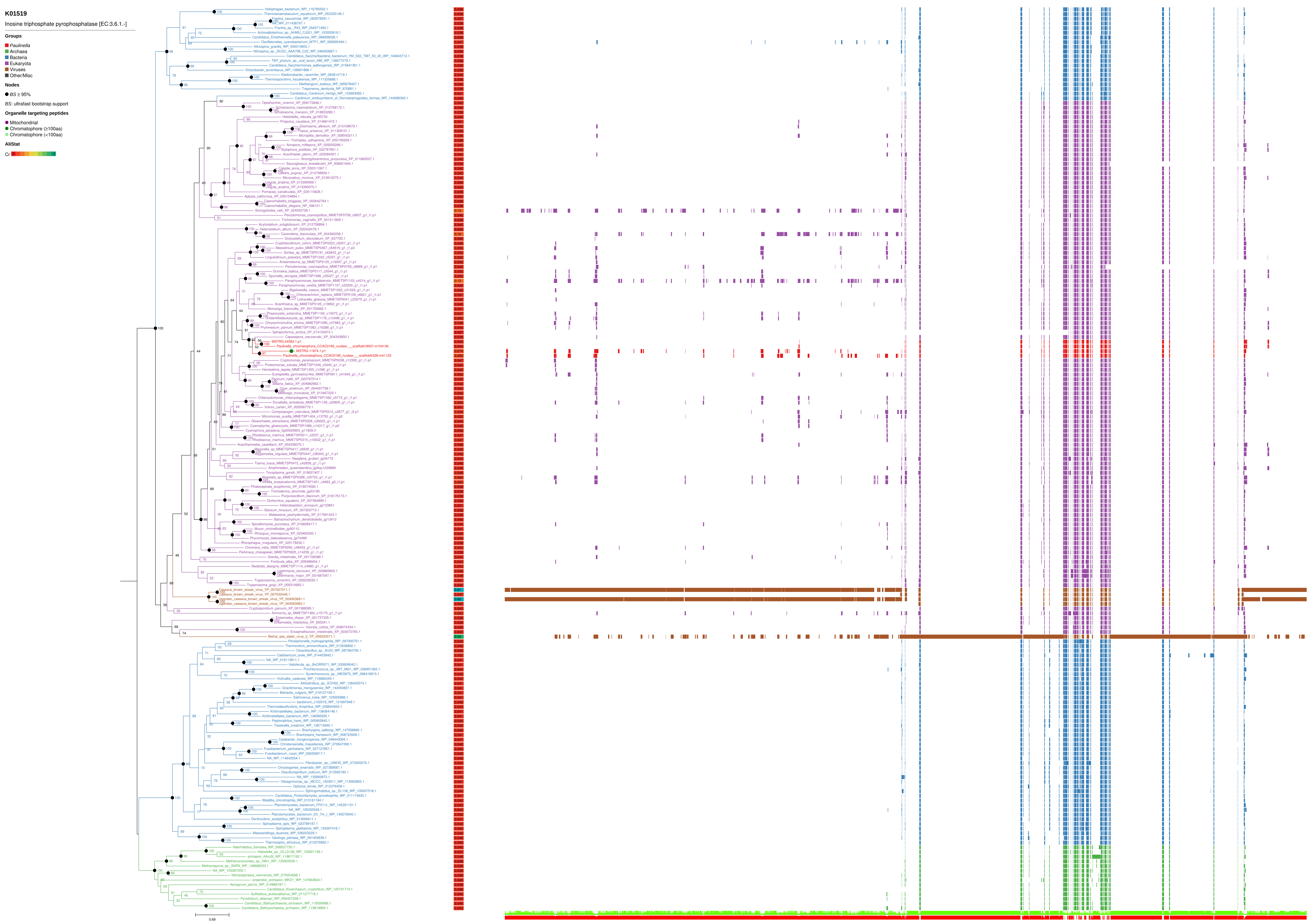
K01519
Inosine triphosphate pyrophosphatase [EC:3.6.1.-]
Groups
Paulinella
Archaea
Bacteria
Eukaryota
Viruses
Other/Misc
Nodes
BS ≥ 95%
BS: ultrafast bootstrap support
Organelle targeting peptides
Mitochondrial
Chromatophore (≥100aa)
Chromatophore (<100aa)
AliStat
Cr
0.69

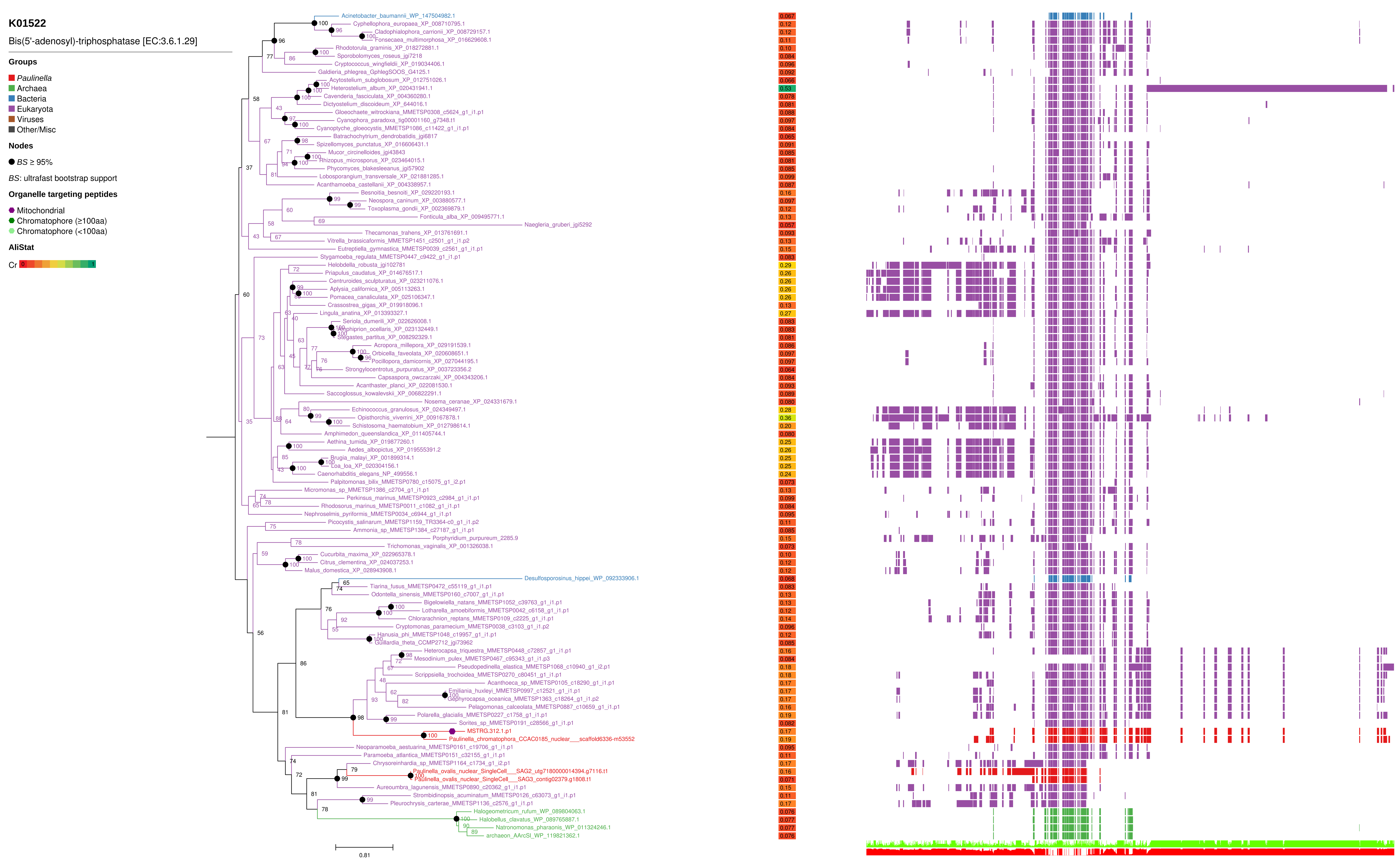
K01522
Bis(5'-adenosyl)-triphosphatase [EC:3.6.1.29]
Groups
Paulinella
Archaea
Bacteria
Eukaryota
Viruses
Other/Misc
Nodes
BS ≥ 95%
BS: ultrafast bootstrap support
Organelle targeting peptides
Mitochondrial
Chromatophore (≥100aa)
Chromatophore (<100aa)
AliStat
Cr
Acinetobacter_baumannii_WP_147504982.1
Cyphellophora_europaea_XP_008710795.1
Cladophialophora_carrionii_XP_008729157.1
Fonsecaea_multimorphosa_XP_016629608.1
Rhodotorula_graminis_XP_018272881.1
Sporobolomyces_roseus_jgi7218
Cryptococcus_wingfieldii_XP_019034406.1
Galdieria_phlegrea_GphlegSOOS_G4125.1
Acytostelium_subglobosum_XP_012751026.1
Heterostelium_album_XP_020431941.1
Cavenderia_fasciculata_XP_004360280.1
Dictyostelium_discoideum_XP_644016.1
Gloeochaete_witrockiana_MMETSP0308_c5624_g1_i1.p1
Cyanophora_paradoxa_tig00001160_g7348.t1
Cyanoptyche_gloeocystis_MMETSP1086_c11422_g1_i1.p1
Batrachochytrium_dendrobatidis_jgi6817
Spizellomyces_punctatus_XP_016606431.1
Mucor_circinelloides_jgi43843
Rhizopus_microsporus_XP_023464015.1
Phycomyces_blakesleeanus_jgi57902
Lobosporangium_transversale_XP_021881285.1
Acanthamoeba_castellanii_XP_004338957.1
Besnoitia_besnoiti_XP_029220193.1
Neospora_caninum_XP_003880577.1
Toxoplasma_gondii_XP_002369879.1
Fonticula_alba_XP_009495771.1
Naegleria_gruberi_jgi5292
Thecamonas_trahens_XP_013761691.1
Vitrella_brassicaformis_MMETSP1451_c2501_g1_i1.p2
Eutreptiella_gymnastica_MMETSP0039_c2561_g1_i1.p1
Stygamoeba_regulata_MMETSP0447_c9422_g1_i1.p1
Helobdella_robusta_jgi102781
Priapulus_caudatus_XP_014676517.1
Centruroides_sculpturatus_XP_023211076.1
Aplysia_californica_XP_005113263.1
Pomacea_canaliculata_XP_025106347.1
Crassostrea_gigas_XP_019918096.1
Lingula_anatina_XP_013393327.1
Seriola_dumerili_XP_022626008.1
Amphiprion_ocellaris_XP_023132449.1
Stegastes_partitus_XP_008292329.1
Acropora_millepora_XP_029191539.1
Orbicella_faveolata_XP_020608651.1
Pocillopora_damicornis_XP_027044195.1
Strongylocentrotus_purpuratus_XP_003723356.2
Capsaspora_owczarzaki_XP_004343206.1
Acanthaster_planci_XP_022081530.1
Saccoglossus_kowalevskii_XP_006822291.1
Nosema_ceranae_XP_024331679.1
Echinococcus_granulosus_XP_024349497.1
Opisthorchis_viverrini_XP_009167878.1
Schistosoma_haematobium_XP_012798614.1
Amphimedon_queenslandica_XP_011405744.1
Aethina_tumida_XP_019877260.1
Aedes_albopictus_XP_019555391.2
Brugia_malayi_XP_001899314.1
Loa_loa_XP_020304156.1
Caenorhabditis_elegans_NP_499556.1
Palpitomonas_bilix_MMETSP0780_c15075_g1_i2.p1
Micromonas_sp_MMETSP1386_c2704_g1_i1.p1
Perkinsus_marinus_MMETSP0923_c2984_g1_i1.p1
Rhodosorus_marinus_MMETSP0011_c1082_g1_i1.p1
Nephroselmis_pyriformis_MMETSP0034_c6944_g1_i1.p1
Picocystis_salinarum_MMETSP1159_TR3364-c0_g1_i1.p2
Ammonia_sp_MMETSP1384_c27187_g1_i1.p1
Porphyridium_purpureum_2285.9
Trichomonas_vaginalis_XP_001326038.1
Cucurbita_maxima_XP_022965378.1
Citrus_clementina_XP_024037253.1
Malus_domestica_XP_028943908.1
Desulfosporosinus_hippei_WP_092333906.1
Tiarina_fusus_MMETSP0472_c55119_g1_i1.p1
Odontella_sinensis_MMETSP0160_c7007_g1_i1.p1
Bigelowiella_natans_MMETSP1052_c39763_g1_i1.p1
Lotharella_amoebiformis_MMETSP0042_c6158_g1_i1.p1
Chlorarachnion_reptans_MMETSP0109_c2225_g1_i1.p1
Cryptomonas_paramecium_MMETSP0038_c3103_g1_i1.p2
Hanusia_phi_MMETSP1048_c19957_g1_i1.p1
Guillardia_theta_CCMP2712_jgi73962
Heterocapsa_triquestra_MMETSP0448_c72857_g1_i1.p1
Mesodinium_pulex_MMETSP0467_c95343_g1_i1.p3
Pseudopedinella_elastica_MMETSP1068_c10940_g1_i2.p1
Scrippsiella_trochoidea_MMETSP0270_c80451_g1_i1.p1
Acanthoeca_sp_MMETSP0105_c18290_g1_i1.p1
Emiliania_huxleyi_MMETSP0997_c12521_g1_i1.p1
Gephyrocapsa_oceanica_MMETSP1363_c18264_g1_i1.p2
Pelagomonas_calceolata_MMETSP0887_c10659_g1_i1.p1
Polarella_glacialis_MMETSP0227_c1758_g1_i1.p1
Sorites_sp_MMETSP0191_c28566_g1_i1.p1
MSTRG.312.1.p1
Paulinella_chromatophora_CCAC0185_nuclear___scaffold6336-m53552
Neoparamoeba_aestuarina_MMETSP0161_c19706_g1_i1.p1
Paramoeba_atlantica_MMETSP0151_c32155_g1_i1.p1
Chrysoreinhardia_sp_MMETSP1164_c1734_g1_i2.p1
Paulinella_ovalis_nuclear_SingleCell___SAG2_utg7180000014394.g7116.t1
Paulinella_ovalis_nuclear_SingleCell___SAG3_contig02379.g1808.t1
Aureoumbra_lagunensis_MMETSP0890_c20362_g1_i1.p1
Strombidinopsis_acuminatum_MMETSP0126_c63073_g1_i1.p1
Pleurochrysis_carterae_MMETSP1136_c2576_g1_i1.p1
Halogeometricum_rufum_WP_089804063.1
Halobellus_clavatus_WP_089765887.1
Natronomonas_pharaonis_WP_011324246.1
archaeon_AArcSl_WP_119821362.1
0.81

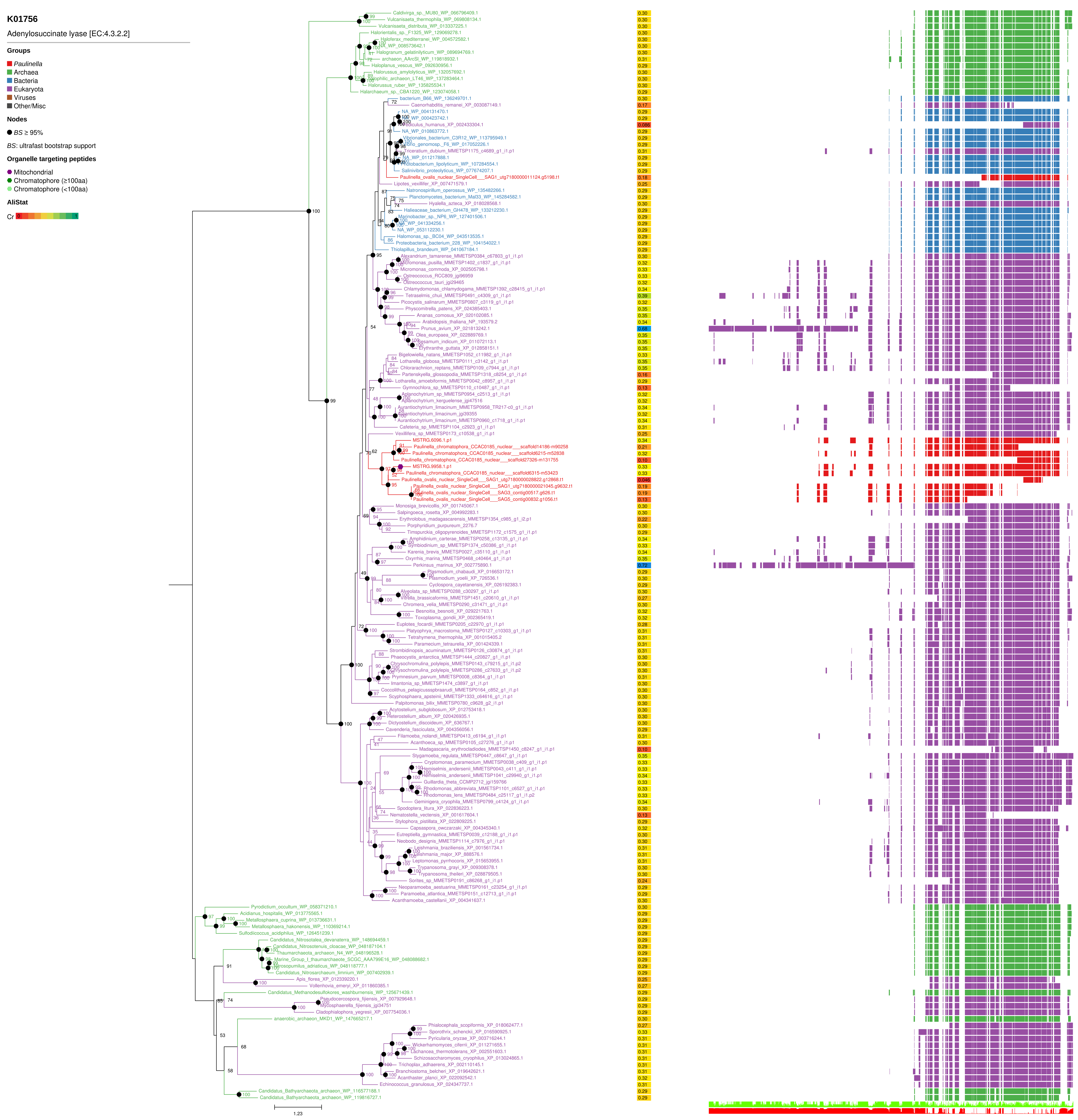

K01756
Adenylosuccinate lyase [EC:4.3.2.2]
Groups
Paulinella
Archaea
Bacteria
Eukaryota
Viruses
Other/Misc
Nodes
BS ≥ 95%
BS: ultrafast bootstrap support
Organelle targeting peptides
Mitochondrial
Chromatophore (≥100aa)
Chromatophore (<100aa)
AliStat
Cr
0
1
Caldivirga_sp._MU80_WP_066796409.1
Vulcanisaeta_thermophila_WP_069808134.1
Vulcanisaeta_distributa_WP_013337225.1
Halorientalis_sp._F1325_WP_129069278.1
Haloferax_mediterranei_WP_004572582.1
NA_WP_008573642.1
Halogranum_gelatinilyticum_WP_089694769.1
archaeon_AArcSl_WP_119818932.1
Haloplanus_vescus_WP_092630956.1
Halorussus_amylolyticus_WP_132057692.1
halophilic_archaeon_LT46_WP_137283464.1
Halorussus_ruber_WP_135825534.1
Halarchaeum_sp._CBA1220_WP_123074058.1
bacterium_B66_WP_136249701.1
Caenorhabditis_remanei_XP_003087149.1
NA_WP_004131470.1
NA_WP_000423742.1
Pediculus_humanus_XP_002433304.1
NA_WP_010863772.1
Vibrionales_bacterium_C3R12_WP_113795949.1
Vibrio_genomosp._F6_WP_017052226.1
Triceratium_dubium_MMETSP1175_c4689_g1_i1.p1
NA_WP_011217888.1
Photobacterium_lipolyticum_WP_107284554.1
Salinivibrio_proteolyticus_WP_077674207.1
Paulinella_ovalis_nuclear_SingleCell___SAG1_utg7180000011124.g5198.t1
Lipotes_vexillifer_XP_007471579.1
Natronospirillum_operosus_WP_135482266.1
Planctomycetes_bacterium_Mal33_WP_145284582.1
Hyalella_azteca_XP_018028568.1
Halieaceae_bacterium_GH478_WP_133212230.1
Marinobacter_sp._NP6_WP_127401506.1
NA_WP_041334256.1
NA_WP_053112230.1
Halomonas_sp._BC04_WP_043513535.1
Proteobacteria_bacterium_228_WP_104154022.1
Thiolapillus_brandeum_WP_041067184.1
Alexandrium_tamarense_MMETSP0384_c67803_g1_i1.p1
Micromonas_pusilla_MMETSP1402_c1837_g1_i1.p1
Micromonas_commoda_XP_002505798.1
Ostreococcus_RCC809_jgi96959
Ostreococcus_tauri_jgi29465
Chlamydomonas_chlamydogama_MMETSP1392_c28415_g1_i1.p1
Tetraselmis_chuii_MMETSP0491_c4309_g1_i1.p1
Picocystis_salinarum_MMETSP0807_c3119_g1_i1.p1
Physcomitrella_patens_XP_024385403.1
Ananas_comosus_XP_020102085.1
Arabidopsis_thaliana_NP_193579.2
Prunus_avium_XP_021813242.1
Olea_europaea_XP_022889769.1
Sesamum_indicum_XP_011072113.1
Erythranthe_guttata_XP_012858151.1
Bigelowiella_natans_MMETSP1052_c11982_g1_i1.p1
Lotharella_globosa_MMETSP0111_c3142_g1_i1.p1
Chlorarachnion_reptans_MMETSP0109_c7944_g1_i1.p1
Partenskyella_glossopodia_MMETSP1318_c8254_g1_i1.p1
Lotharella_amoebiformis_MMETSP0042_c8957_g1_i1.p1
Gymnochlora_sp_MMETSP0110_c10487_g1_i1.p1
Aplanochytrium_sp_MMETSP0954_c2513_g1_i1.p1
Aplanochytrium_kerguelense_jgi47516
Aurantiochytrium_limacinum_MMETSP0958_TR217-c0_g1_i1.p1
Aurantiochytrium_limacinum_jgi39355
Aurantiochytrium_limacinum_MMETSP0960_c1718_g1_i1.p1
Cafeteria_sp_MMETSP1104_c2923_g1_i1.p1
Vexillifera_sp_MMETSP0173_c10538_g1_i1.p1
MSTRG.6096.1.p1
Paulinella_chromatophora_CCAC0185_nuclear___scaffold14186-m90258
Paulinella_chromatophora_CCAC0185_nuclear___scaffold6215-m52838
Paulinella_chromatophora_CCAC0185_nuclear___scaffold27326-m131755
MSTRG.9958.1.p1
Paulinella_chromatophora_CCAC0185_nuclear___scaffold6315-m53423
Paulinella_ovalis_nuclear_SingleCell___SAG1_utg7180000028822.g12868.t1
Paulinella_ovalis_nuclear_SingleCell___SAG1_utg7180000021045.g9632.t1
Paulinella_ovalis_nuclear_SingleCell___SAG3_contig00517.g626.t1
Paulinella_ovalis_nuclear_SingleCell___SAG5_contig00832.g1056.t1
Monosiga_brevicollis_XP_001745067.1
Salpingoeca_rosetta_XP_004992283.1
Erythrolobus_madagascarensis_MMETSP1354_c985_g1_i2.p1
Porphyridium_purpureum_2276.7
Timspurckia_oligopyrenoides_MMETSP1172_c1575_g1_i1.p1
Amphidinium_carterae_MMETSP0258_c13135_g1_i1.p1
Symbiodinium_sp_MMETSP1374_c50386_g1_i1.p1
Karenia_brevis_MMETSP0027_c35110_g1_i1.p1
Oxyrrhis_marina_MMETSP0468_c40464_g1_i1.p1
Perkinsus_marinus_XP_002775890.1
Plasmodium_chabaudi_XP_016653172.1
Plasmodium_yoelii_XP_726536.1
Cyclospora_cayetanensis_XP_026192383.1
Alveolata_sp_MMETSP0288_c30297_g1_i1.p1
Vitrella_brassicaformis_MMETSP1451_c20610_g1_i1.p1
Chromera_velia_MMETSP0290_c31471_g1_i1.p1
Besnoitia_besnoiti_XP_029221763.1
Toxoplasma_gondii_XP_002365419.1
Euplotes_focardii_MMETSP0205_c22970_g1_i1.p1
Platyophrya_macrostoma_MMETSP0127_c10303_g1_i1.p1
Tetrahymena_thermophila_XP_001015405.2
Paramecium_tetraurelia_XP_001424339.1
Strombidinopsis_acuminatum_MMETSP0126_c30874_g1_i1.p1
Phaeocystis_antarctica_MMETSP1444_c20827_g1_i1.p1
Chrysochromulina_polylepis_MMETSP0143_c79215_g1_i1.p2
Chrysochromulina_polylepis_MMETSP0286_c27633_g1_i1.p2
Prymnesium_parvum_MMETSP0008_c8364_g1_i1.p1
Imantonia_sp_MMETSP1474_c3897_g1_i1.p1
Coccolithus_pelagicusssp braarudi_MMETSP0164_c852_g1_i1.p1
Scyphosphaera_apsteinii_MMETSP1333_c64616_g1_i1.p1
Palpitomonas_bilix_MMETSP0780_c9628_g2_i1.p1
Acytostelium_subglobosum_XP_012753418.1
Heterostelium_album_XP_020426935.1
Dictyostelium_discoideum_XP_636767.1
Cavenderia_fasciculata_XP_004356056.1
Filamoeba_nolandi_MMETSP0413_c6194_g1_i1.p1
Acanthoeca_sp_MMETSP0105_c27276_g1_i1.p1
Madagascaria_erythrocladiodes_MMETSP1450_c8247_g1_i1.p1
Stygamoeba_regulata_MMETSP0447_c8647_g1_i1.p1
Cryptomonas_paramecium_MMETSP0038_c409_g1_i1.p1
Hemiselmis_andersenii_MMETSP0043_c411_g1_i1.p1
Hemiselmis_andersenii_MMETSP1041_c29940_g1_i1.p1
Guillardia_theta_CCMP2712_jgi159766
Rhodomonas_abbreviata_MMETSP1101_c6527_g1_i1.p1
Rhodomonas_lens_MMETSP0484_c25117_g1_i1.p2
Geminigera_cryophila_MMETSP0799_c4124_g1_i1.p1
Spodoptera_litura_XP_022836223.1
Nematostella_vectensis_XP_001617604.1
Stylophora_pistillata_XP_022809225.1
Capsaspora_owczarzaki_XP_004345340.1
Eutreptiella_gymnastica_MMETSP0039_c12188_g1_i1.p1
Neobodo_designis_MMETSP1114_c7976_g1_i1.p1
Leishmania_braziliensis_XP_001561734.1
Leishmania_major_XP_888576.1
Leptomonas_pyrrhocoris_XP_015653955.1
Trypanosoma_grayi_XP_009308378.1
Trypanosoma_theileri_XP_028879505.1
Sorites_sp_MMETSP0191_c86268_g1_i1.p1
Neoparamoeba_aestuarina_MMETSP0161_c23254_g1_i1.p1
Paramoeba_atlantica_MMETSP0151_c12713_g1_i1.p1
Acanthamoeba_castellanii_XP_004341637.1
Pyrodictium_occultum_WP_058371210.1
Acidianus_hospitalis_WP_013775565.1
Metallosphaera_cuprina_WP_013736631.1
Metallosphaera_hakonensis_WP_110369214.1
Sulfodiicoccus_acidiphilus_WP_126451239.1
Candidatus_Nitrosotalea_devanaterra_WP_148694459.1
Candidatus_Nitrosotenuis_cloacae_WP_048187104.1
Thaumarchaeota_archaeon_N4_WP_048196528.1
Marine_Group_I_thaumarchaeote_SCGC_AAA799E16_WP_048088682.1
Nitrosopumilus_adriaticus_WP_048118777.1
Candidatus_Nitrosarchaeum_limnium_WP_007402939.1
Apis_florea_XP_012339220.1
Vollenhovia_emeryi_XP_011860385.1
Candidatus_Methanodesulfokores_washburnensis_WP_125671439.1
Pseudocercospora_fijiensis_XP_007929648.1
Mycosphaerella_fijiensis_jgi34751
Cladophialophora_yegresii_XP_007754036.1
anaerobic_archaeon_MKD1_WP_147665217.1
Phialocephala_scopiformis_XP_018062477.1
Sporothrix_schenckii_XP_016590925.1
Pyricularia_oryzae_XP_003716244.1
Wickerhamomyces_ciferrii_XP_011271655.1
Lachancea_thermotolerans_XP_002551603.1
Schizosaccharomyces_cryophilus_XP_013024865.1
Trichoplax_adhaerens_XP_002110145.1
Branchiostoma_belcheri_XP_019642621.1
Acanthaster_planci_XP_022092542.1
Echinococcus_granulosus_XP_024347737.1
Candidatus_Bathyarchaeota_archaeon_WP_116577188.1
Candidatus_Bathyarchaeota_archaeon_WP_119816727.1
1.23

K01768

Adenylate cyclase [EC:4.6.1.1]

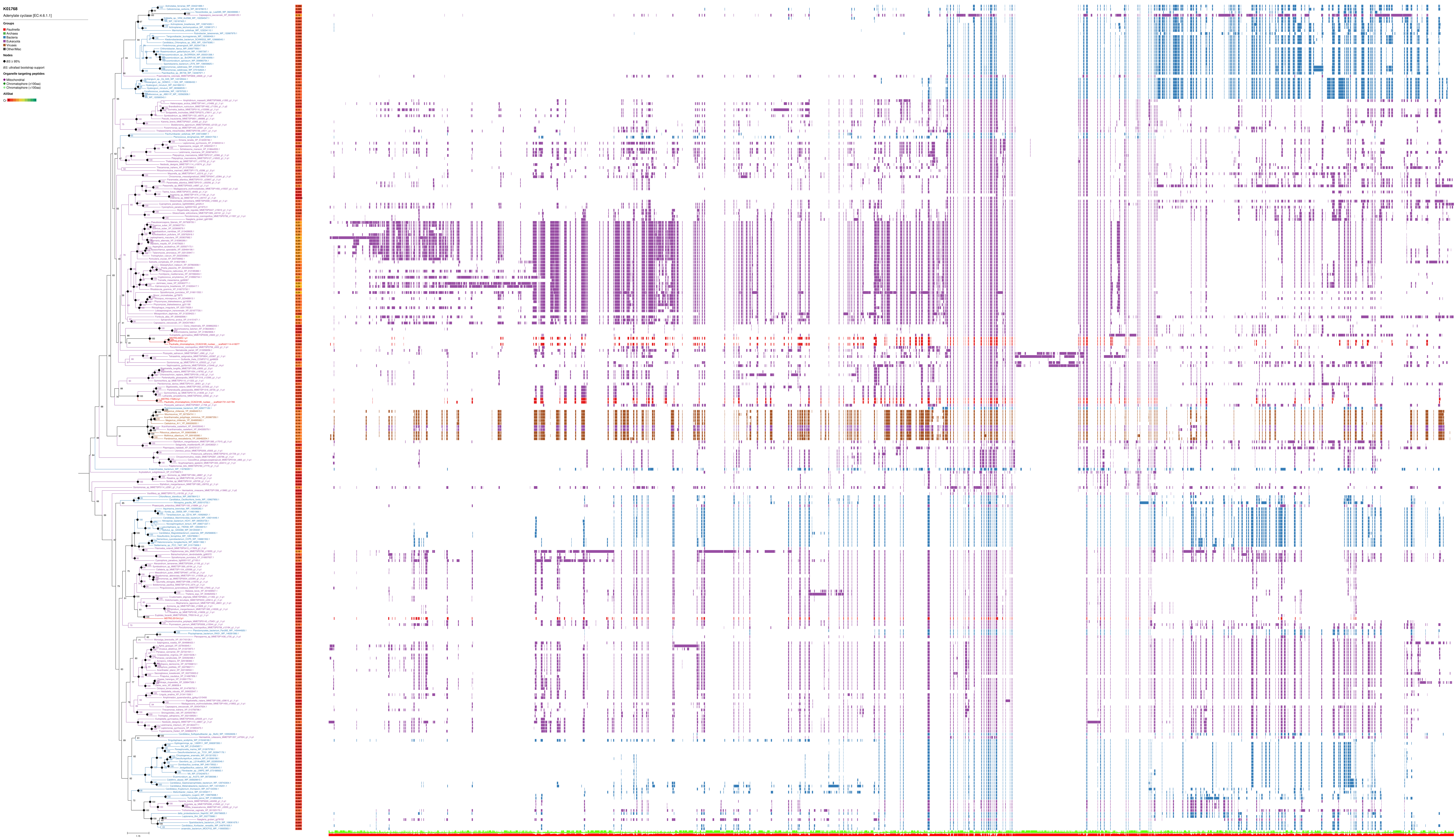

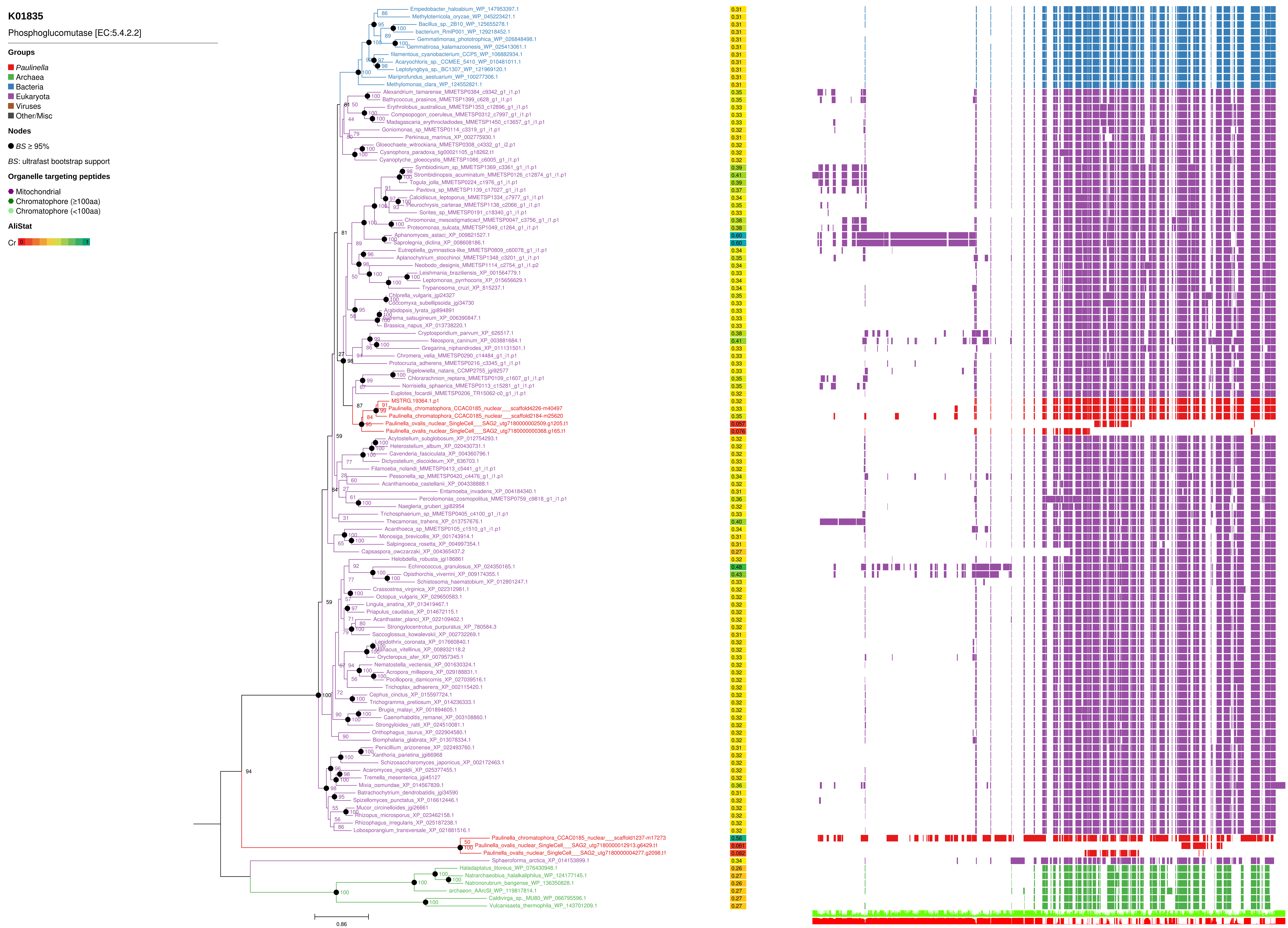
K01835
Phosphoglucomutase [EC:5.4.2.2]
Groups
Paulinella
Archaea
Bacteria
Eukaryota
Viruses
Other/Misc
Nodes
BS ≥ 95%
BS: ultrafast bootstrap support
Organelle targeting peptides
Mitochondrial
Chromatophore (≥100aa)
Chromatophore (<100aa)
AliStat
Cr
0.86

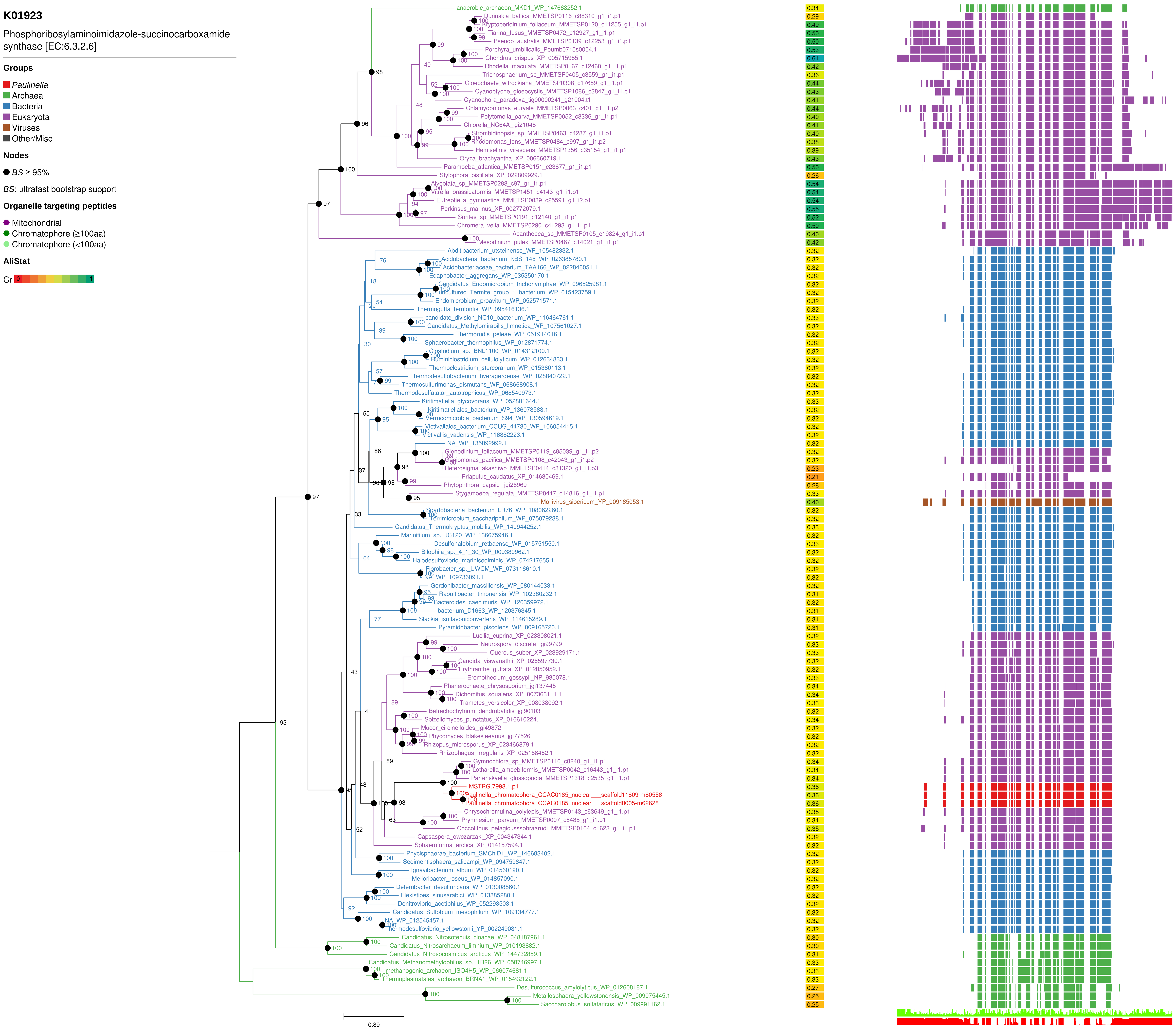

K01923
Phosphoribosylaminoimidazole-succinocarboxamide synthase [EC:6.3.2.6]
Groups
Paulinella
Archaea
Bacteria
Eukaryota
Viruses
Other/Misc
Nodes
BS ≥ 95%
BS: ultrafast bootstrap support
Organelle targeting peptides
Mitochondrial
Chromatophore (≥100aa)
Chromatophore (<100aa)
AliStat
Cr
0
1
anaerobic_archaeon_MKD1_WP_147663252.1
Durinskia_baltica_MMETSP0116_c88310_g1_i1.p1
Kryptoperidinium_foliaceum_MMETSP0120_c11255_g1_i1.p1
Tiarina_fusus_MMETSP0472_c12927_g1_i1.p1
Pseudo_australis_MMETSP0139_c12253_g1_i1.p1
Porphyra_umbilicalis_Poumb0715s0004.1
Chondrus_crispus_XP_005715985.1
Rhodella_maculata_MMETSP0167_c12460_g1_i1.p1
Trichosphaerium_sp_MMETSP0405_c3559_g1_i1.p1
Gloeochaete_witrockiana_MMETSP0308_c17659_g1_i1.p1
Cyanoptyche_gloeocystis_MMETSP1086_c3847_g1_i1.p1
Cyanophora_paradoxa_tig00000241_g21004.t1
Chlamydomonas_euryale_MMETSP0063_c401_g1_i1.p2
Polytomella_parva_MMETSP0052_c8336_g1_i1.p1
Chlorella_NC64A_jgi21048
Strombidinopsis_sp_MMETSP0463_c4287_g1_i1.p1
Rhodomonas_lens_MMETSP0484_c997_g1_i1.p2
Hemiselmis_virescens_MMETSP1356_c35154_g1_i1.p1
Oryza_brachyantha_XP_006660719.1
Paramoeba_atlantica_MMETSP0151_c23877_g1_i1.p1
Stylophora_pistillata_XP_022809929.1
Alveolata_sp_MMETSP0288_c97_g1_i1.p1
Vitrella_brassicaformis_MMETSP1451_c4143_g1_i1.p1
Eutreptiella_gymnastica_MMETSP0039_c25591_g1_i2.p1
Perkinsus_marinus_XP_002772079.1
Sorites_sp_MMETSP0191_c12140_g1_i1.p1
Chromera_velia_MMETSP0290_c41293_g1_i1.p1
Acanthoeca_sp_MMETSP0105_c19824_g1_i1.p1
Mesodinium_pulex_MMETSP0467_c14021_g1_i1.p1
Abditibacterium_utsteinense_WP_105482332.1
Acidobacteria_bacterium_KBS_146_WP_026385780.1
Acidobacteriaceae_bacterium_TAA166_WP_022846051.1
Edaphobacter_aggregans_WP_035350170.1
Candidatus_Endomicrobium_trichonymphae_WP_096525981.1
uncultured_Termite_group_1_bacterium_WP_015423759.1
Endomicrobium_proavitum_WP_052571571.1
Thermogutta_terrifontis_WP_095416136.1
candidate_division_NC10_bacterium_WP_116464761.1
Candidatus_Methylomirabilis_limnetica_WP_107561027.1
Thermorudis_peleae_WP_051914616.1
Sphaerobacter_thermophilus_WP_012871774.1
Clostridium_sp._BNL1100_WP_014312100.1
Ruminiclostridium_cellulolyticum_WP_012634833.1
Thermoclostridium_stercorarium_WP_015360113.1
Thermodesulfobacterium_hveragerdense_WP_028840722.1
Thermosulfurimonas_dismutans_WP_068668908.1
Thermodesulfatator_autotrophicus_WP_068540973.1
Kiritimatiella_glycovorans_WP_052881644.1
Kiritimatiellales_bacterium_WP_136078583.1
Verrucomicrobia_bacterium_S94_WP_130594619.1
Victivallales_bacterium_CCUG_44730_WP_106054415.1
Victivallis_vadensis_WP_116882223.1
NA_WP_135892992.1
Glenodinium_foliaceum_MMETSP0119_c85039_g1_i1.p2
Gephyromonas_pacifica_MMETSP0108_c42043_g1_i1.p2
Heterosigma_akashiwo_MMETSP0414_c31320_g1_i1.p3
Priapulus_caudatus_XP_014680469.1
Phytophthora_capsici_jgi26969
Stygamoeba_regulata_MMETSP0447_c14816_g1_i1.p1
Mollivirus_sibericum_YP_009165053.1
Spartobacteria_bacterium_LR76_WP_108062260.1
Terrimicrobium_sacchariphilum_WP_075079238.1
Candidatus_Thermokryptus_mobilis_WP_140944252.1
Marinifilum_sp._JC120_WP_136675946.1
Desulfohalobium_retbaense_WP_015751550.1
Bilophila_sp._4_1_30_WP_009380962.1
Halodesulfovibrio_marinisediminis_WP_074217655.1
Fibrobacter_sp._UWCM_WP_073116610.1
NA_WP_109736091.1
Gordonibacter_massiliensis_WP_080144033.1
Raoultibacter_timonensis_WP_102380232.1
Bacteroides_caecimuris_WP_120359972.1
bacterium_D1663_WP_120376345.1
Slackia_isoflavoniconvertens_WP_114615289.1
Pyramidobacter_piscolens_WP_009165720.1
Lucilia_cuprina_XP_023308021.1
Neurospora_discreta_jgi99799
Quercus_suber_XP_023929171.1
Candida_viswanathii_XP_026597730.1
Erythranthe_guttata_XP_012850952.1
Eremothecium_gossypii_NP_985078.1
Phanerochaete_chrysosporium_jgi137445
Dichomitus_squalens_XP_007363111.1
Trametes_versicolor_XP_008038092.1
Batrachochytrium_dendrobatidis_jgi90103
Spizellomyces_punctatus_XP_016610224.1
Mucor_circinelloides_jgi49872
Phycomyces_blakesleeanus_jgi77526
Rhizopus_microsporus_XP_023466879.1
Rhizophagus_irregularis_XP_025168452.1
Gymnochlora_sp_MMETSP0110_c8240_g1_i1.p1
Lotharella_amoebiformis_MMETSP0042_c16443_g1_i1.p1
Partenskyella_glossopodia_MMETSP1318_c2535_g1_i1.p1
MSTRG.7998.1.p1
Paulinella_chromatophora_CCAC0185_nuclear___scaffold11809-m80556
Paulinella_chromatophora_CCAC0185_nuclear___scaffold8005-m62628
Chrysochromulina_polylepis_MMETSP0143_c63649_g1_i1.p1
Prymnesium_parvum_MMETSP0007_c5485_g1_i1.p1
Coccolithus_pelagicussspbraarudi_MMETSP0164_c1623_g1_i1.p1
Capsaspora_owczarzaki_XP_004347344.1
Sphaeroforma_arctica_XP_014157594.1
Phycisphaerae_bacterium_SMChiD1_WP_146683402.1
Sedimentisphaera_salicampi_WP_094759847.1
Ignavibacterium_album_WP_014560190.1
Melioribacter_roseus_WP_014857090.1
Deferribacter_desulfuricans_WP_013008560.1
Flexistipes_sinusarabici_WP_013885280.1
Denitrovibrio_acetiphilus_WP_052293503.1
Candidatus_Sulfobium_mesophilum_WP_109134777.1
NA_WP_012545457.1
Thermodesulfovibrio_yellowstonii_YP_002249081.1
Candidatus_Nitrosotenuis_cloacae_WP_048187961.1
Candidatus_Nitrosarchaeum_limnium_WP_010193882.1
Candidatus_Nitrosocosmicus_arcticus_WP_144732859.1
Candidatus_Methanomethylophilus_sp._1R26_WP_058746997.1
methanogenic_archaeon_ISO4H5_WP_066074681.1
Thermoplasmatales_archaeon_BRNA1_WP_015492122.1
Desulfurococcus_amylolyticus_WP_012608187.1
Metallosphaera_yellowstonensis_WP_009075445.1
Saccharolobus_solfataricus_WP_009991162.1
0.89

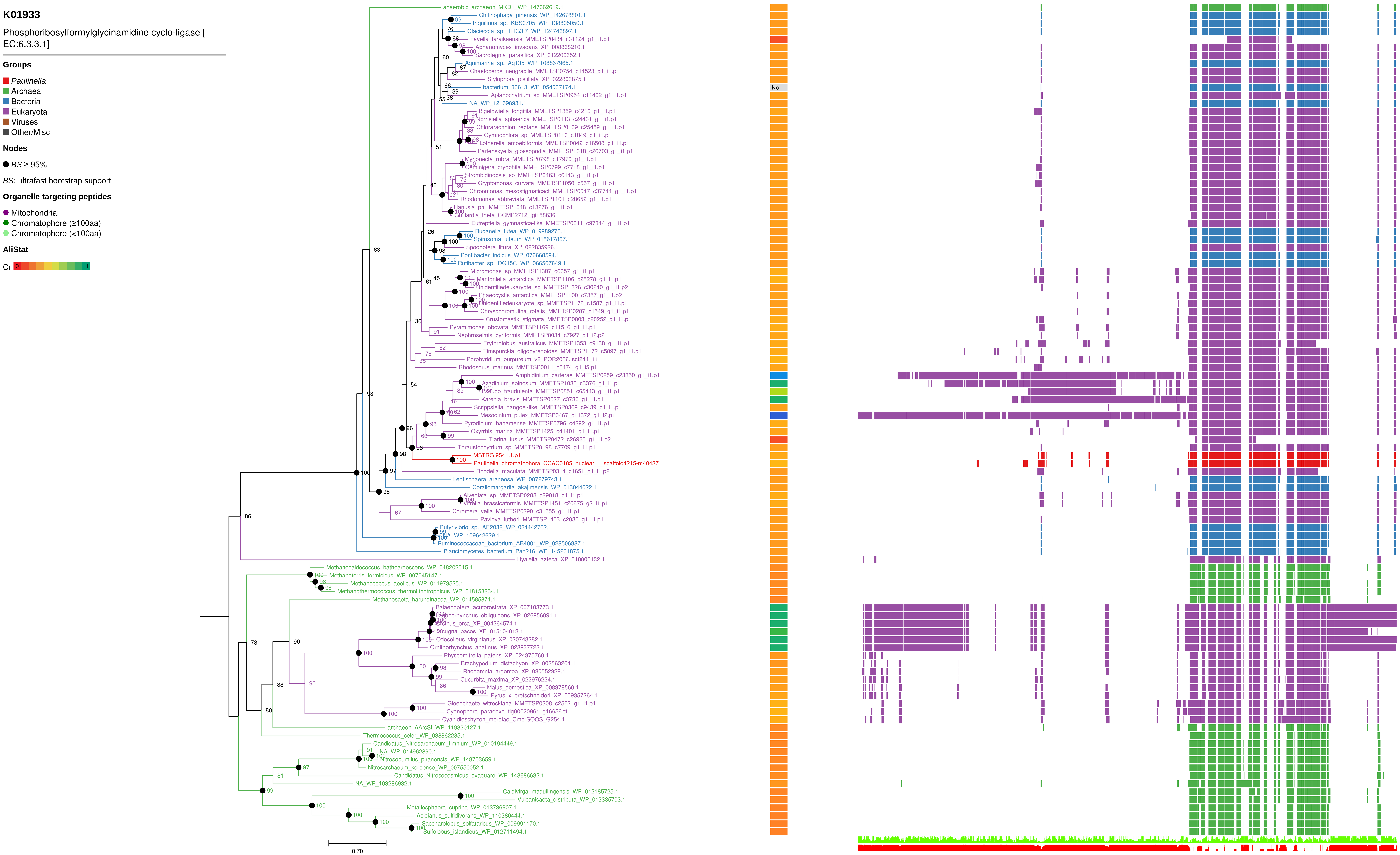
K01933
Phosphoribosylformylglycinamidine cyclo-ligase [EC:6.3.3.1]
Groups
Paulinella
Archaea
Bacteria
Eukaryota
Viruses
Other/Misc
Nodes
BS ≥ 95%
BS: ultrafast bootstrap support
Organelle targeting peptides
Mitochondrial
Chromatophore (≥100aa)
Chromatophore (<100aa)
AliStat
Cr 0 1
anaerobic_archaeon_MKD1_WP_147662619.1
Chitinophaga_pinensis_WP_142678801.1
Inquilinus_sp._KBS0705_WP_138805050.1
Glaciecola_sp._THG3.7_WP_124746897.1
Favella_taraikaensis_MMETSP0434_c31124_g1_i1.p1
Aphanomyces_invadans_XP_008868210.1
Saprolegnia_parasitica_XP_012200652.1
Aquimarina_sp._Aq135_WP_108867965.1
Chaetoceros_neogracile_MMETSP0754_c14523_g1_i1.p1
Stylophora_pistillata_XP_022803875.1
bacterium_336_3_WP_054037174.1
Aplanochytrium_sp_MMETSP0954_c11402_g1_i1.p1
NA_WP_121698931.1
Bigelowiella_longifila_MMETSP1359_c4210_g1_i1.p1
Norrisiella_sphaerica_MMETSP0113_c24431_g1_i1.p1
Chlorarachnion_reptans_MMETSP0109_c25489_g1_i1.p1
Gymnochlora_sp_MMETSP0110_c1849_g1_i1.p1
Lotharella_amoebiformis_MMETSP0042_c16508_g1_i1.p1
Partenskyella_glossopodia_MMETSP1318_c26703_g1_i1.p1
Myrionecta_rubra_MMETSP0798_c17970_g1_i1.p1
Geminigera_cryophila_MMETSP0799_c7718_g1_i1.p1
Strombidinopsis_sp_MMETSP0463_c6143_g1_i1.p1
Cryptomonas_curvata_MMETSP1050_c557_g1_i1.p1
Chroomonas_mesostigmaticacf_MMETSP0047_c37744_g1_i1.p1
Rhodomonas_abbreviata_MMETSP1101_c28652_g1_i1.p1
Hanusia_phi_MMETSP1048_c13276_g1_i1.p1
Guillardia_theta_CCMP2712_jgi158636
Eutreptiella_gymnastica-like_MMETSP0811_c97344_g1_i1.p1
Rudanella_lutea_WP_019989276.1
Spirosoma_luteum_WP_018617867.1
Spodoptera_litura_XP_022835926.1
Pontibacter_indicus_WP_076668594.1
Rufibacter_sp._DG15C_WP_066507649.1
Micromonas_sp_MMETSP1387_c6057_g1_i1.p1
Mantoniella_antarctica_MMETSP1106_c28278_g1_i1.p1
Unidentifiedeukaryote_sp_MMETSP1326_c30240_g1_i1.p2
Phaeocystis_antarctica_MMETSP1100_c7357_g1_i1.p2
Unidentifiedeukaryote_sp_MMETSP1178_c1587_g1_i1.p1
Chrysochromulina_rotalis_MMETSP0287_c1549_g1_i1.p1
Crustomastix_stigmata_MMETSP0803_c20252_g1_i1.p1
Pyramimonas_obovata_MMETSP1169_c11516_g1_i1.p1
Nephroselmis_pyriformis_MMETSP0034_c7927_g1_i2.p2
Erythrolobus_australicus_MMETSP1353_c9138_g1_i1.p1
Timspurckia_oligopyrenoides_MMETSP1172_c5897_g1_i1.p1
Porphyridium_purpureum_v2_POR2056..scf244_11
Rhodosorus_marinus_MMETSP0011_c6474_g1_i5.p1
Amphidinium_carterae_MMETSP0259_c23350_g1_i1.p1
Azadinium_spinosum_MMETSP1036_c3376_g1_i1.p1
Pseudo_fraudulenta_MMETSP0851_c65443_g1_i1.p1
Karenia_brevis_MMETSP0527_c3730_g1_i1.p1
Scrippsiella_hangoei-like_MMETSP0369_c9439_g1_i1.p1
Mesodinium_pulex_MMETSP0467_c11372_g1_i2.p1
Pyrodinium_bahamense_MMETSP0796_c4292_g1_i1.p1
Oxyrrhis_marina_MMETSP1425_c41401_g1_i1.p1
Tiarina_fusus_MMETSP0472_c26920_g1_i1.p2
Thraustochytrium_sp_MMETSP0198_c7709_g1_i1.p1
MSTRG.9541.1.p1
Paulinella_chromatophora_CCAC0185_nuclear___scaffold4215-m40437
Rhodella_maculata_MMETSP0314_c1651_g1_i1.p2
Lentisphaera_araneosa_WP_007279743.1
Coraliomargarita_akajimensis_WP_013044022.1
Alveolata_sp_MMETSP0288_c29818_g1_i1.p1
Vitrella_brassicaformis_MMETSP1451_c20675_g2_i1.p1
Chromera_velia_MMETSP0290_c31555_g1_i1.p1
Pavlova_lutheri_MMETSP1463_c2080_g1_i1.p1
Butyrivibrio_sp._AE2032_WP_034442762.1
NA_WP_109642629.1
Ruminococcaceae_bacterium_AB4001_WP_028506887.1
Planctomycetes_bacterium_Pan216_WP_145261875.1
Hyalella_azteca_XP_018006132.1
Methanocaldococcus_bathoardescens_WP_048202515.1
Methanotorris_formicicus_WP_007045147.1
Methanococcus_aeolicus_WP_011973525.1
Methanothermococcus_thermolithotrophicus_WP_018153234.1
Methanosaeta_harundinacea_WP_014585871.1
Balaenoptera_acutorostrata_XP_007183773.1
Lagenorhynchus_obliquidens_XP_026956891.1
Orcinus_orca_XP_004264574.1
Vicugna_pacos_XP_015104813.1
Odocoileus_virginianus_XP_020748282.1
Ornithorhynchus_anatinus_XP_028937723.1
Physcomitrella_patens_XP_024375760.1
Brachypodium_distachyon_XP_003563204.1
Rhodamnia_argentea_XP_030552928.1
Cucurbita_maxima_XP_022976224.1
Malus_domestica_XP_008378560.1
Pyrus_x_bretschneideri_XP_009357264.1
Gloeochaete_witrockiana_MMETSP0308_c2562_g1_i1.p1
Cyanophora_paradoxa_tig00020961_g16656.t1
Cyanidioschyzon_merolae_CmerSOOS_G254.1
archaeon_AArcSl_WP_119820127.1
Thermococcus_celer_WP_088862285.1
Candidatus_Nitrosarchaeum_limnium_WP_010194449.1
NA_WP_014962890.1
Nitrosopumilus_piranensis_WP_148703659.1
Nitrosarchaeum_koreense_WP_007550052.1
Candidatus_Nitrosocosmicus_exaquare_WP_148686682.1
NA_WP_103286932.1
Caldivirga_maquilingensis_WP_012185725.1
Vulcanisaeta_distributa_WP_013335703.1
Metallosphaera_cuprina_WP_013736907.1
Acidianus_sulfidivorans_WP_110380444.1
Saccharolobus_solfataricus_WP_009991170.1
Sulfolobus_islandicus_WP_012711494.1
No
0.70

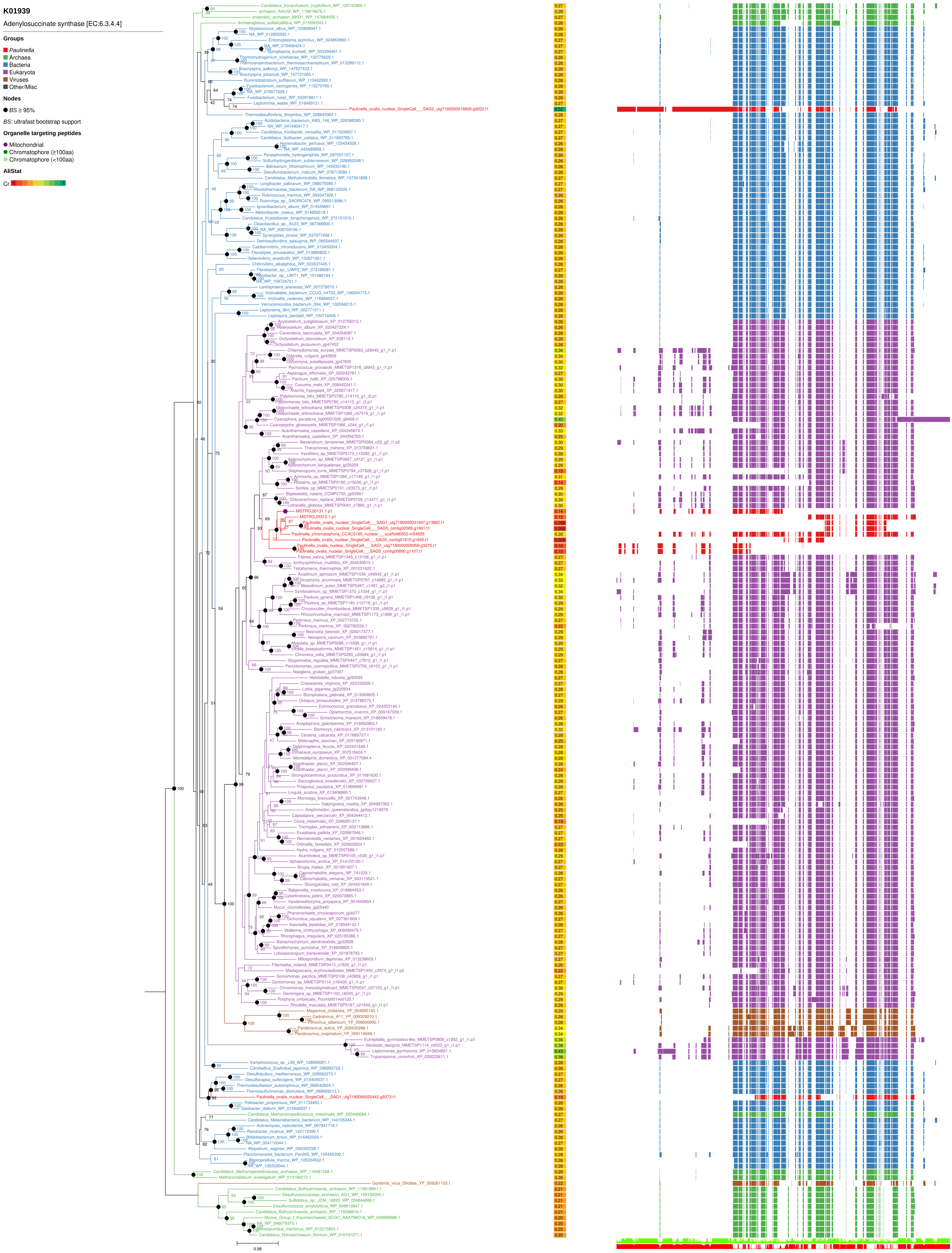
K01939
Adenylosuccinate synthase [EC:6.3.4.4]
Groups
Paulinella
Archaea
Bacteria
Eukaryota
Viruses
Other/Misc
Nodes
BS ≥ 95%
BS: ultrafast bootstrap support
Organelle targeting peptides
Mitochondrial
Chromatophore (≥100aa)
Chromatophore (<100aa)
AliStat
Cr
0
1
0.98

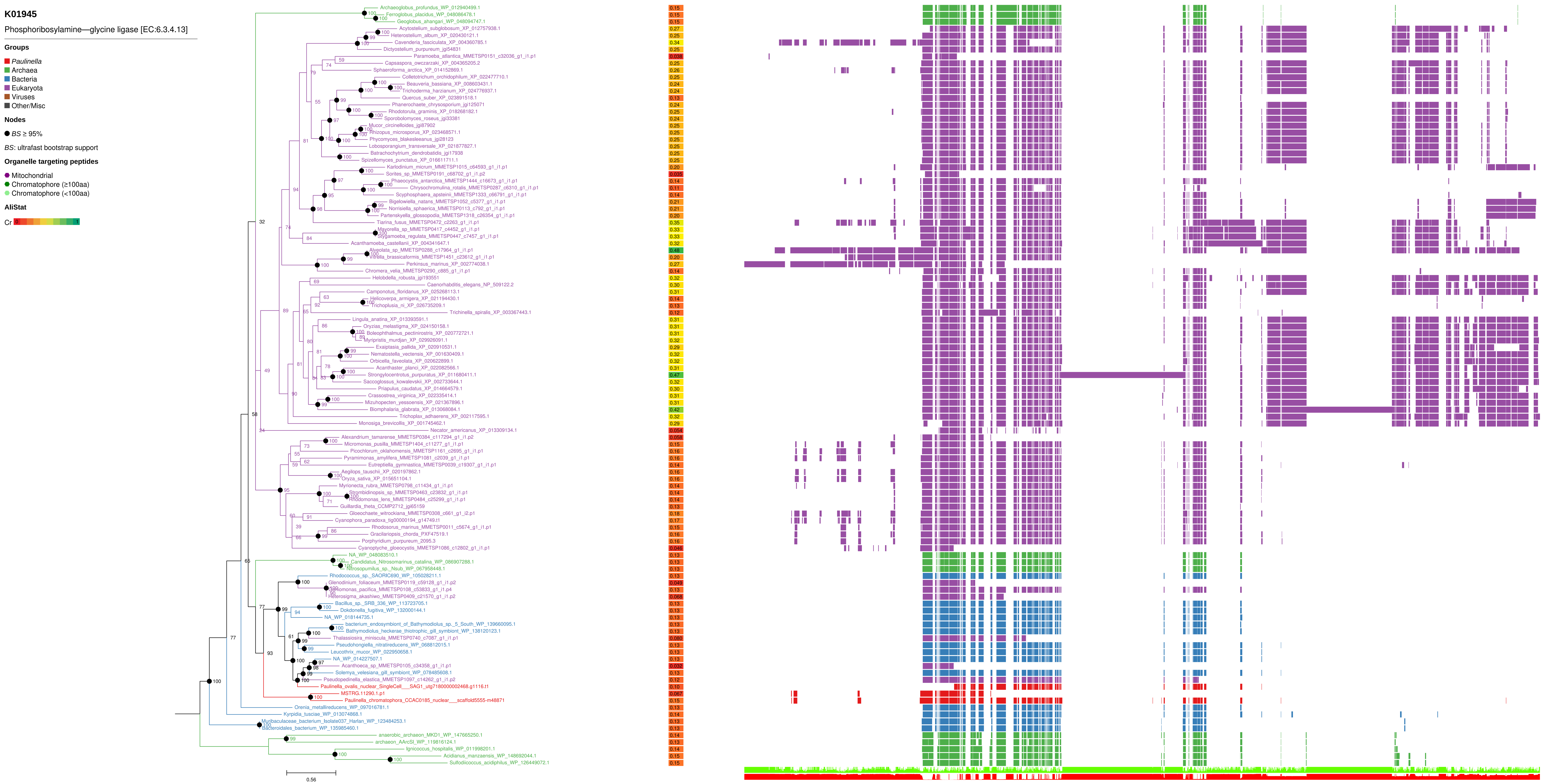
K01945
Phosphoribosylamine—glycine ligase [EC:6.3.4.13]
Groups
Paulinella
Archaea
Bacteria
Eukaryota
Viruses
Other/Misc
Nodes
BS ≥ 95%
BS: ultrafast bootstrap support
Organelle targeting peptides
Mitochondrial
Chromatophore (≥100aa)
Chromatophore (<100aa)
AliStat
Cr
Archaeoglobus_profundus_WP_012940499.1
Ferroglobus_placidus_WP_048086478.1
Geoglobus_ahangari_WP_048094747.1
Acytostelium_subglobosum_XP_012757938.1
Heterostelium_album_XP_020430121.1
Cavenderia_fasciculata_XP_004360785.1
Dictyostelium_purpureum_jgi54831
Paramoeba_atlantica_MMETSP0151_c32036_g1_i1.p1
Capsaspora_owczarzaki_XP_004365205.2
Sphaeroforma_arctica_XP_014152869.1
Colletotrichum_orchidophilum_XP_022477710.1
Beauveria_bassiana_XP_008603431.1
Trichoderma_harzianum_XP_024776937.1
Quercus_suber_XP_023891518.1
Phanerochaete_chrysosporium_jgi125071
Rhodotorula_graminis_XP_018268182.1
Sporobolomyces_roseus_jgi33381
Mucor_circinelloides_jgi87902
Rhizopus_microsporus_XP_023468571.1
Phycomyces_blakesleeanus_jgi28123
Lobosporangium_transversale_XP_021877827.1
Batrachochytrium_dendrobatidis_jgi17938
Spizellomyces_punctatus_XP_016611711.1
Karlodinium_micrum_MMETSP1015_c64593_g1_i1.p1
Sorites_sp_MMETSP0191_c68702_g1_i1.p2
Phaeocystis_antarctica_MMETSP1444_c16673_g1_i1.p1
Chrysochromulina_rotalis_MMETSP0287_c6310_g1_i1.p1
Scyphosphaera_apsteinii_MMETSP1333_c56791_g1_i1.p1
Bigelowiella_natans_MMETSP1052_c5377_g1_i1.p1
Norrisiella_sphaerica_MMETSP0113_c792_g1_i1.p1
Partenskyella_glossopodia_MMETSP1318_c26354_g1_i1.p1
Tiarina_fusus_MMETSP0472_c2263_g1_i1.p1
Mayorella_sp_MMETSP0417_c4452_g1_i1.p1
Stygamoeba_regulata_MMETSP0447_c7457_g1_i1.p1
Acanthamoeba_castellanii_XP_004341647.1
Alveolata_sp_MMETSP0288_c17964_g1_i1.p1
Vitrella_brassicaformis_MMETSP1451_c23612_g1_i1.p1
Perkinsus_marinus_XP_002774038.1
Chromera_velia_MMETSP0290_c885_g1_i1.p1
Helobdella_robusta_jgi193551
Caenorhabditis_elegans_NP_509122.2
Camponotus_floridanus_XP_025268113.1
Helicoverpa_armigera_XP_021194430.1
Trichoplusia_ni_XP_026735209.1
Trichinella_spiralis_XP_003367443.1
Lingula_anatina_XP_013393591.1
Oryzias_melastigma_XP_024150158.1
Boleophthalmus_pectinirostris_XP_020772721.1
Myripristis_murdjan_XP_029926091.1
Exaiptasia_pallida_XP_020910531.1
Nematostella_vectensis_XP_001630409.1
Orbicella_faveolata_XP_020622899.1
Acanthaster_planci_XP_022082566.1
Strongylocentrotus_purpuratus_XP_011680411.1
Saccoglossus_kowalevskii_XP_002733644.1
Priapulus_caudatus_XP_014664579.1
Crassostrea_virginica_XP_022335414.1
Mizuhopecten_yessoensis_XP_021367896.1
Biomphalaria_glabrata_XP_013068084.1
Trichoplax_adhaerens_XP_002117595.1
Monosiga_brevicollis_XP_001745462.1
Necator_americanus_XP_013309134.1
Alexandrium_tamarense_MMETSP0384_c117294_g1_i1.p2
Micromonas_pusilla_MMETSP1404_c11277_g1_i1.p1
Picochlorum_oklahomensis_MMETSP1161_c2695_g1_i1.p1
Pyramimonas_amylifera_MMETSP1081_c2039_g1_i1.p1
Eutreptiella_gymnastica_MMETSP0039_c19307_g1_i1.p1
Aegilops_tauschii_XP_020197862.1
Oryza_sativa_XP_015651104.1
Myrionecta_rubra_MMETSP0798_c11434_g1_i1.p1
Strombidinopsis_sp_MMETSP0463_c23832_g1_i1.p1
Rhodomonas_lens_MMETSP0484_c25299_g1_i1.p1
Guillardia_theta_CCMP2712_jgi65159
Gloeochaete_witrockiana_MMETSP0308_c661_g1_i2.p1
Cyanophora_paradoxa_tig00000194_g14749.t1
Rhodosorus_marinus_MMETSP0011_c5674_g1_i1.p1
Gracilariopsis_chorda_PXF47519.1
Porphyridium_purpureum_2095.3
Cyanoptyche_gloeocystis_MMETSP1086_c12802_g1_i1.p1
NA_WP_048083510.1
Candidatus_Nitrosomarinus_catalina_WP_086907288.1
Nitrosopumilus_sp._Nsub_WP_067958448.1
Rhodococcus_sp._SAORIC690_WP_105028211.1
Glenodinium_foliaceum_MMETSP0119_c59128_g1_i1.p2
Aureomonas_pacifica_MMETSP0108_c53833_g1_i1.p4
Heterosigma_akashiwo_MMETSP0409_c21570_g1_i1.p2
Bacillus_sp._SRB_336_WP_113723705.1
Dokdonella_fugitiva_WP_132000144.1
NA_WP_018144735.1
bacterium_endosymbiont_of_Bathymodiolus_sp._5_South_WP_139660095.1
Bathymodiolus_heckerae_thiotrophic_gill_symbiont_WP_138120123.1
Thalassiosira_miniscula_MMETSP0740_c7087_g1_i1.p1
Pseudohongiella_nitratireducens_WP_068812015.1
Leucothrix_mucor_WP_022950658.1
NA_WP_014227507.1
Acanthoeca_sp_MMETSP0105_c34358_g1_i1.p1
Solemya_velesiana_gill_symbiont_WP_078485608.1
Pseudopedinella_elastica_MMETSP1097_c14262_g1_i1.p2
Paulinella_ovalis_nuclear_SingleCell___SAG1_utg7180000002468.g1116.t1
MSTRG.11290.1.p1
Paulinella_chromatophora_CCAC0185_nuclear___scaffold5555-m48871
Orenia_metallireducens_WP_097016781.1
Kyrpidia_tusciae_WP_013074868.1
Muribaculaceae_bacterium_Isolate037_Harlan_WP_123484253.1
Bacteroidales_bacterium_WP_135985460.1
anaerobic_archaeon_MKD1_WP_147665250.1
archaeon_AArcSl_WP_119816124.1
Ignicoccus_hospitalis_WP_011998201.1
Acidianus_manzaensis_WP_148692044.1
Sulfodiicoccus_acidiphilus_WP_126449072.1
0.56

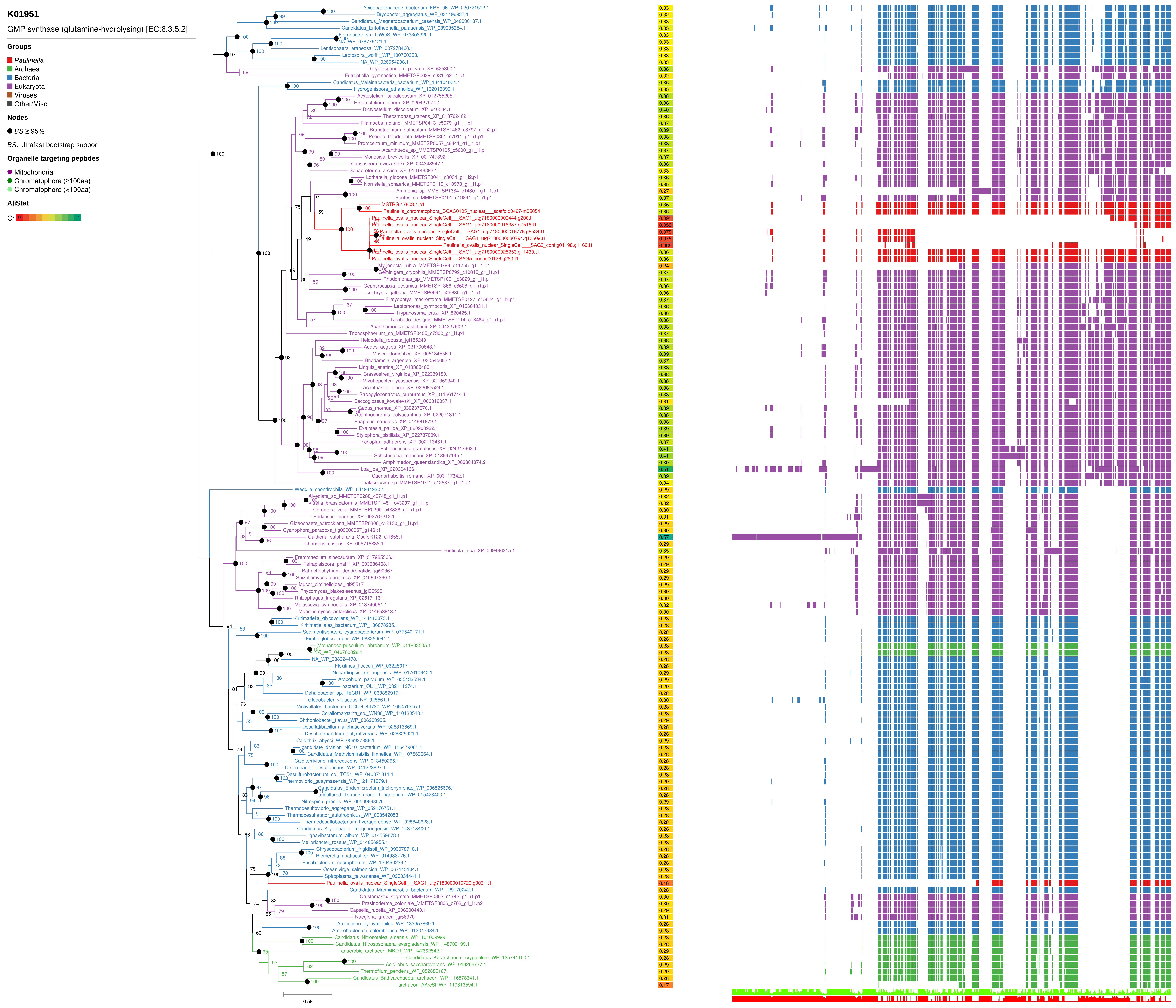

K01951
GMP synthase (glutamine-hydrolysing) [EC:6.3.5.2]
Groups
Paulinella
Archaea
Bacteria
Eukaryota
Viruses
Other/Misc
Nodes
BS ≥ 95%
BS: ultrafast bootstrap support
Organelle targeting peptides
Mitochondrial
Chromatophore (≥100aa)
Chromatophore (<100aa)
AliStat
Cr
0
1
Acidobacteriaceae_bacterium_KBS_96_WP_020721512.1
Bryobacter_aggregatus_WP_031496937.1
Candidatus_Magnetobacterium_casensis_WP_040336137.1
Candidatus_Entotheonella_palauensis_WP_089935354.1
Fibrobacter_sp._UWOS_WP_073306320.1
NA_WP_078776121.1
Lentisphaera_araneosa_WP_007278460.1
Leptospira_wolffii_WP_100760363.1
NA_WP_026054288.1
Cryptosporidium_parvum_XP_625300.1
Eutreptiella_gymnastica_MMETSP0039_c381_g2_i1.p1
Candidatus_Melainabacteria_bacterium_WP_144104034.1
Hydrogenispora_ethanolica_WP_132016899.1
Acytostelium_subglobosum_XP_012755205.1
Heterostelium_album_XP_020427974.1
Dictyostelium_discoideum_XP_640534.1
Thecamonas_trahens_XP_013762482.1
Filamoeba_nolandi_MMETSP0413_c5079_g1_i1.p1
Brandtodinium_nutriculum_MMETSP1462_c8797_g1_i2.p1
Pseudo_fraudulenta_MMETSP0851_c7911_g1_i1.p1
Prorocentrum_minimum_MMETSP0057_c8441_g1_i1.p1
Acanthoeca_sp_MMETSP0105_c5000_g1_i1.p1
Monosiga_brevicollis_XP_001747892.1
Capsaspora_owczarzaki_XP_004343547.1
Sphaeroforma_arctica_XP_014148892.1
Lotharella_globosa_MMETSP0041_c3034_g1_i2.p1
Norrisiella_sphaerica_MMETSP0113_c10978_g1_i1.p1
Ammonia_sp_MMETSP1384_c14801_g1_i1.p1
Sorites_sp_MMETSP0191_c19844_g1_i1.p1
MSTRG.17803.1.p1
Paulinella_chromatophora_CCAC0185_nuclear___scaffold3427-m35054
Paulinella_ovalis_nuclear_SingleCell___SAG1_utg7180000000444.g200.t1
Paulinella_ovalis_nuclear_SingleCell___SAG1_utg7180000016387.g7516.t1
Paulinella_ovalis_nuclear_SingleCell___SAG1_utg7180000018778.g8584.t1
Paulinella_ovalis_nuclear_SingleCell___SAG1_utg7180000030794.g13609.t1
Paulinella_ovalis_nuclear_SingleCell___SAG3_contig01198.g1166.t1
Paulinella_ovalis_nuclear_SingleCell___SAG1_utg7180000025253.g11439.t1
Paulinella_ovalis_nuclear_SingleCell___SAG5_contig00126.g283.t1
Myrionecta_rubra_MMETSP0798_c11755_g1_i1.p1
Geminigera_cryophila_MMETSP0799_c12815_g1_i1.p1
Rhodomonas_sp_MMETSP1091_c3829_g1_i1.p1
Gephyrocapsa_oceanica_MMETSP1366_c8608_g1_i1.p1
Isochrysis_galbana_MMETSP0944_c29689_g1_i1.p1
Platyophrya_macrostoma_MMETSP0127_c15624_g1_i1.p1
Leptomonas_pyrrhocoris_XP_015664031.1
Trypanosoma_cruzi_XP_820425.1
Neobodo_designis_MMETSP1114_c18464_g1_i1.p1
Acanthamoeba_castellanii_XP_004337602.1
Trichosphaerium_sp_MMETSP0405_c7300_g1_i1.p1
Helobdella_robusta_jgi185249
Aedes_aegypti_XP_021700843.1
Musca_domestica_XP_005184556.1
Rhodamnia_argentea_XP_030545683.1
Lingula_anatina_XP_013388480.1
Crassostrea_virginica_XP_022339180.1
Mizuhopecten_yessoensis_XP_021369340.1
Acanthaster_planci_XP_022085524.1
Strongylocentrotus_purpuratus_XP_011661744.1
Saccoglossus_kowalevskii_XP_006812037.1
Gadus_morhua_XP_030237070.1
Acanthochromis_polyacanthus_XP_022071311.1
Priapulus_caudatus_XP_014681879.1
Exaiptasia_pallida_XP_020900922.1
Stylophora_pistillata_XP_022787009.1
Trichoplax_adhaerens_XP_002113461.1
Echinococcus_granulosus_XP_024347903.1
Schistosoma_mansoni_XP_018647145.1
Amphimedon_queenslandica_XP_003384374.2
Loa_loa_XP_020304166.1
Caenorhabditis_remanei_XP_003117342.1
Thalassiosira_sp_MMETSP1071_c12587_g1_i1.p1
Waddlia_chondrophila_WP_041941920.1
Alveolata_sp_MMETSP0288_c6748_g1_i1.p1
Vitrella_brassicaformis_MMETSP1451_c43237_g1_i1.p1
Chromera_velia_MMETSP0290_c48838_g1_i1.p1
Perkinsus_marinus_XP_002767312.1
Gloeochaete_witrockiana_MMETSP0308_c12130_g1_i1.p1
Cyanophora_paradoxa_tig00000057_g146.t1
Galdieria_sulphuraria_GsulpRT22_G1655.1
Chondrus_crispus_XP_005716838.1
Fonticula_alba_XP_009496315.1
Eremothecium_sinecaudum_XP_017985566.1
Tetrapisispora_phaffii_XP_003686408.1
Batrachochytrium_dendrobatidis_jgi90367
Spizellomyces_punctatus_XP_016607360.1
Mucor_circinelloides_jgi95517
Phycomyces_blakesleeanus_jgi35595
Rhizophagus_irregularis_XP_025171131.1
Malassezia_sympodialis_XP_018740081.1
Moesziomyces_antarcticus_XP_014653813.1
Kiritimatiella_glycovorans_WP_144413873.1
Kiritimatiellales_bacterium_WP_136078935.1
Sedimentisphaera_cyanobacteriorum_WP_077540171.1
Fimbriiglobus_ruber_WP_088259041.1
Methanocorpusculum_labreanum_WP_011833505.1
NA_WP_042700028.1
NA_WP_038324478.1
Flexilinea_flocculi_WP_062280171.1
Nocardiopsis_xinjiangensis_WP_017610640.1
Atopobium_parvulum_WP_035432534.1
bacterium_OL1_WP_032111274.1
Dehalobacter_sp._TeCB1_WP_068882917.1
Gloeobacter_violaceus_NP_925561.1
Victivallales_bacterium_CCUG_44730_WP_106051345.1
Coraliomargarita_sp._WN38_WP_110130513.1
Chthoniobacter_flavus_WP_006983935.1
Desulfatibacillum_aliphaticivorans_WP_028313869.1
Desulfatirhabdium_butyrativorans_WP_028325921.1
Caldithrix_abyssi_WP_006927386.1
candidate_division_NC10_bacterium_WP_116479081.1
Candidatus_Methylomirabilis_limnetica_WP_107563664.1
Calditerrivibrio_nitroreducens_WP_013450265.1
Deferribacter_desulfuricans_WP_041223827.1
Desulfurobacterium_sp._TC5I_WP_040371811.1
Thermovibrio_guaymasensis_WP_121171279.1
Candidatus_Endomicrobium_trichonymphae_WP_096525696.1
uncultured_Termite_group_1_bacterium_WP_015423400.1
Nitrospina_gracilis_WP_005006985.1
Thermodesulfovibrio_aggregans_WP_059176751.1
Thermodesulfatator_autotrophicus_WP_068542053.1
Thermodesulfobacterium_hveragerdense_WP_028840628.1
Candidatus_Kryptobacter_tengchongensis_WP_143713400.1
Ignavibacterium_album_WP_014559678.1
Melioribacter_roseus_WP_014856955.1
Chryseobacterium_frigidisoli_WP_090078718.1
Riemerella_anatipestifer_WP_014938776.1
Fusobacterium_necrophorum_WP_129490236.1
Oceanivirga_salmonicida_WP_067143104.1
Spiroplasma_taiwanense_WP_020834441.1
Paulinella_ovalis_nuclear_SingleCell___SAG1_utg7180000019729.g9031.t1
Candidatus_Marinimicrobia_bacterium_WP_129170242.1
Crustomastix_stigmata_MMETSP0803_c1742_g1_i1.p1
Prasinoderma_coloniale_MMETSP0806_c703_g1_i1.p2
Capsella_rubella_XP_006300443.1
Naegleria_gruberi_jgi58970
Aminivibrio_pyruvatiphilus_WP_133957669.1
Aminobacterium_colombiense_WP_013047984.1
Candidatus_Nitrosotalea_sinensis_WP_101009999.1
Candidatus_Nitrososphaera_evergladensis_WP_148702199.1
anaerobic_archaeon_MKD1_WP_147662542.1
Candidatus_Korarchaeum_cryptofilum_WP_125741100.1
Acidilobus_saccharovorans_WP_013266777.1
Thermofilum_pendens_WP_052885187.1
Candidatus_Bathyarchaeota_archaeon_WP_116578341.1
archaeon_AArcSl_WP_119813594.1
0.59

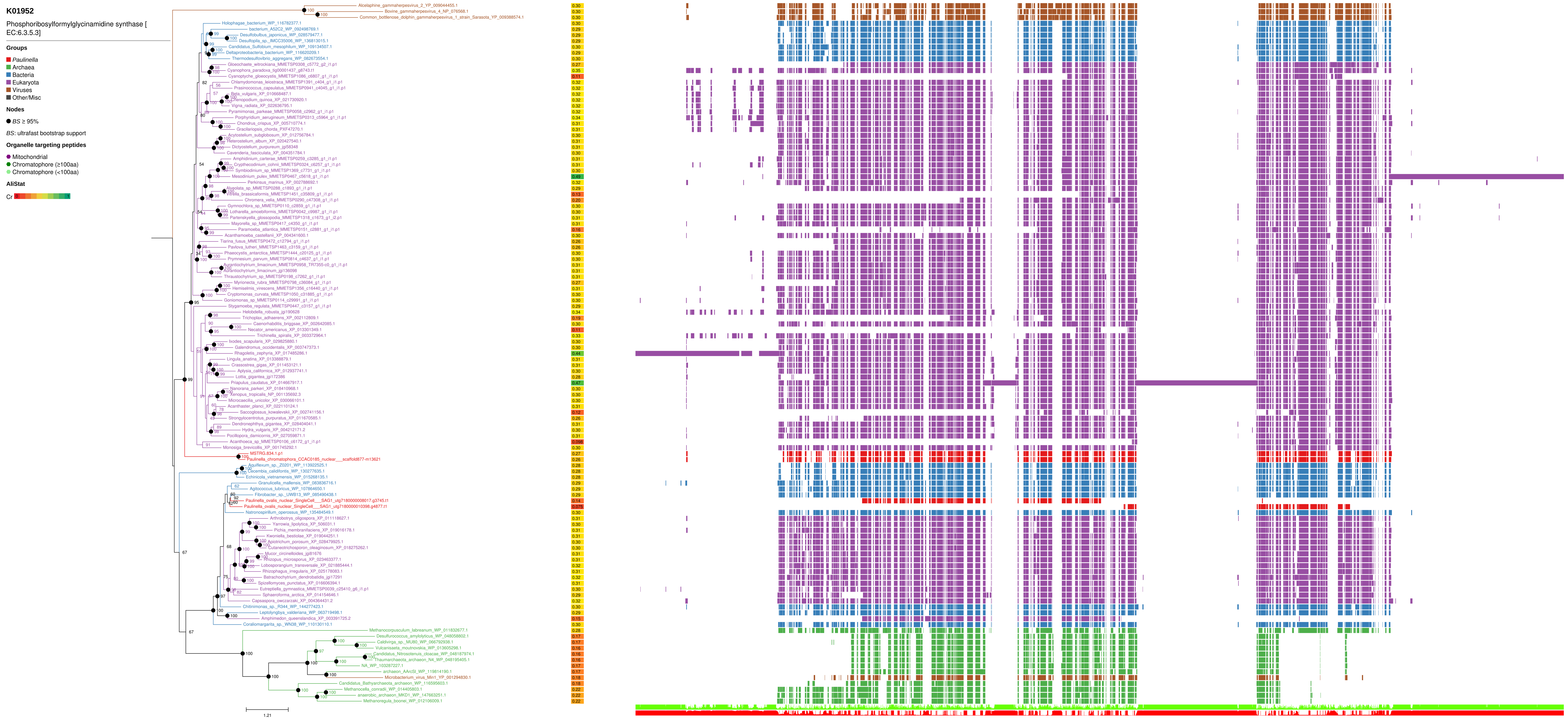
K01952
Phosphoribosylformylglycinamidine synthase [EC:6.3.5.3]
Groups
Paulinella
Archaea
Bacteria
Eukaryota
Viruses
Other/Misc
Nodes
BS ≥ 95%
BS: ultrafast bootstrap support
Organelle targeting peptides
Mitochondrial
Chromatophore (≥100aa)
Chromatophore (<100aa)
AliStat
Cr
Alcelaphine_gammaherpesvirus_2_YP_009044455.1 0.30
Bovine_gammaherpesvirus_4_NP_076568.1 0.30
Common_bottlenose_dolphin_gammaherpesvirus_1_strain_Sarasota_YP_009388574.1 0.30
Holophagae_bacterium_WP_116782377.1 0.30
bacterium_A52C2_WP_092498769.1 0.29
Desulfobulbus_japonicus_WP_028579477.1 0.29
Desulfopila_sp._IMCC35006_WP_136813015.1 0.29
Candidatus_Sulfobium_mesophilum_WP_109134507.1 0.30
Deltaproteobacteria_bacterium_WP_116620209.1 0.29
Thermodesulfovibrio_aggregans_WP_082673554.1 0.30
Gloeochaete_witrockiana_MMETSP0308_c5772_g2_i1.p1 0.27
Cyanophora_paradoxa_tig00001437_g8743.t1 0.35
Cyanoptyche_gloeocystis_MMETSP1086_c6807_g1_i1.p1 0.11
Chlamydomonas_leiostraca_MMETSP1391_c404_g1_i1.p1 0.32
Prasinococcus_capsulatus_MMETSP0941_c4045_g1_i1.p1 0.32
Beta_vulgaris_XP_010668487.1 0.32
Chenopodium_quinoa_XP_021730920.1 0.32
Vigna_radiata_XP_022636795.1 0.32
Pyramimonas_parkeae_MMETSP0058_c2962_g1_i1.p1 0.32
Porphyridium_aerugineum_MMETSP0313_c5964_g1_i1.p1 0.34
Chondrus_crispus_XP_005710774.1 0.31
Gracilariopsis_chorda_PXF47270.1 0.31
Acytostelium_subglobosum_XP_012756784.1 0.30
Heterostelium_album_XP_020427540.1 0.31
Dictyostelium_purpureum_jgi58348 0.31
Cavenderia_fasciculata_XP_004351784.1 0.30
Amphidinium_carterae_MMETSP0259_c3285_g1_i1.p1 0.31
Crypthecodinium_cohnii_MMETSP0324_c6257_g1_i1.p1 0.31
Symbiodinium_sp_MMETSP1369_c7731_g1_i1.p1 0.30
Mesodinium_pulex_MMETSP0467_c5618_g1_i1.p1 0.49
Perkinsus_marinus_XP_002788692.1 0.32
Alveolata_sp_MMETSP0288_c1893_g1_i1.p1 0.29
Vitrella_brassicaformis_MMETSP1451_c35809_g1_i1.p1 0.13
Chromera_velia_MMETSP0290_c47308_g1_i1.p1 0.20
Gymnochlora_sp_MMETSP0110_c2859_g1_i1.p1 0.30
Lotharella_amoebiformis_MMETSP0042_c9987_g1_i1.p1 0.30
Partenskyella_glossopodia_MMETSP1318_c1673_g1_i2.p1 0.31
Mayorella_sp_MMETSP0417_c4350_g1_i1.p1 0.31
Paramoeba_atlantica_MMETSP0151_c2881_g1_i1.p1 0.16
Acanthamoeba_castellanii_XP_004341600.1 0.30
Tiarina_fusus_MMETSP0472_c12794_g1_i1.p1 0.26
Pavlova_lutheri_MMETSP1463_c3159_g1_i1.p1 0.26
Phaeocystis_antarctica_MMETSP1444_c20125_g1_i1.p1 0.30
Prymnesium_parvum_MMETSP0814_c4637_g1_i1.p1 0.30
Aurantiochytrium_limacinum_MMETSP0958_TR7355-c0_g1_i1.p1 0.31
Aurantiochytrium_limacinum_jgi136098 0.31
Thraustochytrium_sp_MMETSP0198_c7262_g1_i1.p1 0.31
Myrionecta_rubra_MMETSP0798_c36084_g1_i1.p1 0.27
Hemiselmis_virescens_MMETSP1356_c16440_g1_i1.p1 0.31
Cryptomonas_curvata_MMETSP1050_c31885_g1_i1.p1 0.30
Goniomonas_sp_MMETSP0114_c29991_g1_i1.p1 0.30
Stygamoeba_regulata_MMETSP0447_c3157_g1_i1.p1 0.29
Helobdella_robusta_jgi190628 0.34
Trichoplax_adhaerens_XP_002112809.1 0.19
Caenorhabditis_briggsae_XP_002642085.1 0.30
Necator_americanus_XP_013301349.1 0.11
Trichinella_spiralis_XP_003372964.1 0.33
Ixodes_scapularis_XP_029825880.1 0.30
Galendromus_occidentalis_XP_003747373.1 0.30
Rhagoletis_zephyria_XP_017485286.1 0.44
Lingula_anatina_XP_013388879.1 0.31
Crassostrea_gigas_XP_011453121.1 0.31
Aplysia_californica_XP_012937741.1 0.30
Lottia_gigantea_jgi172386 0.28
Priapulus_caudatus_XP_014667917.1 0.47
Nanorana_parkeri_XP_018410968.1 0.30
Xenopus_tropicalis_NP_001135692.3 0.30
Microcaecilia_unicolor_XP_030066101.1 0.30
Acanthaster_planci_XP_022110124.1 0.31
Saccoglossus_kowalevskii_XP_002741156.1 0.12
Strongylocentrotus_purpuratus_XP_011670585.1 0.26
Dendronephthya_gigantea_XP_028404041.1 0.31
Hydra_vulgaris_XP_004212171.2 0.30
Pocillopora_damicornis_XP_027059871.1 0.31
Acanthoeca_sp_MMETSP0106_c6172_g1_i1.p1 0.098
Monosiga_brevicollis_XP_001745292.1 0.30
MSTRG.834.1.p1 0.27
Paulinella_chromatophora_CCAC0185_nuclear___scaffold877-m13621 0.26
Aquiflexum_sp._Z0201_WP_113922525.1 0.28
Cecembia_calidifontis_WP_130277635.1 0.28
Echinicola_vietnamensis_WP_015268135.1 0.28
Granulicella_mallensis_WP_083836716.1 0.29
Agitococcus_lubricus_WP_107864650.1 0.29
Fibrobacter_sp._UWB13_WP_085490438.1 0.29
Paulinella_ovalis_nuclear_SingleCell___SAG1_utg7180000008017.g3745.t1 0.14
Paulinella_ovalis_nuclear_SingleCell___SAG1_utg7180000010398.g4877.t1 0.075
Natronospirillum_operossus_WP_135484549.1 0.30
Arthrobotrys_oligospora_XP_011118627.1 0.31
Yarrowia_lipolytica_XP_506031.1 0.30
Pichia_membranifaciens_XP_019016178.1 0.31
Kwoniella_bestiolae_XP_019044251.1 0.31
Apiotrichum_porosum_XP_028479925.1 0.30
Cutaneotrichosporon_oleaginosum_XP_018275262.1 0.30
Mucor_circinelloides_jgi81676 0.31
Rhizopus_microsporus_XP_023463377.1 0.31
Lobosporangium_transversale_XP_021885444.1 0.32
Rhizophagus_irregularis_XP_025178083.1 0.31
Batrachochytrium_dendrobatidis_jgi17291 0.32
Spizellomyces_punctatus_XP_016606394.1 0.31
Eutreptiella_gymnastica_MMETSP0039_c25410_g6_i1.p1 0.30
Sphaeroforma_arctica_XP_014154646.1 0.29
Capsaspora_owczarzaki_XP_004364431.2 0.32
Chitinimonas_sp._R344_WP_144277423.1 0.30
Leptolyngbya_valderiana_WP_063719498.1 0.29
Amphimedon_queenslandica_XP_003391725.2 0.15
Coraliomargarita_sp._WN38_WP_110130110.1 0.30
Methanocorpusculum_labreanum_WP_011832677.1 0.28
Desulfurococcus_amylolyticus_WP_048058802.1 0.17
Caldivirga_sp._MU80_WP_066792938.1 0.17
Vulcanisaeta_moutnovskia_WP_013605298.1 0.16
Candidatus_Nitrosotenuis_cloacae_WP_048187974.1 0.16
Thaumarchaeota_archaeon_N4_WP_048195405.1 0.16
NA_WP_103287227.1 0.17
archaeon_AArcSl_WP_119814190.1 0.17
Microbacterium_virus_Min1_YP_001294830.1 0.18
Candidatus_Bathyarchaeota_archaeon_WP_116595603.1 0.18
Methanocella_conradii_WP_014405803.1 0.22
anaerobic_archaeon_MKD1_WP_147663251.1 0.22
Methanoregula_boonei_WP_012106009.1 0.22
1.21

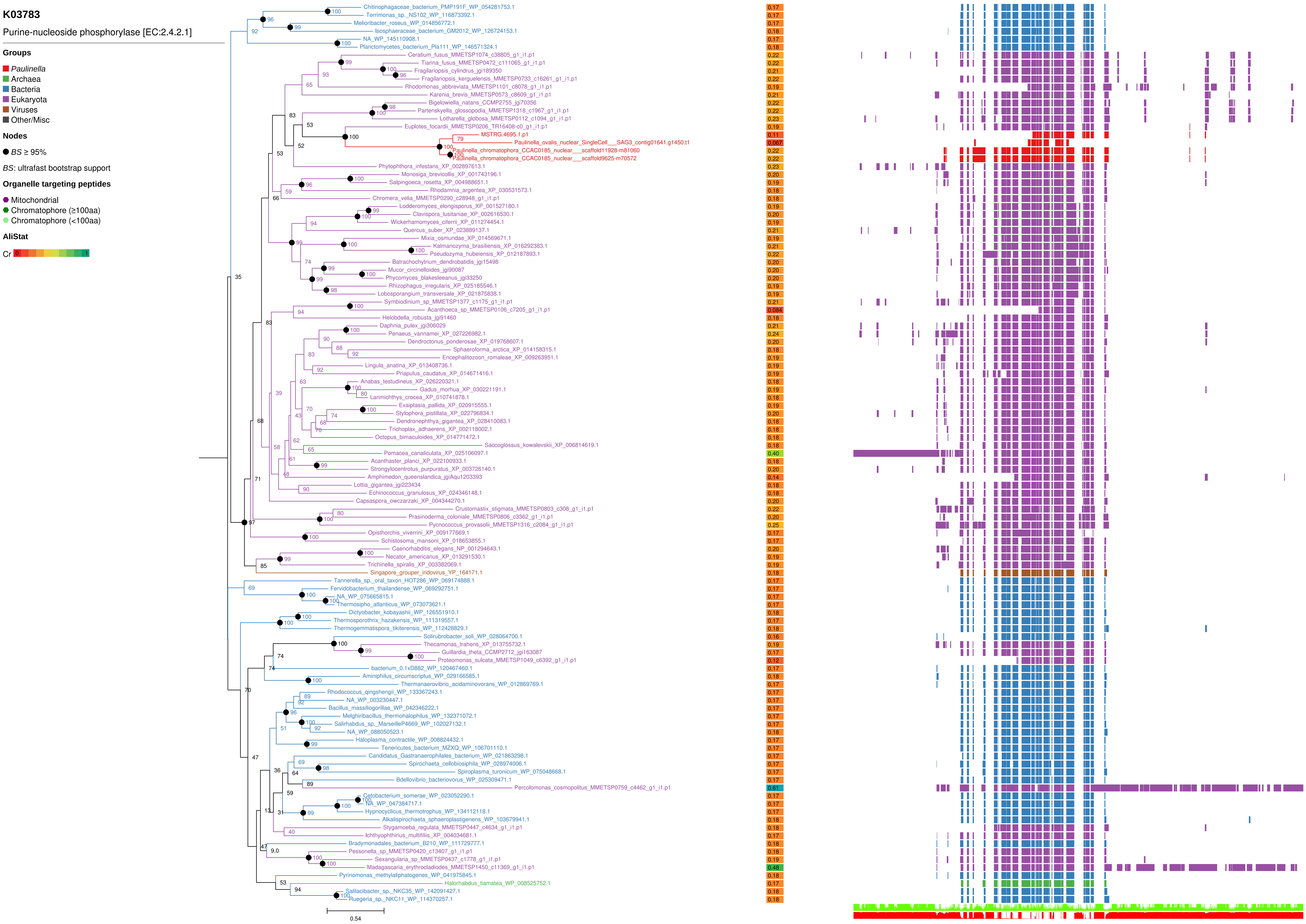
K03783
Purine-nucleoside phosphorylase [EC:2.4.2.1]
Groups
Paulinella
Archaea
Bacteria
Eukaryota
Viruses
Other/Misc
Nodes
BS ≥ 95%
BS: ultrafast bootstrap support
Organelle targeting peptides
Mitochondrial
Chromatophore (≥100aa)
Chromatophore (<100aa)
AliStat
Cr
0
1
Chitinophagaceae_bacterium_PMP191F_WP_054281753.1
Terrimonas_sp._NS102_WP_116873392.1
Melioribacter_roseus_WP_014856772.1
Isosphaeraceae_bacterium_GM2012_WP_126724153.1
NA_WP_145110908.1
Planctomycetes_bacterium_Pla111_WP_146571324.1
Ceratium_fusus_MMETSP1074_c38805_g1_i1.p1
Tiarina_fusus_MMETSP0472_c111065_g1_i1.p1
Fragilariopsis_cylindrus_jgi189350
Fragilariopsis_kerguelensis_MMETSP0733_c16261_g1_i1.p1
Rhodomonas_abbreviata_MMETSP1101_c8078_g1_i1.p1
Karenia_brevis_MMETSP0573_c8609_g1_i1.p1
Bigelowiella_natans_CCMP2755_jgi70356
Partenskyella_glossopodia_MMETSP1318_c1967_g1_i1.p1
Lotharella_globosa_MMETSP0112_c1094_g1_i1.p1
Euplotes_focardii_MMETSP0206_TR16408-c0_g1_i1.p1
MSTRG.4695.1.p1
Paulinella_ovalis_nuclear_SingleCell___SAG3_contig01641.g1450.t1
Paulinella_chromatophora_CCAC0185_nuclear___scaffold11928-m81060
Paulinella_chromatophora_CCAC0185_nuclear___scaffold9625-m70572
Phytophthora_infestans_XP_002897613.1
Monosiga_brevicollis_XP_001743196.1
Salpingoeca_rosetta_XP_004988651.1
Rhodamnia_argentea_XP_030531573.1
Chromera_velia_MMETSP0290_c28948_g1_i1.p1
Lodderomyces_elongisporus_XP_001527180.1
Clavispora_lusitaniae_XP_002616530.1
Wickerhamomyces_ciferrii_XP_011274454.1
Quercus_suber_XP_023889137.1
Mixia_osmundae_XP_014569671.1
Kalmanozyma_brasiliensis_XP_016292383.1
Pseudozyma_hubeiensis_XP_012187893.1
Batrachochytrium_dendrobatidis_jgi15498
Mucor_circinelloides_jgi90087
Phycomyces_blakesleeanus_jgi33250
Rhizophagus_irregularis_XP_025185546.1
Lobosporangium_transversale_XP_021875838.1
Symbiodinium_sp_MMETSP1377_c1175_g1_i1.p1
Acanthoeca_sp_MMETSP0106_c7205_g1_i1.p1
Helobdella_robusta_jgi91460
Daphnia_pulex_jgi306029
Penaeus_vannamei_XP_027226982.1
Dendroctonus_ponderosae_XP_019768607.1
Sphaeroforma_arctica_XP_014158315.1
Encephalitozoon_romaleae_XP_009263951.1
Lingula_anatina_XP_013408736.1
Priapulus_caudatus_XP_014671416.1
Anabas_testudineus_XP_026220321.1
Gadus_morhua_XP_030221191.1
Larimichthys_crocea_XP_010741878.1
Exaiptasia_pallida_XP_020915555.1
Stylophora_pistillata_XP_022796834.1
Dendronephthya_gigantea_XP_028410083.1
Trichoplax_adhaerens_XP_002118002.1
Octopus_bimaculoides_XP_014771472.1
Saccoglossus_kowalevskii_XP_006814619.1
Pomacea_canaliculata_XP_025106097.1
Acanthaster_planci_XP_022100933.1
Strongylocentrotus_purpuratus_XP_003726140.1
Amphimedon_queenslandica_jgiAqu1203393
Lottia_gigantea_jgi223434
Echinococcus_granulosus_XP_024346148.1
Capsaspora_owczarzaki_XP_004344270.1
Crustomastix_stigmata_MMETSP0803_c308_g1_i1.p1
Prasinoderma_coloniale_MMETSP0806_c3362_g1_i1.p1
Pycnococcus_provasolii_MMETSP1316_c2084_g1_i1.p1
Opisthorchis_viverrini_XP_009177669.1
Schistosoma_mansoni_XP_018653855.1
Caenorhabditis_elegans_NP_001294643.1
Necator_americanus_XP_013291530.1
Trichinella_spiralis_XP_003382069.1
Singapore_grouper_iridovirus_YP_164171.1
Tannerella_sp._oral_taxon_HOT286_WP_069174888.1
Fervidobacterium_thailandense_WP_069292751.1
NA_WP_075665815.1
Thermosipho_atlanticus_WP_073073621.1
Dictyobacter_kobayashii_WP_126551910.1
Thermosporothrix_hazakensis_WP_111319557.1
Thermogemmatispora_tikiterensis_WP_112428829.1
Solirubrobacter_soli_WP_028064700.1
Thecamonas_trahens_XP_013755732.1
Guillardia_theta_CCMP2712_jgi163087
Proteomonas_sulcata_MMETSP1049_c6392_g1_i1.p1
bacterium_0.1xD882_WP_120467460.1
Aminiphilus_circumscriptus_WP_029166585.1
Thermanaerovibrio_acidaminovorans_WP_012869769.1
Rhodococcus_qingshengii_WP_133367243.1
NA_WP_003230447.1
Bacillus_massiliogorillae_WP_042346222.1
Melghiribacillus_thermohalophilus_WP_132371072.1
Salirhabdus_sp._Marseille-P4669_WP_102027132.1
NA_WP_088050523.1
Haloplasma_contractile_WP_008824432.1
Tenericutes_bacterium_MZXQ_WP_106701110.1
Candidatus_Gastranaerophilales_bacterium_WP_021863298.1
Spirochaeta_cellobiosiphila_WP_028974006.1
Spiroplasma_turonicum_WP_075048668.1
Bdellovibrio_bacteriovorus_WP_025309471.1
Percolomonas_cosmopolitus_MMETSP0759_c4462_g1_i1.p1
Cetobacterium_somerae_WP_023052290.1
NA_WP_047384717.1
Hypnocyclicus_thermotrophus_WP_134112118.1
Alkalispirochaeta_sphaeroplastigenens_WP_103679941.1
Stygamoeba_regulata_MMETSP0447_c4634_g1_i1.p1
Ichthyophthirius_multifiliis_XP_004034681.1
Bradymonadales_bacterium_B210_WP_111729777.1
Pessonella_sp_MMETSP0420_c13407_g1_i1.p1
Sexangularia_sp_MMETSP0437_c1778_g1_i1.p1
Madagascaria_erythrocladioides_MMETSP1450_c11369_g1_i1.p1
Pyrinomonas_methylaliphatogenes_WP_041975845.1
Halorhabdus_tiamatea_WP_008525752.1
Salilacibacter_sp._NKC35_WP_142091427.1
Ruegeria_sp._NKC11_WP_114370257.1
0.54

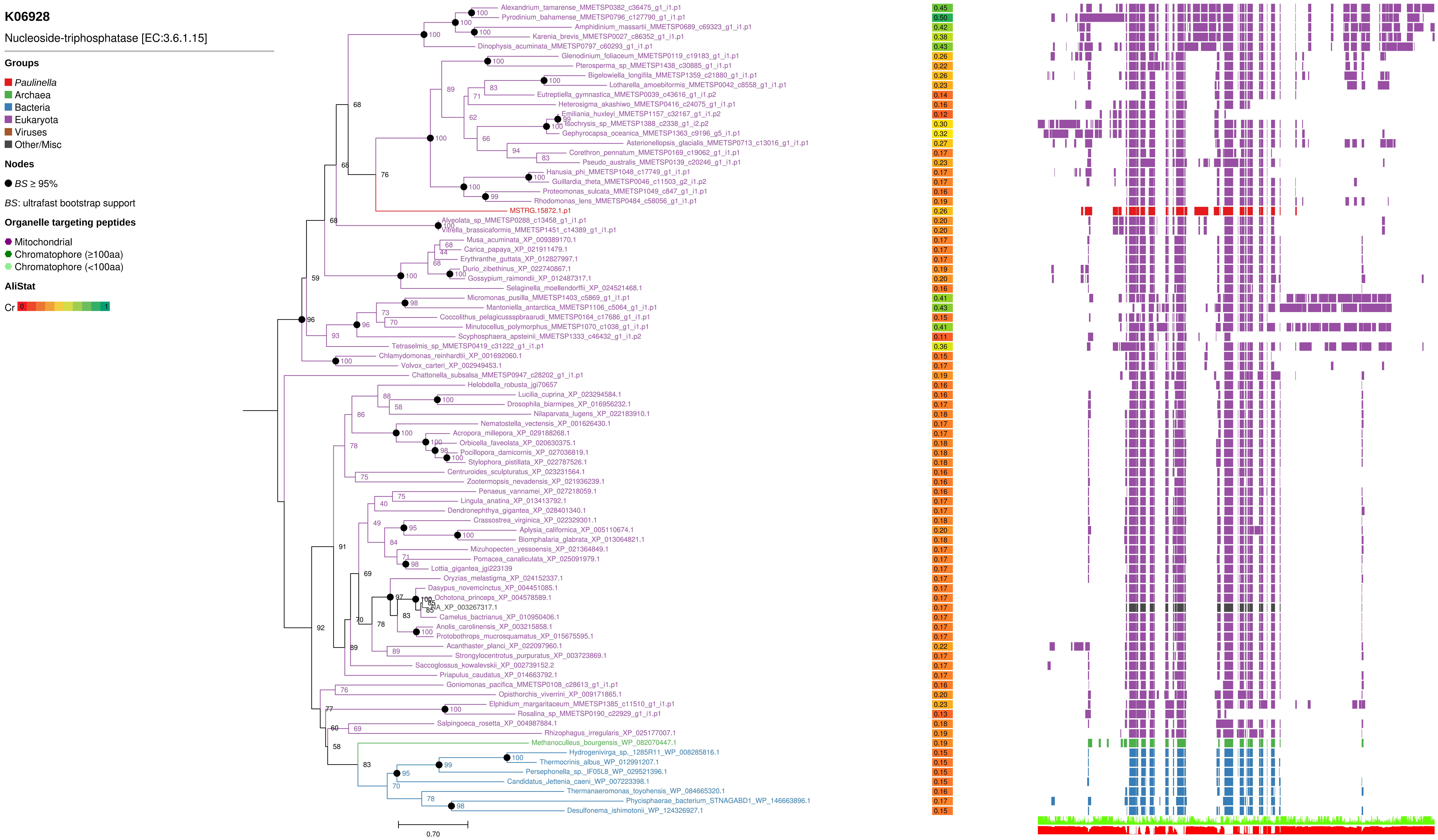

K06928
Nucleoside-triphosphatase [EC:3.6.1.15]
Groups
Paulinella
Archaea
Bacteria
Eukaryota
Viruses
Other/Misc
Nodes
BS ≥ 95%
BS: ultrafast bootstrap support
Organelle targeting peptides
Mitochondrial
Chromatophore (≥100aa)
Chromatophore (<100aa)
AliStat
Cr
Alexandrium_tamarense_MMETSP0382_c36475_g1_i1.p1
Pyrodinium_bahamense_MMETSP0796_c127790_g1_i1.p1
Amphidinium_massartii_MMETSP0689_c69323_g1_i1.p1
Karenia_brevis_MMETSP0027_c86352_g1_i1.p1
Dinophysis_acuminata_MMETSP0797_c60293_g1_i1.p1
Glenodinium_foliaceum_MMETSP0119_c19183_g1_i1.p1
Pterosperma_sp_MMETSP1438_c30885_g1_i1.p1
Bigelowiella_longifila_MMETSP1359_c21880_g1_i1.p1
Lotharella_amoebiformis_MMETSP0042_c8558_g1_i1.p1
Eutreptiella_gymnastica_MMETSP0039_c43616_g1_i1.p2
Heterosigma_akashiwo_MMETSP0416_c24075_g1_i1.p1
Emiliania_huxleyi_MMETSP1157_c32167_g1_i1.p2
Isochrysis_sp_MMETSP1388_c2338_g1_i2.p2
Gephyrocapsa_oceanica_MMETSP1363_c9196_g5_i1.p1
Asterionellopsis_glacialis_MMETSP0713_c13016_g1_i1.p1
Corethron_pennatum_MMETSP0169_c19062_g1_i1.p1
Pseudo_australis_MMETSP0139_c20246_g1_i1.p1
Hanusia_phi_MMETSP1048_c17749_g1_i1.p1
Guillardia_theta_MMETSP0046_c11503_g2_i1.p2
Proteomonas_sulcata_MMETSP1049_c847_g1_i1.p1
Rhodomonas_lens_MMETSP0484_c58056_g1_i1.p1
MSTRG.15872.1.p1
Alveolata_sp_MMETSP0288_c13458_g1_i1.p1
Vitrella_brassicaformis_MMETSP1451_c14389_g1_i1.p1
Musa_acuminata_XP_009389170.1
Carica_papaya_XP_021911479.1
Erythranthe_guttata_XP_012827997.1
Durio_zibethinus_XP_022740867.1
Gossypium_raimondii_XP_012487317.1
Selaginella_moellendorffii_XP_024521468.1
Micromonas_pusilla_MMETSP1403_c5869_g1_i1.p1
Mantoniella_antarctica_MMETSP1106_c5064_g1_i1.p1
Coccolithus_pelagicusssppbraarudi_MMETSP0164_c17686_g1_i1.p1
Minutocellus_polymorphus_MMETSP1070_c1038_g1_i1.p1
Scyphosphaera_apsteinii_MMETSP1333_c46432_g1_i1.p2
Tetraselmis_sp_MMETSP0419_c31222_g1_i1.p1
Chlamydomonas_reinhardtii_XP_001692060.1
Volvox_carteri_XP_002949453.1
Chattonella_subsalsa_MMETSP0947_c28202_g1_i1.p1
Helobdella_robusta_jgi70657
Lucilia_cuprina_XP_023294584.1
Drosophila_biarmipes_XP_016956232.1
Nilaparvata_lugens_XP_022183910.1
Nematostella_vectensis_XP_001626430.1
Acropora_millepora_XP_029188268.1
Orbicella_faveolata_XP_020630375.1
Pocillopora_damicornis_XP_027036819.1
Stylophora_pistillata_XP_022787526.1
Centruroides_sculpturatus_XP_023231564.1
Zootermopsis_nevadensis_XP_021936239.1
Penaeus_vannamei_XP_027218059.1
Lingula_anatina_XP_013413792.1
Dendronephthya_gigantea_XP_028401340.1
Crassostrea_virginica_XP_022329301.1
Aplysia_californica_XP_005110674.1
Biomphalaria_glabrata_XP_013064821.1
Mizuhopecten_yessoensis_XP_021364849.1
Pomacea_canaliculata_XP_025091979.1
Lottia_gigantea_jgi223139
Oryzias_melastigma_XP_024152337.1
Dasypus_novemcinctus_XP_004451085.1
Ochotona_princeps_XP_004578589.1
NA_XP_003267317.1
Camelus_bactrianus_XP_010950406.1
Anolis_carolinensis_XP_003215858.1
Protobothrops_mucrosquamatus_XP_015675595.1
Acanthaster_planci_XP_022097960.1
Strongylocentrotus_purpuratus_XP_003723869.1
Saccoglossus_kowalevskii_XP_002739152.2
Priapulus_caudatus_XP_014663792.1
Goniomonas_pacifica_MMETSP0108_c28613_g1_i1.p1
Opisthorchis_viverrini_XP_009171865.1
Elphidium_margaritaceum_MMETSP1385_c11510_g1_i1.p1
Rosalina_sp_MMETSP0190_c22929_g1_i1.p1
Salpingoeca_rosetta_XP_004987884.1
Rhizophagus_irregularis_XP_025177007.1
Methanoculleus_bourgensis_WP_082070447.1
Hydrogenivirga_sp._1285R11_WP_008285816.1
Thermocrinis_albus_WP_012991207.1
Persephonella_sp._IF05L8_WP_029521396.1
Candidatus_Jettenia_caeni_WP_007223398.1
Thermanaeromonas_toyohensis_WP_084665320.1
Phycisphaerae_bacterium_STNAGABD1_WP_146663896.1
Desulfonema_ishimotonii_WP_124326927.1
0.70

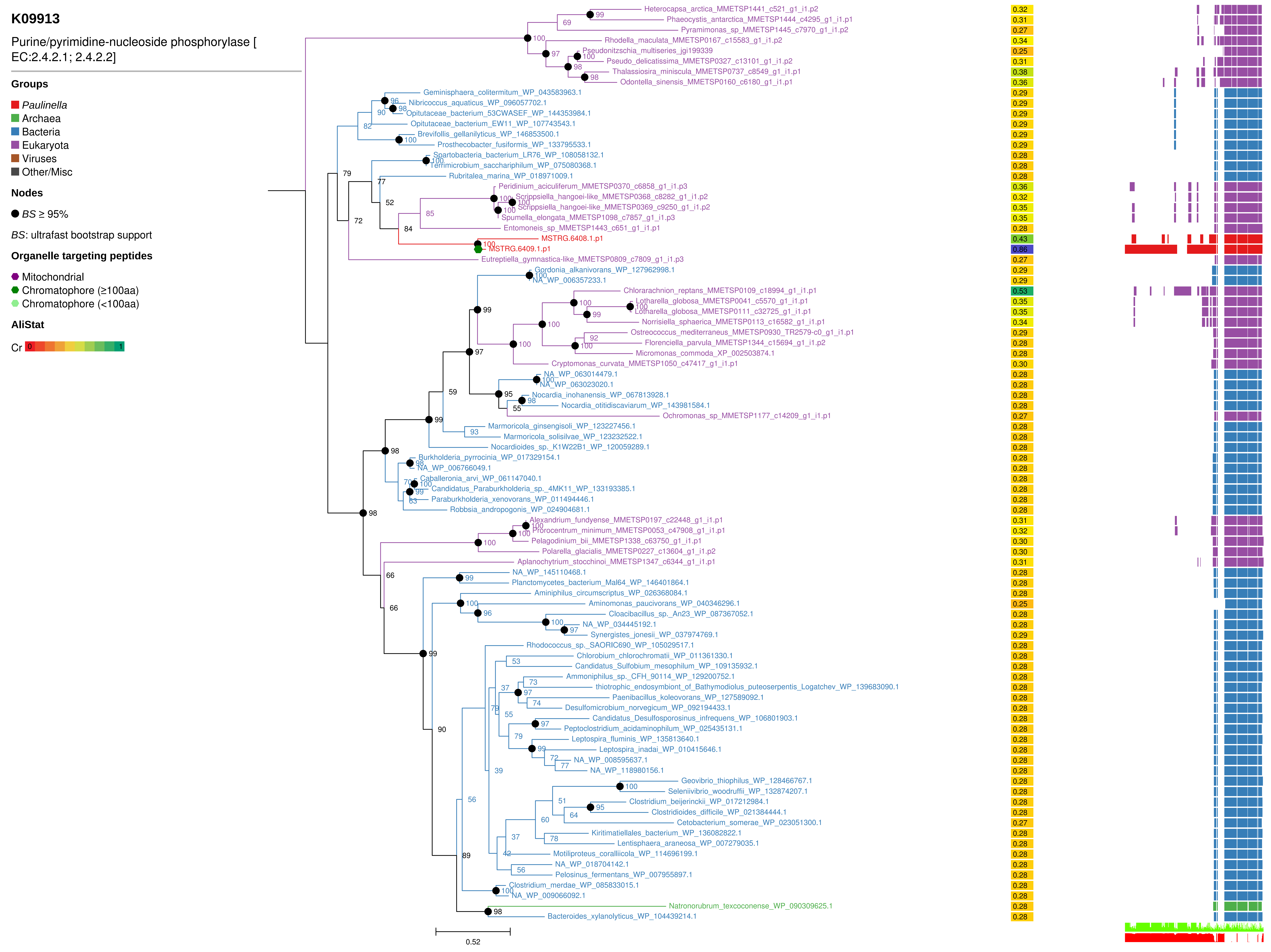
K09913
Purine/pyrimidine-nucleoside phosphorylase [EC:2.4.2.1; 2.4.2.2]
Groups
Paulinella
Archaea
Bacteria
Eukaryota
Viruses
Other/Misc
Nodes
BS ≥ 95%
BS: ultrafast bootstrap support
Organelle targeting peptides
Mitochondrial
Chromatophore (≥100aa)
Chromatophore (<100aa)
AliStat
Cr 0 1
Heterocapsa_arctica_MMETSP1441_c521_g1_i1.p2
Phaeocystis_antarctica_MMETSP1444_c4295_g1_i1.p1
Pyramimonas_sp_MMETSP1445_c7970_g1_i1.p2
Rhodella_maculata_MMETSP0167_c15583_g1_i1.p2
Pseudonitzschia_multiseries_jgi199339
Pseudo_delicatissima_MMETSP0327_c13101_g1_i1.p2
Thalassiosira_miniscula_MMETSP0737_c8549_g1_i1.p1
Odontella_sinensis_MMETSP0160_c6180_g1_i1.p1
Geminisphaera_colitermitum_WP_043583963.1
Nibricoccus_aquaticus_WP_096057702.1
Opitutaceae_bacterium_53CWASEF_WP_144353984.1
Opitutaceae_bacterium_EW11_WP_107743543.1
Brevifollis_gellanilyticus_WP_146853500.1
Prosthecobacter_fusiformis_WP_133795533.1
Spartobacteria_bacterium_LR76_WP_108058132.1
Terrimicrobium_sacchariphilum_WP_075080368.1
Rubritalea_marina_WP_018971009.1
Peridinium_aciculiferum_MMETSP0370_c6858_g1_i1.p3
Scrippsiella_hangoei-like_MMETSP0368_c8282_g1_i1.p2
Scrippsiella_hangoei-like_MMETSP0369_c9250_g1_i1.p2
Spumella_elongata_MMETSP1098_c7857_g1_i1.p3
Entomoneis_sp_MMETSP1443_c651_g1_i1.p1
MSTRG.6408.1.p1
MSTRG.6409.1.p1
Eutreptiella_gymnastica-like_MMETSP0809_c7809_g1_i1.p3
Gordonia_alkanivorans_WP_127962998.1
NA_WP_006357233.1
Chlorarachnion_reptans_MMETSP0109_c18994_g1_i1.p1
Lotharella_globosa_MMETSP0041_c5570_g1_i1.p1
Lotharella_globosa_MMETSP0111_c32725_g1_i1.p1
Norrisiella_sphaerica_MMETSP0113_c16582_g1_i1.p1
Ostreococcus_mediterraneus_MMETSP0930_TR2579-c0_g1_i1.p1
Florenciella_parvula_MMETSP1344_c15694_g1_i1.p2
Micromonas_commoda_XP_002503874.1
Cryptomonas_curvata_MMETSP1050_c47417_g1_i1.p1
NA_WP_063014479.1
NA_WP_063023020.1
Nocardia_inohanensis_WP_067813928.1
Nocardia_otitidiscaviarum_WP_143981584.1
Ochromonas_sp_MMETSP1177_c14209_g1_i1.p1
Marmoricola_ginsengisoli_WP_123227456.1
Marmoricola_solisilvae_WP_123232522.1
Nocardioides_sp._K1W22B1_WP_120059289.1
Burkholderia_pyrrocinia_WP_017329154.1
NA_WP_006766049.1
Caballeronia_arvi_WP_061147040.1
Candidatus_Paraburkholderia_sp._4MK11_WP_133193385.1
Paraburkholderia_xenovorans_WP_011494446.1
Robbsia_andropogonis_WP_024904681.1
Alexandrium_fundyense_MMETSP0197_c22448_g1_i1.p1
Prorocentrum_minimum_MMETSP0053_c47908_g1_i1.p1
Pelagodinium_bii_MMETSP1338_c63750_g1_i1.p1
Polarella_glacialis_MMETSP0227_c13604_g1_i1.p2
Aplanochytrium_stocchinoi_MMETSP1347_c6344_g1_i1.p1
NA_WP_145110468.1
Planctomycetes_bacterium_Mal64_WP_146401864.1
Aminiphilus_circumscriptus_WP_026368084.1
Aminomonas_paucivorans_WP_040346296.1
Cloacibacillus_sp._An23_WP_087367052.1
NA_WP_034445192.1
Synergistes_jonesii_WP_037974769.1
Rhodococcus_sp._SAORIC690_WP_105029517.1
Chlorobium_chlorochromatii_WP_011361330.1
Candidatus_Sulfobium_mesophilum_WP_109135932.1
Ammoniphilus_sp._CFH_90114_WP_129200752.1
thiotrophic_endosymbiont_of_Bathymodiolus_puteoserpentis_Logatchev_WP_139683090.1
Paenibacillus_koleovorans_WP_127589092.1
Desulfomicrobium_norvegicum_WP_092194433.1
Candidatus_Desulfosporosinus_infrequens_WP_106801903.1
Peptoclostridium_acidaminophilum_WP_025435131.1
Leptospira_fluminis_WP_135813640.1
Leptospira_inadai_WP_010415646.1
NA_WP_008595637.1
NA_WP_118980156.1
Geovibrio_thiophilus_WP_128466767.1
Seleniivibrio_woodruffii_WP_132874207.1
Clostridium_beijerinckii_WP_017212984.1
Clostridioides_difficile_WP_021384444.1
Cetobacterium_somerae_WP_023051300.1
Kiritimatiellales_bacterium_WP_136082822.1
Lentisphaera_araneosa_WP_007279035.1
Motiliproteus_coralliicola_WP_114696199.1
NA_WP_018704142.1
Pelosinus_fermentans_WP_007955897.1
Clostridium_merdae_WP_085833015.1
NA_WP_009066092.1
Natronorubrum_texcoconense_WP_090309625.1
Bacteroides_xylanolyticus_WP_104439214.1
0.52

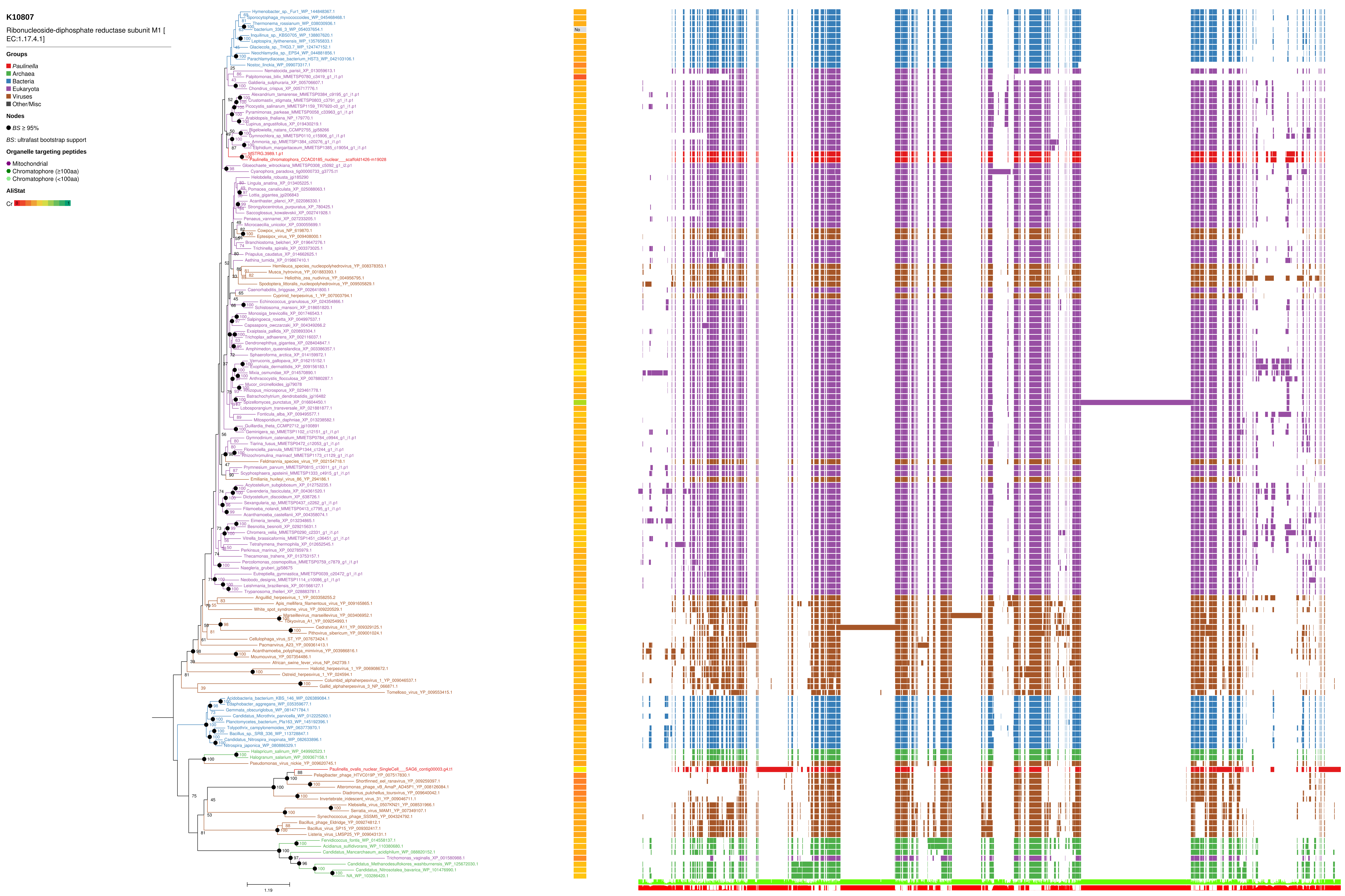

K10807
Ribonucleoside-diphosphate reductase subunit M1 [
EC:1.17.4.1]
Groups
Paulinella
Archaea
Bacteria
Eukaryota
Viruses
Other/Misc
Nodes
BS ≥ 95%
BS: ultrafast bootstrap support
Organelle targeting peptides
Mitochondrial
Chromatophore (≥100aa)
Chromatophore (<100aa)
AliStat
Cr
0
1

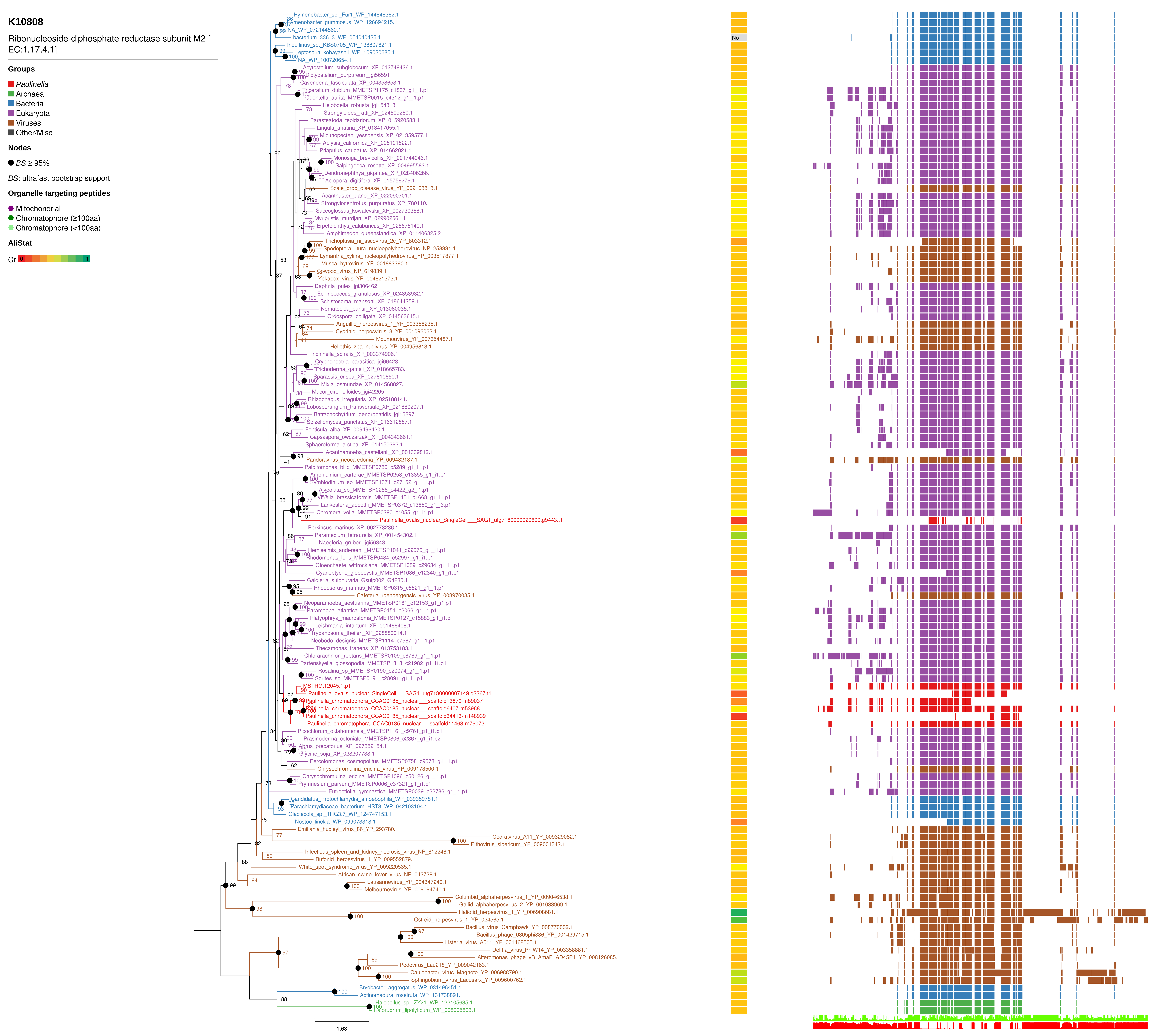
K10808
Ribonucleoside-diphosphate reductase subunit M2 [EC:1.17.4.1]
Groups
Paulinella
Archaea
Bacteria
Eukaryota
Viruses
Other/Misc
Nodes
BS ≥ 95%
BS: ultrafast bootstrap support
Organelle targeting peptides
Mitochondrial
Chromatophore (≥100aa)
Chromatophore (<100aa)
AliStat
Cr 0 1
Hymenobacter_sp._Fur1_WP_144848362.1
Hymenobacter_gummosus_WP_126694215.1
NA_WP_072144860.1
bacterium_336_3_WP_054040425.1
Inquilinus_sp._KBS0705_WP_138807621.1
Leptospira_kobayashii_WP_109020685.1
NA_WP_100720654.1
Acytostelium_subglobosum_XP_012749426.1
Dictyostelium_purpureum_jgi56591
Cavenderia_fasciculata_XP_004358653.1
Triceratium_dubium_MMETSP1175_c1837_g1_i1.p1
Odontella_aurita_MMETSP0015_c4312_g1_i1.p1
Helobdella_robusta_jgi154313
Strongyloides_ratti_XP_024509260.1
Parasteatoda_tepidariorum_XP_015920583.1
Lingula_anatina_XP_013417055.1
Mizuhopecten_yessoensis_XP_021359577.1
Aplysia_californica_XP_005101522.1
Priapulus_caudatus_XP_014662021.1
Monosiga_brevicollis_XP_001744046.1
Salpingoeca_rosetta_XP_004995583.1
Dendronephthya_gigantea_XP_028406266.1
Acropora_digitifera_XP_015756279.1
Scale_drop_disease_virus_YP_009163813.1
Acanthaster_planci_XP_022090701.1
Strongylocentrotus_purpuratus_XP_780110.1
Saccoglossus_kowalevskii_XP_002730368.1
Myripristis_murdjan_XP_029902561.1
Erpetoichthys_calabaricus_XP_028675149.1
Amphimedon_queenslandica_XP_011406825.2
Trichoplusia_ni_ascovirus_2c_YP_803312.1
Spodoptera_litura_nucleopolyhedrovirus_NP_258331.1
Lymantria_xylina_nucleopolyhedrovirus_YP_003517877.1
Musca_hytrovirus_YP_001883390.1
Cowpox_virus_NP_619839.1
Yokapox_virus_YP_004821373.1
Daphnia_pulex_jgi306462
Echinococcus_granulosus_XP_024353982.1
Schistosoma_mansoni_XP_018644259.1
Nematocida_parisii_XP_013060035.1
Ordospora_colligata_XP_014563615.1
Anguillid_herpesvirus_1_YP_003358235.1
Cyprinid_herpesvirus_3_YP_001096062.1
Moumouvirus_YP_007354487.1
Heliothis_zea_nudivirus_YP_004956813.1
Trichinella_spiralis_XP_003374906.1
Cryphonectria_parasitica_jgi66428
Trichoderma_gamsii_XP_018665783.1
Sparassis_crispa_XP_027610650.1
Mixia_osmundae_XP_014568827.1
Mucor_circinelloides_jgi42205
Rhizophagus_irregularis_XP_025188141.1
Lobosporangium_transversale_XP_021880207.1
Batrachochytrium_dendrobatidis_jgi16297
Spizellomyces_punctatus_XP_016612857.1
Fonticula_alba_XP_009496420.1
Capsaspora_owczarzaki_XP_004343661.1
Sphaeroforma_arctica_XP_014150292.1
Acanthamoeba_castellanii_XP_004339812.1
Pandoravirus_neocaledonia_YP_009482187.1
Palpitomonas_bilix_MMETSP0780_c5289_g1_i1.p1
Amphidinium_carterae_MMETSP0258_c13855_g1_i1.p1
Symbiodinium_sp_MMETSP1374_c27152_g1_i1.p1
Alveolata_sp_MMETSP0288_c4422_g2_i1.p1
Vitrella_brassicaformis_MMETSP1451_c1668_g1_i1.p1
Lankesteria_abbottii_MMETSP0372_c13850_g1_i3.p1
Chromera_velia_MMETSP0290_c1055_g1_i1.p1
Paulinella_ovalis_nuclear_SingleCell___SAG1_utg7180000020600.g9443.t1
Perkinsus_marinus_XP_002773236.1
Paramecium_tetraurelia_XP_001454302.1
Naegleria_gruberi_jgi56348
Hemiselmis_andersenii_MMETSP1041_c22070_g1_i1.p1
Rhodomonas_lens_MMETSP0484_c52997_g1_i1.p1
Gloeochaete_wittrockiana_MMETSP1089_c29634_g1_i1.p1
Cyanoptyche_gloeocystis_MMETSP1086_c12340_g1_i1.p1
Galdieria_sulphuraria_Gsulp002_G4230.1
Rhodosorus_marinus_MMETSP0315_c5521_g1_i1.p1
Cafeteria_roenbergensis_virus_YP_003970085.1
Neoparamoeba_aestuarina_MMETSP0161_c12153_g1_i1.p1
Paramoeba_atlantica_MMETSP0151_c2066_g1_i1.p1
Platyophrya_macrostoma_MMETSP0127_c15883_g1_i1.p1
Leishmania_infantum_XP_001466408.1
Trypanosoma_theileri_XP_028880014.1
Neobodo_designis_MMETSP1114_c7987_g1_i1.p1
Thecamonas_trahens_XP_013753183.1
Chlorarachnion_reptans_MMETSP0109_c8769_g1_i1.p1
Partenskyella_glossopodia_MMETSP1318_c21982_g1_i1.p1
Rosalina_sp_MMETSP0190_c20074_g1_i1.p1
Sorites_sp_MMETSP0191_c28091_g1_i1.p1
MSTRG.12045.1.p1
Paulinella_ovalis_nuclear_SingleCell___SAG1_utg7180000007149.g3367.t1
Paulinella_chromatophora_CCAC0185_nuclear___scaffold13870-m89037
Paulinella_chromatophora_CCAC0185_nuclear___scaffold6407-m53968
Paulinella_chromatophora_CCAC0185_nuclear___scaffold34413-m148939
Paulinella_chromatophora_CCAC0185_nuclear___scaffold11463-m79073
Picochlorum_oklahomensis_MMETSP1161_c9761_g1_i1.p1
Prasinoderma_coloniale_MMETSP0806_c2367_g1_i1.p2
Abrus_precatorius_XP_027352154.1
Glycine_soja_XP_028207738.1
Percolomonas_cosmopolitus_MMETSP0758_c9578_g1_i1.p1
Chrysochromulina_ericina_virus_YP_009173500.1
Chrysochromulina_ericina_MMETSP1096_c50126_g1_i1.p1
Prymnesium_parvum_MMETSP0006_c37321_g1_i1.p1
Eutreptiella_gymnastica_MMETSP0039_c22786_g1_i1.p1
Candidatus_Protochlamydia_amoebophila_WP_039359781.1
Parachlamydiaceae_bacterium_HST3_WP_042103104.1
Glaciecola_sp._THG3.7_WP_124747153.1
Nostoc_linckia_WP_099073318.1
Emiliania_huxleyi_virus_86_YP_293780.1
Cedratvirus_A11_YP_009329082.1
Pithovirus_sibericum_YP_009001342.1
Infectious_spleen_and_kidney_necrosis_virus_NP_612246.1
Bufonid_herpesvirus_1_YP_009552879.1
White_spot_syndrome_virus_YP_009220535.1
African_swine_fever_virus_NP_042738.1
Lausannevirus_YP_004347240.1
Melbournevirus_YP_009094740.1
Columbid_alphaherpesvirus_1_YP_009046538.1
Gallid_alphaherpesvirus_2_YP_001033969.1
Haliotid_herpesvirus_1_YP_006908681.1
Ostreid_herpesvirus_1_YP_024565.1
Bacillus_virus_Camphawk_YP_008770002.1
Bacillus_phage_0305phi836_YP_001429715.1
Listeria_virus_A511_YP_001468505.1
Delftia_virus_PhiW14_YP_003358881.1
Alteromonas_phage_vB_AmaP_AD45P1_YP_008126085.1
Podovirus_Lau218_YP_009042163.1
Caulobacter_virus_Magneto_YP_006988790.1
Sphingobium_virus_Lacusarx_YP_009600762.1
Bryobacter_aggregatus_WP_031496451.1
Actinomadura_roseirufa_WP_131738891.1
Halobellus_sp._ZY21_WP_122105635.1
Halorubrum_lipolyticum_WP_008005803.1
1.63
No

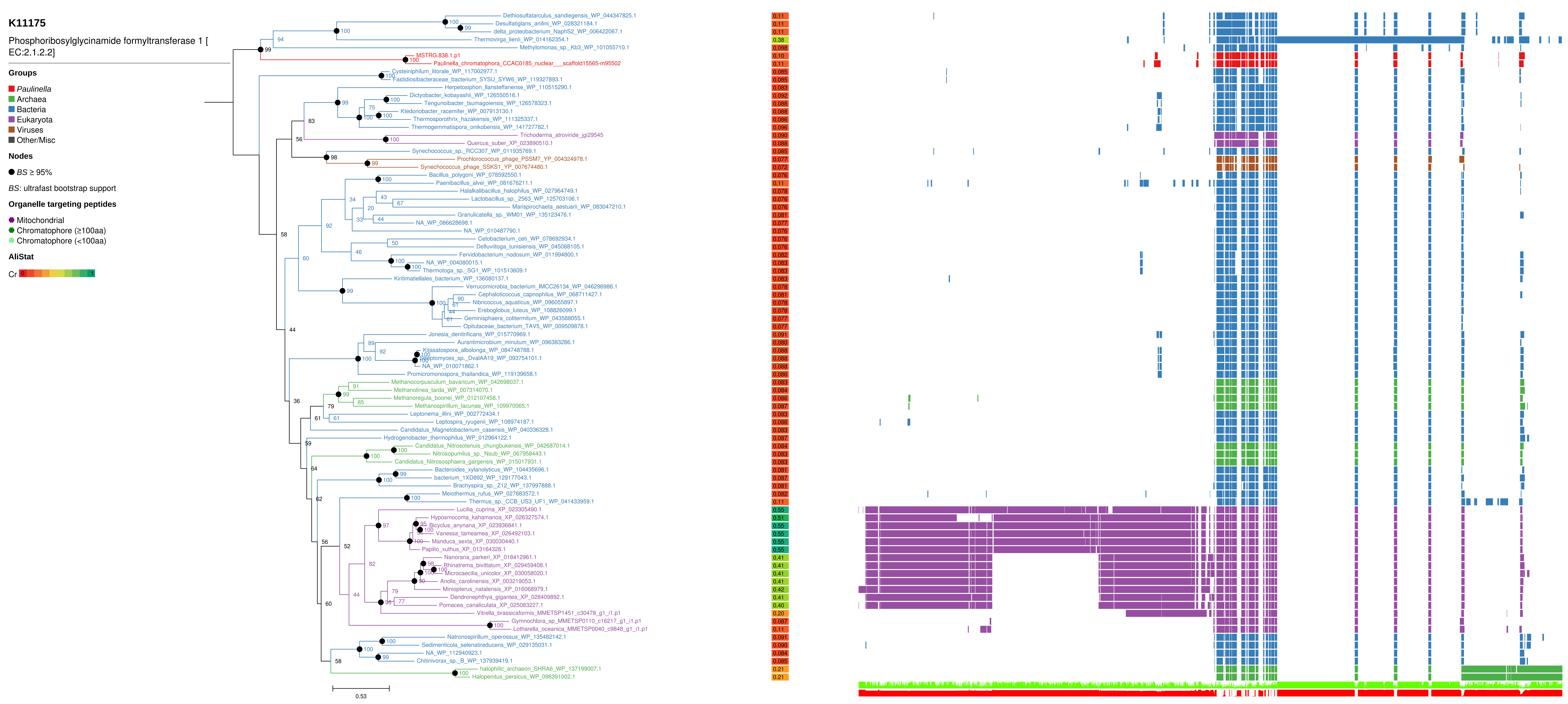

K11175
Phosphoribosylglycinamide formyltransferase 1 [EC:2.1.2.2]
Groups
Paulinella
Archaea
Bacteria
Eukaryota
Viruses
Other/Misc
Nodes
BS ≥ 95%
BS: ultrafast bootstrap support
Organelle targeting peptides
Mitochondrial
Chromatophore (≥100aa)
Chromatophore (<100aa)
AliStat
Cr
Dethiosulfatarculus_sandiegensis_WP_044347825.1
Desulfatiglans_anilini_WP_028321184.1
delta_proteobacterium_NaphS2_WP_006422067.1
Thermovirga_lienii_WP_014162354.1
Methylomonas_sp._Kb3_WP_101055710.1
MSTRG.838.1.p1
Paulinella_chromatophora_CCAC0185_nuclear___scaffold15565-m95502
Cysteiniphilum_litorale_WP_117002977.1
Fastidiosibacteraceae_bacterium_SYSU_SYW6_WP_119327893.1
Herpetosiphon_llansteffanense_WP_110515290.1
Dictyobacter_kobayashii_WP_126550156.1
Tengunoibacter_tsumagoiensis_WP_126578323.1
Ktedonobacter_racemifer_WP_007913130.1
Thermosporothrix_hazakensis_WP_111325337.1
Thermogemmatispora_onikobensis_WP_141727782.1
Trichoderma_atroviride_jgi29545
Quercus_suber_XP_023890510.1
Synechococcus_sp._RCC307_WP_011935769.1
Prochlorococcus_phage_PSSM7_YP_004324978.1
Synechococcus_phage_SSKS1_YP_007674480.1
Bacillus_polygoni_WP_078592550.1
Paenibacillus_alvei_WP_081676211.1
Halalkalibacillus_halophilus_WP_027964749.1
Lactobacillus_sp._2563_WP_125703106.1
Marispirochaeta_aestuarii_WP_083047210.1
Granulicatella_sp._WM01_WP_135123476.1
NA_WP_086628698.1
NA_WP_010487790.1
Cetobacterium_ceti_WP_078692934.1
Defluviitoga_tunisiensis_WP_045088105.1
Fervidobacterium_nodosum_WP_011994800.1
NA_WP_004080015.1
Thermotoga_sp._SG1_WP_101513609.1
Kiritimatiellales_bacterium_WP_136080137.1
Verrucomicrobia_bacterium_IMCC26134_WP_046296986.1
Cephaloticoccus_capnophilus_WP_068711427.1
Nibricoccus_aquaticus_WP_096055897.1
Ereboglobus_luteus_WP_108826099.1
Geminisphaera_colitermitum_WP_043588055.1
Opitutaceae_bacterium_TAV5_WP_009509878.1
Jonesia_denitrificans_WP_015770969.1
Aurantimicrobium_minutum_WP_096383286.1
Kitasatospora_albolonga_WP_084748788.1
Streptomyces_sp._DvalAA19_WP_093754101.1
NA_WP_010071862.1
Promicromonospora_thailandica_WP_119139658.1
Methanocorpusculum_bavaricum_WP_042698037.1
Methanolinea_tarda_WP_007314070.1
Methanoregula_boonei_WP_012107458.1
Methanospirillum_lacunae_WP_109970065.1
Leptonema_illini_WP_002772434.1
Leptospira_ryugenii_WP_108974187.1
Candidatus_Magnetobacterium_casensis_WP_040336328.1
Hydrogenobacter_thermophilus_WP_012964122.1
Candidatus_Nitrosotenuis_chungbukensis_WP_042687014.1
Nitrosopumilus_sp._Nsub_WP_067958443.1
Candidatus_Nitrososphaera_gargensis_WP_015017931.1
Bacteroides_xylanolyticus_WP_104435696.1
bacterium_1XD892_WP_129177043.1
Brachyspira_sp._Z12_WP_137997888.1
Meiothermus_rufus_WP_027883572.1
Thermus_sp._CCB_US3_UF1_WP_041433959.1
Lucilia_cuprina_XP_023305490.1
Hyposmocoma_kahamanoa_XP_026327574.1
Bicyclus_anynana_XP_023936841.1
Vanessa_tameamea_XP_026492103.1
Manduca_sexta_XP_030030440.1
Papilio_xuthus_XP_013164328.1
Nanorana_parkeri_XP_018412961.1
Rhinatrema_bivittatum_XP_029459408.1
Microcaecilia_unicolor_XP_030058020.1
Anolis_carolinensis_XP_003219053.1
Miniopterus_natalensis_XP_016068979.1
Dendronephthya_gigantea_XP_028409892.1
Pomacea_canaliculata_XP_025083227.1
Vitrella_brassicaformis_MMETSP1451_c30478_g1_i1.p1
Gymnochlora_sp_MMETSP0110_c16217_g1_i1.p1
Lotharella_oceanica_MMETSP0040_c9848_g1_i1.p1
Natronospirillum_operosus_WP_135482142.1
Sedimenticola_selenatireducens_WP_029135031.1
NA_WP_112940923.1
Chitinivorax_sp._B_WP_137939419.1
halophilic_archaeon_SHRA6_WP_137199007.1
Halopenitus_persicus_WP_096391002.1
0.53

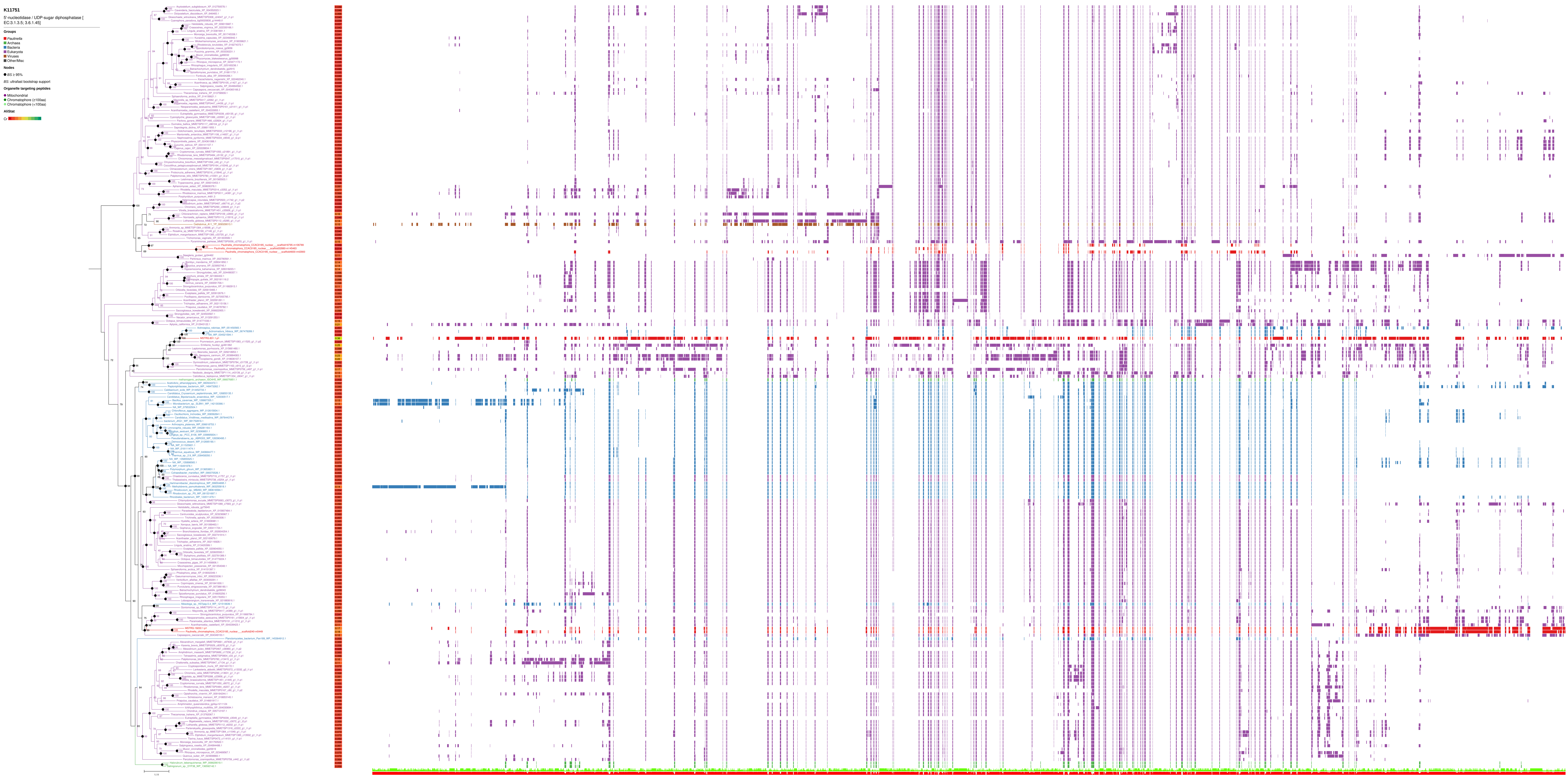
K11751
5'-nucleotidase / UDP-sugar diphosphatase [
EC:3.1.3.5; 3.6.1.45]
Groups
Paulinella
Archaea
Bacteria
Eukaryota
Viruses
Other/Misc
Nodes
BS ≥ 95%
BS: ultrafast bootstrap support
Organelle targeting peptides
Mitochondrial
Chromatophore (≥100aa)
Chromatophore (<100aa)
AliStat
Cr 0 1

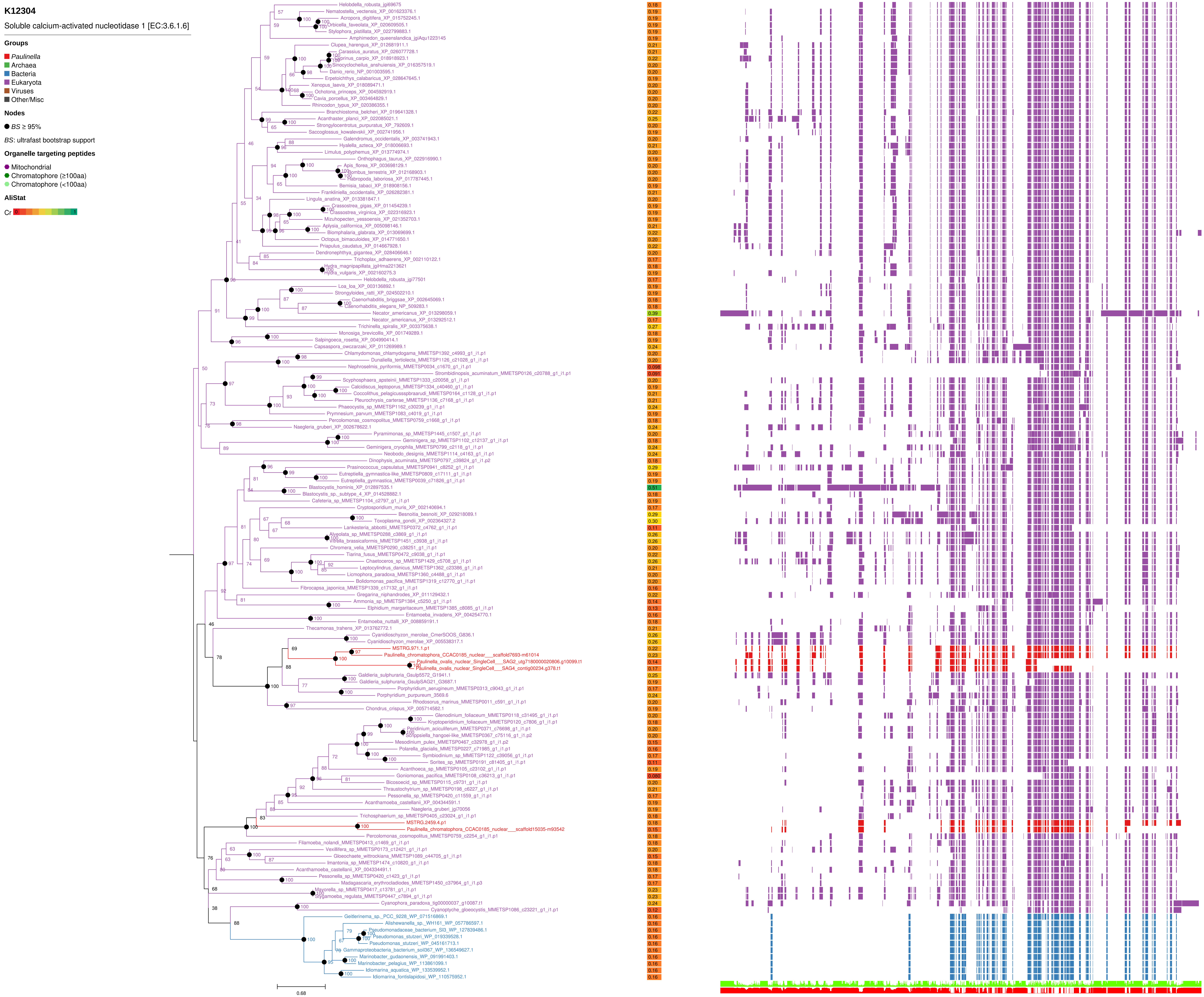
K12304
Soluble calcium-activated nucleotidase 1 [EC:3.6.1.6]
Groups
Paulinella
Archaea
Bacteria
Eukaryota
Viruses
Other/Misc
Nodes
BS ≥ 95%
BS: ultrafast bootstrap support
Organelle targeting peptides
Mitochondrial
Chromatophore (≥100aa)
Chromatophore (<100aa)
AliStat
Cr
0
1
0.68

K13293

cAMP-specific phosphodiesterase 4 [EC:3.1.4.53]

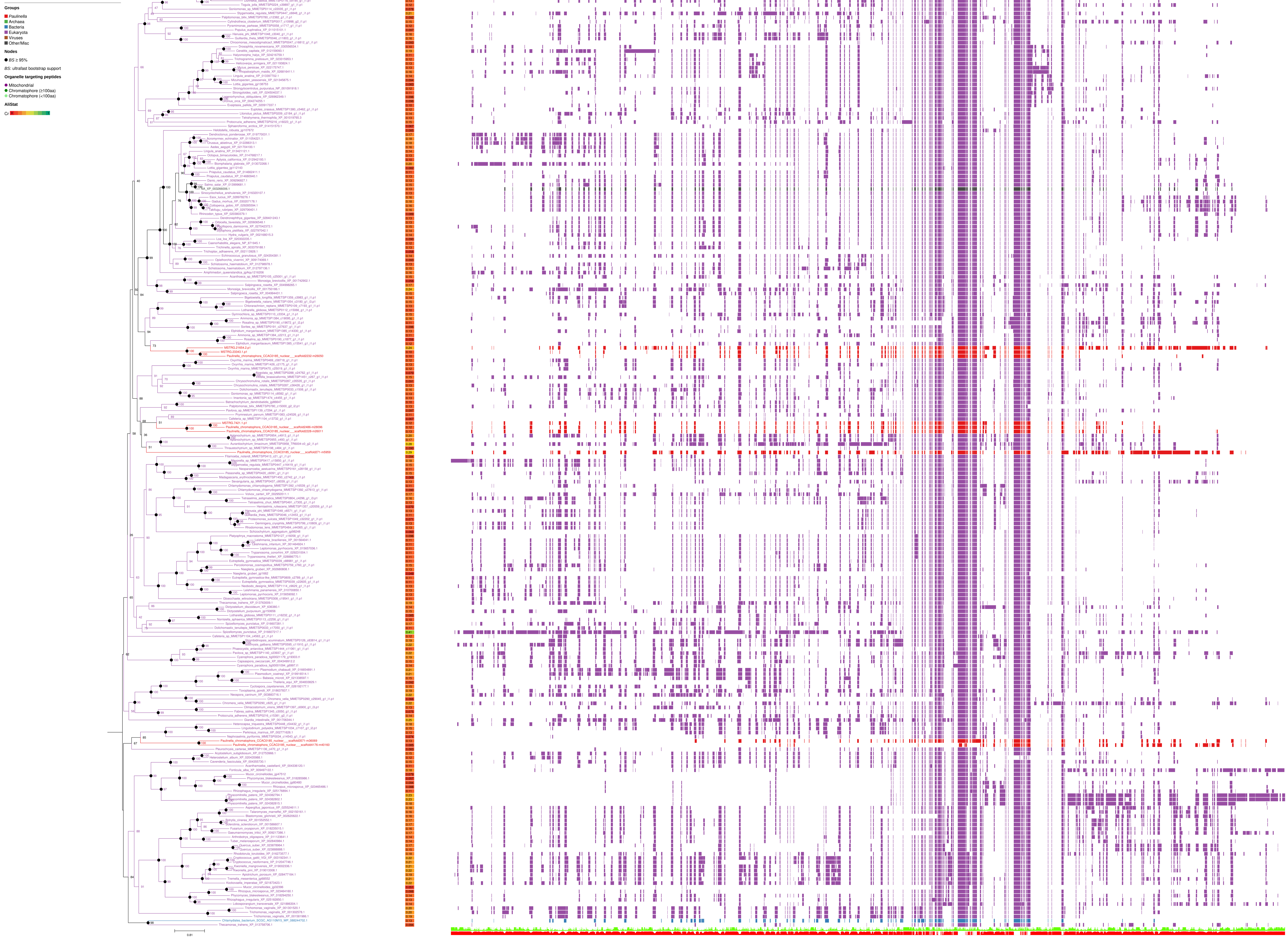

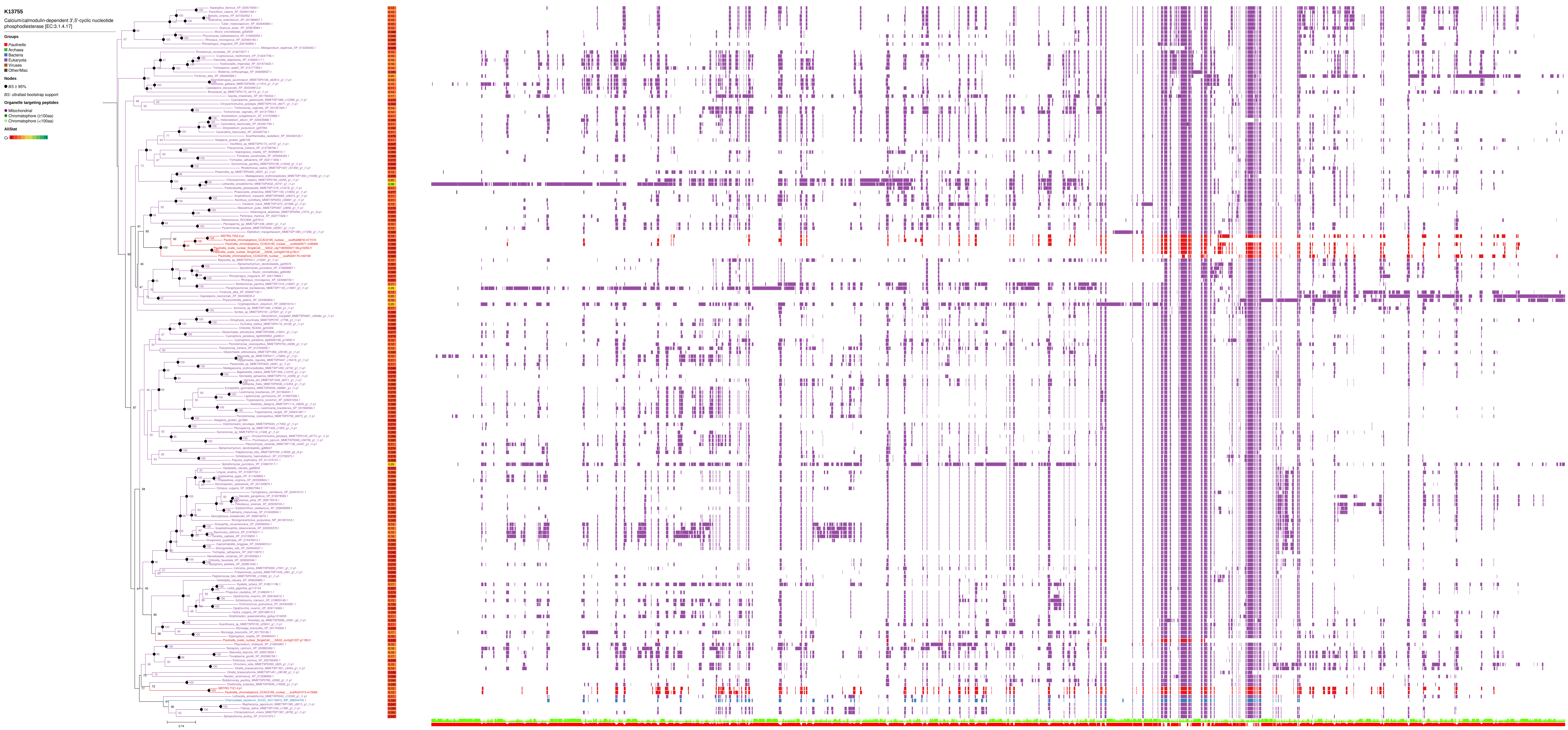

K13755
Calcium/calmodulin-dependent 3',5'-cyclic nucleotide phosphodiesterase [EC:3.1.4.17]
Groups
Paulinella
Archaea
Bacteria
Eukaryota
Viruses
Other/Misc
Nodes
BS ≥ 95%
BS: ultrafast bootstrap support
Organelle targeting peptides
Mitochondrial
Chromatophore (≥100aa)
Chromatophore (<100aa)
AliStat
Cr 0 1
0.74

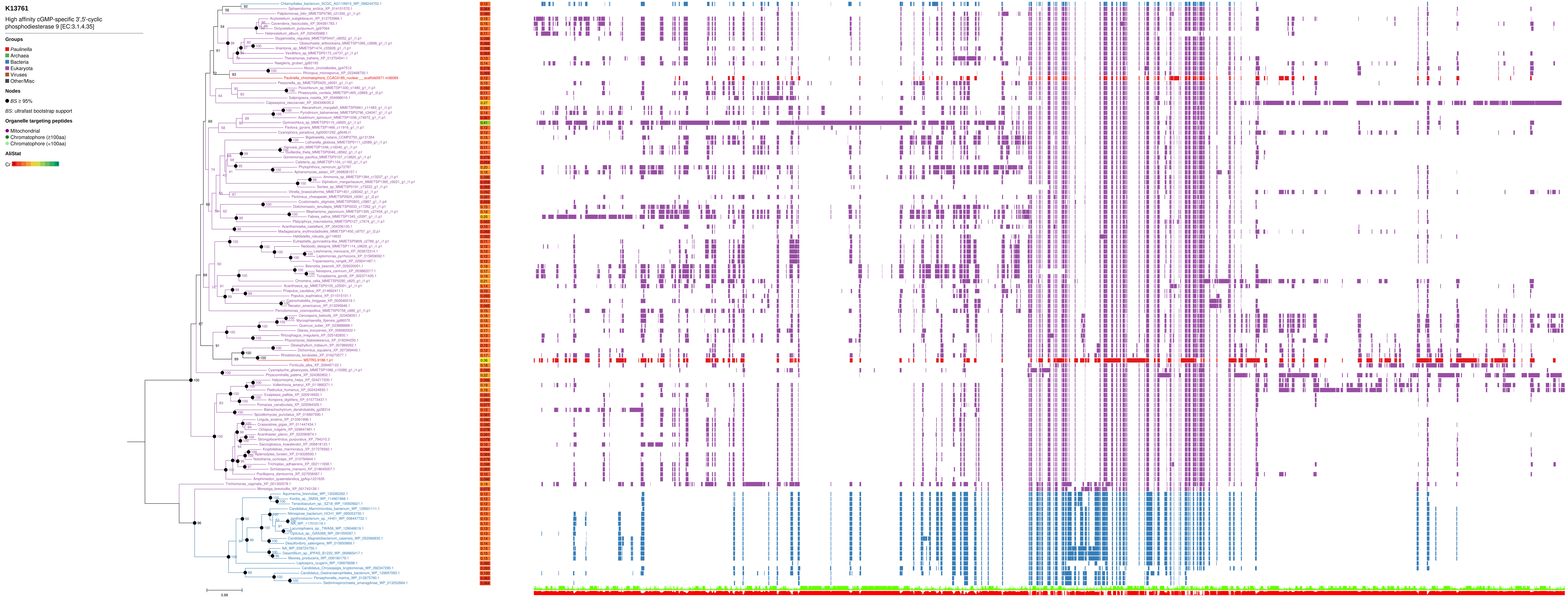

K13761
High affinity cGMP-specific 3',5'-cyclic phosphodiesterase 9 [EC:3.1.4.35]
Groups
Paulinella
Archaea
Bacteria
Eukaryota
Viruses
Other/Misc
Nodes
BS ≥ 95%
BS: ultrafast bootstrap support
Organelle targeting peptides
Mitochondrial
Chromatophore (≥100aa)
Chromatophore (<100aa)
AliStat
Cr 0 1

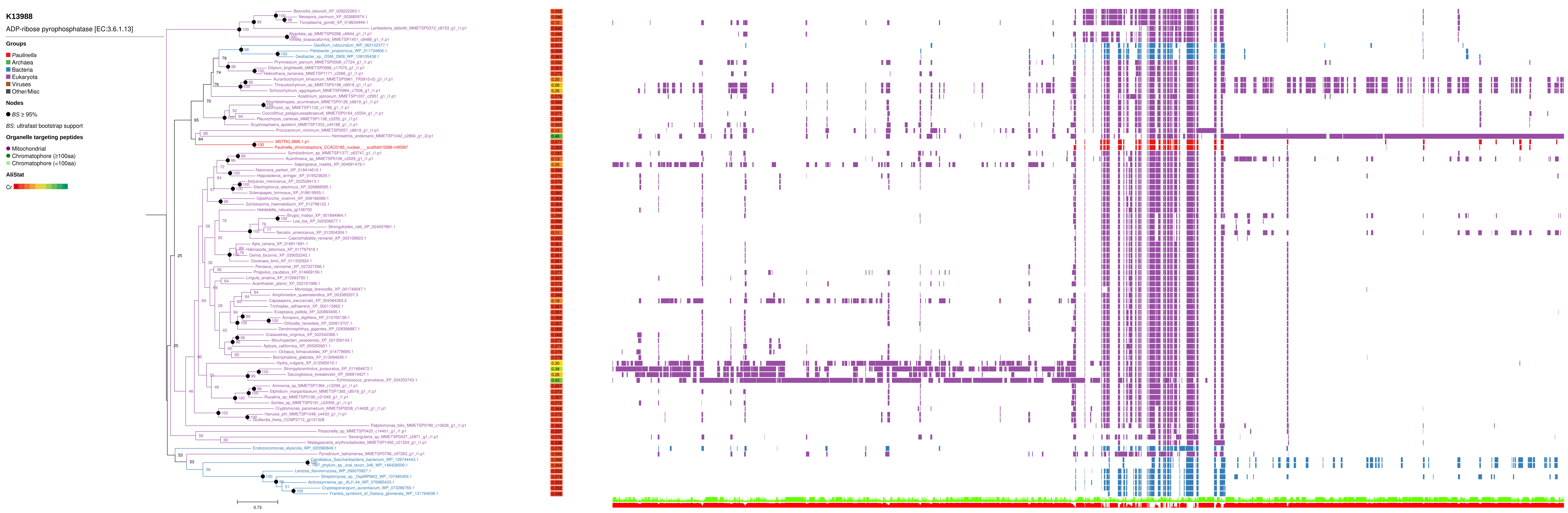
K13988
ADP-ribose pyrophosphatase [EC:3.6.1.13]
Groups
Paulinella
Archaea
Bacteria
Eukaryota
Viruses
Other/Misc
Nodes
BS ≥ 95%
BS: ultrafast bootstrap support
Organelle targeting peptides
Mitochondrial
Chromatophore (≥100aa)
Chromatophore (<100aa)
AliStat
Cr
0.73

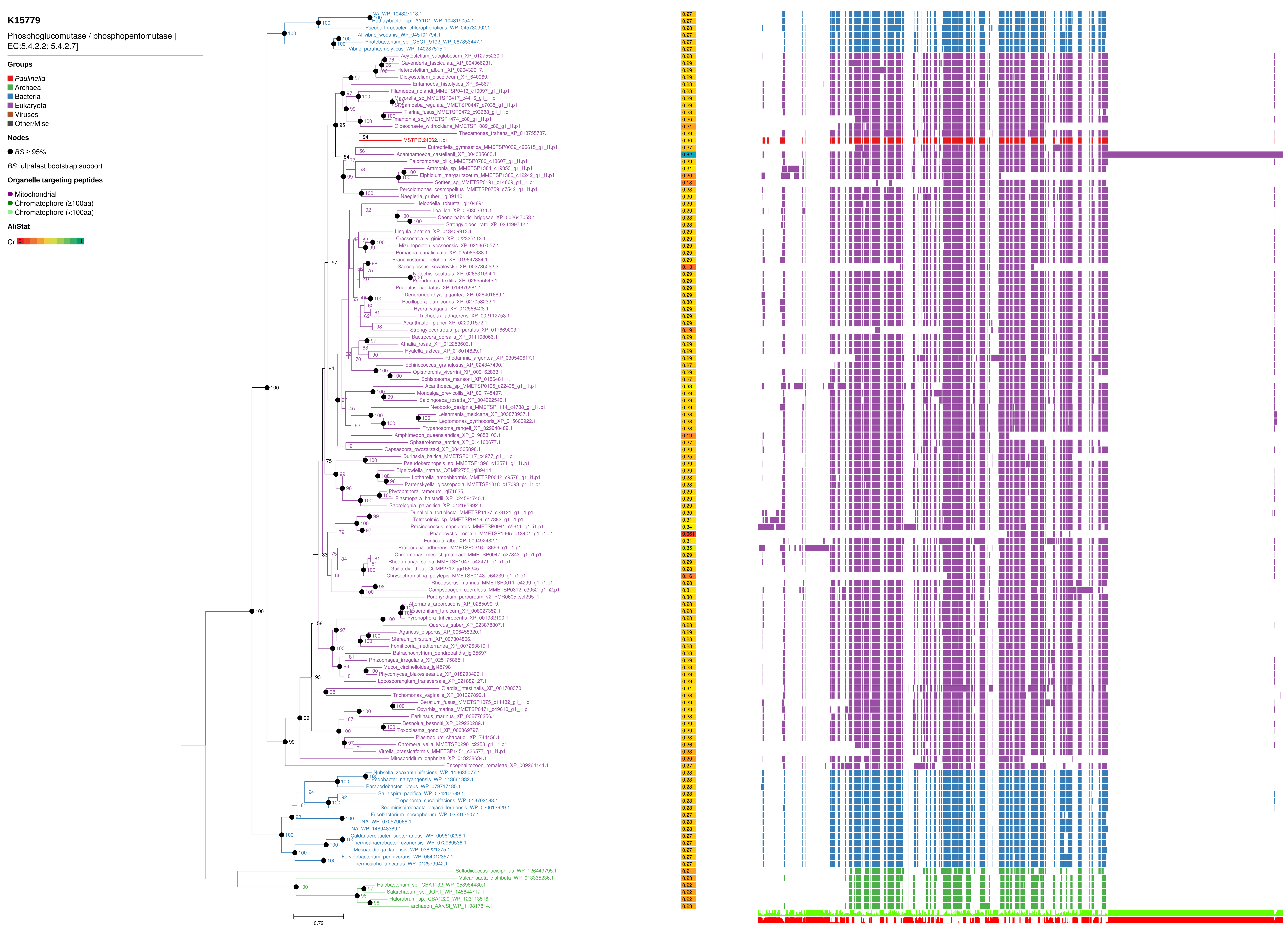

K15779
Phosphoglucomutase / phosphopentomutase [EC:5.4.2.2; 5.4.2.7]
Groups
Paulinella
Archaea
Bacteria
Eukaryota
Viruses
Other/Misc
Nodes
BS ≥ 95%
BS: ultrafast bootstrap support
Organelle targeting peptides
Mitochondrial
Chromatophore (≥100aa)
Chromatophore (<100aa)
AliStat
Cr
NA_WP_104327113.1
Rathayibacter_sp._AY1D1_WP_104319054.1
Pseudarthrobacter_chlorophenolicus_WP_045730902.1
Aliivibrio_wodanis_WP_045101794.1
Photobacterium_sp._CECT_9192_WP_087853447.1
Vibrio_parahaemolyticus_WP_140287515.1
Acytostelium_subglobosum_XP_012755230.1
Cavenderia_fasciculata_XP_004366231.1
Heterostelium_album_XP_020432017.1
Dictyostelium_discoideum_XP_640969.1
Entamoeba_histolytica_XP_648671.1
Filamoeba_nolandi_MMETSP0413_c19097_g1_i1.p1
Mayorella_sp_MMETSP0417_c4416_g1_i1.p1
Stygamoeba_regulata_MMETSP0447_c7035_g1_i1.p1
Tiarina_fusus_MMETSP0472_c93688_g1_i1.p1
Imantonia_sp_MMETSP1474_c80_g1_i1.p1
Gloeochaete_wittrockiana_MMETSP1089_c86_g1_i1.p1
Thecamonas_trahens_XP_013755787.1
MSTRG.24662.1.p1
Eutreptiella_gymnastica_MMETSP0039_c26615_g1_i1.p1
Acanthamoeba_castellanii_XP_004335683.1
Palpitomonas_bilix_MMETSP0780_c13607_g1_i1.p1
Ammonia_sp_MMETSP1384_c19353_g1_i1.p1
Elphidium_margaritaceum_MMETSP1385_c12242_g1_i1.p1
Sorites_sp_MMETSP0191_c14869_g1_i1.p1
Percolomonas_cosmopolitus_MMETSP0759_c7542_g1_i1.p1
Naegleria_gruberi_jgi39110
Helobdella_robusta_jgi104891
Loa_loa_XP_020303311.1
Caenorhabditis_briggsae_XP_002647053.1
Strongyloides_ratti_XP_024499742.1
Lingula_anatina_XP_013409913.1
Crassostrea_virginica_XP_022325113.1
Mizuhopecten_yessoensis_XP_021367057.1
Pomacea_canaliculata_XP_025085388.1
Branchiostoma_belcheri_XP_019647384.1
Saccoglossus_kowalevskii_XP_002735052.2
Notechis_scutatus_XP_026531094.1
Pseudonaja_textilis_XP_026555645.1
Priapulus_caudatus_XP_014675581.1
Dendronephthya_gigantea_XP_028401689.1
Pocillopora_damicornis_XP_027053232.1
Hydra_vulgaris_XP_012566428.1
Trichoplax_adhaerens_XP_002112753.1
Acanthaster_planci_XP_022091572.1
Strongylocentrotus_purpuratus_XP_011669003.1
Bactrocera_dorsalis_XP_011198066.1
Athalia_rosae_XP_012253603.1
Hyalella_azteca_XP_018014829.1
Rhodamnia_argentea_XP_030540617.1
Echinococcus_granulosus_XP_024347490.1
Opisthorchis_viverrini_XP_009162863.1
Schistosoma_mansoni_XP_018648111.1
Acanthoeca_sp_MMETSP0105_c22438_g1_i1.p1
Monosiga_brevicollis_XP_001745497.1
Salpingoeca_rosetta_XP_004992540.1
Neobodo_designis_MMETSP1114_c4788_g1_i1.p1
Leishmania_mexicana_XP_003878937.1
Leptomonas_pyrrhocoris_XP_015660922.1
Trypanosoma_rangeli_XP_029240489.1
Amphimedon_queenslandica_XP_019858103.1
Sphaeroforma_arctica_XP_014160677.1
Capsaspora_owczarzaki_XP_004365898.1
Durinskia_baltica_MMETSP0117_c4977_g1_i1.p1
Pseudokeronopsis_sp_MMETSP1396_c13571_g1_i1.p1
Bigelowiella_natans_CCMP2755_jgi89414
Lotharella_amoebiformis_MMETSP0042_c9578_g1_i1.p1
Partenskyella_glossopodia_MMETSP1318_c17093_g1_i1.p1
Phytophthora_ramorum_jgi71625
Plasmopara_halstedii_XP_024581740.1
Saprolegnia_parasitica_XP_012195992.1
Dunaliella_tertiolecta_MMETSP1127_c23121_g1_i1.p1
Tetraselmis_sp_MMETSP0419_c17882_g1_i1.p1
Prasinococcus_capsulatus_MMETSP0941_c5611_g1_i1.p1
Phaeocystis_cordata_MMETSP1465_c13401_g1_i1.p1
Fonticula_alba_XP_009492482.1
Protocruzia_adherens_MMETSP0216_c8699_g1_i1.p1
Chroomonas_mesostigmatica_MMETSP0047_c27343_g1_i1.p1
Rhodomonas_salina_MMETSP1047_c42471_g1_i1.p1
Guillardia_theta_CCMP2712_jgi166345
Chrysochromulina_polylepis_MMETSP0143_c64239_g1_i1.p1
Rhodosorus_marinus_MMETSP0011_c4299_g1_i1.p1
Compsopogon_coeruleus_MMETSP0312_c3052_g1_i2.p1
Porphyridium_purpureum_v2_POR0605..scf295_1
Alternaria_arborescens_XP_028509919.1
Exserohilum_turcicum_XP_008027352.1
Pyrenophora_triticirepentis_XP_001932190.1
Quercus_suber_XP_023878807.1
Agaricus_bisporus_XP_006458320.1
Stereum_hirsutum_XP_007304806.1
Fomitiporia_mediterranea_XP_007263819.1
Batrachochytrium_dendrobatidis_jgi35697
Rhizophagus_irregularis_XP_025175865.1
Mucor_circinelloides_jgi45798
Phycomyces_blakesleeanus_XP_018293429.1
Lobosporangium_transversale_XP_021882127.1
Giardia_intestinalis_XP_001708370.1
Trichomonas_vaginalis_XP_001327899.1
Ceratium_fusus_MMETSP1075_c11482_g1_i1.p1
Oxyrrhis_marina_MMETSP0471_c49610_g1_i1.p1
Perkinsus_marinus_XP_002778256.1
Besnoitia_besnoiti_XP_029220289.1
Toxoplasma_gondii_XP_002369797.1
Plasmodium_chabaudi_XP_744456.1
Chromera_velia_MMETSP0290_c2253_g1_i1.p1
Vitrella_brassicaformis_MMETSP1451_c36577_g1_i1.p1
Mitosporidium_daphniae_XP_013238634.1
Encephalitozoon_romaleae_XP_009264141.1
Nubsella_zeaxanthinifaciens_WP_113635077.1
Pedobacter_nanyangensis_WP_113661332.1
Parapedobacter_luteus_WP_079717185.1
Salinispira_pacifica_WP_024267589.1
Treponema_succinifaciens_WP_013702188.1
Sediminispirochaeta_bajacaliforniensis_WP_020613929.1
Fusobacterium_necrophorum_WP_035917507.1
NA_WP_070579066.1
NA_WP_148948389.1
Caldanaerobacter_subterraneus_WP_009610298.1
Thermoanaerobacter_uzonensis_WP_072969536.1
Mesoaciditoga_lauensis_WP_036221275.1
Fervidobacterium_pennivorans_WP_064012357.1
Thermosipho_africanus_WP_012579942.1
Sulfodiicoccus_acidiphilus_WP_126449795.1
Vulcanisaeta_distributa_WP_013335236.1
Halobacterium_sp._CBA1132_WP_058984430.1
Salarchaeum_sp._JOR1_WP_145844717.1
Halorubrum_sp._CBA1229_WP_123113516.1
archaeon_AArcSl_WP_119817814.1
0.72

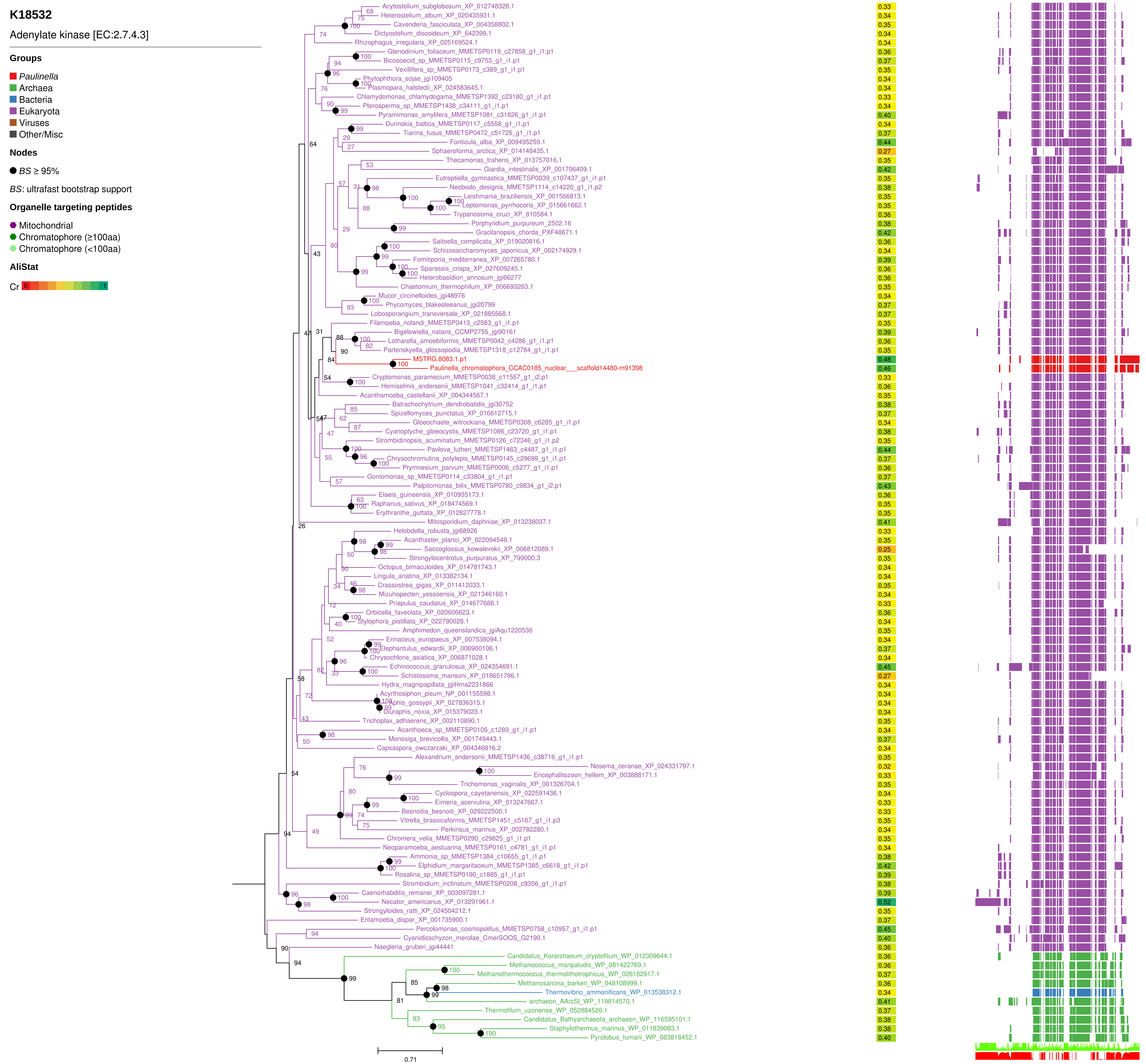
K18532
Adenylate kinase [EC:2.7.4.3]
Groups
Paulinella
Archaea
Bacteria
Eukaryota
Viruses
Other/Misc
Nodes
BS ≥ 95%
BS: ultrafast bootstrap support
Organelle targeting peptides
Mitochondrial
Chromatophore (≥100aa)
Chromatophore (<100aa)
AliStat
Cr 0 1
Acytostelium_subglobosum_XP_012748328.1
Heterostelium_album_XP_020435931.1
Cavenderia_fasciculata_XP_004358802.1
Dictyostelium_discoideum_XP_642399.1
Rhizophagus_irregularis_XP_025169524.1
Glenodinium_foliaceum_MMETSP0119_c27858_g1_i1.p1
Bicosoecid_sp_MMETSP0115_c9753_g1_i1.p1
Vexillifera_sp_MMETSP0173_c389_g1_i1.p1
Phytophthora_sojae_jgi109405
Plasmopara_halstedii_XP_024583645.1
Chlamydomonas_chlamydogama_MMETSP1392_c23180_g1_i1.p1
Pterosperma_sp_MMETSP1438_c34111_g1_i1.p1
Pyramimonas_amylifera_MMETSP1081_c31826_g1_i1.p1
Durinskia_baltica_MMETSP0117_c5558_g1_i1.p1
Tiarina_fusus_MMETSP0472_c51725_g1_i1.p1
Fonticula_alba_XP_009495259.1
Sphaeroforma_arctica_XP_014148435.1
Thecamonas_trahens_XP_013757016.1
Giardia_intestinalis_XP_001706409.1
Eutreptiella_gymnastica_MMETSP0039_c107437_g1_i1.p1
Neobodo_designis_MMETSP1114_c14220_g1_i1.p2
Leishmania_braziliensis_XP_001566813.1
Leptomonas_pyrrhocoris_XP_015661662.1
Trypanosoma_cruzi_XP_810584.1
Porphyridium_purpureum_2502.16
Gracilariopsis_chorda_PXF48671.1
Saitoella_complicata_XP_019020816.1
Schizosaccharomyces_japonicus_XP_002174929.1
Fomitiporia_mediterranea_XP_007265780.1
Sparassis_crispa_XP_027609245.1
Heterobasidion_annosum_jgi66277
Chaetomium_thermophilum_XP_006693263.1
Mucor_circinelloides_jgi48976
Phycomyces_blakesleeanus_jgi20799
Lobosporangium_transversale_XP_021885568.1
Filamoeba_nolandi_MMETSP0413_c2583_g1_i1.p1
Bigelowiella_natans_CCMP2755_jgi90161
Lotharella_amoebiformis_MMETSP0042_c4286_g1_i1.p1
Partenskyella_glossopodia_MMETSP1318_c12784_g1_i1.p1
MSTRG.8083.1.p1
Paulinella_chromatophora_CCAC0185_nuclear___scaffold14480-m91398
Cryptomonas_paramecium_MMETSP0038_c11557_g1_i2.p1
Hemiselmis_andersenii_MMETSP1041_c32414_g1_i1.p1
Acanthamoeba_castellanii_XP_004344567.1
Batrachochytrium_dendrobatidis_jgi30752
Spizellomyces_punctatus_XP_016612715.1
Gloeochaete_witrockiana_MMETSP0308_c6285_g1_i1.p1
Cyanoptyche_gloeocystis_MMETSP1086_c23720_g1_i1.p1
Strombidinopsis_acuminatum_MMETSP0126_c72346_g1_i1.p2
Pavlova_lutheri_MMETSP1463_c4487_g1_i1.p1
Chrysochromulina_polylepis_MMETSP0145_c29699_g1_i1.p1
Prymnesium_parvum_MMETSP0006_c5277_g1_i1.p1
Goniomonas_sp_MMETSP0114_c33804_g1_i1.p1
Palpitomonas_bilix_MMETSP0780_c9834_g1_i2.p1
Elaeis_guineensis_XP_010935173.1
Raphanus_sativus_XP_018474569.1
Erythranthe_guttata_XP_012827778.1
Mitosporidium_daphniae_XP_013238037.1
Helobdella_robusta_jgi68926
Acanthaster_planci_XP_022094549.1
Saccoglossus_kowalevskii_XP_006812089.1
Strongylocentrotus_purpuratus_XP_799000.3
Octopus_bimaculoides_XP_014781743.1
Lingula_anatina_XP_013382134.1
Crassostrea_gigas_XP_011412033.1
Mizuhopecten_yessoensis_XP_021346160.1
Priapulus_caudatus_XP_014677688.1
Orbicella_faveolata_XP_020606623.1
Stylophora_pistillata_XP_022790028.1
Amphimedon_queenslandica_jgiAqu1220536
Erinaceus_europaeus_XP_007538094.1
Elephantulus_edwardii_XP_006900106.1
Chrysochloris_asiatica_XP_006871028.1
Echinococcus_granulosus_XP_024354691.1
Schistosoma_mansoni_XP_018651786.1
Hydra_magnipapillata_jgiHma2231866
Acyrthosiphon_pisum_NP_001155598.1
Aphis_gossypii_XP_027836315.1
Diuraphis_noxia_XP_015379023.1
Trichoplax_adhaerens_XP_002110890.1
Acanthoeca_sp_MMETSP0105_c1289_g1_i1.p1
Monosiga_brevicollis_XP_001749443.1
Capsaspora_owczarzaki_XP_004346816.2
Alexandrium_andersonii_MMETSP1436_c38716_g1_i1.p1
Nosema_ceranae_XP_024331797.1
Encephalitozoon_hellem_XP_003888171.1
Trichomonas_vaginalis_XP_001326704.1
Cyclospora_cayetanensis_XP_022591436.1
Eimeria_acervulina_XP_013247667.1
Besnoitia_besnoiti_XP_029222500.1
Vitrella_brassicaformis_MMETSP1451_c5167_g1_i1.p3
Perkinsus_marinus_XP_002782280.1
Chromera_velia_MMETSP0290_c29825_g1_i1.p1
Neoparamoeba_aestuarina_MMETSP0161_c4781_g1_i1.p1
Ammonia_sp_MMETSP1384_c10655_g1_i1.p1
Elphidium_margaritaceum_MMETSP1385_c6618_g1_i1.p1
Rosalina_sp_MMETSP0190_c1885_g1_i1.p1
Strombidium_inclinatum_MMETSP0208_c9356_g1_i1.p1
Caenorhabditis_remanei_XP_003097281.1
Necator_americanus_XP_013291961.1
Strongyloides_ratti_XP_024504212.1
Entamoeba_dispar_XP_001735900.1
Percolomonas_cosmopolitus_MMETSP0758_c10957_g1_i1.p1
Cyanidioschyzon_merolae_CmerSOOS_G2190.1
Naegleria_gruberi_jgi44441
Candidatus_Korarchaeum_cryptofilum_WP_012309644.1
Methanococcus_maripaludis_WP_081422769.1
Methanothermococcus_thermolithotrophicus_WP_026182917.1
Methanosarcina_barkeri_WP_048108999.1
Thermovibrio_ammonificans_WP_013538312.1
archaeon_AArcSl_WP_119814570.1
Thermofilum_uzonense_WP_052884520.1
Candidatus_Bathyarchaeota_archaeon_WP_116595101.1
Staphylothermus_marinus_WP_011839083.1
Pyrolobus_fumarii_WP_083818452.1
0.71

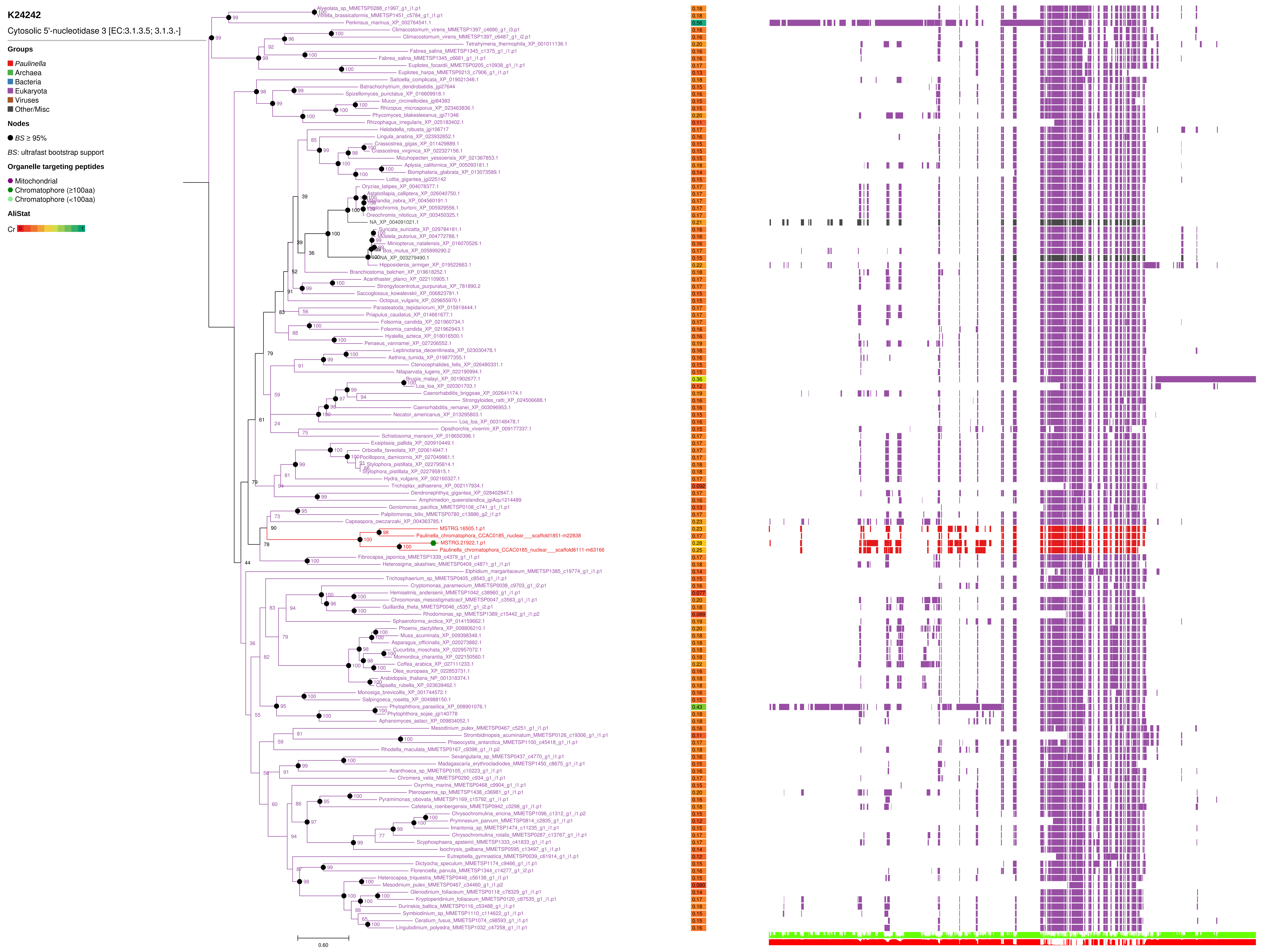
K24242
Cytosolic 5'-nucleotidase 3 [EC:3.1.3.5; 3.1.3.-]
Groups
Paulinella
Archaea
Bacteria
Eukaryota
Viruses
Other/Misc
Nodes
BS ≥ 95%
BS: ultrafast bootstrap support
Organelle targeting peptides
Mitochondrial
Chromatophore (≥100aa)
Chromatophore (<100aa)
AliStat
Cr
0.60

K01663

Imidazole glycerol-phosphate synthase [EC:4.3.2.10]

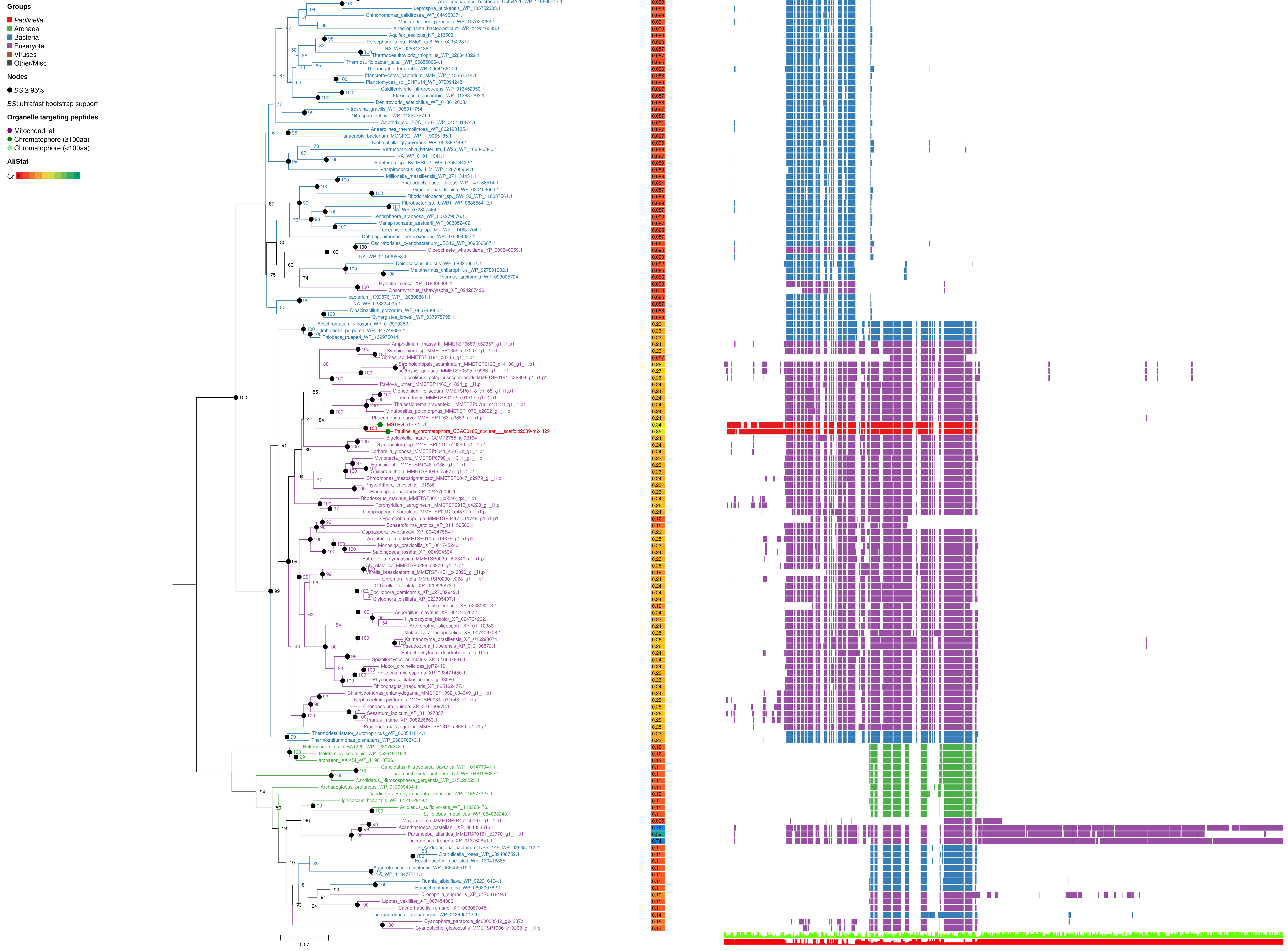

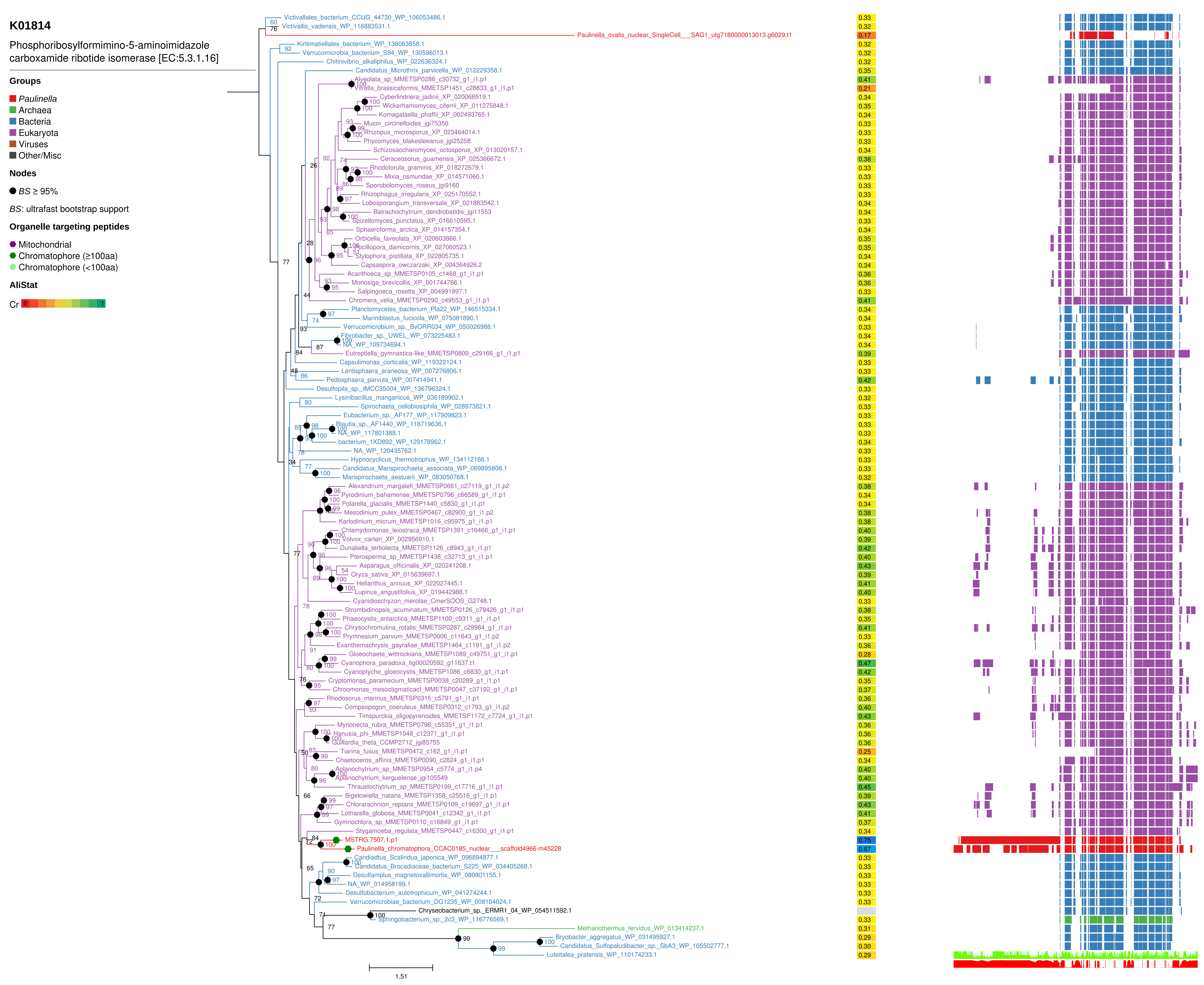

K01814
Phosphoribosylformimino-5-aminoimidazole carboxamide ribotide isomerase [EC:5.3.1.16]
Groups
Paulinella
Archaea
Bacteria
Eukaryota
Viruses
Other/Misc
Nodes
BS ≥ 95%
BS: ultrafast bootstrap support
Organelle targeting peptides
Mitochondrial
Chromatophore (≥100aa)
Chromatophore (<100aa)
AliStat
Cr 0 1
Victivallales_bacterium_CCUG_44730_WP_106053486.1
Victivallis_vadensis_WP_116883531.1
Paulinella_ovalis_nuclear_SingleCell___SAG1_utg7180000013013.g6029.t1
Kiritimatiellales_bacterium_WP_136063858.1
Verrucomicrobia_bacterium_S94_WP_130596013.1
Chitinivibrio_alkaliphilus_WP_022636324.1
Candidatus_Microthrix_parvicella_WP_012229358.1
Alveolata_sp_MMETSP0288_c30732_g1_i1.p1
Vitrella_brassicaformis_MMETSP1451_c28833_g1_i1.p1
Cyberlindnera_jadinii_XP_020068519.1
Wickerhamomyces_ciferrii_XP_011275848.1
Komagataella_phaffii_XP_002493765.1
Mucor_circinelloides_jgi75350
Rhizopus_microsporus_XP_023464014.1
Phycomyces_blakesleeanus_jgi25258
Schizosaccharomyces_octosporus_XP_013020157.1
Ceraceosorus_guamensis_XP_025366672.1
Rhodotorula_graminis_XP_018272679.1
Mixia_osmundae_XP_014571060.1
Sporobolomyces_roseus_jgi9160
Rhizophagus_irregularis_XP_025170552.1
Lobosporangium_transversale_XP_021883542.1
Batrachochytrium_dendrobatidis_jgi11553
Spizellomyces_punctatus_XP_016610595.1
Sphaeroforma_arctica_XP_014157354.1
Orbicella_faveolata_XP_020603866.1
Pocillopora_damicornis_XP_027060523.1
Stylophora_pistillata_XP_022805735.1
Capsaspora_owczarzaki_XP_004364926.2
Acanthoeca_sp_MMETSP0105_c1468_g1_i1.p1
Monosiga_brevicollis_XP_001744766.1
Salpingoeca_rosetta_XP_004991997.1
Chromera_velia_MMETSP0290_c49553_g1_i1.p1
Planctomycetes_bacterium_Pla22_WP_146515334.1
Mariniblastus_fucicola_WP_075081890.1
Verrucomicrobium_sp._BvORR034_WP_050026988.1
Fibrobacter_sp._UWEL_WP_073225483.1
NA_WP_109734694.1
Eutreptiella_gymnastica-like_MMETSP0809_c29166_g1_i1.p1
Capsulimonas_corticalis_WP_119322124.1
Lentisphaera_araneosa_WP_007276806.1
Pedosphaera_parvula_WP_007414941.1
Desulfopila_sp._IMCC35004_WP_136796324.1
Lysinibacillus_manganicus_WP_036189902.1
Spirochaeta_cellobiosiphila_WP_028973821.1
Eubacterium_sp._AF177_WP_117909823.1
Blautia_sp._AF1440_WP_118719636.1
NA_WP_117801388.1
bacterium_1XD892_WP_129178962.1
NA_WP_120435762.1
Hypnocyclicus_thermotrophus_WP_134112166.1
Candidatus_Marispirochaeta_associata_WP_069895808.1
Marispirochaeta_aestuarii_WP_083050768.1
Alexandrium_margalefi_MMETSP0661_c27119_g1_i1.p2
Pyrodinium_bahamense_MMETSP0796_c66589_g1_i1.p1
Polarella_glacialis_MMETSP1440_c5830_g1_i1.p1
Mesodinium_pulex_MMETSP0467_c82900_g1_i1.p2
Karlodinium_micrum_MMETSP1016_c95975_g1_i1.p1
Chlamydomonas_leiostraca_MMETSP1391_c16466_g1_i1.p1
Volvox_carteri_XP_002956910.1
Dunaliella_tertiolecta_MMETSP1126_c8943_g1_i1.p1
Pterosperma_sp_MMETSP1438_c32713_g1_i1.p1
Asparagus_officinalis_XP_020241208.1
Oryza_sativa_XP_015639697.1
Helianthus_annuus_XP_022027445.1
Lupinus_angustifolius_XP_019442988.1
Cyanidioschyzon_merolae_CmerSOOS_G2748.1
Strombidinopsis_acuminatum_MMETSP0126_c79426_g1_i1.p1
Phaeocystis_antarctica_MMETSP1100_c9311_g1_i1.p1
Chrysochromulina_rotalis_MMETSP0287_c29984_g1_i1.p1
Prymnesium_parvum_MMETSP0006_c11643_g1_i1.p2
Exanthemachrysis_gayraliae_MMETSP1464_c1191_g1_i1.p2
Gloeochaete_wittrockiana_MMETSP1089_c49751_g1_i1.p1
Cyanophora_paradoxa_tig00020592_g11637.t1
Cyanoptyche_gloeocystis_MMETSP1086_c6830_g1_i1.p1
Cryptomonas_paramecium_MMETSP0038_c20289_g1_i1.p1
Chroomonas_mesostigmaticacf_MMETSP0047_c37192_g1_i1.p1
Rhodosorus_marinus_MMETSP0315_c5791_g1_i1.p1
Compsopogon_coeruleus_MMETSP0312_c1793_g1_i1.p2
Timspurckia_oligopyrenoides_MMETSP1172_c7724_g1_i1.p1
Myrionecta_rubra_MMETSP0798_c55351_g1_i1.p1
Hanusia_phi_MMETSP1048_c12371_g1_i1.p1
Guillardia_theta_CCMP2712_jgi85755
Tiarina_fusus_MMETSP0472_c162_g1_i1.p1
Chaetoceros_affinis_MMETSP0090_c2824_g1_i1.p1
Aplanochytrium_sp_MMETSP0954_c5774_g1_i1.p4
Aplanochytrium_kerguelense_jgi105549
Thraustochytrium_sp_MMETSP0199_c17716_g1_i1.p1
Bigelowiella_natans_MMETSP1358_c25516_g1_i1.p1
Chlorarachnion_reptans_MMETSP0109_c19697_g1_i1.p1
Lotharella_globosa_MMETSP0041_c12342_g1_i1.p1
Gymnochlora_sp_MMETSP0110_c16849_g1_i1.p1
Stygamoeba_regulata_MMETSP0447_c16300_g1_i1.p1
MSTRG.7507.1.p1
Paulinella_chromatophora_CCAC0185_nuclear___scaffold4966-m45228
Candiadtus_Scalindua_japonica_WP_096894877.1
Candidatus_Brocadiaceae_bacterium_S225_WP_034405268.1
Desulfamplus_magnetovallimortis_WP_080801155.1
NA_WP_014958199.1
Desulfobacterium_autotrophicum_WP_041274244.1
Verrucomicrobiae_bacterium_DG1235_WP_008104024.1
Chryseobacterium_sp._ERMR1_04_WP_054511592.1
Sphingobacterium_sp._2c3_WP_116776569.1
Methanothermus_fervidus_WP_013414237.1
Bryobacter_aggregatus_WP_031495927.1
Candidatus_Sulfopaludibacter_sp._SbA3_WP_105502777.1
Luteitalea_pratensis_WP_110174233.1
1.51

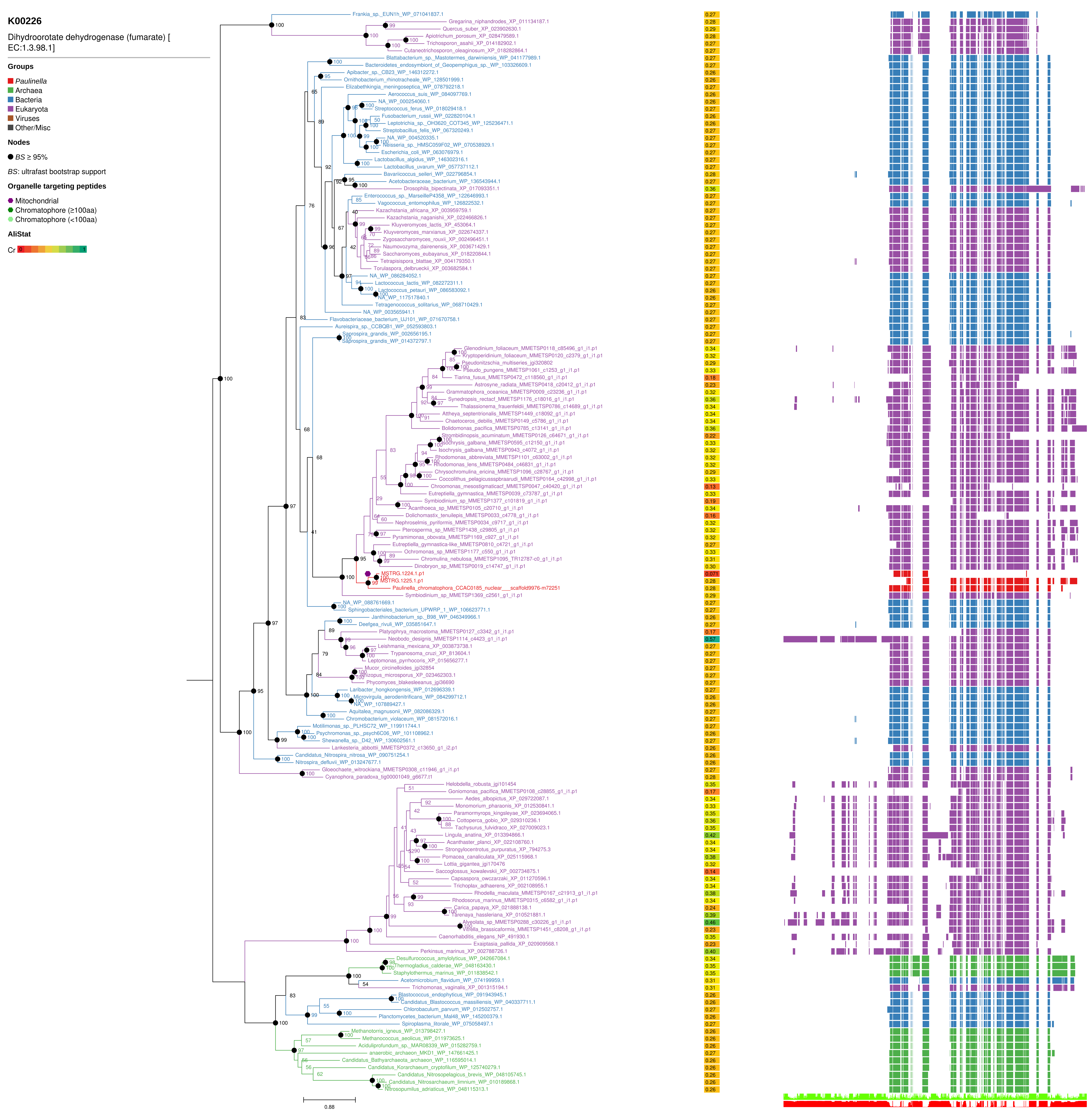
K00226
Dihydroorotate dehydrogenase (fumarate) [EC:1.3.98.1]
Groups
Paulinella
Archaea
Bacteria
Eukaryota
Viruses
Other/Misc
Nodes
BS ≥ 95%
BS: ultrafast bootstrap support
Organelle targeting peptides
Mitochondrial
Chromatophore (≥100aa)
Chromatophore (<100aa)
AliStat
Cr
0.88

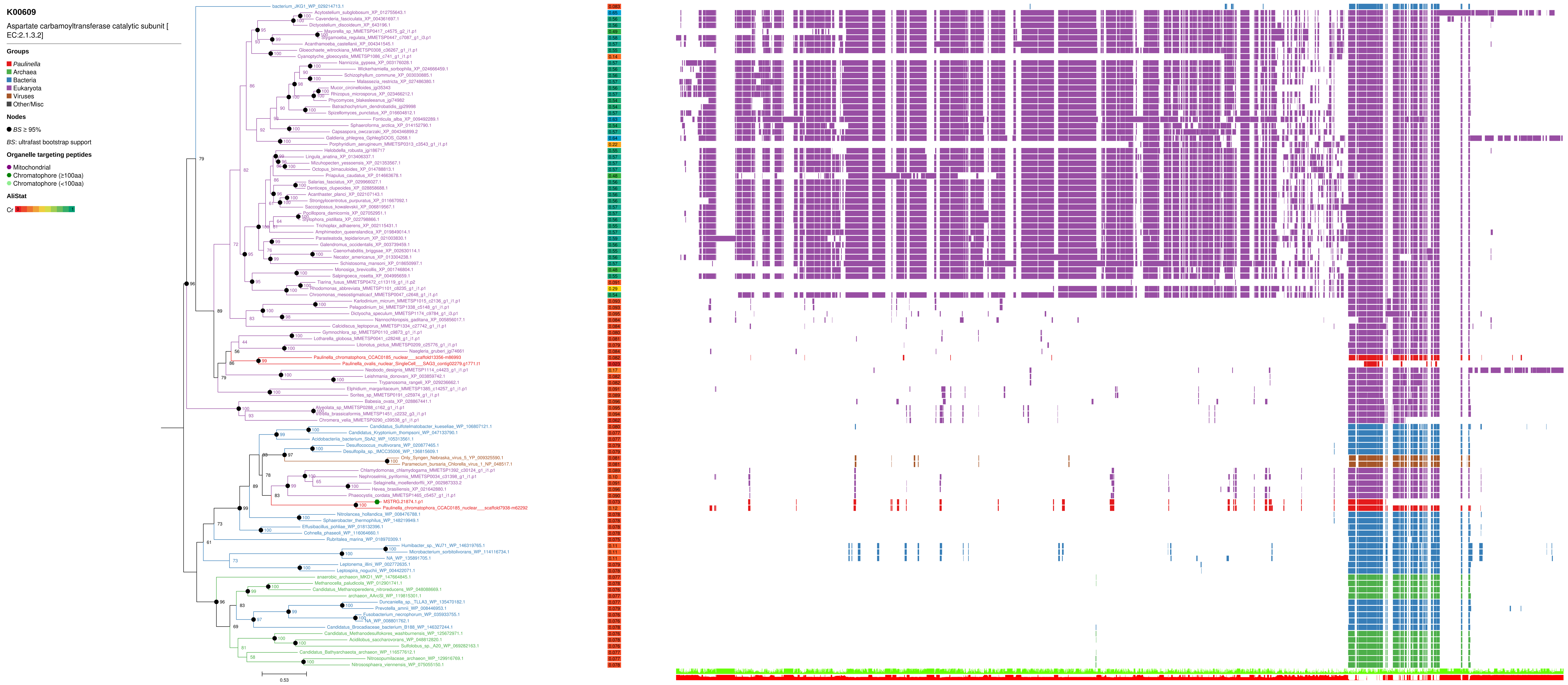

K00609
Aspartate carbamoyltransferase catalytic subunit [EC:2.1.3.2]
Groups
Paulinella
Archaea
Bacteria
Eukaryota
Viruses
Other/Misc
Nodes
BS ≥ 95%
BS: ultrafast bootstrap support
Organelle targeting peptides
Mitochondrial
Chromatophore (≥100aa)
Chromatophore (<100aa)
AliStat
Cr
0.53

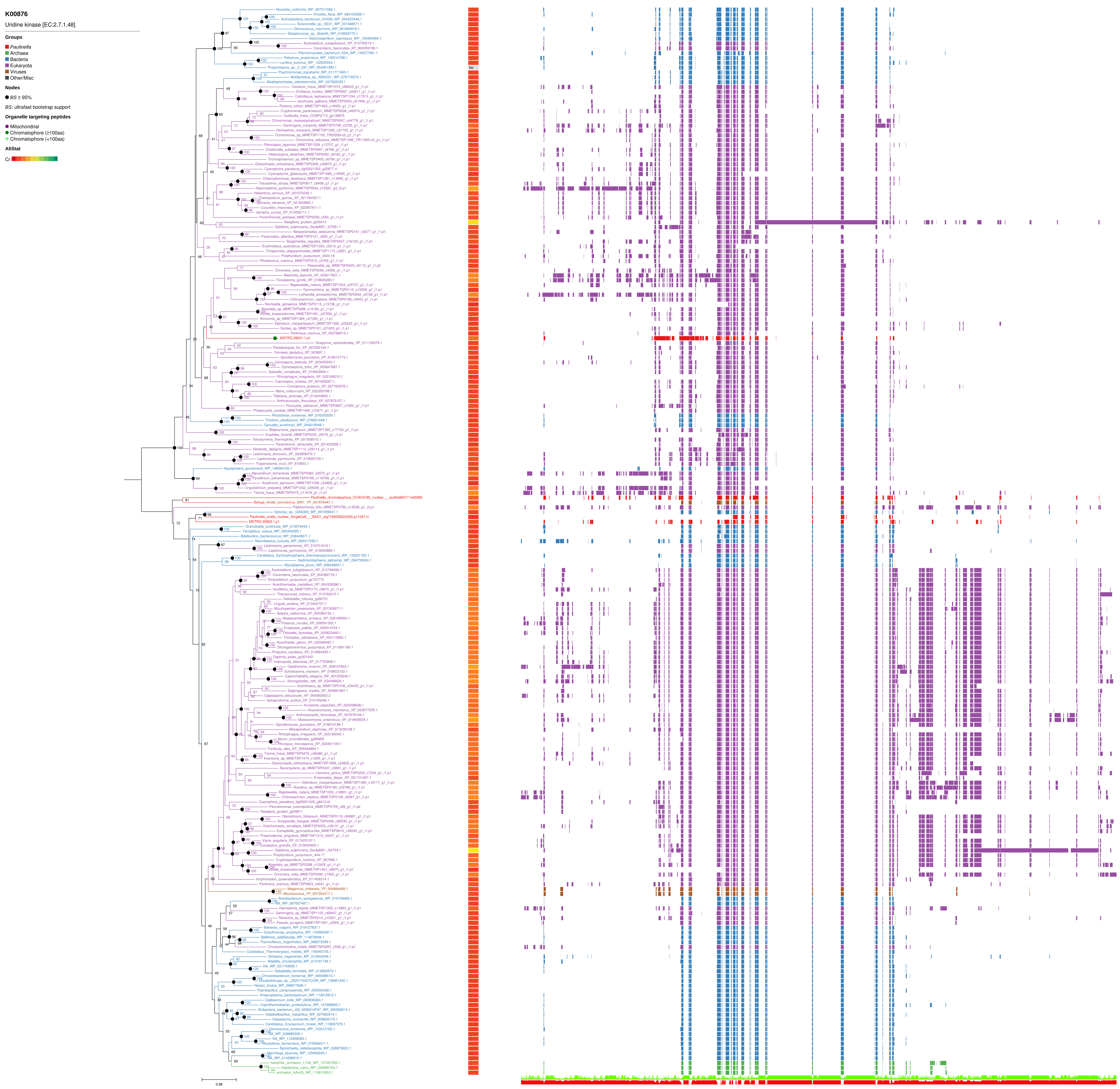
K00876
Uridine kinase [EC:2.7.1.48]
Groups
Paulinella
Archaea
Bacteria
Eukaryota
Viruses
Other/Misc
Nodes
BS ≥ 95%
BS: ultrafast bootstrap support
Organelle targeting peptides
Mitochondrial
Chromatophore (≥100aa)
Chromatophore (<100aa)
AliStat
Cr
0.98

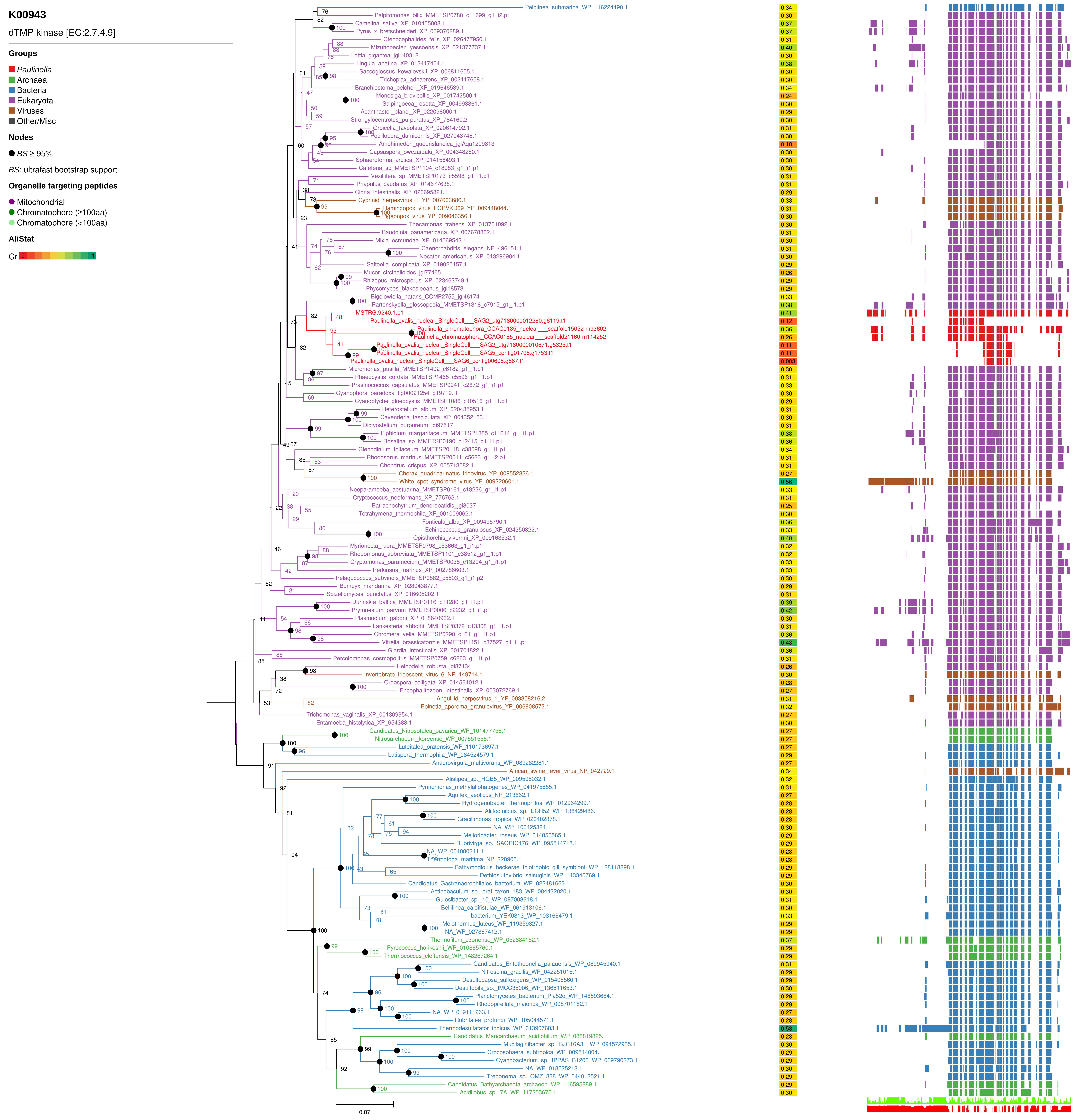

K00943
dTMP kinase [EC:2.7.4.9]
Groups
Paulinella
Archaea
Bacteria
Eukaryota
Viruses
Other/Misc
Nodes
BS ≥ 95%
BS: ultrafast bootstrap support
Organelle targeting peptides
Mitochondrial
Chromatophore (≥100aa)
Chromatophore (<100aa)
AliStat
Cr
Pelolinea_submarina_WP_116224490.1
Palpitomonas_bilix_MMETSP0780_c11699_g1_i2.p1
Camelina_sativa_XP_010455008.1
Pyrus_x_bretschneideri_XP_009370289.1
Ctenocephalides_felis_XP_026477950.1
Mizuhopecten_yessoensis_XP_021377737.1
Lottia_gigantea_jgi140318
Lingula_anatina_XP_013417404.1
Saccoglossus_kowalevskii_XP_006811655.1
Trichoplax_adhaerens_XP_002117658.1
Branchiostoma_belcheri_XP_019646589.1
Monosiga_brevicollis_XP_001742500.1
Salpingoeca_rosetta_XP_004993861.1
Acanthaster_planci_XP_022098000.1
Strongylocentrotus_purpuratus_XP_784160.2
Orbicella_faveolata_XP_020614792.1
Pocillopora_damicornis_XP_027048748.1
Amphimedon_queenslandica_jgiAqu1209813
Capsaspora_owczarzaki_XP_004348250.1
Sphaeroforma_arctica_XP_014156493.1
Cafeteria_sp_MMETSP1104_c18983_g1_i1.p1
Vexillifera_sp_MMETSP0173_c5598_g1_i1.p1
Priapulus_caudatus_XP_014677638.1
Ciona_intestinalis_XP_026695821.1
Cyprinid_herpesvirus_1_YP_007003686.1
Flamingopox_virus_FGPVKD09_YP_009448044.1
Pigeonpox_virus_XP_009046356.1
Thecamonas_trahens_XP_013761092.1
Baudoinia_panamericana_XP_007678862.1
Mixia_osmundae_XP_014569543.1
Caenorhabditis_elegans_NP_496151.1
Necator_americanus_XP_013296904.1
Saitoella_complicata_XP_019025157.1
Mucor_circinelloides_jgi77465
Rhizopus_microsporus_XP_023462749.1
Phycomyces_blakesleeanus_jgi18573
Bigelowiella_natans_CCMP2755_jgi46174
Partenskyella_glossopodia_MMETSP1318_c7915_g1_i1.p1
MSTRG.9240.1.p1
Paulinella_ovalis_nuclear_SingleCell___SAG2_utg7180000012280.g6119.t1
Paulinella_chromatophora_CCAC0185_nuclear___scaffold15052-m93602
Paulinella_chromatophora_CCAC0185_nuclear___scaffold21160-m114252
Paulinella_ovalis_nuclear_SingleCell___SAG2_utg7180000010671.g5325.t1
Paulinella_ovalis_nuclear_SingleCell___SAG5_contig01795.g1753.t1
Paulinella_ovalis_nuclear_SingleCell___SAG6_contig00608.g567.t1
Micromonas_pusilla_MMETSP1402_c6182_g1_i1.p1
Phaeocystis_cordata_MMETSP1465_c5596_g1_i1.p1
Prasinococcus_capsulatus_MMETSP0941_c2672_g1_i1.p1
Cyanophora_paradoxa_tig00021254_g19719.t1
Cyanoptyche_gloeocystis_MMETSP1086_c10516_g1_i1.p1
Heterostelium_album_XP_020435953.1
Cavenderia_fasciculata_XP_004352153.1
Dictyostelium_purpureum_jgi97517
Elphidium_margaritaceum_MMETSP1385_c11614_g1_i1.p1
Rosalina_sp_MMETSP0190_c12415_g1_i1.p1
Glenodinium_foliaceum_MMETSP0118_c38098_g1_i1.p1
Rhodosorus_marinus_MMETSP0011_c5623_g1_i2.p1
Chondrus_crispus_XP_005713082.1
Cherax_quadricarinatus_iridovirus_YP_009552336.1
White_spot_syndrome_virus_YP_009220601.1
Neoparamoeba_aestuarina_MMETSP0161_c18226_g1_i1.p1
Cryptococcus_neoformans_XP_776763.1
Batrachochytrium_dendrobatidis_jgi8037
Tetrahymena_thermophila_XP_001009062.1
Fonticula_alba_XP_009495790.1
Echinococcus_granulosus_XP_024350322.1
Opisthorchis_viverrini_XP_009163532.1
Myrionecta_rubra_MMETSP0798_c53663_g1_i1.p1
Rhodomonas_abbreviata_MMETSP1101_c38512_g1_i1.p1
Cryptomonas_paramecium_MMETSP0038_c13204_g1_i1.p1
Perkinsus_marinus_XP_002786603.1
Pelagococcus_subviridis_MMETSP0882_c5503_g1_i1.p2
Bombyx_mandarina_XP_028043877.1
Spizellomyces_punctatus_XP_016605202.1
Durinskia_baltica_MMETSP0116_c11280_g1_i1.p1
Prymnesium_parvum_MMETSP0006_c2232_g1_i1.p1
Plasmodium_gaboni_XP_018640932.1
Lankesteria_abbottii_MMETSP0372_c13308_g1_i1.p1
Chromera_velia_MMETSP0290_c161_g1_i1.p1
Vitrella_brassicaformis_MMETSP1451_c37527_g1_i1.p1
Giardia_intestinalis_XP_001704822.1
Percolomonas_cosmopolitus_MMETSP0759_c6263_g1_i1.p1
Helobdella_robusta_jgi87434
Invertebrate_iridescent_virus_6_NP_149714.1
Ordospora_colligata_XP_014564012.1
Encephalitozoon_intestinalis_XP_003072769.1
Anguillid_herpesvirus_1_YP_003358216.2
Epinotia_aporema_granulovirus_YP_006908572.1
Trichomonas_vaginalis_XP_001309954.1
Entamoeba_histolytica_XP_654383.1
Candidatus_Nitrosotalea_bavarica_WP_101477756.1
Nitrosarchaeum_koreense_WP_007551555.1
Luteitalea_pratensis_WP_110173697.1
Lutispora_thermophila_WP_084524579.1
Anaerovirgula_multivorans_WP_089282281.1
African_swine_fever_virus_NP_042729.1
Alistipes_sp._HGB5_WP_009598032.1
Pyrinomonas_methylaliphatogenes_WP_041975885.1
Aquifex_aeolicus_NP_213662.1
Hydrogenobacter_thermophilus_WP_012964299.1
Aliifodinibius_sp._ECH52_WP_138429486.1
Gracilimonas_tropica_WP_020402878.1
NA_WP_100425324.1
Melioribacter_roseus_WP_014856565.1
Rubrivirga_sp._SAORIC476_WP_095514718.1
NA_WP_004080341.1
Thermotoga_maritima_NP_228905.1
Bathymodiolus_heckerae_thiotrophic_gill_symbiont_WP_138118898.1
Dethiosulfovibrio_salsuginis_WP_143340769.1
Candidatus_Gastranaerophilales_bacterium_WP_022481663.1
Actinobaculum_sp._oral_taxon_183_WP_084432020.1
Gulosibacter_sp._10_WP_087008618.1
Bellilinea_caldifistulae_WP_061913106.1
bacterium_YEK0313_WP_103168479.1
Meiothermus_luteus_WP_119359827.1
NA_WP_027887412.1
Thermofilum_uzonense_WP_052884152.1
Pyrococcus_horikoshii_WP_010885760.1
Thermococcus_cleftensis_WP_148267264.1
Candidatus_Entotheonella_palauensis_WP_089945940.1
Nitrospina_gracilis_WP_042251016.1
Desulfocapsa_sulfexigens_WP_015405560.1
Desulfopila_sp._IMCC35006_WP_136811653.1
Planctomycetes_bacterium_Pla52o_WP_146593664.1
Rhodopirellula_maiorica_WP_008701182.1
NA_WP_019111263.1
Rubritalea_profundi_WP_105044571.1
Thermodesulfatator_indicus_WP_013907683.1
Candidatus_Mancarchaeum_acidiphilum_WP_088819825.1
Mucilaginibacter_sp._BJC16A31_WP_094572935.1
Crocosphaera_subtropica_WP_009544004.1
Cyanobacterium_sp._IPPAS_B1200_WP_069790373.1
NA_WP_018525218.1
Treponema_sp._OMZ_838_WP_044013521.1
Candidatus_Bathyarchaeota_archaeon_WP_116595889.1
Acidilobus_sp._7A_WP_117353675.1
0.87

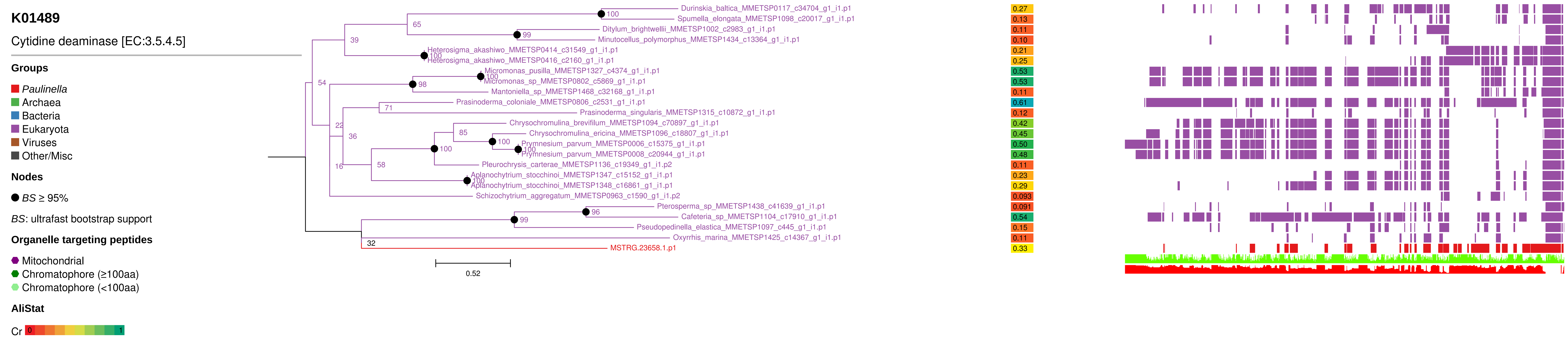

K01489
Cytidine deaminase [EC:3.5.4.5]
Groups
Paulinella
Archaea
Bacteria
Eukaryota
Viruses
Other/Misc
Nodes
BS ≥ 95%
BS: ultrafast bootstrap support
Organelle targeting peptides
Mitochondrial
Chromatophore (≥100aa)
Chromatophore (<100aa)
AliStat
Cr 0 1
Durinskia_baltica_MMETSP0117_c34704_g1_i1.p1
Spumella_elongata_MMETSP1098_c20017_g1_i1.p1
Ditylum_brightwellii_MMETSP1002_c2983_g1_i1.p1
Minutocellus_polymorphus_MMETSP1434_c13364_g1_i1.p1
Heterosigma_akashiwo_MMETSP0414_c31549_g1_i1.p1
Heterosigma_akashiwo_MMETSP0416_c2160_g1_i1.p1
Micromonas_pusilla_MMETSP1327_c4374_g1_i1.p1
Micromonas_sp_MMETSP0802_c5869_g1_i1.p1
Mantoniella_sp_MMETSP1468_c32168_g1_i1.p1
Prasinoderma_coloniale_MMETSP0806_c2531_g1_i1.p1
Prasinoderma_singularis_MMETSP1315_c10872_g1_i1.p1
Chrysochromulina_brevifilum_MMETSP1094_c70897_g1_i1.p1
Chrysochromulina_ericina_MMETSP1096_c18807_g1_i1.p1
Prymnesium_parvum_MMETSP0006_c15375_g1_i1.p1
Prymnesium_parvum_MMETSP0008_c20944_g1_i1.p1
Pleurochrysis_carterae_MMETSP1136_c19349_g1_i1.p2
Aplanochytrium_stocchinoi_MMETSP1347_c15152_g1_i1.p1
Aplanochytrium_stocchinoi_MMETSP1348_c16861_g1_i1.p1
Schizochytrium_aggregatum_MMETSP0963_c1590_g1_i1.p2
Pterosperma_sp_MMETSP1438_c41639_g1_i1.p1
Cafeteria_sp_MMETSP1104_c17910_g1_i1.p1
Pseudopedinella_elastica_MMETSP1097_c445_g1_i1.p1
Oxyrrhis_marina_MMETSP1425_c14367_g1_i1.p1
MSTRG.23658.1.p1
100
65
99
39
100
54
100
98
71
22
85
36
100
100
100
16
58
100
96
99
32
0.52
0.27
0.13
0.11
0.10
0.21
0.25
0.53
0.53
0.11
0.61
0.12
0.42
0.45
0.50
0.48
0.11
0.23
0.29
0.093
0.091
0.54
0.15
0.11
0.33

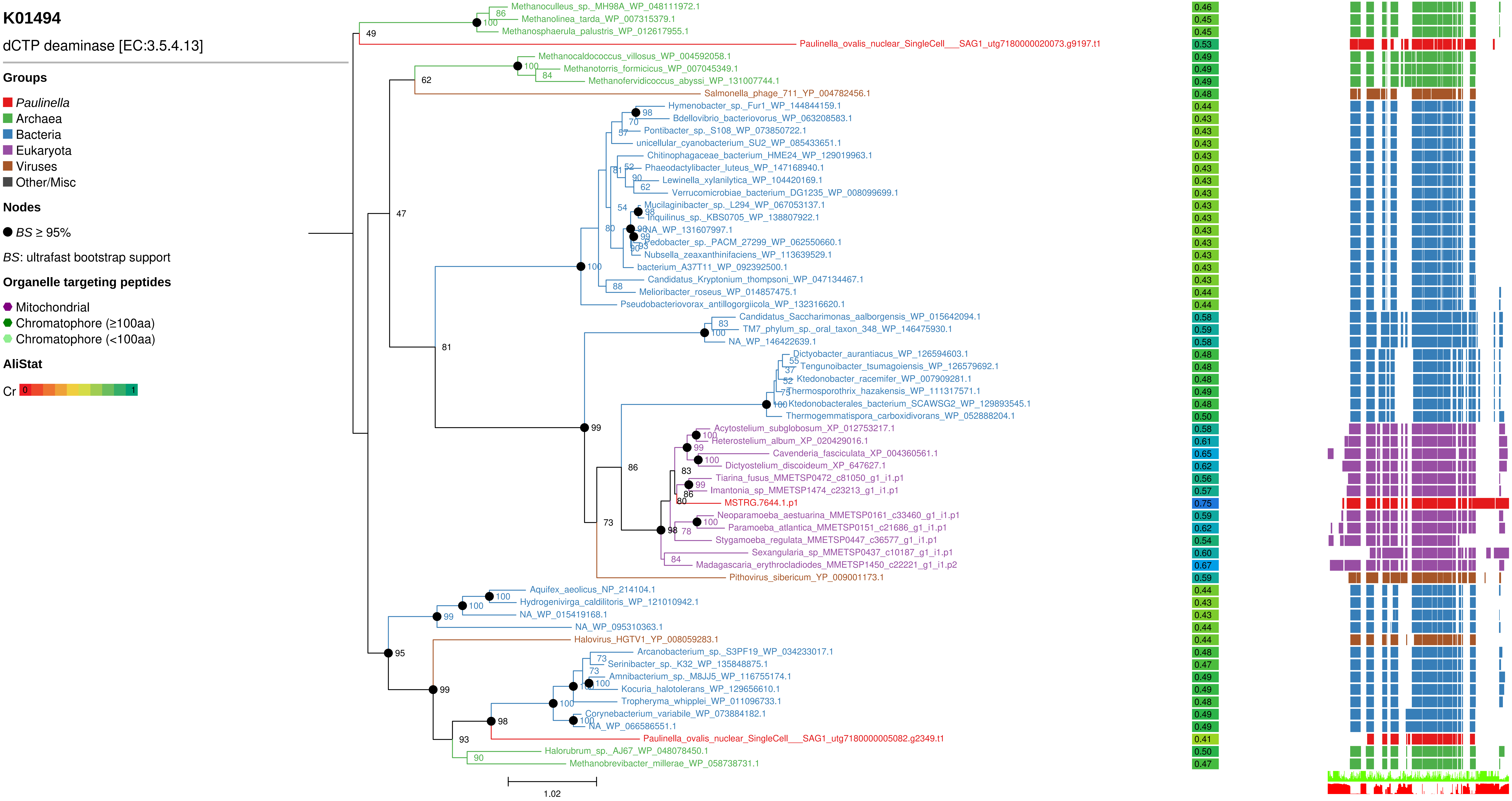

K01494
dCTP deaminase [EC:3.5.4.13]
Groups
Paulinella
Archaea
Bacteria
Eukaryota
Viruses
Other/Misc
Nodes
BS ≥ 95%
BS: ultrafast bootstrap support
Organelle targeting peptides
Mitochondrial
Chromatophore (≥100aa)
Chromatophore (<100aa)
AliStat
Cr 0 1
Methanoculleus_sp._MH98A_WP_048111972.1
Methanolinea_tarda_WP_007315379.1
Methanosphaerula_palustris_WP_012617955.1
Paulinella_ovalis_nuclear_SingleCell___SAG1_utg7180000020073.g9197.t1
Methanocaldococcus_villosus_WP_004592058.1
Methanotorris_formicicus_WP_007045349.1
Methanofervidicoccus_abyssi_WP_131007744.1
Salmonella_phage_711_YP_004782456.1
Hymenobacter_sp._Fur1_WP_144844159.1
Bdellovibrio_bacteriovorus_WP_063208583.1
Pontibacter_sp._S108_WP_073850722.1
unicellular_cyanobacterium_SU2_WP_085433651.1
Chitinophagaceae_bacterium_HME24_WP_129019963.1
Phaeodactylibacter_luteus_WP_147168940.1
Lewinella_xylanilytica_WP_104420169.1
Verrucomicrobiae_bacterium_DG1235_WP_008099699.1
Mucilaginibacter_sp._L294_WP_067053137.1
Inquilinus_sp._KBS0705_WP_138807922.1
NA_WP_131607997.1
Pedobacter_sp._PACM_27299_WP_062550660.1
Nubsella_zeaxanthinifaciens_WP_113639529.1
bacterium_A37T11_WP_092392500.1
Candidatus_Kryptonium_thompsoni_WP_047134467.1
Melioribacter_roseus_WP_014857475.1
Pseudobacteriovorax_antillogorgiicola_WP_132316620.1
Candidatus_Saccharimonas_aalborgensis_WP_015642094.1
TM7_phylum_sp._oral_taxon_348_WP_146475930.1
NA_WP_146422639.1
Dictyobacter_aurantiacus_WP_126594603.1
Tengunoibacter_tsumagoiensis_WP_126579692.1
Ktedonobacter_racemifer_WP_007909281.1
Thermosporothrix_hazakensis_WP_111317571.1
Ktedonobacterales_bacterium_SCAWSG2_WP_129893545.1
Thermogemmatispora_carboxidivorans_WP_052888204.1
Acytostelium_subglobosum_XP_012753217.1
Heterostelium_album_XP_020429016.1
Cavenderia_fasciculata_XP_004360561.1
Dictyostelium_discoideum_XP_647627.1
Tiarina_fusus_MMETSP0472_c81050_g1_i1.p1
Imantonia_sp_MMETSP1474_c23213_g1_i1.p1
MSTRG.7644.1.p1
Neoparamoeba_aestuarina_MMETSP0161_c33460_g1_i1.p1
Paramoeba_atlantica_MMETSP0151_c21686_g1_i1.p1
Stygamoeba_regulata_MMETSP0447_c36577_g1_i1.p1
Sexangularia_sp_MMETSP0437_c10187_g1_i1.p1
Madagascaria_erythrocladiodes_MMETSP1450_c22221_g1_i1.p2
Pithovirus_sibericum_YP_009001173.1
Aquifex_aeolicus_NP_214104.1
Hydrogenivirga_caldilitoris_WP_121010942.1
NA_WP_015419168.1
NA_WP_095310363.1
Halovirus_HGTV1_YP_008059283.1
Arcanobacterium_sp._S3PF19_WP_034233017.1
Serinibacter_sp._K32_WP_135848875.1
Amnibacterium_sp._M8JJ5_WP_116755174.1
Kocuria_halotolerans_WP_129656610.1
Tropheryma_whipplei_WP_011096733.1
Corynebacterium_variabile_WP_073884182.1
NA_WP_066586551.1
Paulinella_ovalis_nuclear_SingleCell___SAG1_utg7180000005082.g2349.t1
Halorubrum_sp._AJ67_WP_048078450.1
Methanobrevibacter_millerae_WP_058738731.1
1.02

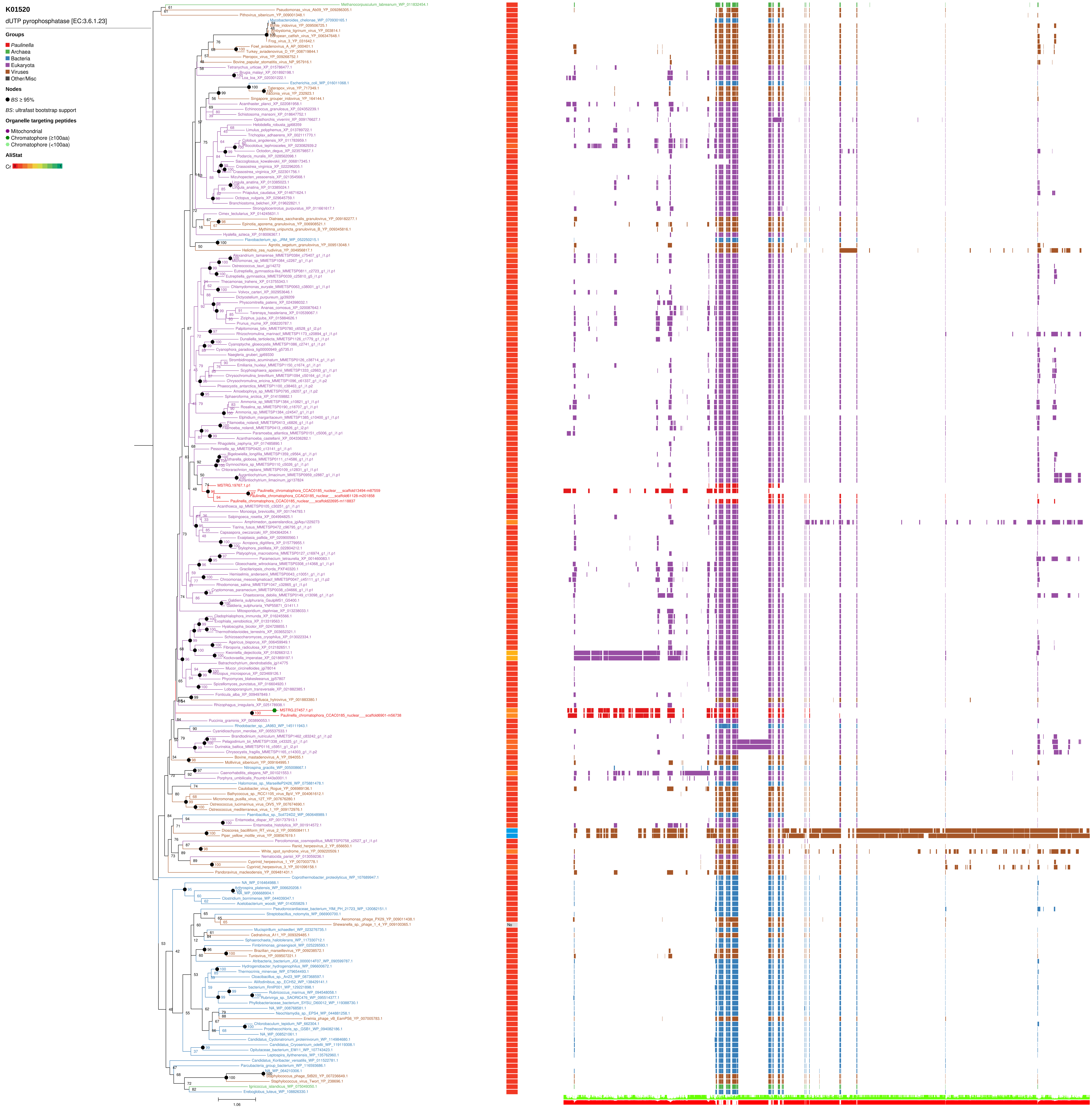
K01520
dUTP pyrophosphatase [EC:3.6.1.23]
Groups
Paulinella
Archaea
Bacteria
Eukaryota
Viruses
Other/Misc
Nodes
BS ≥ 95%
BS: ultrafast bootstrap support
Organelle targeting peptides
Mitochondrial
Chromatophore (≥100aa)
Chromatophore (<100aa)
AliStat
Cr
1.06

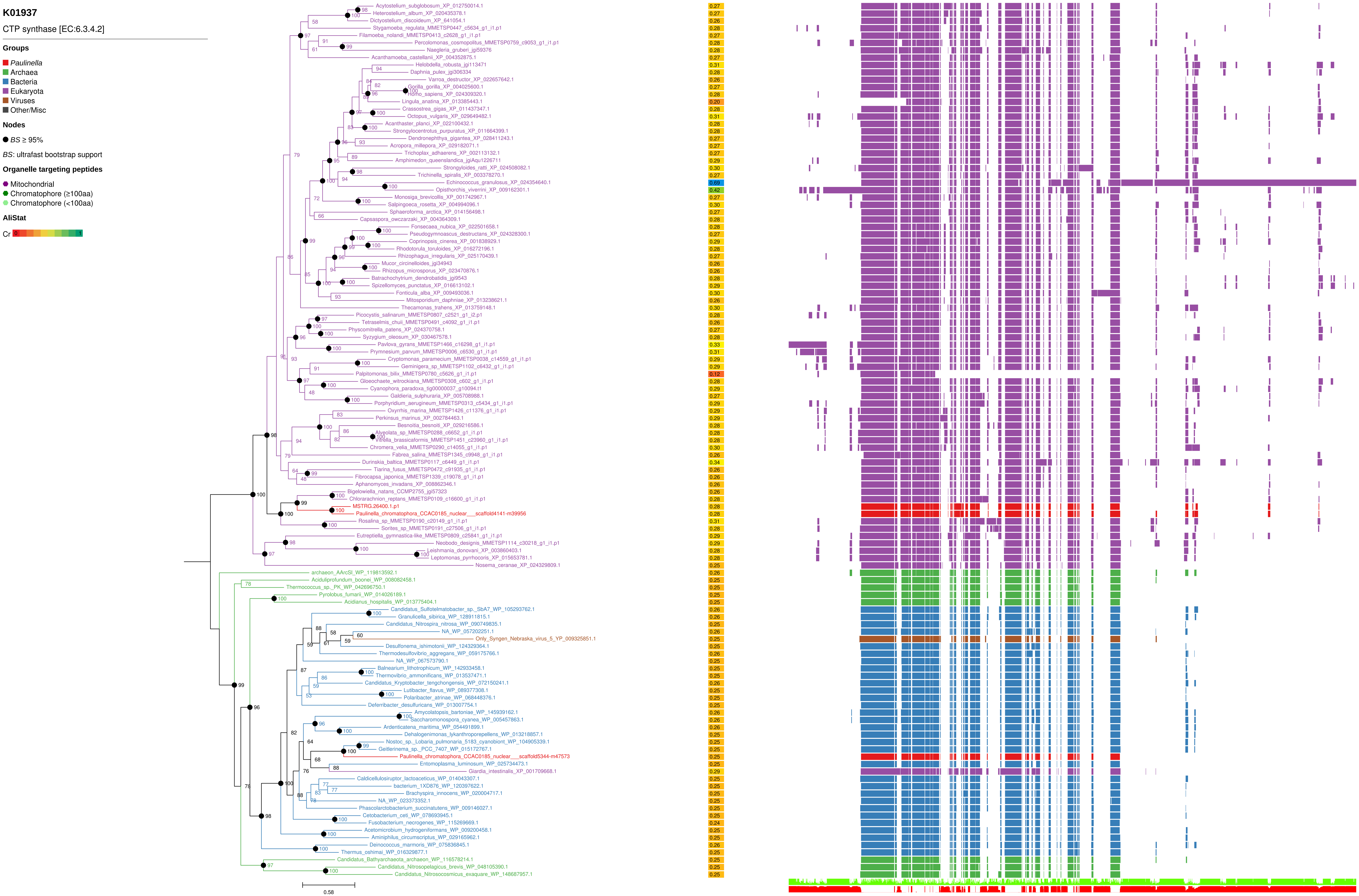
K01937
CTP synthase [EC:6.3.4.2]
Groups
Paulinella
Archaea
Bacteria
Eukaryota
Viruses
Other/Misc
Nodes
BS ≥ 95%
BS: ultrafast bootstrap support
Organelle targeting peptides
Mitochondrial
Chromatophore (≥100aa)
Chromatophore (<100aa)
AliStat
Cr 0 1
Acytostelium_subglobosum_XP_012750014.1 0.27
Heterostelium_album_XP_020435378.1 0.27
Dictyostelium_discoideum_XP_641054.1 0.26
Stygamoeba_regulata_MMETSP0447_c5634_g1_i1.p1 0.28
Filamoeba_nolandi_MMETSP0413_c2628_g1_i1.p1 0.27
Percolomonas_cosmopolitus_MMETSP0759_c9053_g1_i1.p1 0.28
Naegleria_gruberi_jgi59376 0.28
Acanthamoeba_castellanii_XP_004352875.1 0.27
Helobdella_robusta_jgi113471 0.31
Daphnia_pulex_jgi306334 0.28
Varroa_destructor_XP_022657642.1 0.26
Gorilla_gorilla_XP_004025600.1 0.27
Homo_sapiens_XP_024309320.1 0.28
Lingula_anatina_XP_013385443.1 0.20
Crassostrea_gigas_XP_011437347.1 0.28
Octopus_vulgaris_XP_029649482.1 0.31
Acanthaster_planci_XP_022100432.1 0.28
Strongylocentrotus_purpuratus_XP_011664399.1 0.28
Dendronephthya_gigantea_XP_028411243.1 0.27
Acropora_millepora_XP_029182071.1 0.27
Trichoplax_adhaerens_XP_002113132.1 0.27
Amphimedon_queenslandica_jgiAqu1226711 0.29
Strongyloides_ratti_XP_024508082.1 0.30
Trichinella_spiralis_XP_003378270.1 0.27
Echinococcus_granulosus_XP_024354640.1 0.69
Opisthorchis_viverrini_XP_009162301.1 0.42
Monosiga_brevicollis_XP_001742967.1 0.27
Salpingoeca_rosetta_XP_004994096.1 0.30
Sphaeroforma_arctica_XP_014156498.1 0.27
Capsaspora_owczarzaki_XP_004364309.1 0.28
Fonsecaea_nubica_XP_022501658.1 0.28
Pseudogymnoascus_destructans_XP_024328300.1 0.27
Coprinopsis_cinerea_XP_001838929.1 0.29
Rhodotorula_toruloides_XP_016272196.1 0.28
Rhizophagus_irregularis_XP_025170439.1 0.27
Mucor_circinelloides_jgi34943 0.26
Rhizopus_microsporus_XP_023470876.1 0.26
Batrachochytrium_dendrobatidis_jgi9543 0.28
Spizellomyces_punctatus_XP_016613102.1 0.29
Fonticula_alba_XP_009493036.1 0.30
Mitosporidium_daphniae_XP_013238621.1 0.26
Thecamonas_trahens_XP_013759148.1 0.30
Picocystis_salinarum_MMETSP0807_c2521_g1_i2.p1 0.28
Tetraselmis_chuii_MMETSP0491_c4092_g1_i1.p1 0.26
Physcomitrella_patens_XP_024370758.1 0.27
Syzygium_oleosum_XP_030467578.1 0.28
Pavlova_gyrans_MMETSP1466_c16298_g1_i1.p1 0.33
Prymnesium_parvum_MMETSP0006_c6530_g1_i1.p1 0.31
Cryptomonas_paramecium_MMETSP0038_c14559_g1_i1.p1 0.29
Geminigera_sp_MMETSP1102_c6432_g1_i1.p1 0.29
Palpitomonas_bilix_MMETSP0780_c5626_g1_i1.p1 0.12
Gloeochaete_witrockiana_MMETSP0308_c602_g1_i1.p1 0.28
Cyanophora_paradoxa_tig00000037_g10094.t1 0.29
Galdieria_sulphuraria_XP_005708988.1 0.27
Porphyridium_aerugineum_MMETSP0313_c5434_g1_i1.p1 0.29
Oxyrrhis_marina_MMETSP1426_c11376_g1_i1.p1 0.29
Perkinsus_marinus_XP_002784463.1 0.29
Besnoitia_besnoiti_XP_029216586.1 0.28
Alveolata_sp_MMETSP0288_c6652_g1_i1.p1 0.28
Vitrella_brassicaformis_MMETSP1451_c23960_g1_i1.p1 0.28
Chromera_velia_MMETSP0290_c14055_g1_i1.p1 0.30
Fabrea_salina_MMETSP1345_c9948_g1_i1.p1 0.26
Durinskia_baltica_MMETSP0117_c6449_g1_i1.p1 0.34
Tiarina_fusus_MMETSP0472_c91935_g1_i1.p1 0.26
Fibrocapsa_japonica_MMETSP1339_c19078_g1_i1.p1 0.26
Aphanomyces_invadans_XP_008862346.1 0.26
Bigelowiella_natans_CCMP2755_jgi57323 0.26
Chlorarachnion_reptans_MMETSP0109_c16600_g1_i1.p1 0.28
MSTRG.26400.1.p1 0.28
Paulinella_chromatophora_CCAC0185_nuclear___scaffold4141-m39956 0.28
Rosalina_sp_MMETSP0190_c20149_g1_i1.p1 0.31
Sorites_sp_MMETSP0191_c27506_g1_i1.p1 0.28
Eutreptiella_gymnastica-like_MMETSP0809_c25841_g1_i1.p1 0.29
Neobodo_designis_MMETSP1114_c30218_g1_i1.p1 0.29
Leishmania_donovani_XP_003860403.1 0.28
Leptomonas_pyrrhocoris_XP_015653781.1 0.28
Nosema_ceranae_XP_024329809.1 0.25
archaeon_AArcSl_WP_119813592.1 0.26
Aciduliprofundum_boonei_WP_008082458.1 0.25
Thermococcus_sp._PK_WP_042696750.1 0.25
Pyrolobus_fumarii_WP_014026189.1 0.25
Acidianus_hospitalis_WP_013775404.1 0.25
Candidatus_Sulfotelmatobacter_sp._SbA7_WP_105293762.1 0.26
Granulicella_sibirica_WP_128911815.1 0.26
Candidatus_Nitrospira_nitrosa_WP_090749835.1 0.25
NA_WP_057202251.1 0.26
Only_Syngen_Nebraska_virus_5_YP_009325851.1 0.25
Desulfonema_ishimotonii_WP_124329364.1 0.25
Thermodesulfovibrio_aggregans_WP_059175766.1 0.26
NA_WP_067573790.1 0.25
Balnearium_lithotrophicum_WP_142933458.1 0.25
Thermovibrio_ammonificans_WP_013537471.1 0.25
Candidatus_Kryptobacter_tengchongensis_WP_072150241.1 0.26
Lutibacter_flavus_WP_089377308.1 0.25
Polaribacter_atrinae_WP_068448376.1 0.25
Deferribacter_desulfuricans_WP_013007754.1 0.25
Amycolatopsis_bartoniae_WP_145939162.1 0.26
Saccharomonospora_cyanea_WP_005457863.1 0.26
Ardenticatena_maritima_WP_054491899.1 0.26
Dehalogenimonas_lykanthroporepellens_WP_013218857.1 0.25
Nostoc_sp._Lobaria_pulmonaria_5183_cyanobiont_WP_104905339.1 0.25
Geitlerinema_sp._PCC_7407_WP_015172767.1 0.25
Paulinella_chromatophora_CCAC0185_nuclear___scaffold5344-m47573 0.25
Entomoplasma_luminosum_WP_025734473.1 0.25
Giardia_intestinalis_XP_001709668.1 0.29
Caldicellulosiruptor_lactoaceticus_WP_014043307.1 0.25
bacterium_1XD876_WP_120397622.1 0.25
Brachyspira_innocens_WP_020004717.1 0.25
NA_WP_023373352.1 0.25
Phascolarctobacterium_succinatutens_WP_009146027.1 0.25
Cetobacterium_ceti_WP_078693945.1 0.25
Fusobacterium_necrogenes_WP_115269669.1 0.24
Acetomicrobium_hydrogeniformans_WP_009200458.1 0.25
Aminiphilus_circumscriptus_WP_029165962.1 0.25
Deinococcus_marmoris_WP_075836845.1 0.26
Thermus_oshimai_WP_016329877.1 0.25
Candidatus_Bathyarchaeota_archaeon_WP_116578214.1 0.25
Candidatus_Nitrosopelagicus_brevis_WP_048105390.1 0.25
Candidatus_Nitrosocosmicus_exaquare_WP_148687957.1 0.25
0.58

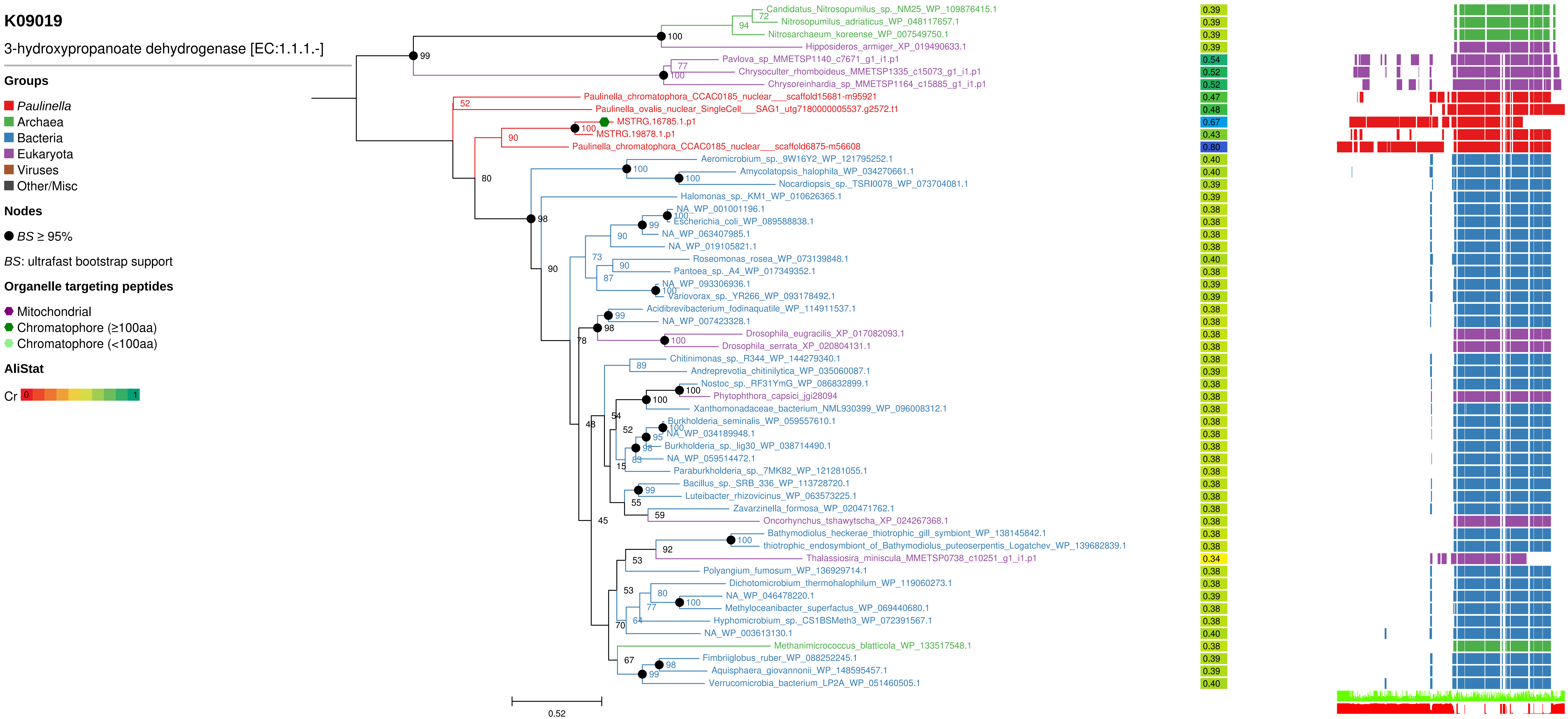
K09019
3-hydroxypropanoate dehydrogenase [EC:1.1.1.-]
Groups
Paulinella
Archaea
Bacteria
Eukaryota
Viruses
Other/Misc
Nodes
BS ≥ 95%
BS: ultrafast bootstrap support
Organelle targeting peptides
Mitochondrial
Chromatophore (≥100aa)
Chromatophore (<100aa)
AliStat
Cr 0 1
Candidatus_Nitrosopumilus_sp._NM25_WP_109876415.1
Nitrosopumilus_adriaticus_WP_048117657.1
Nitrosarchaeum_koreense_WP_007549750.1
Hipposideros_armiger_XP_019490633.1
Pavlova_sp_MMETSP1140_c7671_g1_i1.p1
Chrysoculter_rhomboideus_MMETSP1335_c15073_g1_i1.p1
Chrysoreinhardia_sp_MMETSP1164_c15885_g1_i1.p1
Paulinella_chromatophora_CCAC0185_nuclear___scaffold15681-m95921
Paulinella_ovalis_nuclear_SingleCell___SAG1_utg7180000005537.g2572.t1
MSTRG.16785.1.p1
MSTRG.19878.1.p1
Paulinella_chromatophora_CCAC0185_nuclear___scaffold6875-m56608
Aeromicrobium_sp._9W16Y2_WP_121795252.1
Amycolatopsis_halophila_WP_034270661.1
Nocardiopsis_sp._TSRI0078_WP_073704081.1
Halomonas_sp._KM1_WP_010626365.1
NA_WP_001001196.1
Escherichia_coli_WP_089588838.1
NA_WP_063407985.1
NA_WP_019105821.1
Roseomonas_rosea_WP_073139848.1
Pantoea_sp._A4_WP_017349352.1
NA_WP_093306936.1
Variovorax_sp._YR266_WP_093178492.1
Acidibrevibacterium_fodinaquatile_WP_114911537.1
NA_WP_007423328.1
Drosophila_eugracilis_XP_017082093.1
Drosophila_serrata_XP_020804131.1
Chitinimonas_sp._R344_WP_144279340.1
Andreprevotia_chitinilytica_WP_035060087.1
Nostoc_sp._RF31YmG_WP_086832899.1
Phytophthora_capsici_jgi28094
Xanthomonadaceae_bacterium_NML930399_WP_096008312.1
Burkholderia_seminalis_WP_059557610.1
NA_WP_034189948.1
Burkholderia_sp._lig30_WP_038714490.1
NA_WP_059514472.1
Paraburkholderia_sp._7MK82_WP_121281055.1
Bacillus_sp._SRB_336_WP_113728720.1
Luteibacter_rhizovicinus_WP_063573225.1
Zavarzinella_formosa_WP_020471762.1
Oncorhynchus_tshawytscha_XP_024267368.1
Bathymodiolus_heckerae_thiotrophic_gill_symbiont_WP_138145842.1
thiotrophic_endosymbiont_of_Bathymodiolus_puteoserpentis_Logatchev_WP_139682839.1
Thalassiosira_miniscula_MMETSP0738_c10251_g1_i1.p1
Polyangium_fumosum_WP_136929714.1
Dichotomicrobium_thermohalophilum_WP_119060273.1
NA_WP_046478220.1
Methyloceanibacter_superfactus_WP_069440680.1
Hyphomicrobium_sp._CS1BSMeth3_WP_072391567.1
NA_WP_003613130.1
Methanimicrococcus_blatticola_WP_133517548.1
Fimbriiglobus_ruber_WP_088252245.1
Aquisphaera_giovannonii_WP_148595457.1
Verrucomicrobia_bacterium_LP2A_WP_051460505.1
0.52

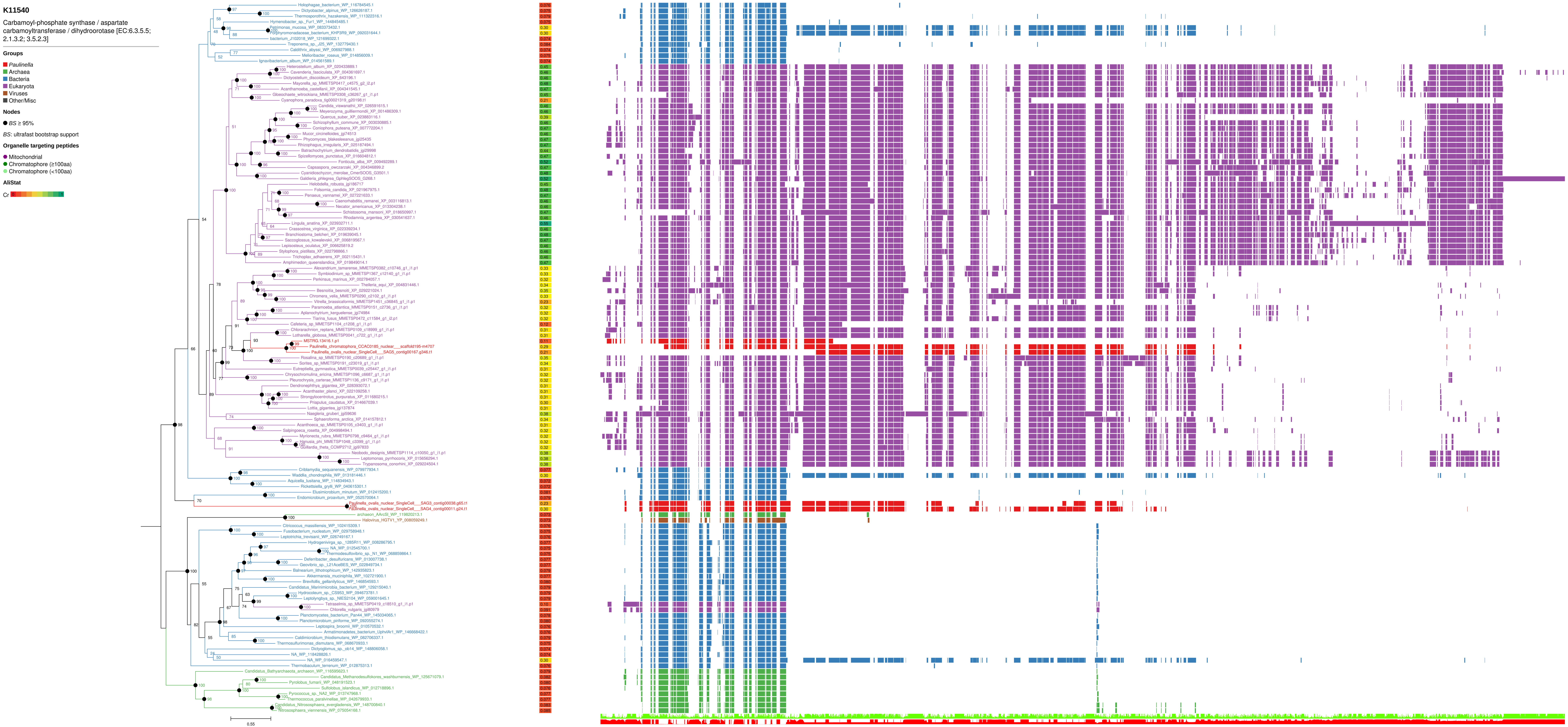
K11540
Carbamoyl-phosphate synthase / aspartate carbamoyltransferase / dihydroorotase [EC:6.3.5.5; 2.1.3.2; 3.5.2.3]
Groups
Paulinella
Archaea
Bacteria
Eukaryota
Viruses
Other/Misc
Nodes
BS ≥ 95%
BS: ultrafast bootstrap support
Organelle targeting peptides
Mitochondrial
Chromatophore (≥100aa)
Chromatophore (<100aa)
AliStat
Cr
0.55

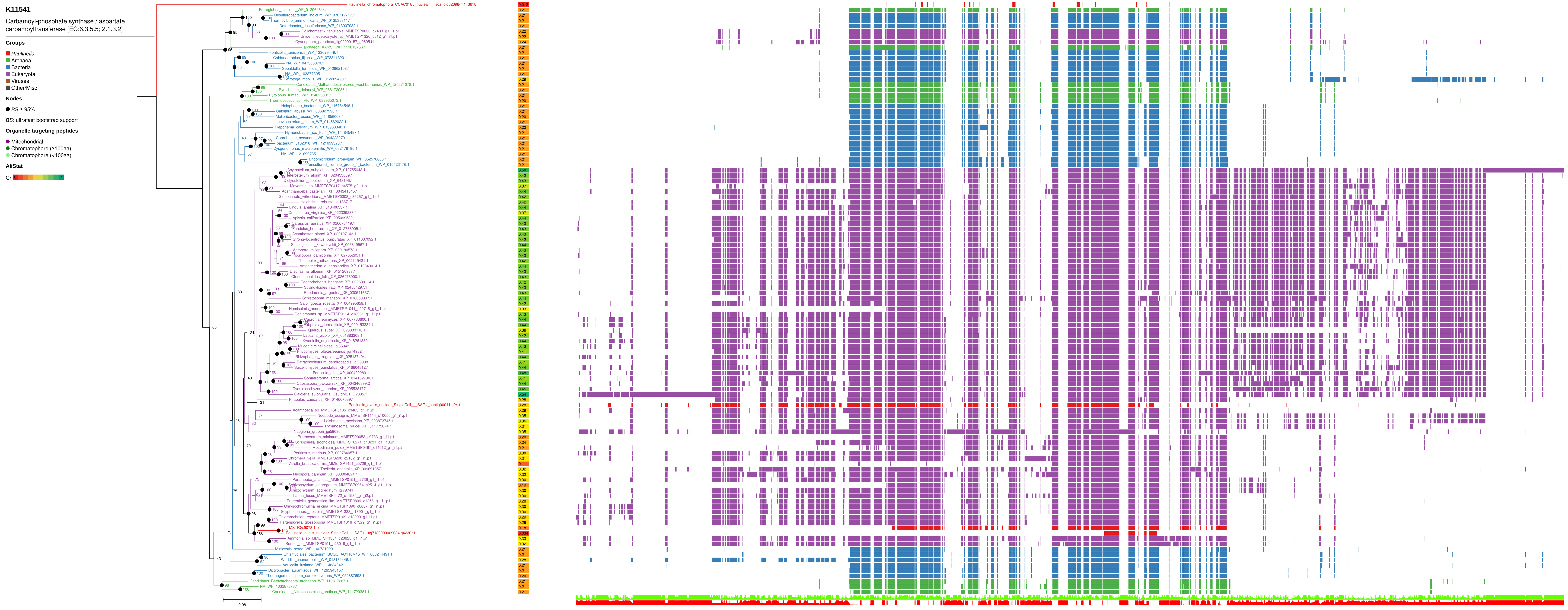
K11541
Carbamoyl-phosphate synthase / aspartate carbamoyltransferase [EC:6.3.5.5; 2.1.3.2]
Groups
Paulinella
Archaea
Bacteria
Eukaryota
Viruses
Other/Misc
Nodes
BS ≥ 95%
BS: ultrafast bootstrap support
Organelle targeting peptides
Mitochondrial
Chromatophore (≥100aa)
Chromatophore (<100aa)
AliStat
Cr
0.98

K13421

Uridine monophosphate synthetase [EC:2.4.2.10; 4.1.1.23]

Groups

- *Paulinella*
- Archaea
- Bacteria
- Eukaryota
- Viruses
- Other/Misc

Nodes

- *BS* ≥ 95%

BS: ultrafast bootstrap support

Organelle targeting peptides

- Mitochondrial
- Chromatophore (≥100aa)
- Chromatophore (<100aa)

AliStat

Cr 0 – 1

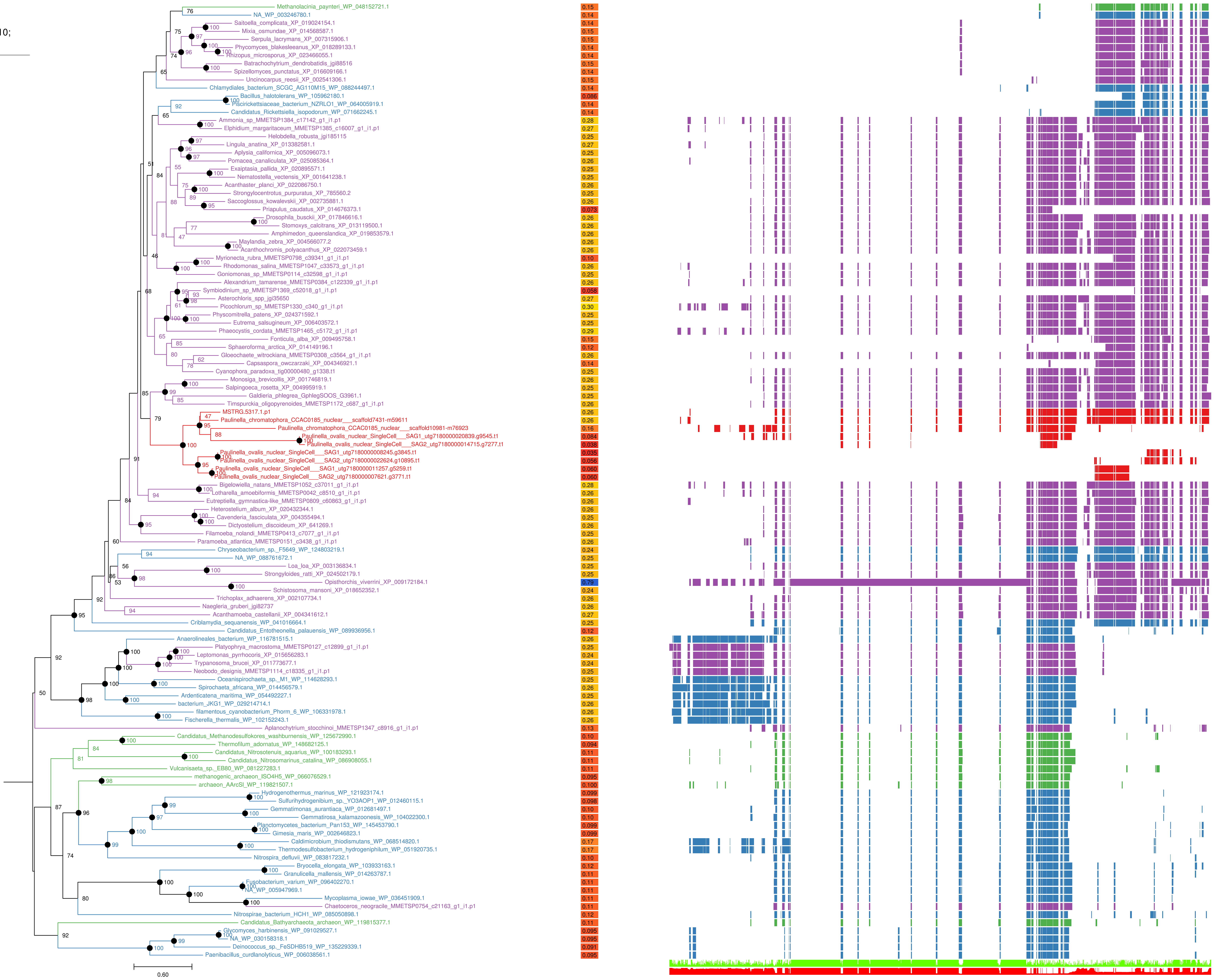

K13998

Dihydrofolate reductase / thymidylate synthase [EC:1.5.1.3; 2.1.1.45]

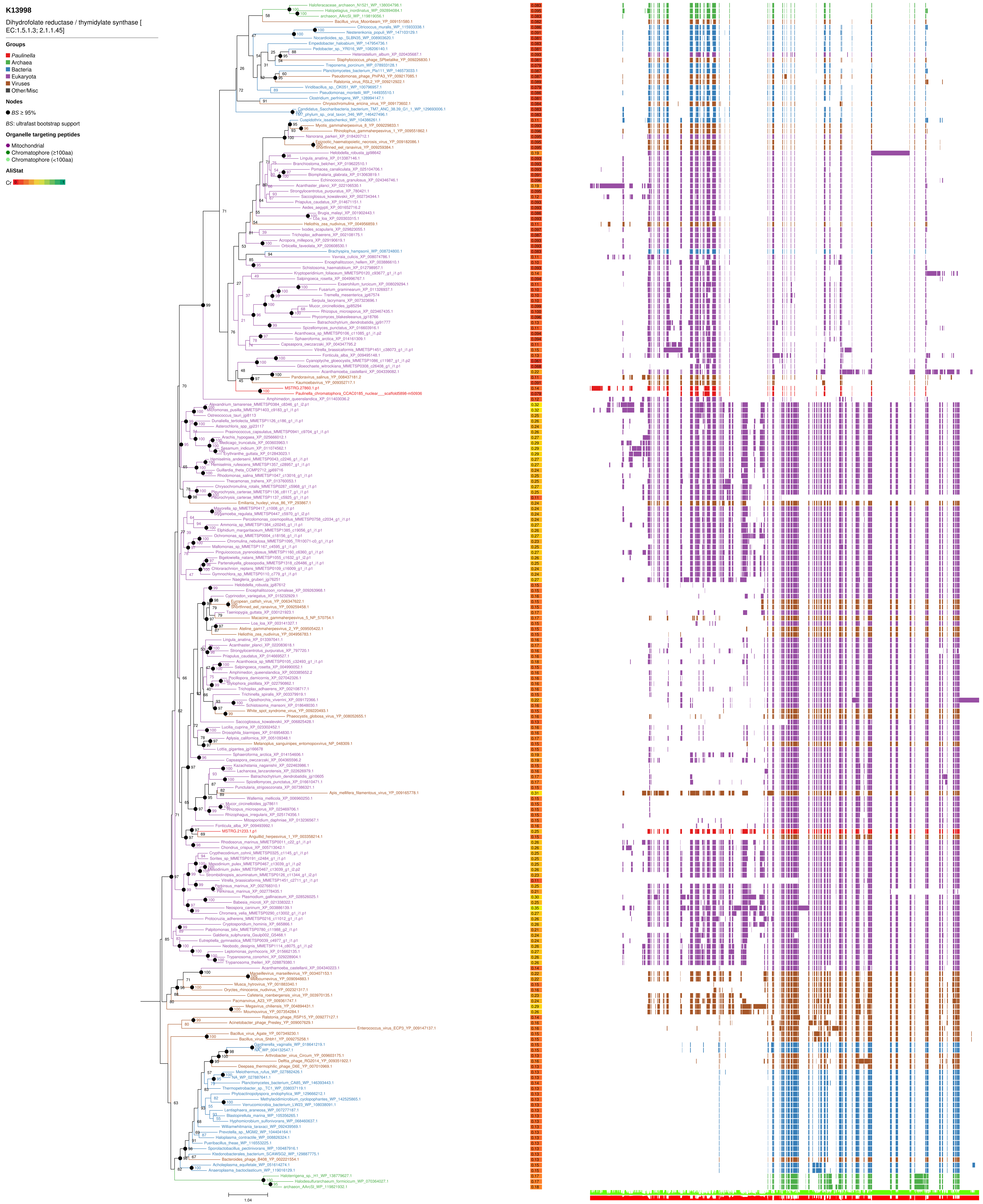

K16330

Pseudouridylate synthase / pseudouridine kinase [EC:4.2.1.70; 2.7.1.83]

Groups

- *Paulinella*
- Archaea
- Bacteria
- Eukaryota
- Viruses
- Other/Misc

Nodes

- *BS* ≥ 95%

BS: ultrafast bootstrap support

Organelle targeting peptides

- Mitochondrial
- Chromatophore (≥100aa)
- Chromatophore (<100aa)

AliStat

Cr 0 – 1

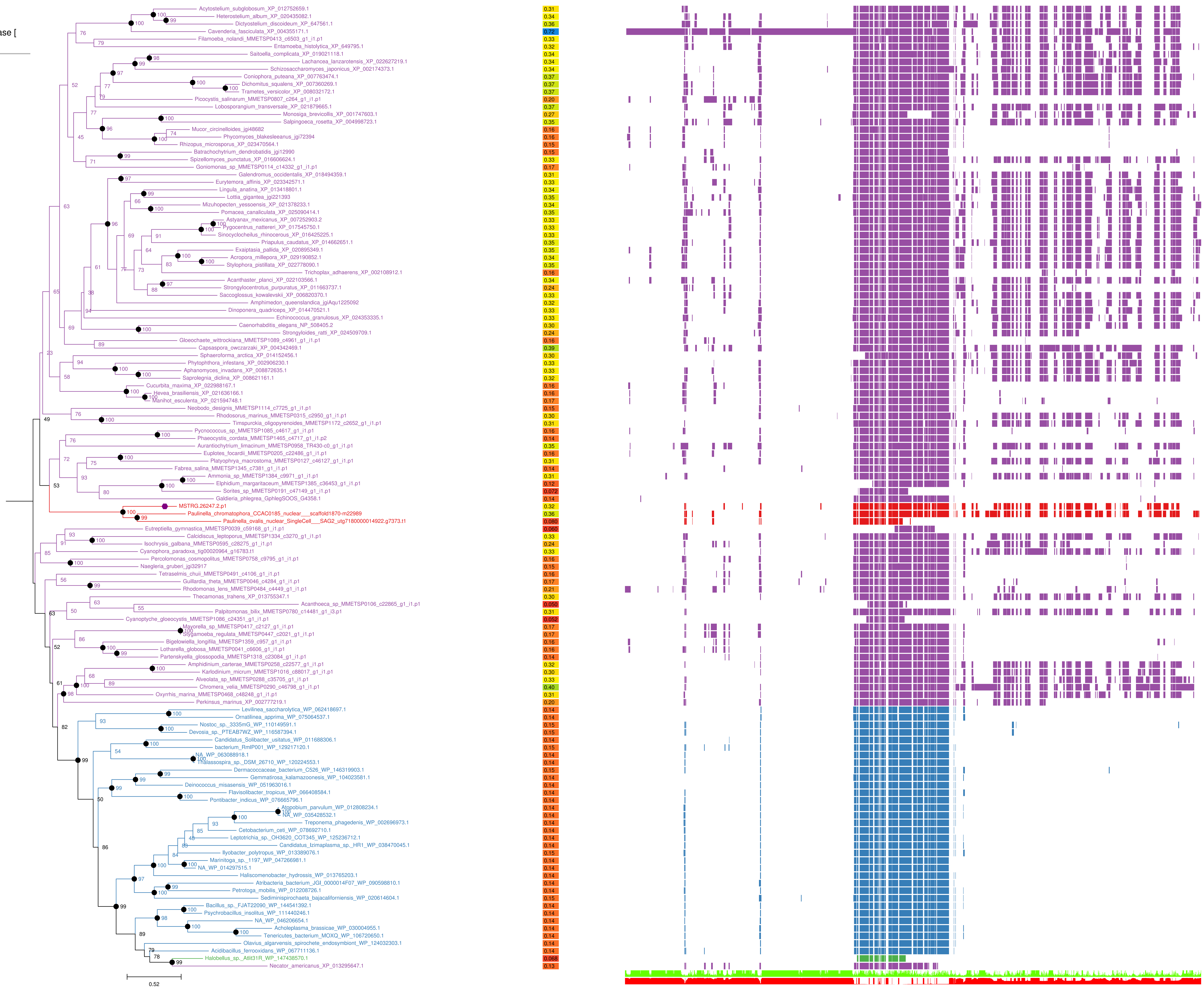

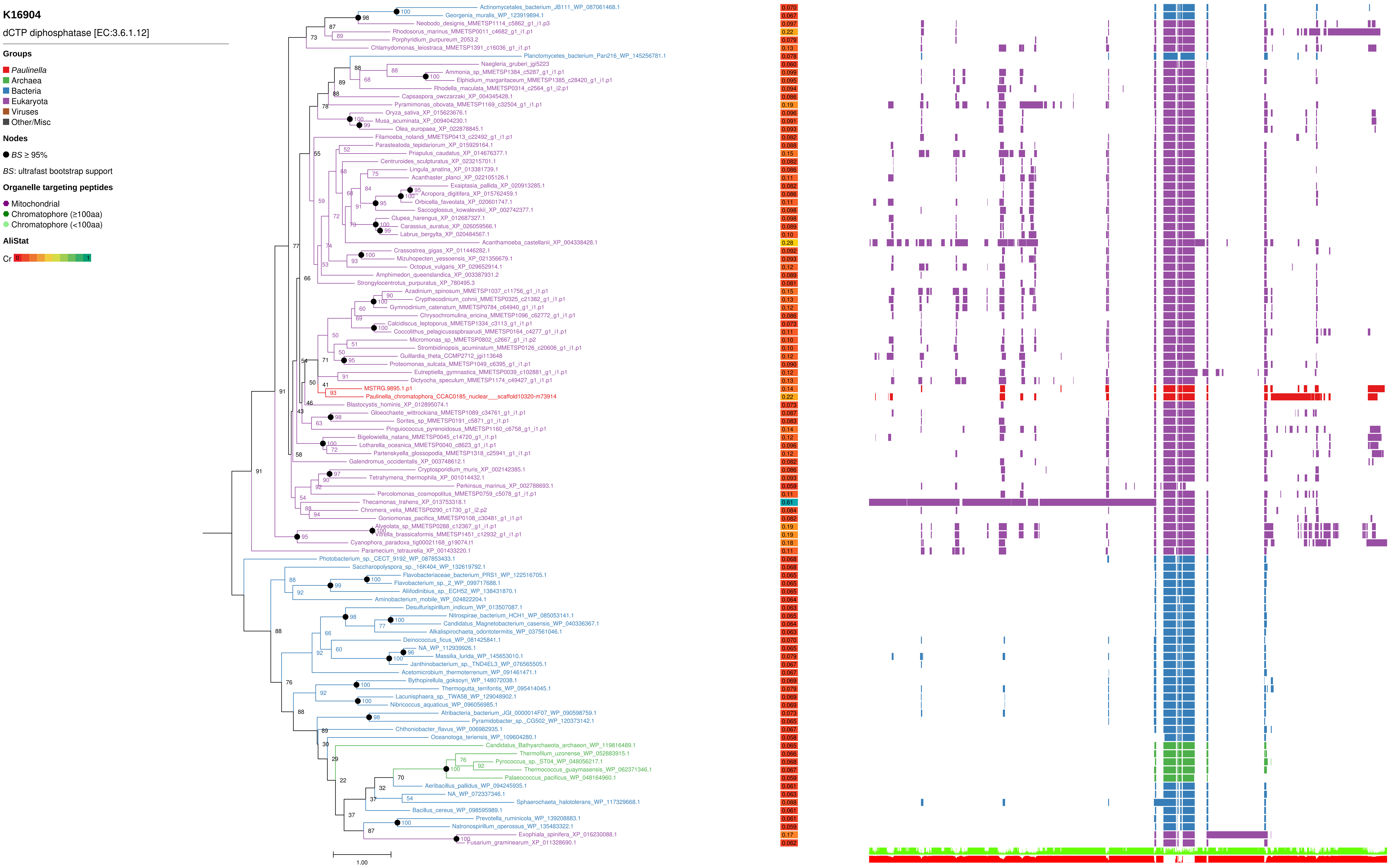

K16904
dCTP diphosphatase [EC:3.6.1.12]
Groups
Paulinella
Archaea
Bacteria
Eukaryota
Viruses
Other/Misc
Nodes
BS ≥ 95%
BS: ultrafast bootstrap support
Organelle targeting peptides
Mitochondrial
Chromatophore (≥100aa)
Chromatophore (<100aa)
AliStat
Cr
Actinomycetales_bacterium_JB111_WP_087061468.1
Georgenia_muralis_WP_123919894.1
Neobodo_designis_MMETSP1114_c5862_g1_i1.p3
Rhodosorus_marinus_MMETSP0011_c4682_g1_i1.p1
Porphyridium_purpureum_2053.2
Chlamydomonas_leiostraca_MMETSP1391_c16036_g1_i1.p1
Planctomycetes_bacterium_Pan216_WP_145256781.1
Naegleria_gruberi_jgi5223
Ammonia_sp_MMETSP1384_c5287_g1_i1.p1
Elphidium_margaritaceum_MMETSP1385_c28420_g1_i1.p1
Rhodella_maculata_MMETSP0314_c2564_g1_i2.p1
Capsaspora_owczarzaki_XP_004345428.1
Pyramimonas_obovata_MMETSP1169_c32504_g1_i1.p1
Oryza_sativa_XP_015623676.1
Musa_acuminata_XP_009404230.1
Olea_europaea_XP_022878845.1
Filamoeba_nolandi_MMETSP0413_c22492_g1_i1.p1
Parasteatoda_tepidariorum_XP_015929164.1
Priapulus_caudatus_XP_014676377.1
Centruroides_sculpturatus_XP_023215701.1
Lingula_anatina_XP_013381739.1
Acanthaster_planci_XP_022105126.1
Exaiptasia_pallida_XP_020913285.1
Acropora_digitifera_XP_015762459.1
Orbicella_faveolata_XP_020601747.1
Saccoglossus_kowalevskii_XP_002742377.1
Clupea_harengus_XP_012687327.1
Carassius_auratus_XP_026059566.1
Labrus_bergylta_XP_020484567.1
Acanthamoeba_castellanii_XP_004338428.1
Crassostrea_gigas_XP_011446282.1
Mizuhopecten_yessoensis_XP_021356679.1
Octopus_vulgaris_XP_029652914.1
Amphimedon_queenslandica_XP_003387931.2
Strongylocentrotus_purpuratus_XP_780495.3
Azadinium_spinosum_MMETSP1037_c11756_g1_i1.p1
Crypthecodinium_cohnii_MMETSP0325_c21382_g1_i1.p1
Gymnodinium_catenatum_MMETSP0784_c64940_g1_i1.p1
Chrysochromulina_ericina_MMETSP1096_c62772_g1_i1.p1
Calcidiscus_leptoporus_MMETSP1334_c3113_g1_i1.p1
Coccolithus_pelagicusssppbraarudi_MMETSP0164_c4277_g1_i1.p1
Micromonas_sp_MMETSP0802_c2667_g1_i1.p2
Strombidinopsis_acuminatum_MMETSP0126_c20606_g1_i1.p1
Guillardia_theta_CCMP2712_jgi113648
Proteomonas_sulcata_MMETSP1049_c6395_g1_i1.p1
Eutreptiella_gymnastica_MMETSP0039_c102881_g1_i1.p1
Dictyocha_speculum_MMETSP1174_c49427_g1_i1.p1
MSTRG.9895.1.p1
Paulinella_chromatophora_CCAC0185_nuclear___scaffold10320-m73914
Blastocystis_hominis_XP_012895074.1
Gloeochaete_wittrockiana_MMETSP1089_c34761_g1_i1.p1
Sorites_sp_MMETSP0191_c5871_g1_i1.p1
Pinguiococcus_pyrenoidosus_MMETSP1160_c6758_g1_i1.p1
Bigelowiella_natans_MMETSP0045_c14720_g1_i1.p1
Lotharella_oceanica_MMETSP0040_c8623_g1_i1.p1
Partenskyella_glossopodia_MMETSP1318_c25941_g1_i1.p1
Galendromus_occidentalis_XP_003748612.1
Cryptosporidium_muris_XP_002142385.1
Tetrahymena_thermophila_XP_001014432.1
Perkinsus_marinus_XP_002788693.1
Percolomonas_cosmopolitus_MMETSP0759_c5078_g1_i1.p1
Thecamonas_trahens_XP_013753318.1
Chromera_velia_MMETSP0290_c1730_g1_i2.p2
Goniomonas_pacifica_MMETSP0108_c30481_g1_i1.p1
Alveolata_sp_MMETSP0288_c12367_g1_i1.p1
Vitrella_brassicaformis_MMETSP1451_c12932_g1_i1.p1
Cyanophora_paradoxa_tig00021168_g19074.t1
Paramecium_tetraurelia_XP_001433220.1
Photobacterium_sp._CECT_9192_WP_087853433.1
Saccharopolyspora_sp._16K404_WP_132619792.1
Flavobacteriaceae_bacterium_PRS1_WP_122516705.1
Flavobacterium_sp._2_WP_099717688.1
Aliifodinibius_sp._ECH52_WP_138431870.1
Aminobacterium_mobile_WP_024822204.1
Desulfurispirillum_indicum_WP_013507087.1
Nitrospirae_bacterium_HCH1_WP_085053141.1
Candidatus_Magnetobacterium_casensis_WP_040336367.1
Alkalispirochaeta_odontotermitis_WP_037561046.1
Deinococcus_ficus_WP_081425841.1
NA_WP_112939926.1
Massilia_lurida_WP_145653010.1
Janthinobacterium_sp._TND4EL3_WP_076565505.1
Acetomicrobium_thermoterrenum_WP_091461471.1
Bythopirellula_goksoyri_WP_148072038.1
Thermogutta_terrifontis_WP_095414045.1
Lacunisphaera_sp._TWA58_WP_129048902.1
Nibricoccus_aquaticus_WP_096056985.1
Atribacteria_bacterium_JGI_0000014F07_WP_090598759.1
Pyramidobacter_sp._CG502_WP_120373142.1
Chthonobacter_flavus_WP_006982935.1
Oceanotoga_teriensis_WP_109604280.1
Candidatus_Bathyarchaeota_archaeon_WP_119816489.1
Thermofilum_uzonense_WP_052883915.1
Pyrococcus_sp._ST04_WP_048056217.1
Thermococcus_guaymasensis_WP_062371346.1
Palaeococcus_pacificus_WP_048164960.1
Aeribacillus_pallidus_WP_094245935.1
NA_WP_072337346.1
Sphaerochaeta_halotolerans_WP_117329668.1
Bacillus_cereus_WP_098595989.1
Prevotella_ruminicola_WP_139208883.1
Natronospirillum_operossus_WP_135483322.1
Exophiala_spinifera_XP_016230088.1
Fusarium_graminearum_XP_011328690.1
1.00
